# Cross-sectional data accurately model longitudinal growth in the craniofacial skeleton

**DOI:** 10.1038/s41598-023-46018-x

**Published:** 2023-11-07

**Authors:** Kevin M. Middleton, Dana L. Duren, Kieran P. McNulty, Heesoo Oh, Manish Valiathan, Richard J. Sherwood

**Affiliations:** 1 Division of Biological Sciences, University of Missouri, Columbia, MO USA; 2 Department of Orthopaedic Surgery, University of Missouri School of Medicine, Columbia, MO USA; 3 Department of Pathology and Anatomical Sciences, University of Missouri School of Medicine, Columbia, MO USA; 4 Department of Anthropology, University of Minnesota, Minneapolis, MN USA; 5 Department of Orthodontics, Arthur A. Dugoni School of Dentistry, University of the Pacific, San Francisco, CA USA; 6 Department of Orthodontics, School of Dental Medicine, Case Western Reserve University, Cleveland, OH USA

**Keywords:** Oral anatomy, Skeleton, Quantitative trait, Development

## Abstract

Dense, longitudinal sampling represents the ideal for studying biological growth. However, longitudinal samples are not typically possible, due to limits of time, prohibitive cost, or health concerns of repeat radiologic imaging. In contrast, cross-sectional samples have few such drawbacks, but it is not known how well estimates of growth milestones can be obtained from cross-sectional samples. The Craniofacial Growth Consortium Study (CGCS) contains longitudinal growth data for approximately 2000 individuals. Single samples from the CGCS for individuals representing cross-sectional data were used to test the ability to predict growth parameters in linear trait measurements separately by sex. Testing across a range of cross-sectional sample sizes from 5 to the full sample, we found that means from repeated samples were able to approximate growth rates determined from the full longitudinal CGCS sample, with mean absolute differences below 1 mm at cross-sectional sample sizes greater than ~ 200 individuals. Our results show that growth parameters and milestones can be accurately estimated from cross-sectional data compared to population-level estimates from complete longitudinal data, underscoring the utility of such datasets in growth modeling. This method can be applied to other forms of growth (e.g., stature) and to cases in which repeated radiographs are not feasible (e.g., cone-beam CT).

## Introduction

Longitudinal biological growth data provide tremendous potential for investigating details of growth trajectories and their milestones. Requiring both dedicated participant commitment and long-term financial obligations, the development of longitudinal datasets for humans is logistically complex. As a result, such studies can be limited in the range of biological variation captured. The challenge is greater for studies of skeletal growth where radiographic imaging is ideal for assessment but where repeated exposure to radiation severely limits the maximum number of observations per individual. In contrast, cross-sectional studies are logistically simple, are able to enroll a wider range of participants, and can be completed within a shorter timescale by not requiring participants to age naturally for years or decades. For these reasons, the efficiency of cross-sectional data allows for larger data sets^1^. While the benefits of cross-sectional data are clear, the question remains as to what limitations are imposed by cross-sectional analysis of growth.

To answer this question, we use examples from the field of craniofacial growth. Assessment of growth status is important in a number of craniofacial fields from orthodontics to craniofacial and maxillofacial surgery, where optimal treatment timing is critical. Studies into craniofacial growth have regularly used relatively small sample sizes of 100 or fewer individuals^2–5^. Frequently, those individuals are binned into yearly age classes (e.g., participants between their 12th and 13th birthdays are "12 year-olds") for which means or percentiles are calculated^6–10^. In an alternate approach, several studies have examined only two timepoints for an individual^11,12^, typically before and after the adolescent growth spurt. Although technically a longitudinal study, utilizing only two observation timepoints severely limits inferences regarding patterns of growth. Our own work in craniofacial growth modeling and clinical application has often highlighted the benefits of longitudinal data^13–18^, and we have had similar success modeling human stature and skeletal maturation with longitudinal data^19–24^.

When analyzed statistically, age groups, facial types, or sexes are most often statistically compared to one another via parametric independent samples *t*-tests or non-parametric *U*-tests tests to answer questions like "do sexes differ significantly in age at peak growth velocity?" or "do facial classes differ in peak growth velocity at age 12?". However, longitudinal data have also been modeled using multilevel polynomial functions^16,25–28^. In clinical practice, patient-specific measurements from one or more observations are often compared to standards from reference populations^6,8,10,29–31^ or a set of graphic standards such as the Bolton Standards^32^. In the latter, standards are used as a "target" for comparison between the patient's observed morphology and an ideal or mean configuration (i.e., a "normal" facial configuration)^10^. It remains unclear how well cross-sectional, or limited-range longitudinal data, accurately reflect the true underlying longitudinal growth pattern in the craniofacial complex. Thus, determining the agreement between age-specific craniofacial trait values estimated from longitudinal and those from cross-sectional data is of significant importance.

Herein we evaluate the ability of a large cross-sectional sample to replicate growth metrics estimated from well-characterized, similarly large longitudinal samples, and we address the impact of cross-sectional sample size on prediction ability in craniofacial growth milestones. Rather than choosing "ideal" or "normal" morphologies, the present analysis examines the full range of variability present in untreated individuals from historic growth studies. Importantly, this sample includes two sources of variation: naturally occurring phenotypic variation as well as measurement error associated with radiographs and landmark digitization.

To fully utilize the rich dataset described below, which includes a large number of serial observations for an extremely large sample of individuals, we model growth using a multilevel^28,33,34^ double-logistic growth equation^35^. This model allows for a smooth growth curve that includes two periods of rapid growth, an asymptote at growth cessation, and biologically meaningful parameters for the timing and magnitude of growth milestones. Finally, we assess the performance of these growth models using two primary applications of growth data: (1) estimation of measurement percentile intervals (i.e., growth standards) and (2) growth rates and ages at peak growth velocity. The former is used to assess a patient’s current status and future growth potential relative to the entire population (e.g., similar to standards for standing height in children). The latter can be used to determine timing relative to peak growth velocity^13^ or to determine if growth cessation has been achieved^14^, which are critical aspects of patient assessment and treatment planning.

## Methods

### Sample

All data for the present analysis derive from the Craniofacial Growth Consortium Study (CGCS). The CGCS combines data from six separate historic growth studies that span 100 years (1919–2018)^13^. Although this historic sample is primarily composed of self-identified white individuals, it is represented by populations spanning the geographic breadth of North America and has considerable time depth. We have previously shown that the different growth studies that constitute the CGCS do not differ in their overall growth parameters, and thus it is likely that that the performance of cross-sectional data relative to longitudinal data is unlikely to vary among population samples^13^. For this study, the sample includes all individuals from the CGCS with at least two cephalographs (n = 959 females; n = 980 males), yielding a data set with a median count of 9 cephalographs per individual (range: 2–22). The Institutional Review Board of the University of Missouri-Columbia approved all procedures used in this study, and all methods were performed in accordance with the relevant guidelines and regulations. Informed consent was waived by the institutional review board of University of Missouri-Columbia due to the retrospective nature of the study.

### Landmark data collection and computation of craniofacial measures

Linear traits are measured between pairs of anatomical locations (e.g., nasion and basion). To capture the two-dimensional (x, y) coordinates, we used a standard landmarking protocol. The full landmarking protocol, including study- and date-specific corrections for radiographic enlargement are provided in Sherwood et al.^13^, and we include only an overview here. Landmarks (*n* = 119) were placed using the eDigit software (Craniofacial Research Instrumentation Lab; Arthur A. Dugoni School of Dentistry, University of the Pacific) by three separate assessors and the coordinates averaged^36^. This software system includes a series of internal checks against large deviations among the three replicate landmark sets by ensuring that landmarks fall within a specified error envelope^36^.

From the full landmark set, we focused on 12 linear cephalometric traits, defined by pairs of (x, y) coordinates, broadly describing the shape of the basicranium, palate, face, and mandible. Trait values were calculated as inter-landmark distances and corrected for radiographic enlargement prior to analysis^13^. We analyzed growth trajectories and estimated growth rates for these twelve traits separately by sex and compared the performance of modeling these data longitudinally or via cross-sectional subsets.

### Statistical methods

**Figure 1:**
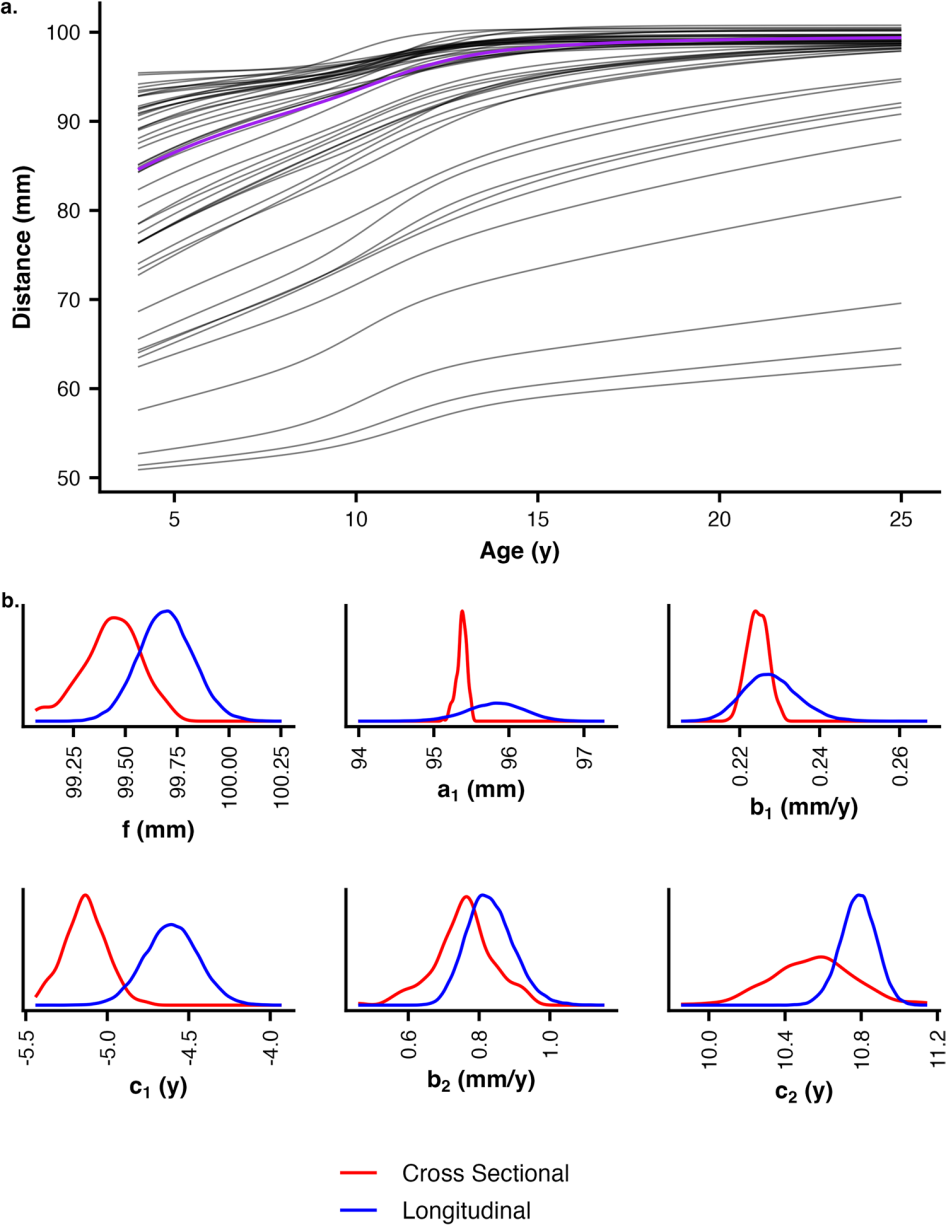
(**a**) The prior predictive check for female nasion to basion distance. The purple line represents the prediction from the mean prior for each of the six double logistic equation parameters. The gray lines represent 50 predicted growth curves using parameter values randomly sampled from the trait-specific priors. These lines give a representative set of predicted growth patterns before the data has influenced the model. (**b**) Posterior densities for parameter estimates. Six posterior distributions are compared for the longitudinal model (blue lines) and the median of the full-sample cross-sectional models (red line). *f* and *a*_1_ represent asymptotic and pre-pubertal lengths, *b*_1_ and *b*_2_ rates of growth, and *c*_1_ and *c*_2_ ages.

We modeled the growth of craniofacial traits as a function of age: *y*(*age*), using a double logistic equation proposed by Bock et al.^35^

$$y\left( {age} \right) = \frac{{a_{1} }}{{1 + e^{{ - b_{1} \left( {age - c_{1} } \right)}} }} + \frac{{f - a_{1} }}{{1 + e^{{ - b_{2} \left( {age - c_{2} } \right)}} }}$$

in which growth is modeled with two additive phases: prepubertal and adolescent. In the equation above, the *f* and *a*_*1*_ parameters represent the asymptotic and prepubertal trait size measurements, *b*_*1*_ and *b*_*2*_ rates of growth, and *c*_*1*_ and *c*_*2*_ ages at maximal growth rates. These six parameters, when applied across an *age* range, result in a continuously increasing length measurement with two periods of rapid growth (e.g., purple line in Fig. 1a). Although in the original presentation of this growth equation, which focused on modeling stature, asymptotic size at cessation (*f*) was assumed to be known a priori, here we estimate *f* from the data. Other modeling approaches have been used to estimate growth parameters, including polynomial and related spline functions, but we prefer the double logistic equation here. While other models are fit more easily, the double logistic equation has several biologically relevant characteristics that make it particularly well suited for studying growth. First, the parameter estimates have coefficients that are directly interpretable, representing distances (*f* and a_*1*_ in mm), rates (*b*_*1*_ and b_*2*_ in mm/year), or ages (*c*_*1*_ and c_2_ in years). Second, the *f* parameter represents the asymptotic size at growth cessation^13,14^. Finally, by restricting the *b*_*1*_ and *b*_*2*_ rate parameters to be positive, growth can be restricted to increase monotonically. The second two benefits are not possible with polynomial modeling.

Long-term growth studies like those that make up the CGCS are increasingly rare and unlikely to be repeated. Thus, we sought to effectively replicate the results of dense longitudinal data modeling using cross-sectional data. Using an identical analysis protocol, we compare the performance of models fit to cross-sectional subsets of the full data set to a longitudinal model fit using the full data set. The cross-sectional models analyzed in this set of comparisons varied in the number of individuals included. For this comparison, we consider the longitudinal model to represent the best approximation of the growth pattern for a trait and evaluate how well that pattern is approximated by the analysis of cross-sectional samples. The reference model was a Bayesian multilevel model, where we translated the double logistic equation into model syntax with priors for parameter estimates. That model has following specification:

$$y_{i} \sim Normal\left( {\mu_{i} ,\sigma } \right)$$

$$\mu_{i} = \frac{{a_{1} }}{{1 + e^{{ - b_{1} \left( {age_{i} - c_{1} } \right)}} }} + \frac{{f - a_{1} }}{{1 + e^{{ - b_{2} \left( {age_{i} - c_{2} } \right)}} }} + a_{ID} \left[ {ID} \right]$$

$$f \sim Normal\left( {Pr_{f} ,2} \right)$$

$$a_{1} \sim Normal\left( {Pr_{{a_{1} }} ,2} \right)$$

$$b_{1} \sim Normal\left( {Pr_{{b_{1} }} ,0.2} \right)$$

$$c_{1} \sim Normal\left( {Pr_{{c_{1} }} ,1} \right)$$

$$b_{2} \sim Normal\left( {Pr_{{b_{2} }} ,0.2} \right)$$

$$c_{2} \sim Normal\left( {Pr_{{c_{2} }} ,1} \right)$$

$$\sigma \sim Exponential\left( {0.25} \right)$$

$$a_{{ID,{ }j}} \sim Normal\left( {0,\sigma_{ID} } \right),{\text{ for }}j = 1..n_{ID}$$

$$\sigma_{ID} \sim Exponential\left( {0.25} \right).$$

In this model description, *y*_*i*_ represents the *i*th observed value of a trait for an individual with a particular *ID*. This value follows a normal distribution with a mean (μ_i_) defined by the double logistic equation. *a*_*ID*_*[ID]* represents the random intercept for each individual, which is drawn from a normal distribution with a standard deviation estimated from the data (*σ*_*ID*_) and represents an individual-specific size offset from the population-level growth pattern. The remaining lines *f* through *σ*_*ID*_ represent the priors, which are distributions for the priors for the model parameters, including trait specific values for the means of priors for each parameter (*Pr*).

In order to determine how well a cross-sectional sample can approximate a complete longitudinal sample for the goal of estimating growth milestones, we first needed to subsample the full dataset into smaller sets for which we could estimate those milestones. We generated 200 random cross-sectional subsets of the data for each of *n* = 5, 10, 20, 50, 100, 200, 300, 500, and ~ 870–881 individuals to explore the role of cross-sectional sample size in estimation of growth patterns. Importantly, each of these datasets included only a single observation per individual (i.e., mimicking a cross-sectional data set). Sample sizes for the largest cross-sectional samples varied from 870 to 881 due to differences in the observations of individual trait values across the full data set.

A model was then fit for each of these randomly generated subsets similar to the model above, but without the separate intercept for individual or its associated standard deviation. Aside from only including individuals with two or more observations, we did not impose any additional conditions on the resampling procedure, such as restriction to those with observations within a certain age range or individuals with a minimum number of measurement points. For example, individuals for whom only two observations are present will be disproportionately overrepresented in the larger samples.

Bayesian inference requires a prior for each parameter to be estimated (i.e., *f*, *a*_*1*_, *b*_*1*_, etc.). These priors define the plausible range of values for a specific parameter, which allow the sampler to efficiently search for posterior values. For example, a prior for *f* with a mean of 100 mm and standard deviation of 2 mm means that 95% of the prior weight for *f* falls between 96 and 104 mm. Priors for Bayesian models were set to be broad but mildly regularizing and determined using a genetic algorithm^13,37–39^ with the rgenoud package (version 5.9-0.3)^40^. These priors included positive constraints on the *b*_*1*_ and *b*_*2*_ rate parameters to ensure monotonically increasing length estimates. Adequacy of priors, neither too restrictive nor too wide, was assessed via prior predictive checks (Fig. 1a). These checks were visually inspected to ensure that the outcome scale of predicted distance approximated the same scale as the observed measures and that the priors were not overly restrictive to exploration of the parameter space.

Bayesian inference via Monte Carlo methods requires a mechanism for sampling from the posterior given the data, the priors, and the model. Models were estimated using Hamiltonian Monte Carlo via the *stan* statistical programming language^41,42^ with the cmdstanr package (version 0.5.0)^43^ in R (version 4.3)^44^. Models were sampled for 10,000 iterations with 50% warmup in four parallel chains. Starting values for parameters were drawn randomly from the priors separately for each chain. Model convergence was assessed by inspection of $\hat{R}$ values and rank histograms^45^. After sampling, model parameter estimates had ~ 2000–30,000 effective samples.

### Posteriors, prediction intervals, and growth rates

**Figure 2:**
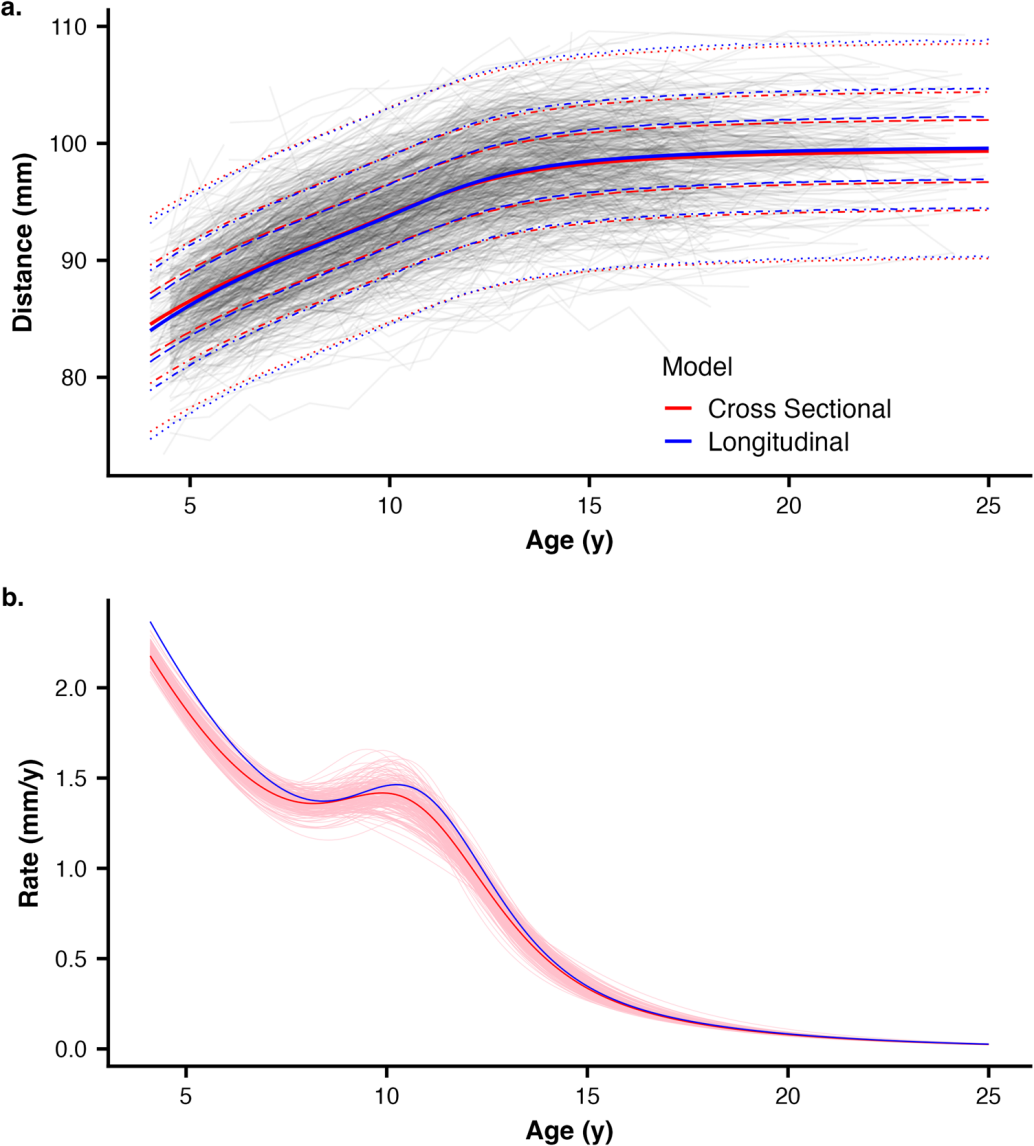
(**a**) Comparison of size percentiles. The dark red and blue lines represent median cross-sectional and longitudinal model estimates, respectively. The dotted and dashed lines represent 1st, 10th, 25th, 75th, 90th, and 99th percentiles, which are nearly identical between cross-sectional and longitudinal models as indicated by near overlap of percentiles. (**b**) Comparison of estimated growth rates as the first derivative the growth curve. As in (**a**), the dark red and blue lines represent median cross-sectional and longitudinal model estimates. All 200 estimated rates for the full-sample cross-sectional analysis are shown in pink lines, indicating general agreement between the two approaches.

Bayesian sampling results in posteriors, which include simultaneous estimates for all parameters in the model, which are summarized for presentation and comparison. While the longitudinal model had a single posterior, each of the 200 randomly resampled cross-sectional models had a separate posterior. To combine these posteriors for comparison, we created a distribution of the median parameter estimates from all the cross-sectional models for each sample size (Fig. 1b). To compare posterior predictive ability between longitudinal and cross-sectional models, which represent percentile size intervals, we calculated middle 50%, 80% and 98% quantile intervals from the posteriors (Fig. 2a), using the aggregated samples from the set of 200 random datasets. Second, we calculated the first derivative of the predicted trait size over time, the estimate of growth rate^13,16,23,46–48^ separately for each of the 200 random samples as well as for the median cross-sectional model and the longitudinal model (Fig. 2b).

### Effects of sample size

**Figure 3:**
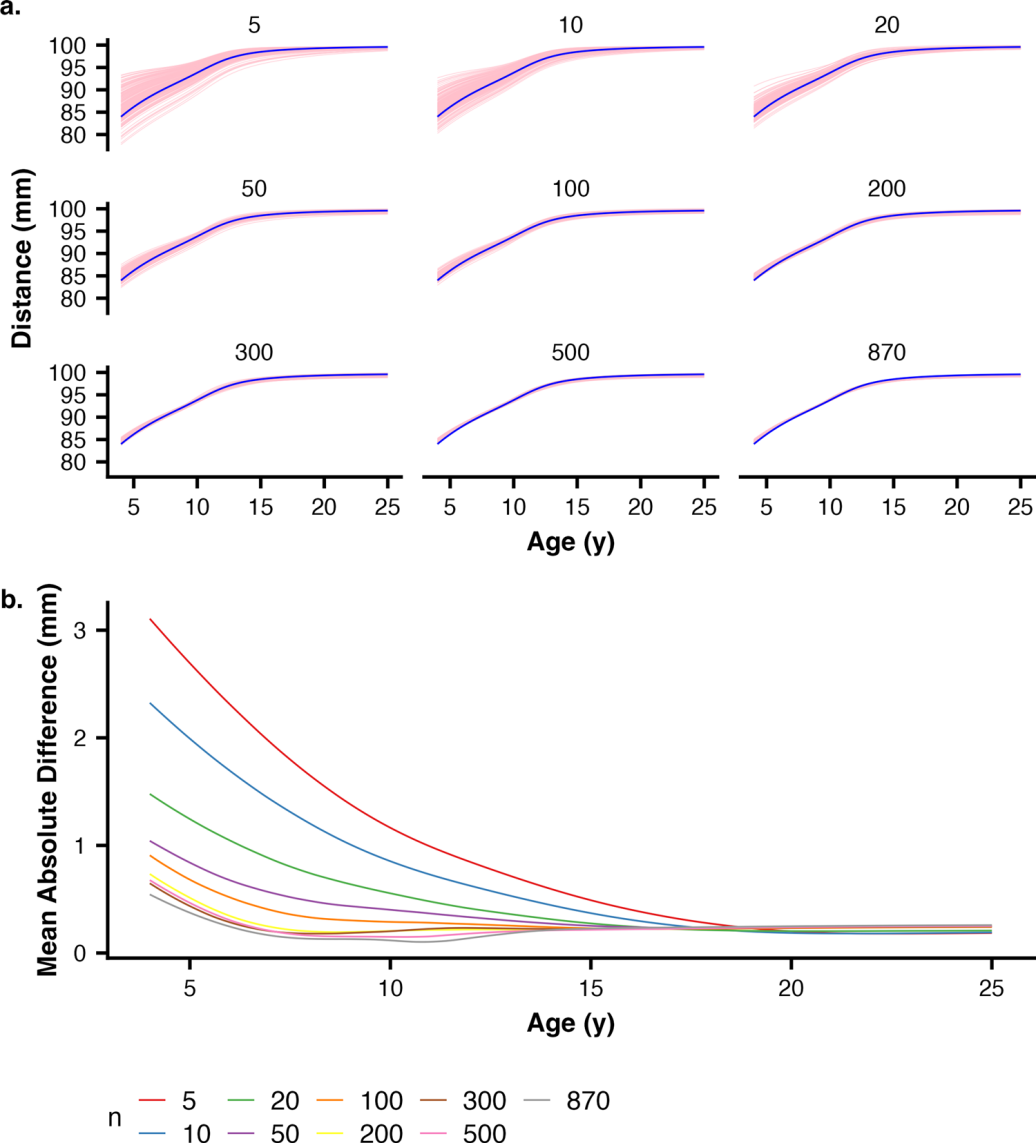
(**a**) Effect of sample size on nasion to basion distance prediction. The blue line represents the median predicted measurement from the longitudinal model. Each of the pink lines represents one of 200 random subsample models. As sample size increases, the effect of single extreme points has gradually less effect. (**b**) Differences in predictive ability. Mean absolute difference between the 200 samples and the longitudinal model was calculated across the predictive range. Average difference was usually less than 1 mm except for sample sizes below ~ 50, even though individual differences vary considerably more as seen in (**a**).

To assess the role of sample size in the ability of a cross-sectional sample to approximate a hierarchical longitudinal analysis, we analyzed subsamples ranging from the full sample size (n = 959 females or 980 males) down to n = 5 observations (i.e., a model fit to only 5 data points). These models were fit 200 times each using different random samples and posterior predictive mean measurements were calculated (Fig. 3a). Finally, to quantify the difference between the predictive ability across sample size, we calculated the mean absolute difference between each of the cross-sectional sample’s predicted sizes and predicted measures from the longitudinal model (Fig. 3b).

## Results

Overall results were similar across all traits and in both sexes. Thus, for simplicity we present only those results for one trait: the distance from Nasion to Basion in the female CGCS participants (Figs. 1 and 2). Full analysis of all traits in both sexes are included in the supplementary information (https://figshare.com/s/a0d90329654c2a3d8fcf).

### Posterior distributions

We focus here on the comparison of the full sample cross-sectional models to the longitudinal model. Comparisons of posterior distributions of parameter estimates (Fig. 1b) are similar between the longitudinal model and the median of the 200 cross-sectional models where one sample was drawn from each individual ("Full sample"). Most parameter estimates show almost complete overlap between the two approaches. When the posteriors differ (e.g., *f* and *c*_1_), the difference is small on the parameter scale: 0.5 mm or 0.5 years.

### Posterior predictions and growth rates

Median posterior predictive size was nearly identical between the longitudinal and cross-sectional models (Fig. 2a). Similarly, the 98% prediction intervals (the age-specific range in which 98% of new observations are expected to be located) agree very well, and only small deviations are observable at the earliest ages. Furthermore, both 98% intervals very well encompass the observed data, indicating that the double-logistic model used here is able to model the growth of craniofacial traits. Median predicted growth rates and age at peak growth velocity estimated from the growth curves differ by less than 0.1 mm on average (RMSD = 0.064 mm/y; Fig. 2b).

### Effects of sample size

As predicted, models estimated from small sample sizes show greater variation in predicted growth pattern. At young ages, models with 5–20 observations show greater variation than models fit to larger datasets (Fig. 3a), with some deviating by 10 mm or more (i.e., > 10% error). At sample sizes of 200 or more, all of the random-sample cross-sectional models closely approximate the longitudinal model. For the full sample model, the two are largely indistinguishable (Fig. 3a, lower right panel). Mean absolute difference between the predicted measure from the cross-sectional models and the predicted measure from the longitudinal model follows a similar pattern: as sample size increases, deviations decrease (Fig. 3b). Across the age range, all sample sizes of 100 or more show mean absolute deviations of 1 mm or less. We compared the growth milestones peak growth velocity (PGV) and age at peak growth velocity (aPGV) to those estimated from the longitudinal model across the different cross-sectional sample size. We found that PGV was <  ~ 0.5 mm/y and aPGV <  ~ 0.5 y, on average, with larger samples showing lower error in general (Supplemental information).

## Discussion

Longitudinal approaches are recognized as the gold standard for growth modeling^49,50^. As discussed above, however, longitudinal studies incur significant costs in terms of resources and participant commitment. When feasible to carry out, the advantage to this approach comes with the characterization of ontogenetic trajectories at the individual level in addition to the population level. It is clear that individuals, even though they might be similarly sized at the beginning and end of a growth spurt, may experience unique patterns of rate changes and thus differences in the timing of milestone achievements because the pacing of growth differs among individuals. We operate under the paradigm that longitudinal approaches provide a unique opportunity for an increased understanding of the variation in trajectories and of the influences on that variation. That knowledge can then be applied to analysis conducted on cross-sectional datasets to improve those results.

### Population modeling

The common goal of growth modeling using both longitudinal and cross-sectional data is to use observed data for individuals to estimate the patterns of trait change over time and the timings and magnitudes of growth milestones. In longitudinal samples such as the CGCS, experimenters can observe variation between different individuals in growth trajectories as well as within individual variation attributable to the combined sources of measurement error. In cross-sectional data, both of these sources of variation—–between individuals and measurement—are present, but they are not directly estimable. Thus, an implicit assumption of cross-sectional data is that the observed trait value falls at the true measurement for that individual at that age. Without any sense for measurement error, the observed value must be taken as accurate. That this assumption cannot be true is made clear by observing longitudinal data. Mean trait values increase monotonically, but individual observations do not, a pattern which is exhibited in Fig. 2a: measurements for some individuals appear to increase in size and then decrease, before increasing again. Thus, with longitudinal data, a single individual’s trait measurements will increase monotonically on average but will appear to locally increase and decrease. We do not believe that individuals are growing and shrinking in successive measurements but rather that we are observing a "noisy" pattern around a changing trait value representing growth. Nonetheless, without any additional information or a model which explicitly includes measurement error, single observations for any single individual must be assumed to be the true measure. In other words, with cross-sectional data, the observed values are assumed to accurately represent the real measurement.

If interest lies only in the population mean growth pattern or in percentile intervals for observations at a specific age, then the ratio of phenotypic variation to measurement error is of less concern. Indeed, Cock^51^ argued that cross-sectional data are useful in the study of growth but only for population patterns. Our results underscore the value of cross-sectional data in elucidating population patterns, and we add that via (1) biologically grounded modeling, (2) priors that mildly constrain parameter estimates, and (3) Bayesian sampling, population growth patterns can be used to inform individual-level prediction. Improved individual prediction is a long-term goal of this research program. With only a single data point, individual trajectories will closely resemble a shifted population curve. However, with two or more observations (e.g., pre- and post-treatment), particularly if these fall at or around the timing of peak growth velocity, the population growth pattern can be used to inform and potentially constrain the range of possible trait values at maturity.

### Sources of variation in data influencing parameter estimates

Observed craniofacial growth data can vary as a result of phenotypic variation in a population or via sources of error, each of which can impact parameter estimates and subsequent analyses. In the context of the craniofacial traits studied here, measurement error is introduced from a range of possible sources, including those stemming from collection of the radiograph (e.g., enlargement factor), non-orthogonal positioning of the participant to the radiographic plate^13^, and the scanning, digitizing and landmarking of the radiograph^36^. Although steps are taken to mitigate each of these sources of error, some of the observed variation in craniofacial traits inevitably stems from these sources. This experimental error is added to naturally occurring phenotypic variation. Despite these drawbacks, we have shown that the overall measurement error of a very large cross-sectional sample approximates the total variation of a longitudinal sample when all individuals are included in the sample, as indicated by the similarity in ranges of percentile intervals and overlap of the observed data with the 98% posterior interval (Fig. 2a). Although the CGCS is ancestrally homogeneous and we focus here on craniofacial traits, we believe that our results are broadly applicable to other groups and other traits. Our overall results are to be expected, because phenotypic variation and measurement error are indistinguishable from one another in real data measured on individuals such as the CGCS. In contrast, simulated data traditionally model only phenotypic variation either explicitly or implicitly.

Both phenotypic variation and measurement error contribute to observed (i.e., "apparent") variation, but in the absence of additional information, such as repeated sampling of the same individual at the same age, the relative magnitudes of each are unknown. Longitudinal modeling offers one approach to estimate measurement error for a trait. For a monotonically increasing trait value, the observed measurements could be assumed to oscillate around the true trait measurement, with deviations drawn from a normal distribution with a mean of zero. Indeed, the multilevel longitudinal model with random intercepts for each participant used in this study fits exactly this model. Using the multilevel longitudinal model, we estimated the median measurement error across all traits in both sexes at 0.87 mm (~ 1.2%). However, the percent error was lower for larger traits (r =  − 0.64, *P* < 0.001) where more of the observed variation can be attributed to inter-individual variation.

When considering phenotypes with large absolute magnitude relative to their associated measurement error, such as stature, observed variation can be reliably assigned as phenotypic variation rather than due to measurement error^23,46–48,52,53^. In stature (or recumbent length in the very young), substantial increase is seen during growth. For example, an infant that is 80 cm at age 1 may increase to 180 cm at age 18. If the measurement error remains constant across the age range at ~ 0.2 cm, then the percent error decreases from 0.25% at age 1 year to 0.11% at age 18. Comparing this to the craniofacial traits examined here, largest craniofacial trait studied here (Nasion to Menton distance in males) is six times smaller than the stature of even a one-year-old child. The consequence is that models of large magnitude traits with relatively low measurement error (e.g., stature) will be better able to estimate population-level variation, whereas relatively small traits where measurement error can be relatively greater (e.g., nasion-menton) are more challenging to model longitudinally. Further, cross-sectional modeling across age may be more sensitive to such error, whereas longitudinal measurements on individual children can have internal checks on serial measures. Care must be taken, therefore, in craniofacial growth modeling, particularly in cross-sectional population modeling. Comparing PGV and aPGV milestones estimated from cross-sectional samples to the complete longitudinal data (Supplemental Information Fig. 1) for the smallest trait (ANS-PNS), a mid-sized trait (Sella-Gonion), and the largest trait (Nasion-Menton), we find no clear pattern in aPGV. It is slightly overestimated by about 6 months for ANS-PNS and N-M, but very accurately estimated for S-Go. Similarly, PGV is very accurate for ANS-PNS and N-M but slightly underestimated for S-Go by ~ 0.4 mm/y. Given that the CGCS represents the combined sample from separate growth studies spanning nearly 100 years, each of which used different imaging protocols and technologies, we find this level of accuracy to be acceptable. In the supplemental information, we provide these comparisons for all traits in both sexes.

### How large of a cross-sectional sample is adequate?

We found that cross-sectional modeling using increasingly larger subsets of the entire CGCS sample of participants approximates longitudinal models very well for craniofacial traits. Both percentile size intervals and rates of growth from cross-sectional subsamples agree with longitudinal models fit to the full dataset (Fig. 2). Although any single model fit to a relatively small dataset may deviate substantially from the population pattern, an aggregated summary of a large number of models is a good approximation, even if those subsample sizes are relatively small (Fig. 3). It is important to note that the random samples in this study are themselves drawn from a very large sample (> 7000 observations per trait for each sex). A single random set of even many hundred individuals might not adequately represent the population-level patterns, but repeated resampling of those data will provide an improved estimate of the overall growth pattern compared to a single model estimate (Fig. 3a). In these data, we found that a sample of at least 200 individuals used with resampling approaches to best estimate population growth patterns from cross-sectional data.

In conclusion, we found that cross-sectional data can provide robust estimates of not only growth in craniofacial traits over time, but also milestones derived from those growth curves, including peak growth velocity and age at peak growth velocity. Importantly, these results are similar to those from a large, densely sampled longitudinal data dataset, provided that the cross-sectional data are sampled repeatedly and at sufficiently large size. Our results provide a method by which to fully utilize cross-sectional data in situations when longitudinal samples are not possible, for example due to time, cost, or health concerns. We envision application of this cross-sectional resampling methodology to the analysis of 3D craniofacial imaging via cone-beam computed-tomography (CBCT). Although CBCT involves a relatively low dose of radiation, serial imaging for research purposes is neither warranted nor feasible. The methods described here will prove invaluable to the study of not only craniofacial growth, but other forms of growth.

### Supplementary Information

Supplementary Information.

## Data Availability

Supporting information for this manuscript is available via Figshare (https://figshare.com/s/a0d90329654c2a3d8fcf), which includes R and stan code to reproduce all analysÞes and figures. Due to the raw data containing personal health information (PHI), we are not able to share the raw data. However, we do share the posterior samples for both longitudinal and cross-sectional analyses, which allow users to carry out all subsequent analyses. Requests for data should be directed to Richard J. Sherwood (sherwoodrj@health.missouri.edu; Department of Pathology and Anatomical Sciences, University of Missouri School of Medicine, Columbia, Missouri, USA).
