# Supplementary material for: Cross-sectional data accurately model longitudinal growth in the craniofacial skeleton
Source: Sci Rep. 2023 Nov 7;13:19294. doi: 10.1038/s41598-023-46018-x (PMC10630296; doi:10.1038/s41598-023-46018-x)
Supplement: Supplementary file 1 — Supplementary Information. [file 41598_2023_46018_MOESM1_ESM.pdf]

Modeling craniofacial growth: Can cross-sectional data approximate true longitudinal growth?

Supplemental Information

Contents

1 Summary for all traits **4**

1.1 Females 4

1.2 Males 4

2 Comparison of milestones for ANS-PNS, Sella-Gonion, and Nasion-Menton **5**

3 Female, ANS-PNS **7**

4 Female, Articulare-Pogonion **14**

5 Female, Condylion-Gonion **21**

6 Female, Condylion-Pogonion **28**

7 Female, Gonion-Pogonion **35**

8 Female, Menton-ANS **42**

9 Female, Nasion-ANS **49**

10 Female, Nasion-Basion **56**

11 Female, Nasion-Menton **63**

12 Female, Sella-Basion **70**

13 Female, Sella-Gonion **77**

14 Female, Sella-Nasion **84**

15 Male, ANS-PNS **91**

16 Male, Articulare-Pogonion **98**

17 Male, Condylion-Gonion **105**

18 Male, Condylion-Pogonion **112**

19 Male, Gonion-Pogonion **119**

20 Male, Menton-ANS **126**

21 Male, Nasion-ANS **133**

22	Male, Nasion-Basion	140
23	Male, Nasion-Menton	147
24	Male, Sella-Basion	154
25	Male, Sella-Gonion	161
26	Male, Sella-Nasion	168

1 Summary for all traits

The tables below show the total number of observations, the median number of observations per participant, and the total number of participants with measurements for each trait.

1.1 Females

	Total Observations	Median Observations	Total Participants
Female ans pns	7525	9	871
Female articulare pogonion	7432	9	871
Female condylion gonion	7514	9	871
Female condylion pogonion	7452	9	871
Female gonion pogonion	7464	9	871
Female menton ans	7389	9	871
Female nasion ans	7519	9	871
Female nasion basion	7430	9	870
Female nasion menton	7384	9	871
Female sella basion	7438	9	870
Female sella gonion	7527	9	871
Female sella nasion	7520	9	871

1.2 Males

	Total Observations	Median Observations	Total Participants
Male ans pns	7664	9	880
Male articulare pogonion	7549	9	881
Male condylion gonion	7654	9	881
Male condylion pogonion	7580	9	881
Male gonion pogonion	7598	9	881
Male menton ans	7484	9	879
Male nasion ans	7654	9	880
Male nasion basion	7548	9	879
Male nasion menton	7486	9	880
Male sella basion	7559	9	879
Male sella gonion	7673	9	881
Male sella nasion	7667	9	881

2 Comparison of milestones for ANS-PNS, Sella-Gonion, and Nasion-Menton

Figure 1. The difference between estimated aPGV and PGV milestones for ANS-PNS, Sella-Gonion, and Nasion-Menton. We calculated the difference between the estimated aPGV and PGV for each cross-sectional sample and the full longitudinal data. The red point parks the median difference, and the bars are the 95% middle quantile calculated from all 200 iterations.

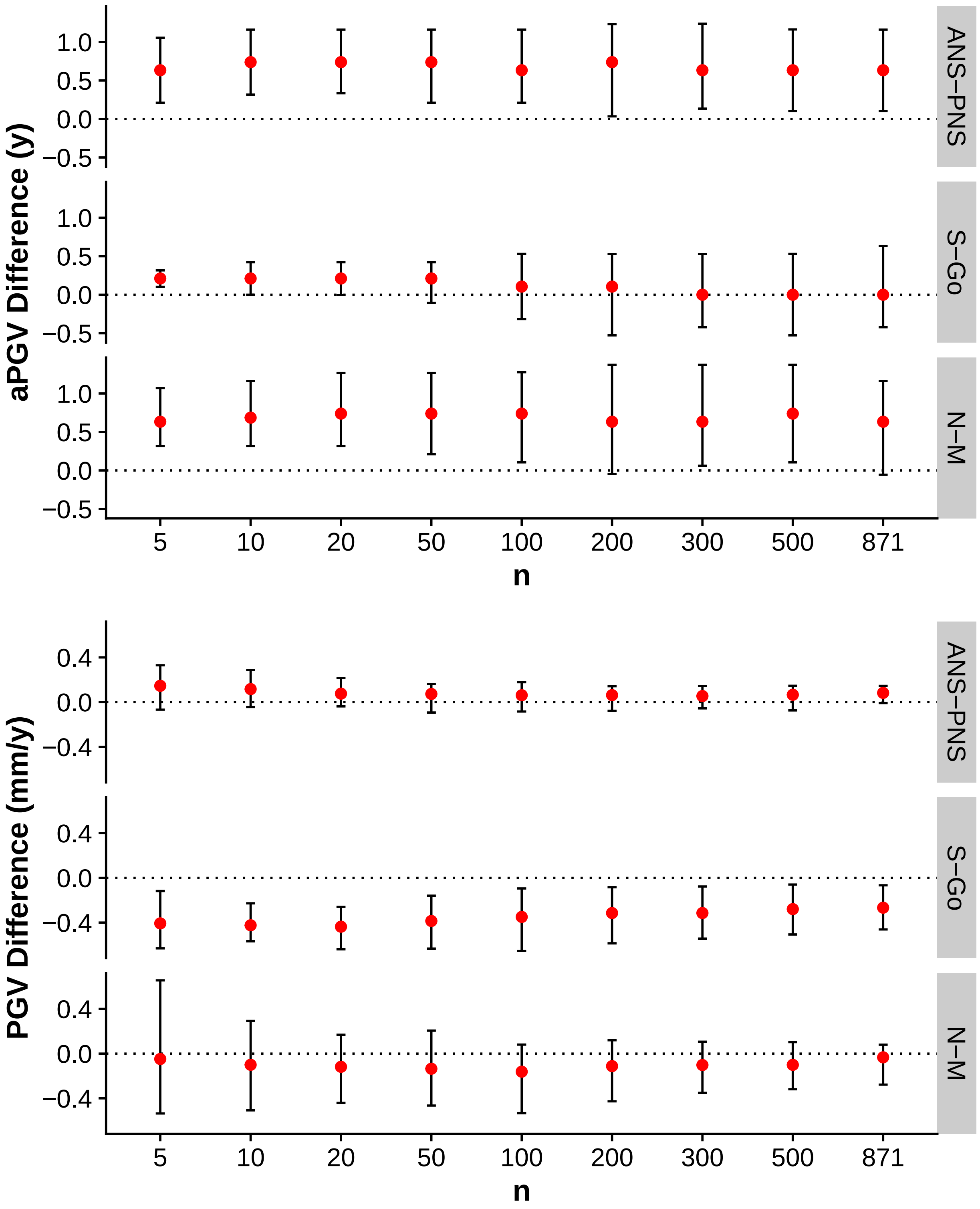

aPGV Difference (y)
1.0
0.5
0.0
−0.5
ANS−PNS
S−Go
N−M
5
10
20
50
100
200
300
500
871
n
PGV Difference (mm/y)
0.4
0.0
−0.4
ANS−PNS
S−Go
N−M
5
10
20
50
100
200
300
500
871
n

3 Female, ANS-PNS

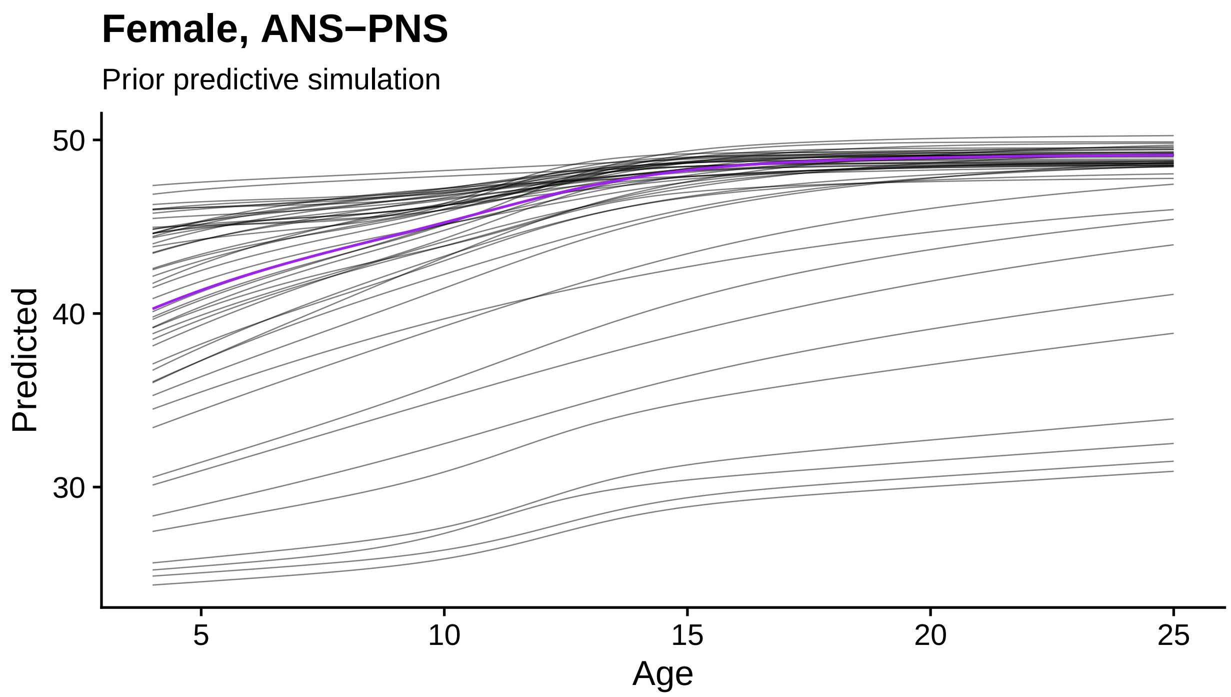

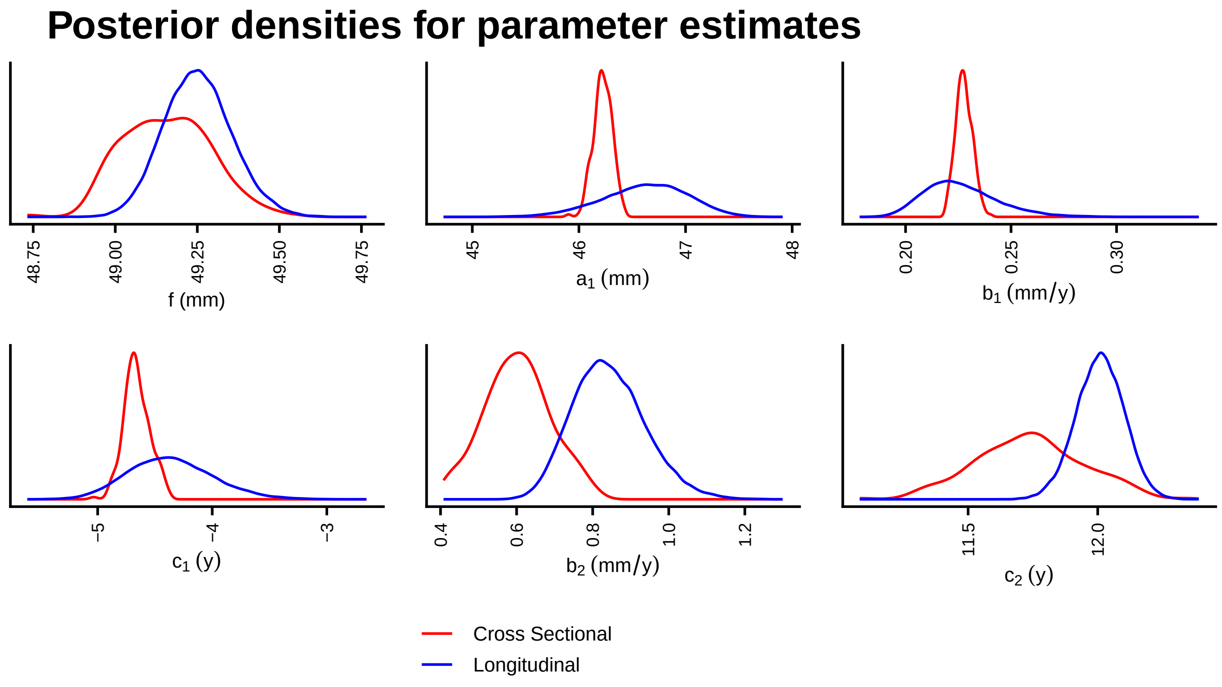

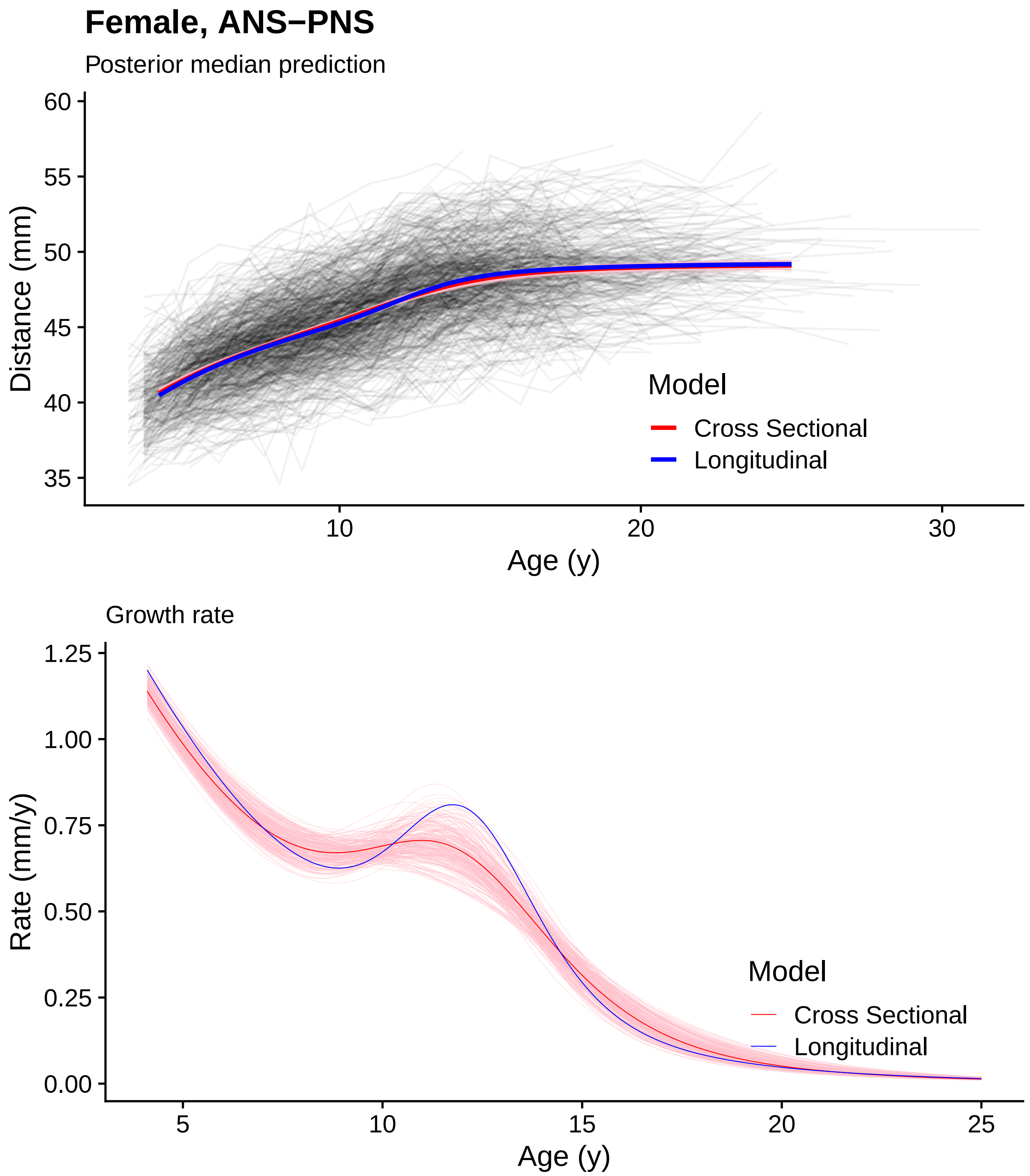
Female, ANS−PNS
Posterior median prediction
Distance (mm)
Age (y)
Model
Cross Sectional
Longitudinal
Growth rate
Rate (mm/y)
Age (y)
Model
Cross Sectional
Longitudinal

Table 3: Longitudinal Model Summary

Parameter	Mean	Median	Std. Dev.	MAD	5%	95%	$\hat{r}$	Bulk ESS	Tail ESS
f	49.25	49.25	0.104	0.103	49.09	49.43	1	5434	12856
a1	46.62	46.64	0.401	0.402	45.92	47.24	1	17250	20509
b1	0.23	0.22	0.017	0.017	0.20	0.26	1	16444	20074
c1	-4.36	-4.38	0.369	0.368	-4.93	-3.72	1	18607	22783
b2	0.84	0.84	0.096	0.095	0.70	1.01	1	21174	26046
c2	12.01	12.01	0.092	0.093	11.86	12.16	1	57315	30149
sigma	1.23	1.23	0.010	0.010	1.21	1.25	1	62575	30117
sigma_ID	2.13	2.12	0.051	0.051	2.04	2.21	1	75073	31152

Table 4: Median Coefficients

Model	f	a_1	b_1	c_1	b_2	c_2	σ	σ_{ID}
Longitudinal	49.25	46.64	0.22	-4.38	0.84	12.01	1.23	2.12
Cross Sectional	49.16	46.23	0.23	-4.67	0.60	11.74	2.43	NA

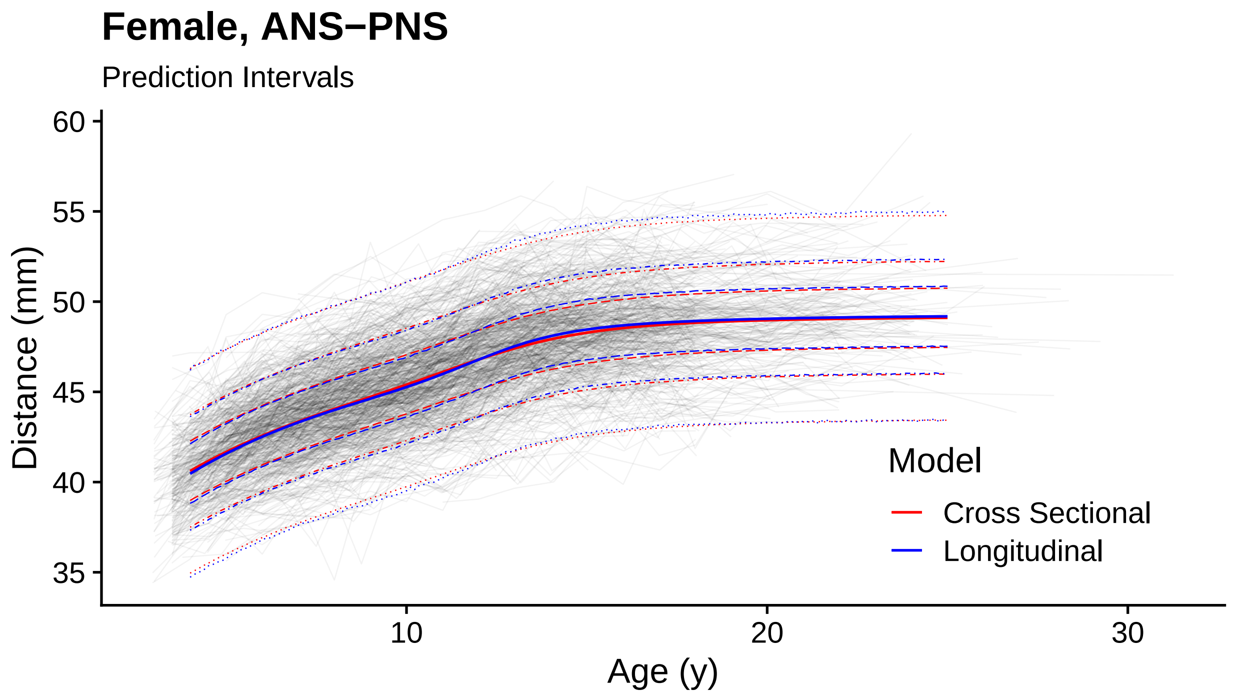
Female, ANS−PNS
Prediction Intervals
Distance (mm)
60
55
50
45
40
35
10
20
30
Age (y)
Model
Cross Sectional
Longitudinal

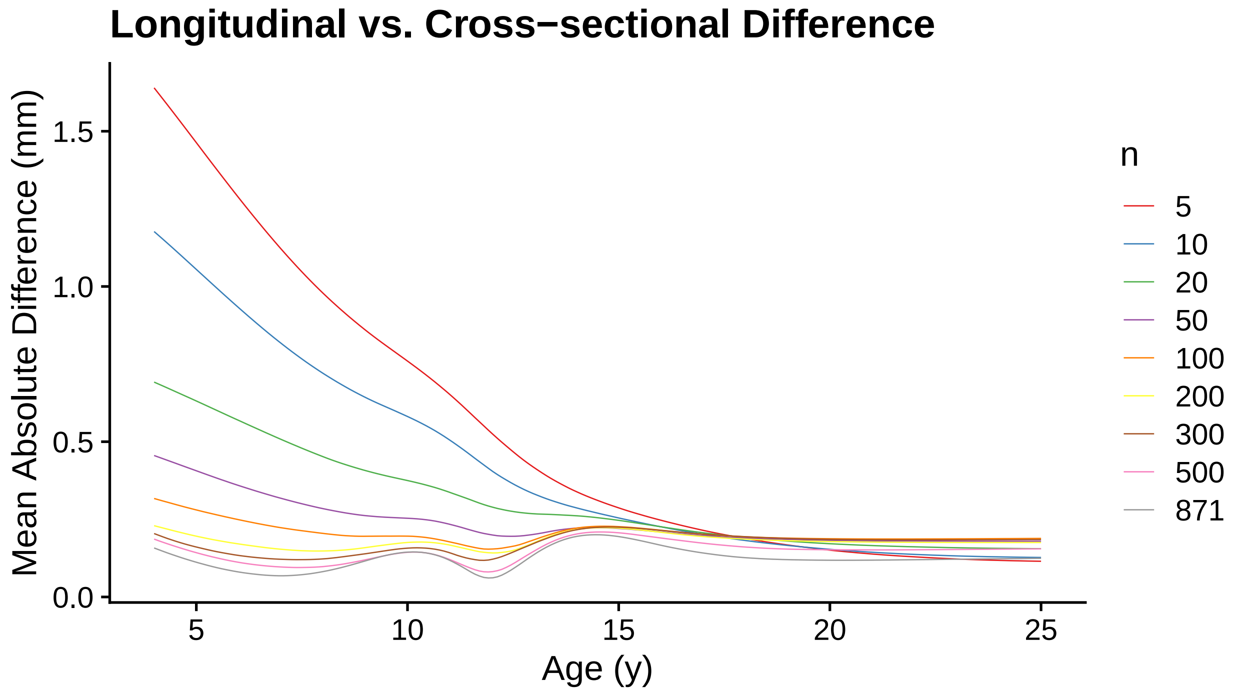
Longitudinal vs. Cross−sectional Difference
Mean Absolute Difference (mm)
1.5
1.0
0.5
0.0
5
10
15
20
25
Age (y)
n
5
10
20
50
100
200
300
500
871

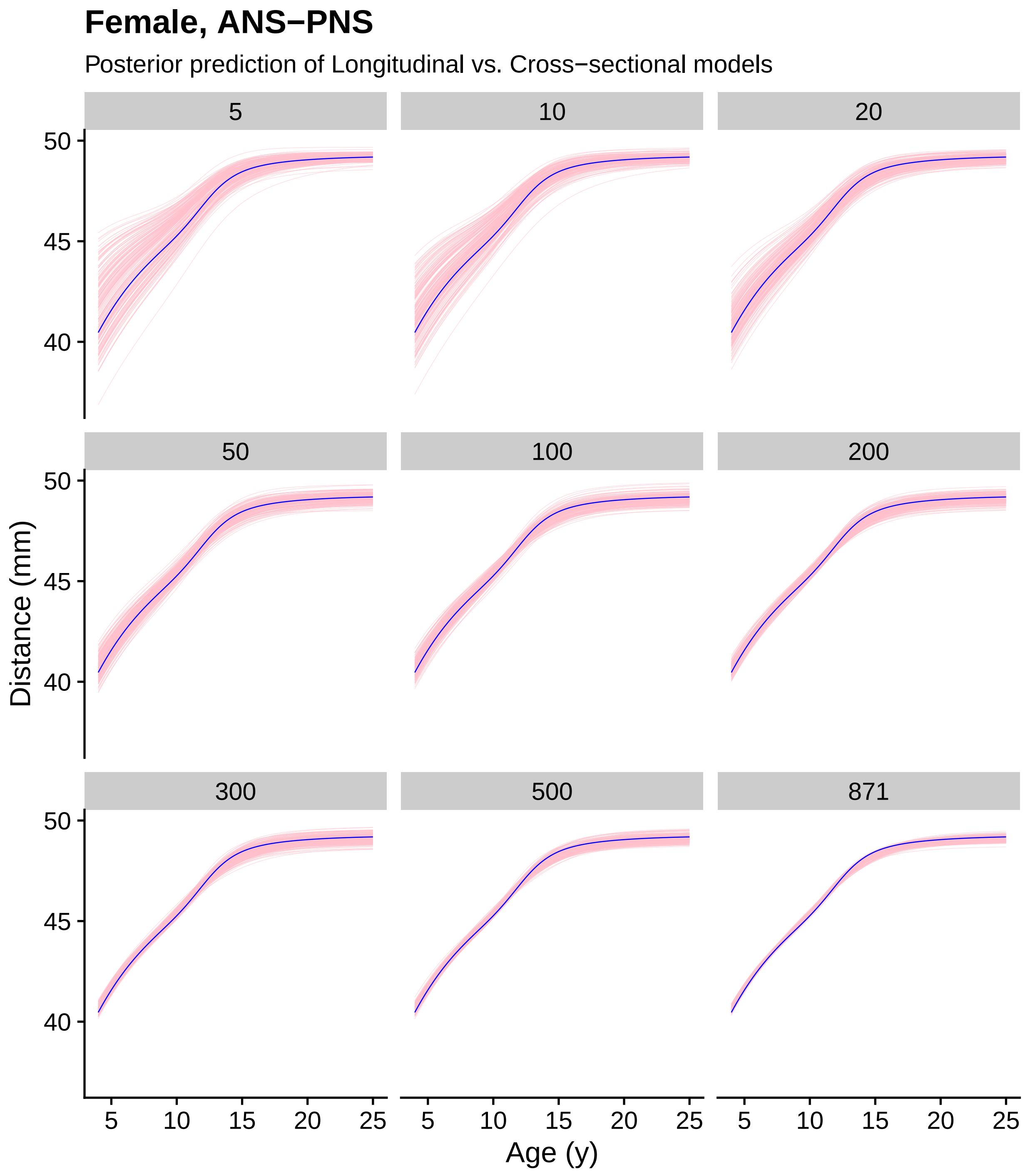

Female, ANS−PNS
Posterior prediction of Longitudinal vs. Cross−sectional models
5
10
20
50
100
200
300
500
871
Distance (mm)
Age (y)

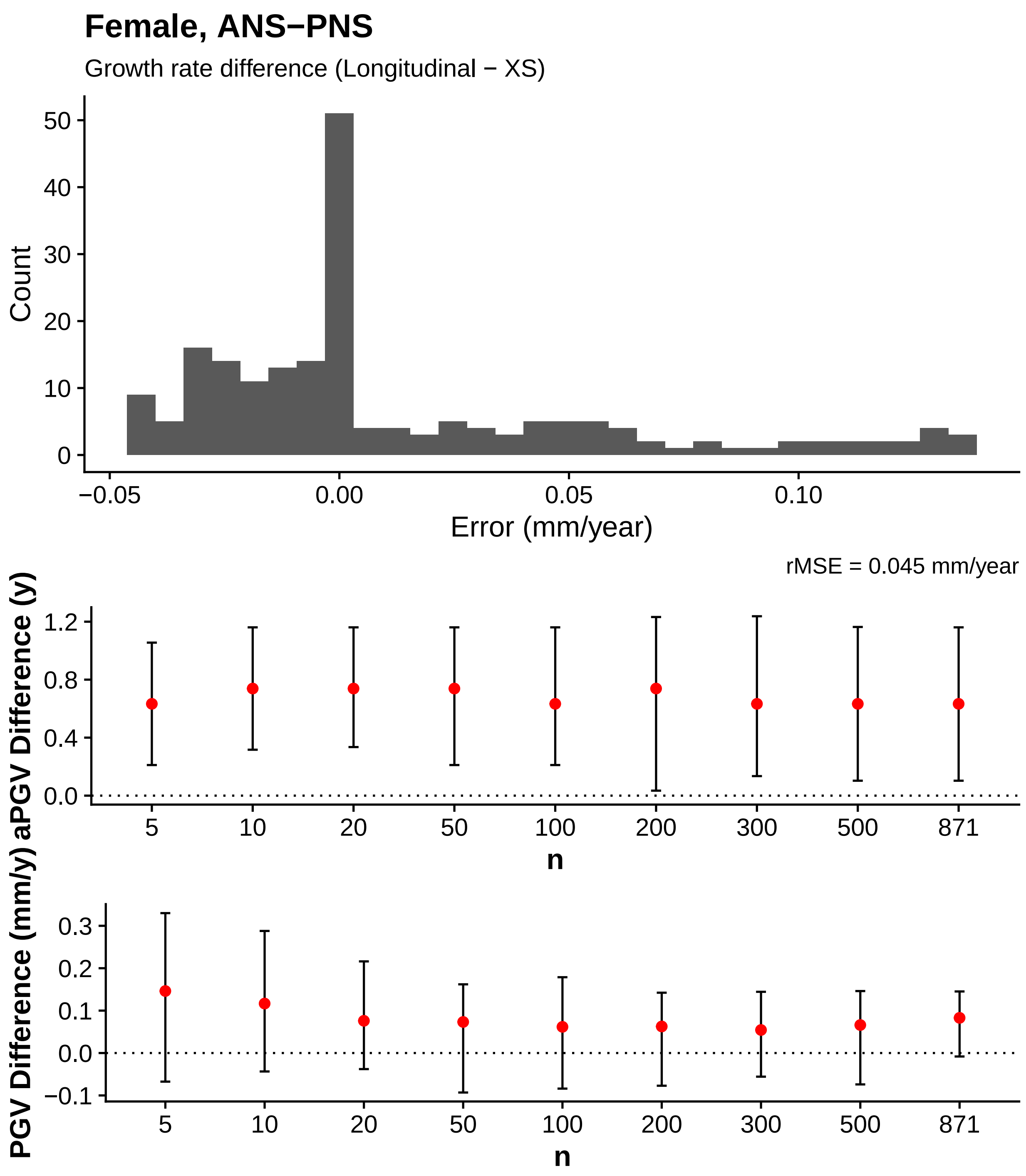
Female, ANS−PNS
Growth rate difference (Longitudinal − XS)
Count
Error (mm/year)
rMSE = 0.045 mm/year
aPGV Difference (y)
PGV Difference (mm/y)
n
5
10
20
50
100
200
300
500
871

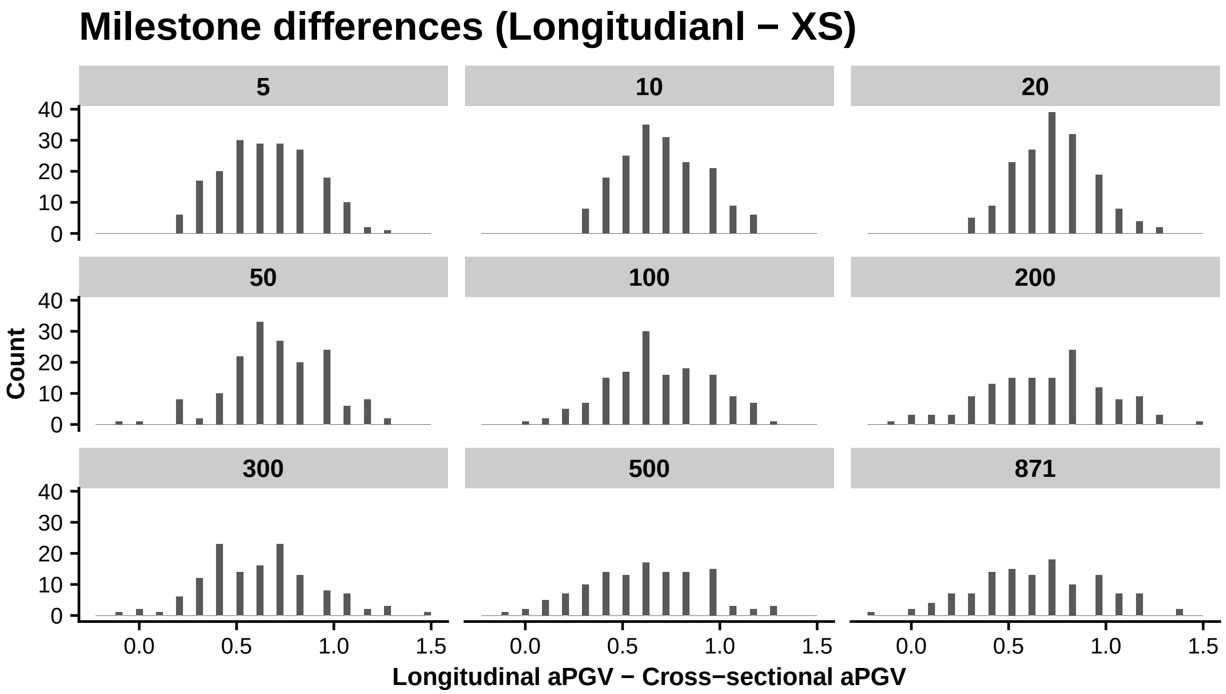
Milestone differences (Longitudianl − XS)
5
10
20
50
100
200
300
500
871
Count
Longitudinal aPGV − Cross−sectional aPGV

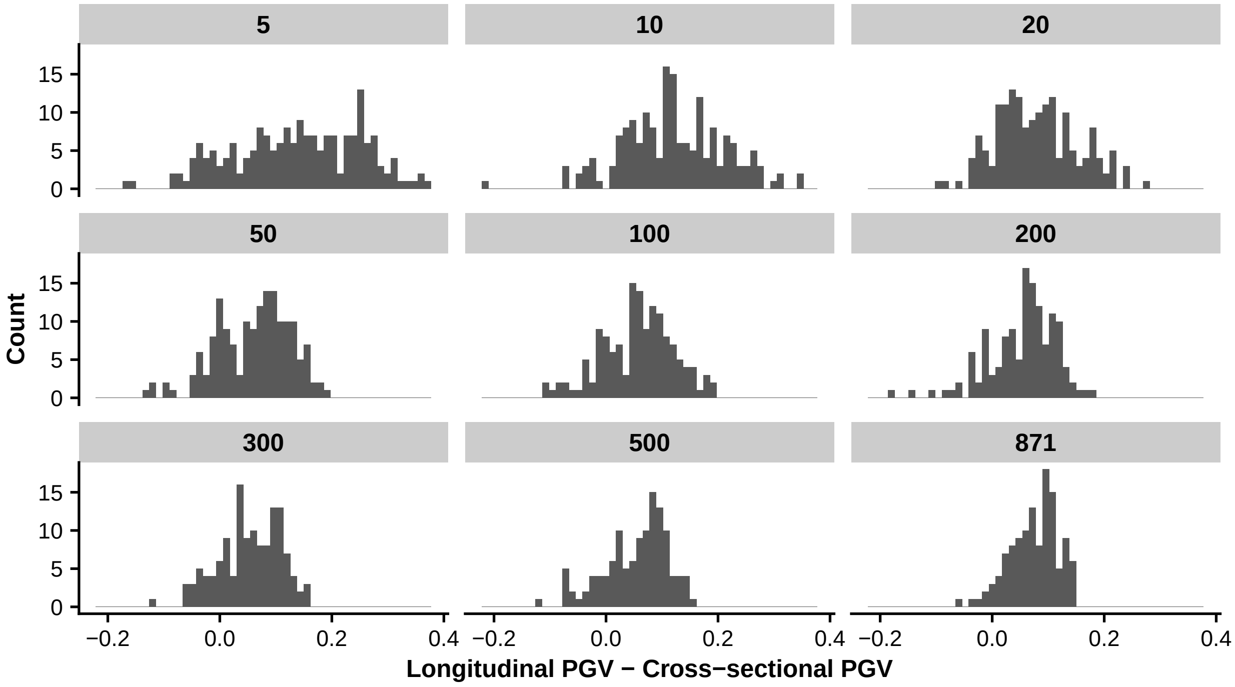
5
10
20
50
100
200
300
500
871
Count
Longitudinal PGV − Cross−sectional PGV

4 Female, Articulare-Pogonion

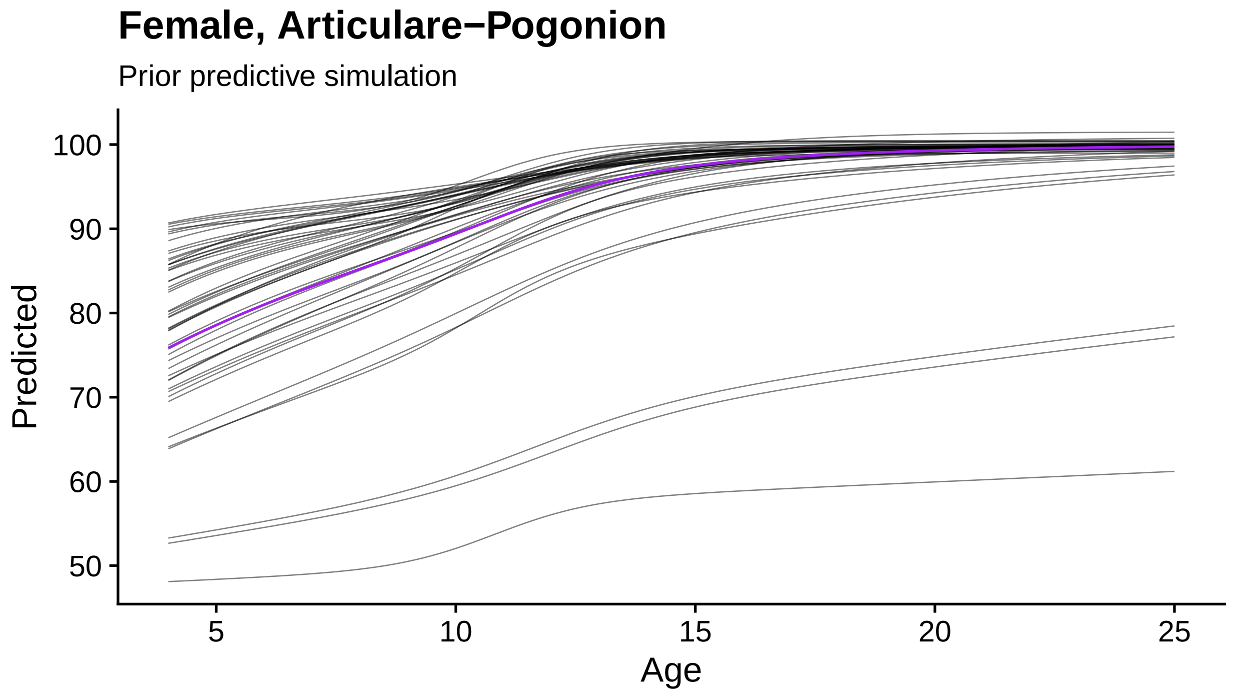

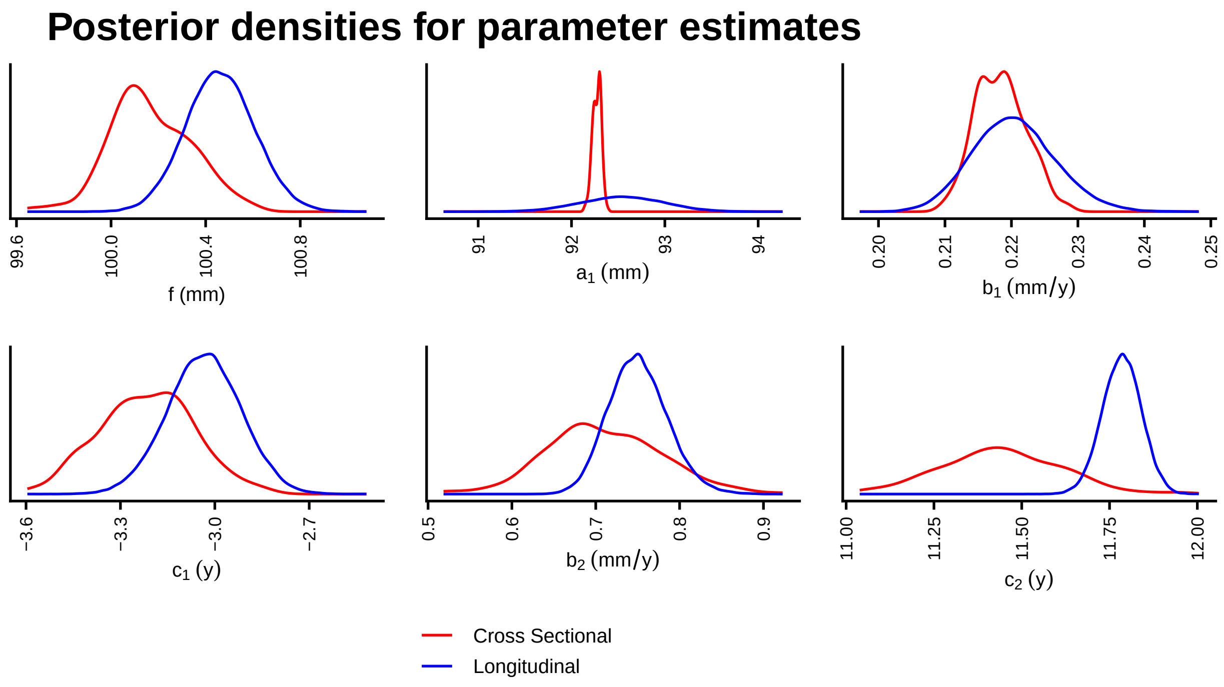

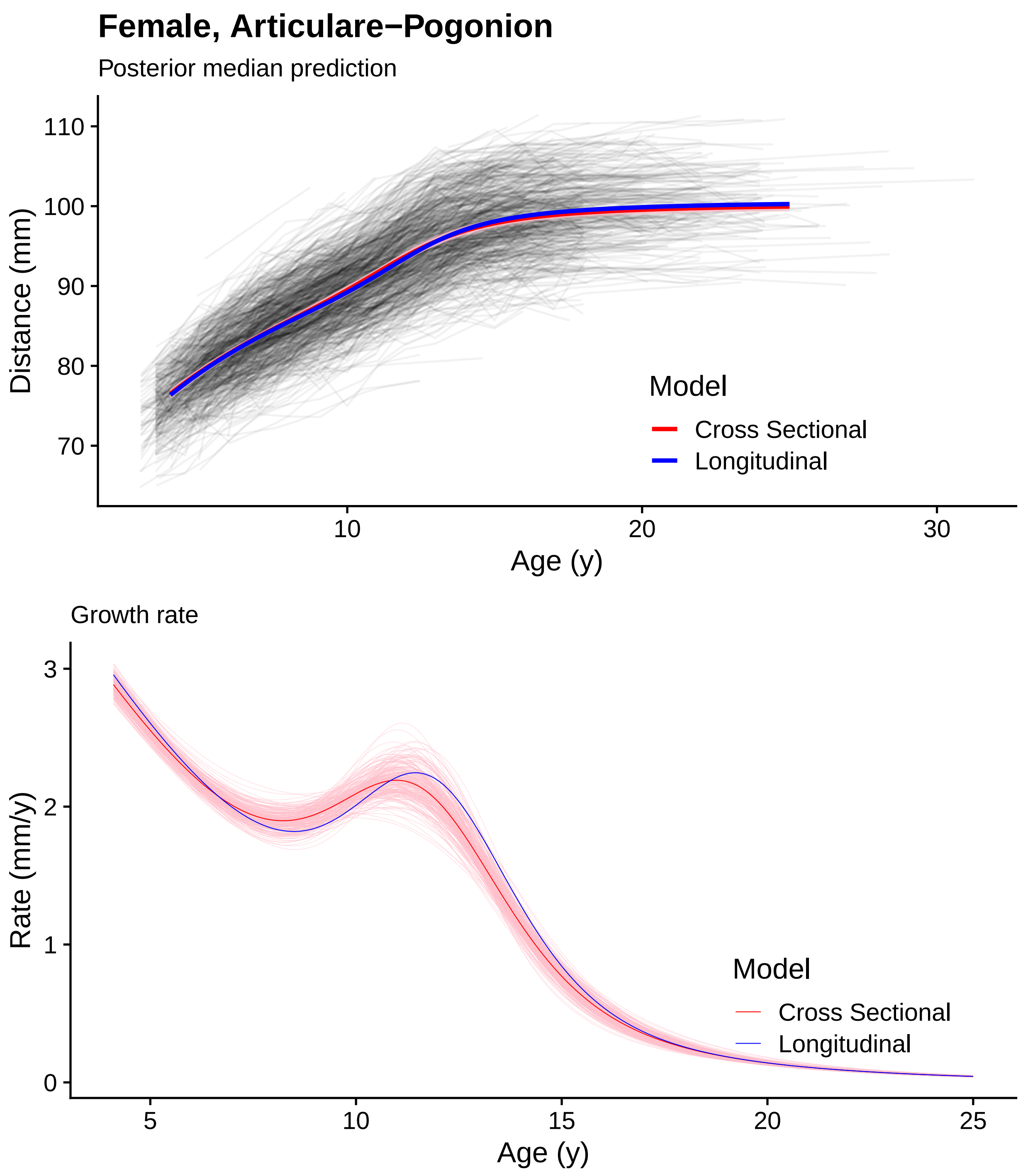
Female, Articulare−Pogonion
Posterior median prediction
Distance (mm)
110
100
90
80
70
10
20
30
Age (y)
Model
Cross Sectional
Longitudinal
Growth rate
Rate (mm/y)
3
2
1
0
5
10
15
20
25
Age (y)
Model
Cross Sectional
Longitudinal

Table 5: Longitudinal Model Summary

Parameter	Mean	Median	Std. Dev.	MAD	5%	95%	$\hat{r}$	Bulk ESS	Tail ESS
f	100.46	100.46	0.146	0.145	100.22	100.70	1	2915	8073
a1	92.54	92.54	0.444	0.446	91.81	93.26	1	31030	28361
b1	0.22	0.22	0.006	0.006	0.21	0.23	1	17744	26922
c1	-3.03	-3.03	0.117	0.117	-3.23	-2.84	1	38165	31685
b2	0.75	0.75	0.035	0.035	0.70	0.81	1	25214	31780
c2	11.78	11.79	0.055	0.056	11.69	11.87	1	50868	32219
sigma	1.81	1.81	0.015	0.015	1.79	1.84	1	69775	30222
sigma_ID	3.94	3.94	0.093	0.093	3.79	4.10	1	78874	30166

Table 6: Median Coefficients

Model	f	a_1	b_1	c_1	b_2	c_2	σ	σ_{ID}
Longitudinal	100.46	92.54	0.22	-3.03	0.75	11.79	1.81	3.94
Cross Sectional	100.15	92.28	0.22	-3.22	0.70	11.44	4.32	NA

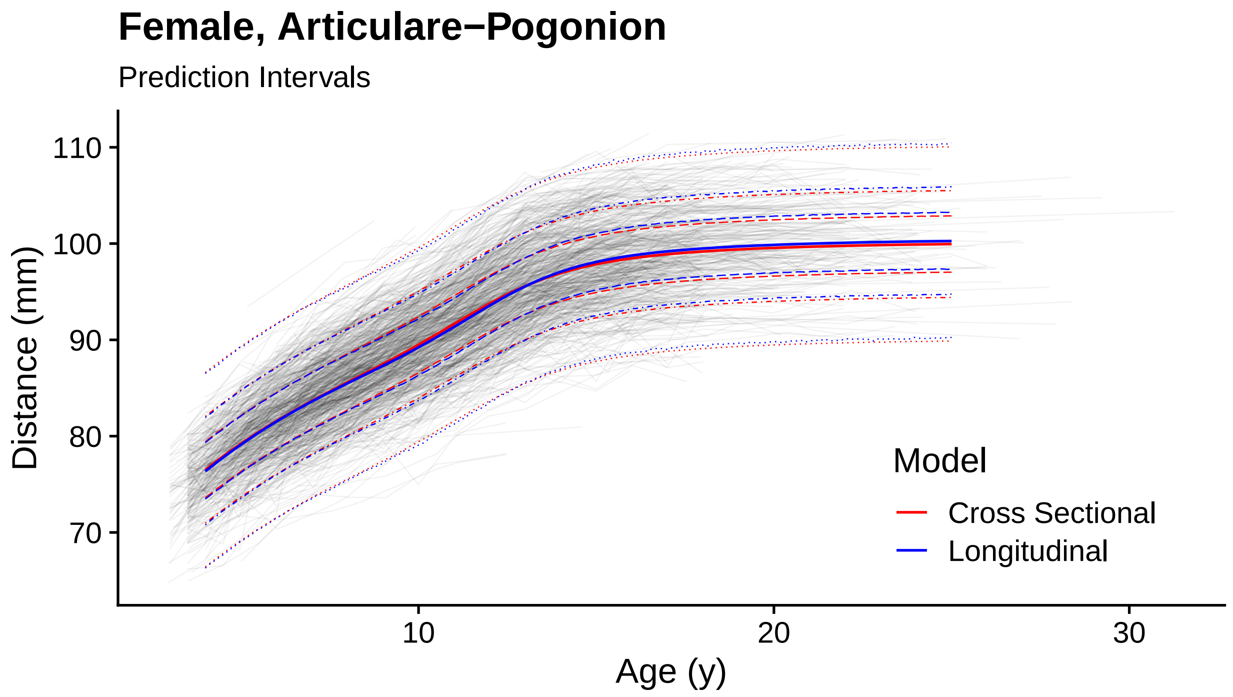
Female, Articulare−Pogonion
Prediction Intervals
Distance (mm)
110
100
90
80
70
10
20
30
Age (y)
Model
Cross Sectional
Longitudinal

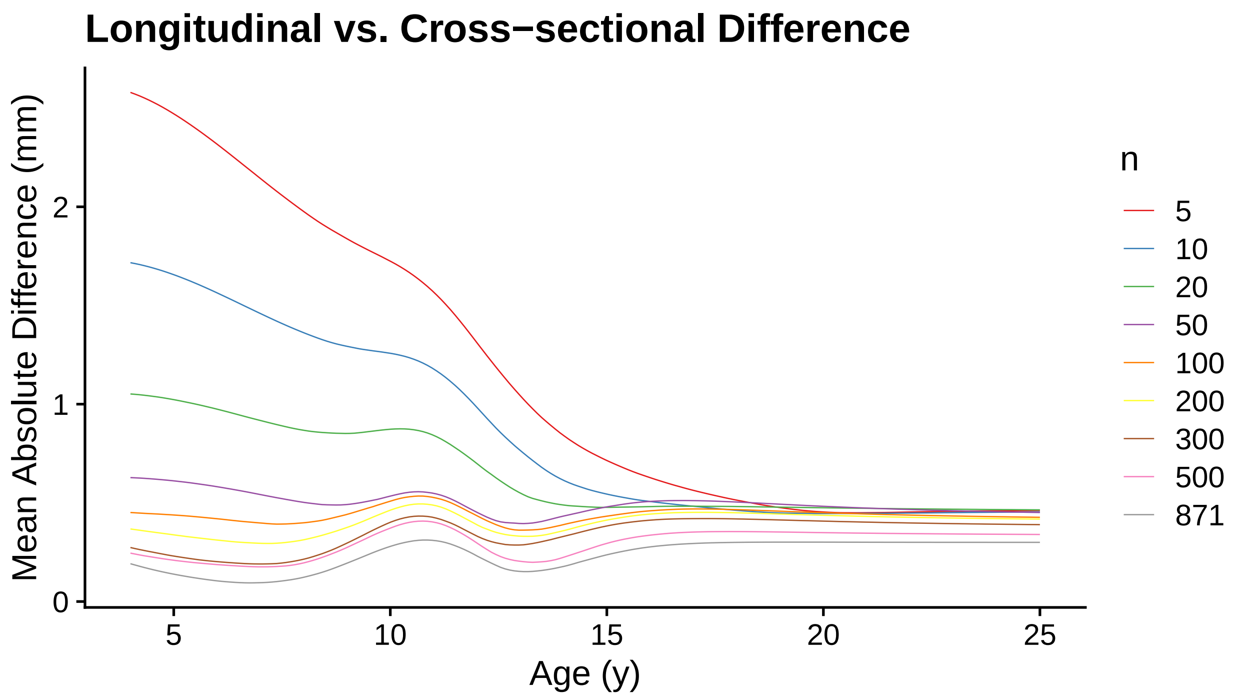
Longitudinal vs. Cross−sectional Difference
Mean Absolute Difference (mm)
2
1
0
5
10
15
20
25
Age (y)
n
5
10
20
50
100
200
300
500
871

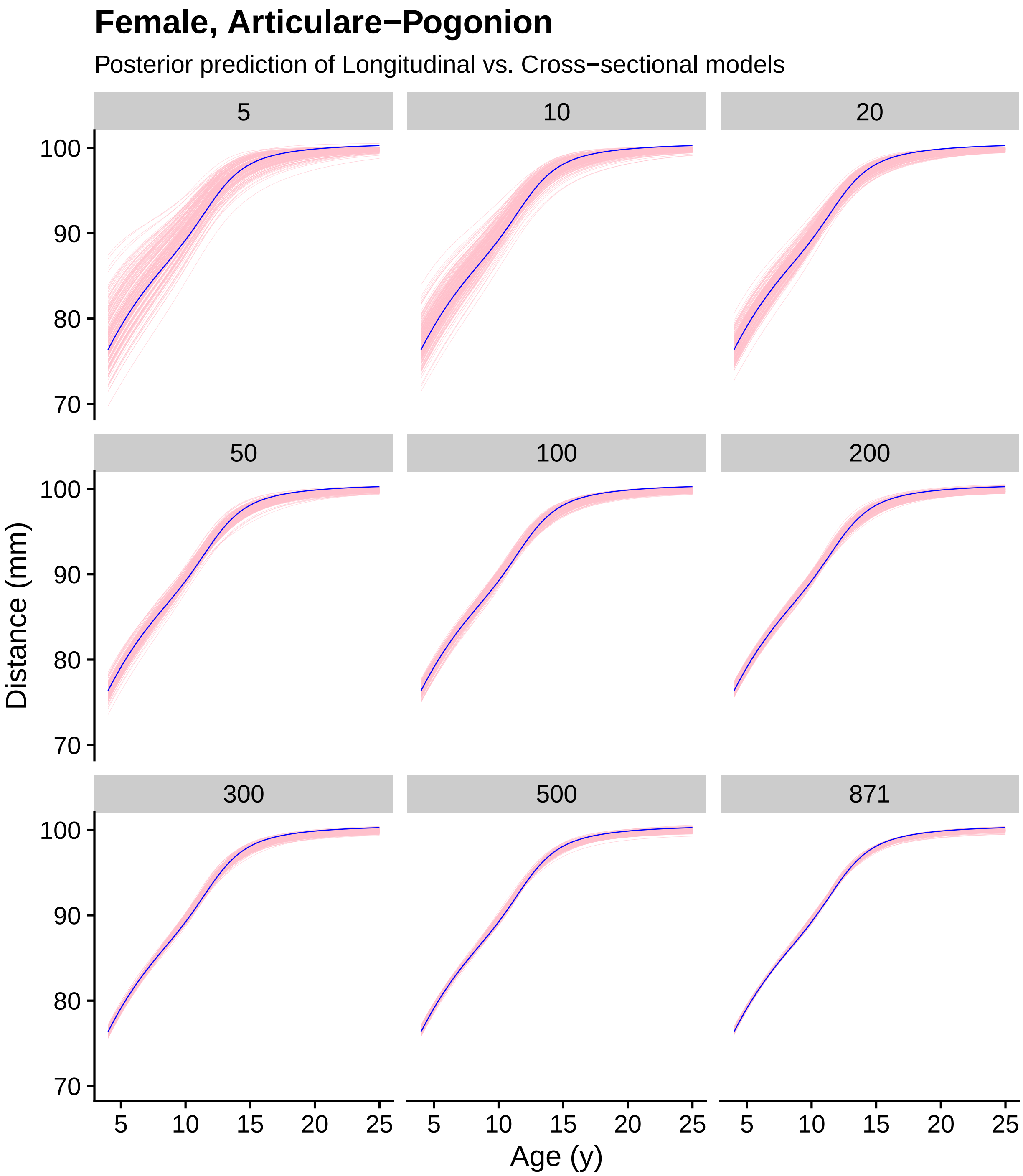

Female, Articulare−Pogonion
Posterior prediction of Longitudinal vs. Cross−sectional models
5
10
20
50
100
200
300
500
871
Distance (mm)
100
90
80
70
5
10
15
20
25
Age (y)

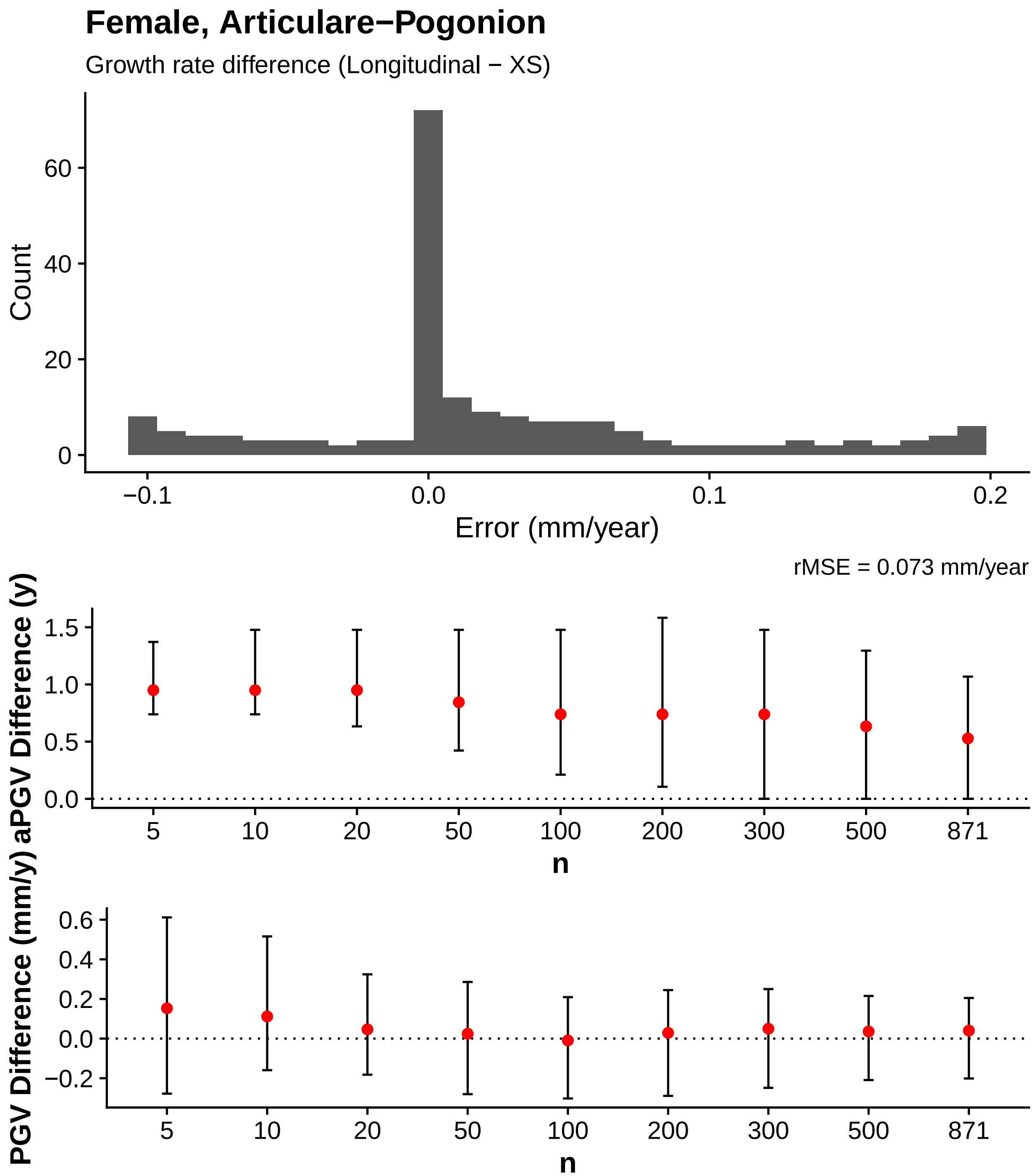
Female, Articulare−Pogonion
Growth rate difference (Longitudinal − XS)
Count
Error (mm/year)
rMSE = 0.073 mm/year
aPGV Difference (y)
n
PGV Difference (mm/y)
n

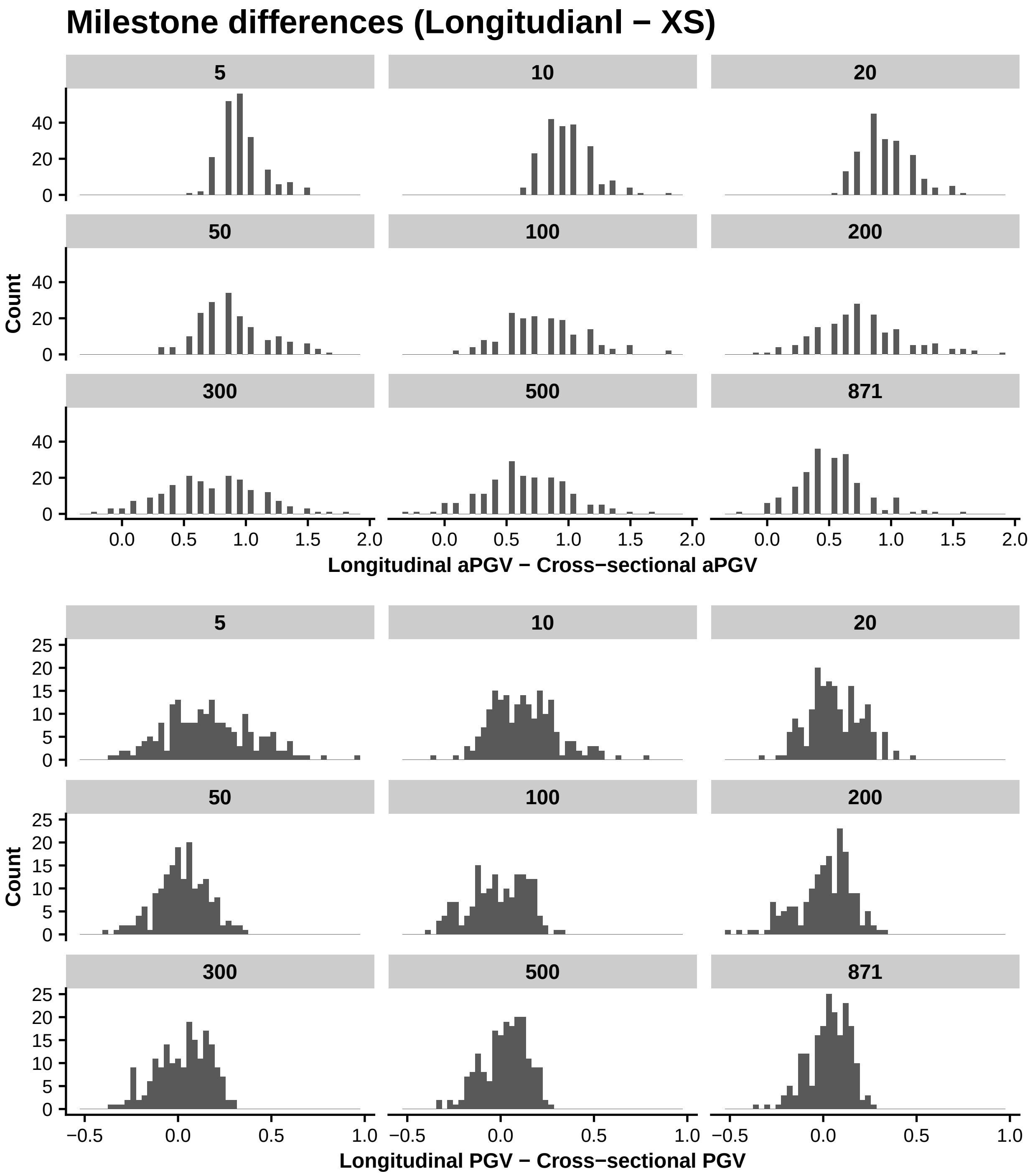
Milestone differences (Longitudianl − XS)
5
10
20
50
100
200
300
500
871
Count
Longitudinal aPGV − Cross−sectional aPGV
Longitudinal PGV − Cross−sectional PGV

5 Female, Condylion-Gonion

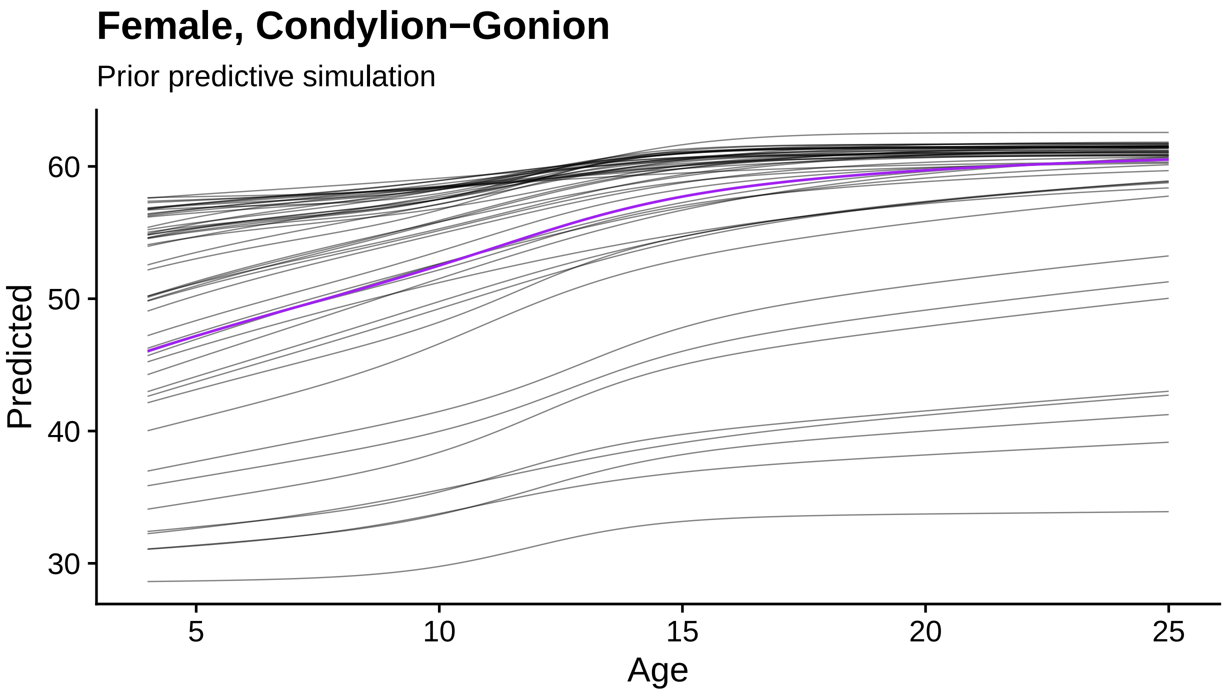

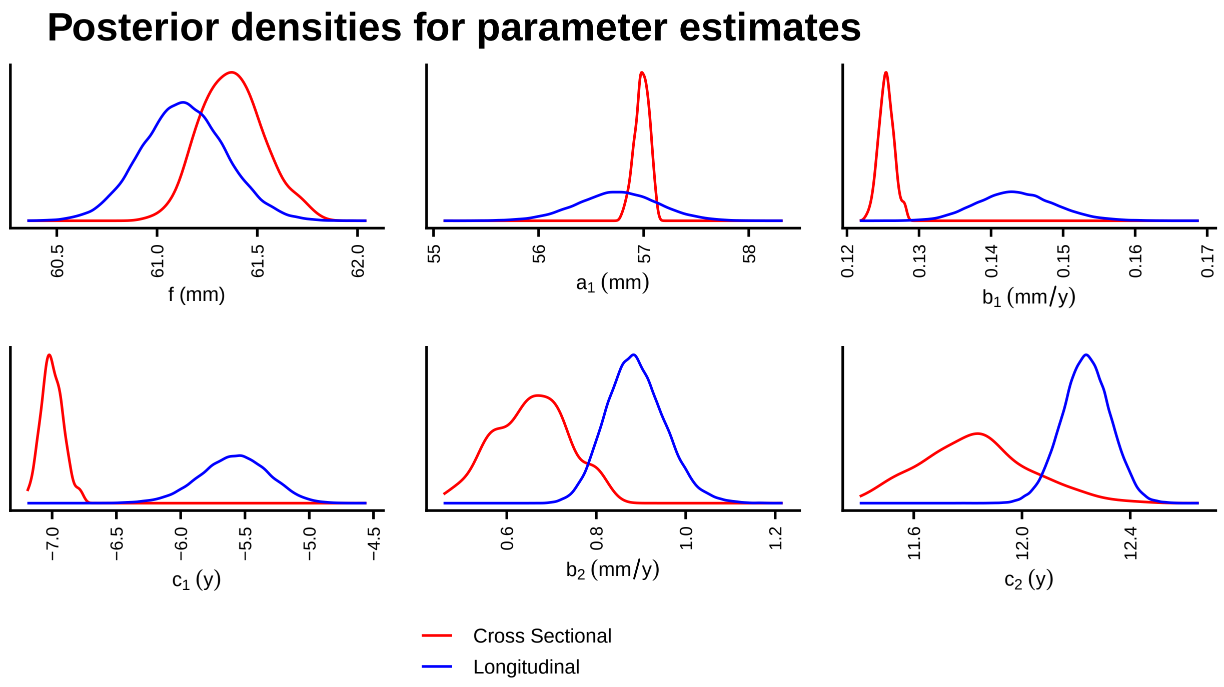

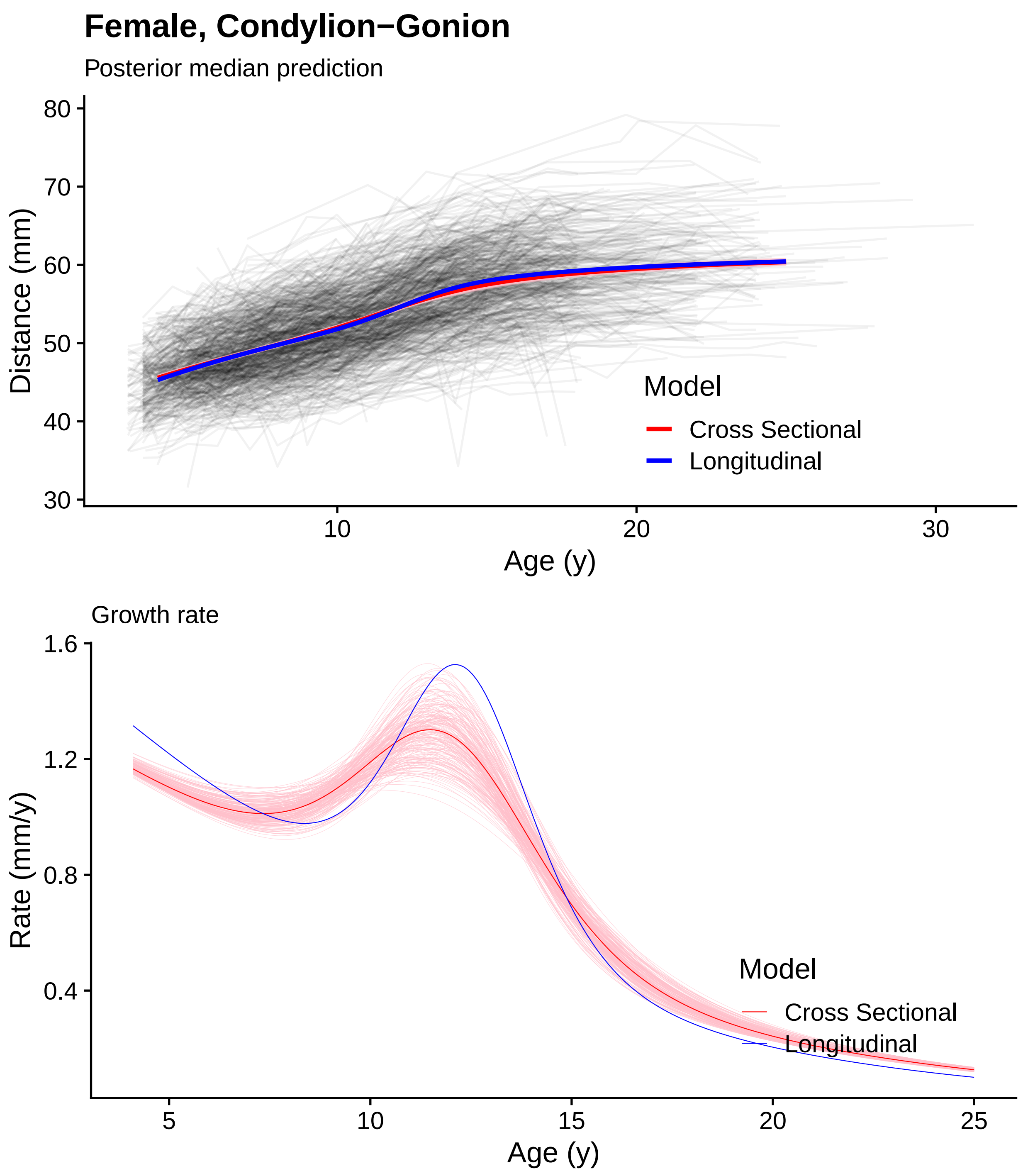

Female, Condylion−Gonion
Posterior median prediction
Distance (mm)
80
70
60
50
40
30
10
20
30
Age (y)
Model
Cross Sectional
Longitudinal
Growth rate
Rate (mm/y)
1.6
1.2
0.8
0.4
5
10
15
20
25
Age (y)
Model
Cross Sectional
Longitudinal

Table 7: Longitudinal Model Summary

Parameter	Mean	Median	Std. Dev.	MAD	5%	95%	$\hat{r}$	Bulk ESS	Tail ESS
f	61.13	61.13	0.209	0.208	60.79	61.48	1	10263	19584
a1	56.75	56.75	0.383	0.379	56.12	57.38	1	39839	32061
b1	0.14	0.14	0.005	0.005	0.14	0.15	1	19809	26480
c1	-5.59	-5.58	0.268	0.267	-6.04	-5.16	1	41077	31662
b2	0.89	0.89	0.066	0.065	0.79	1.00	1	42297	31739
c2	12.24	12.24	0.090	0.090	12.09	12.38	1	53213	34863
sigma	2.04	2.04	0.017	0.017	2.02	2.07	1	69768	31133
sigma_ID	4.30	4.30	0.102	0.101	4.14	4.47	1	89353	31028

Table 8: Median Coefficients

Model	f	a_1	b_1	c_1	b_2	c_2	σ	σ_{ID}
Longitudinal	61.13	56.75	0.14	-5.58	0.89	12.24	2.04	4.3
Cross Sectional	61.37	56.98	0.13	-7.01	0.66	11.83	4.73	NA

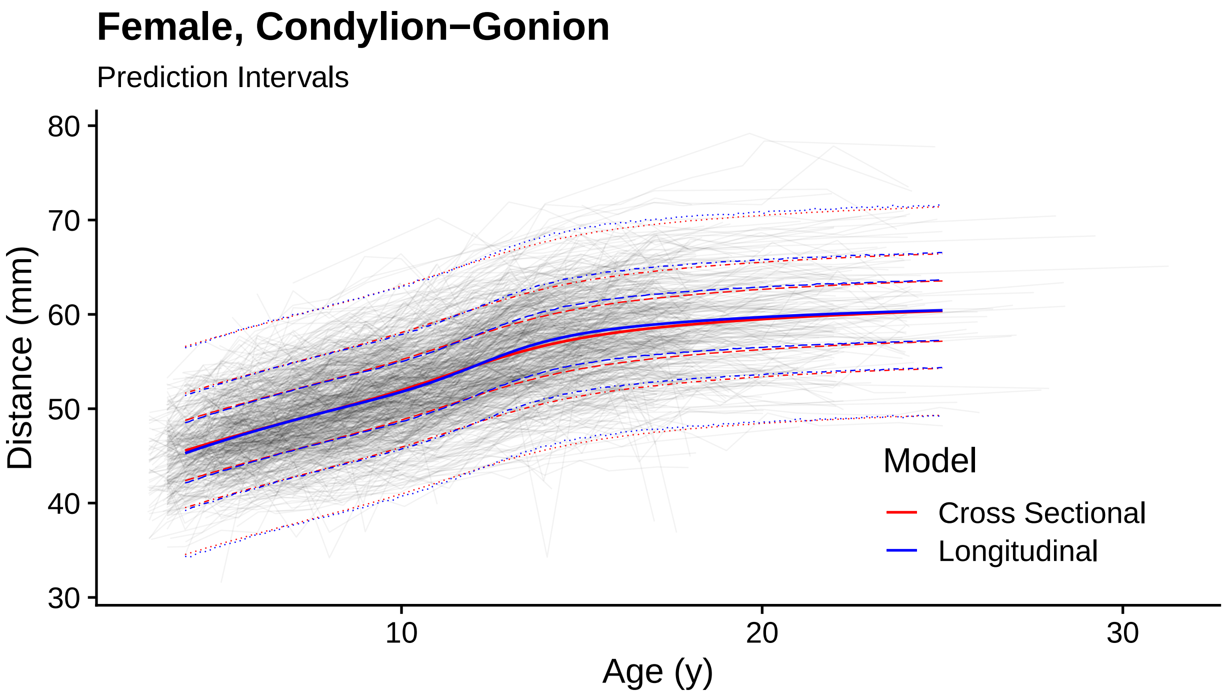
Female, Condylion−Gonion
Prediction Intervals
Distance (mm)
80
70
60
50
40
30
10
20
30
Age (y)
Model
Cross Sectional
Longitudinal

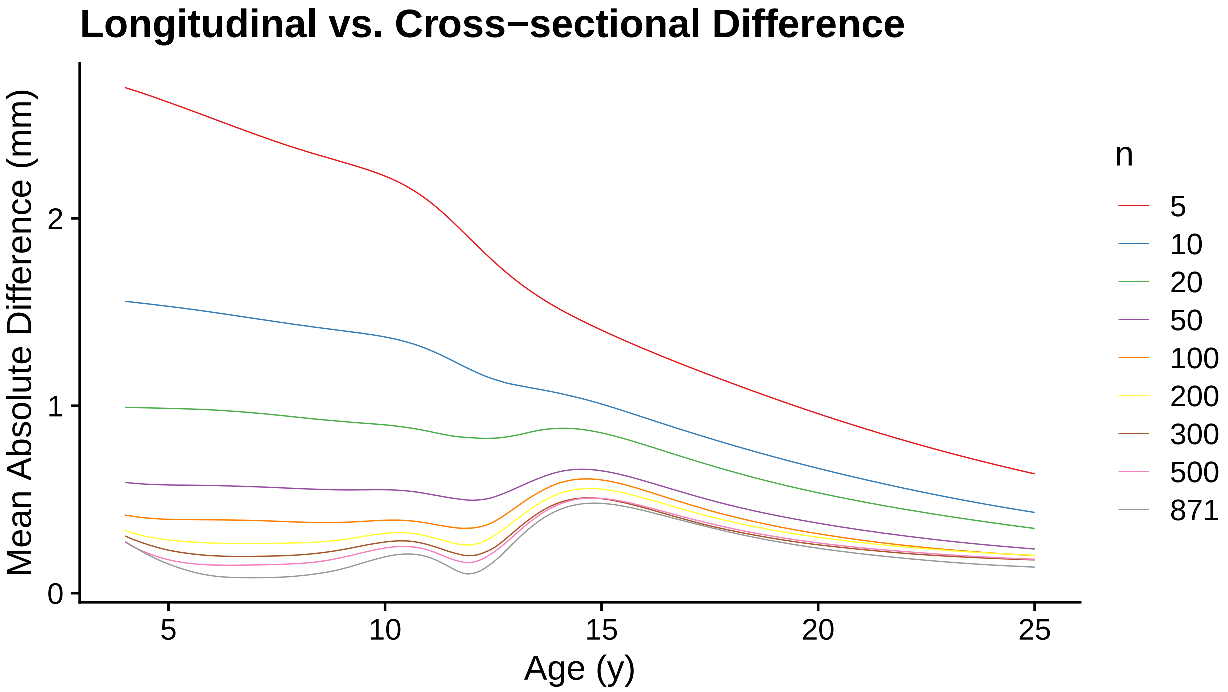
Longitudinal vs. Cross−sectional Difference
Mean Absolute Difference (mm)
2
1
0
5
10
15
20
25
Age (y)
n
5
10
20
50
100
200
300
500
871

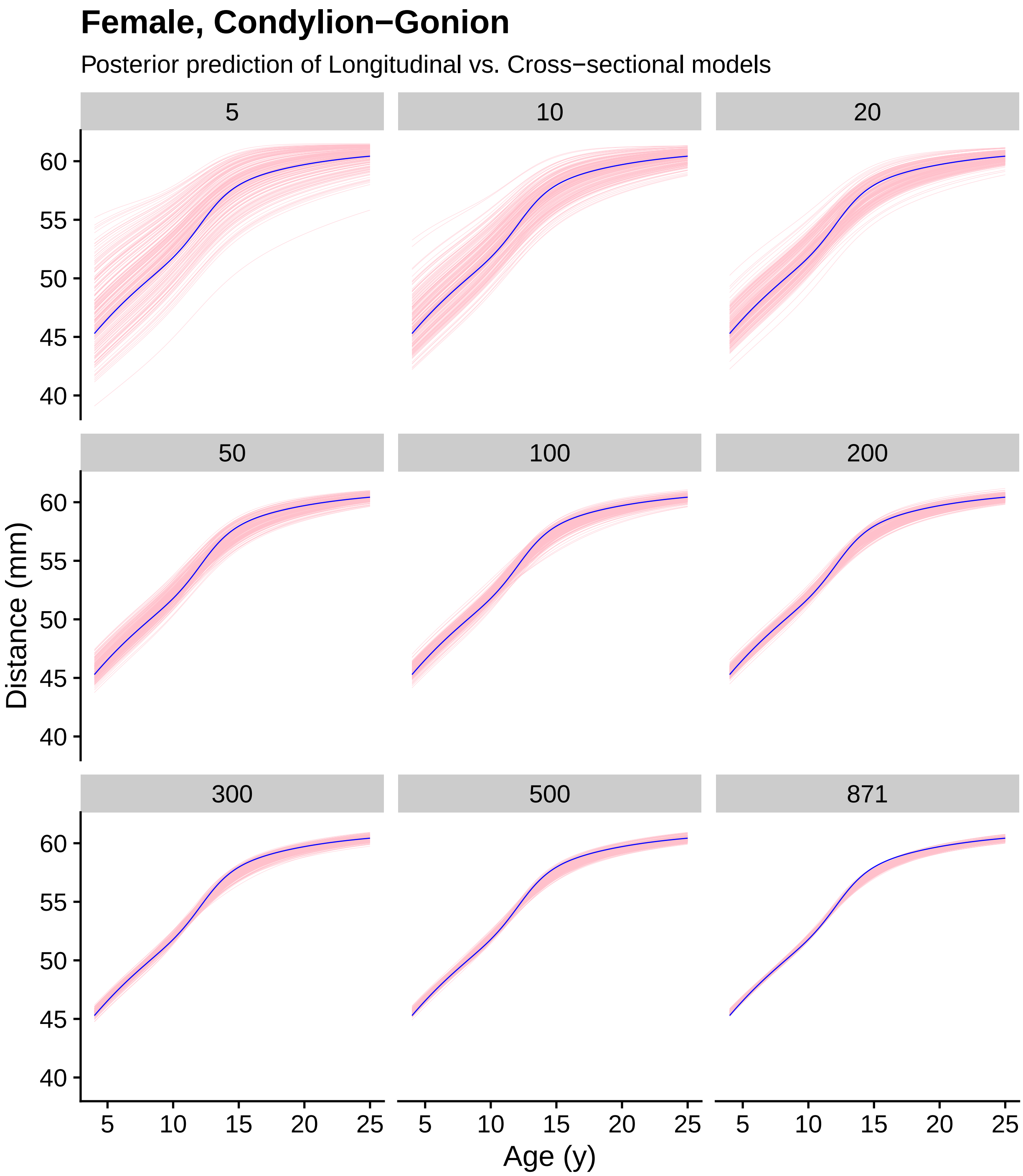
Female, Condylion−Gonion
Posterior prediction of Longitudinal vs. Cross−sectional models
5
10
20
50
100
200
300
500
871
Distance (mm)
Age (y)

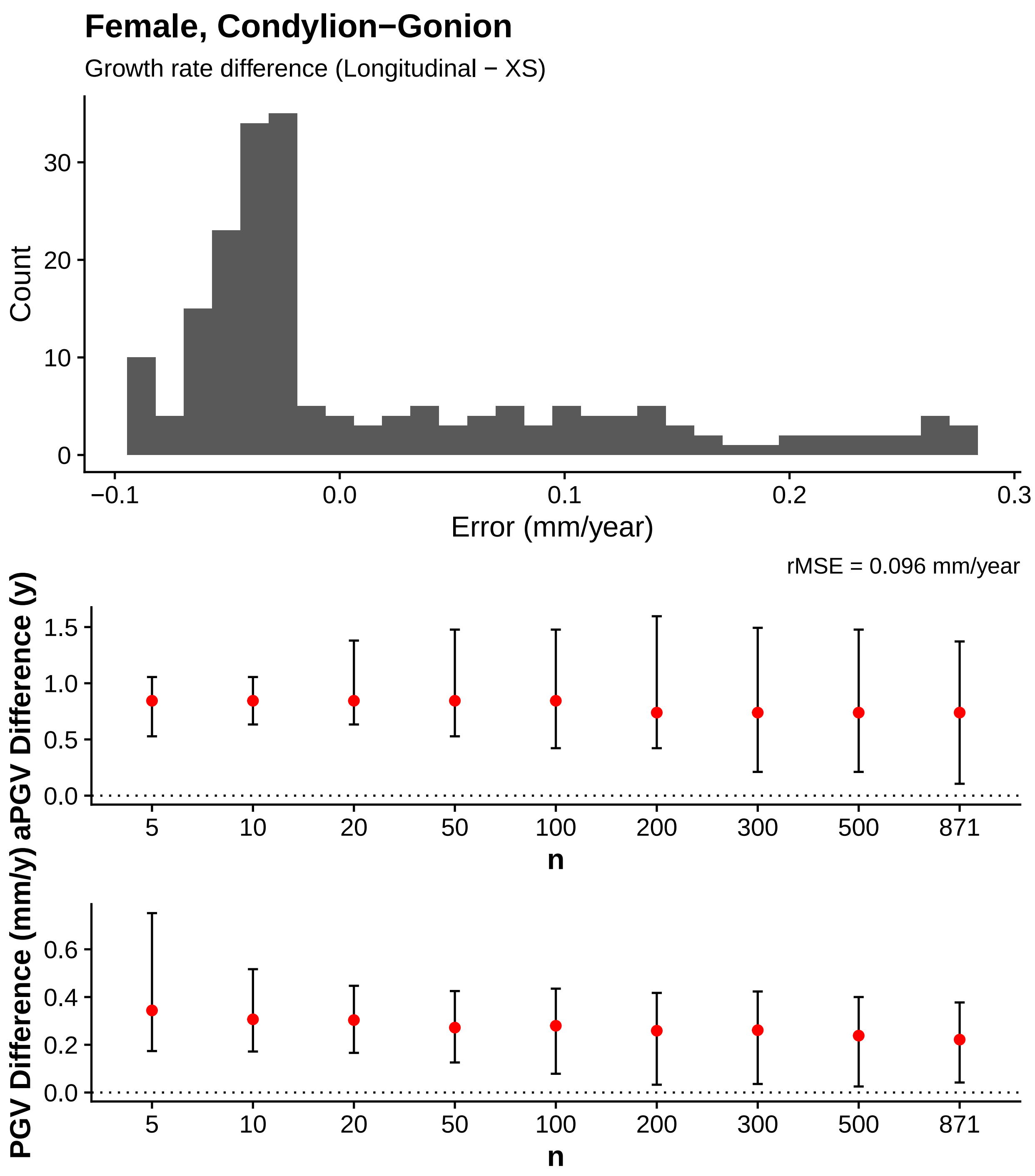

Female, Condylion−Gonion
Growth rate difference (Longitudinal − XS)
Count
30
20
10
0
−0.1
0.0
0.1
0.2
0.3
Error (mm/year)
rMSE = 0.096 mm/year
aPGV Difference (y)
1.5
1.0
0.5
0.0
5
10
20
50
100
200
300
500
871
n
PGV Difference (mm/y)
0.6
0.4
0.2
0.0
5
10
20
50
100
200
300
500
871
n

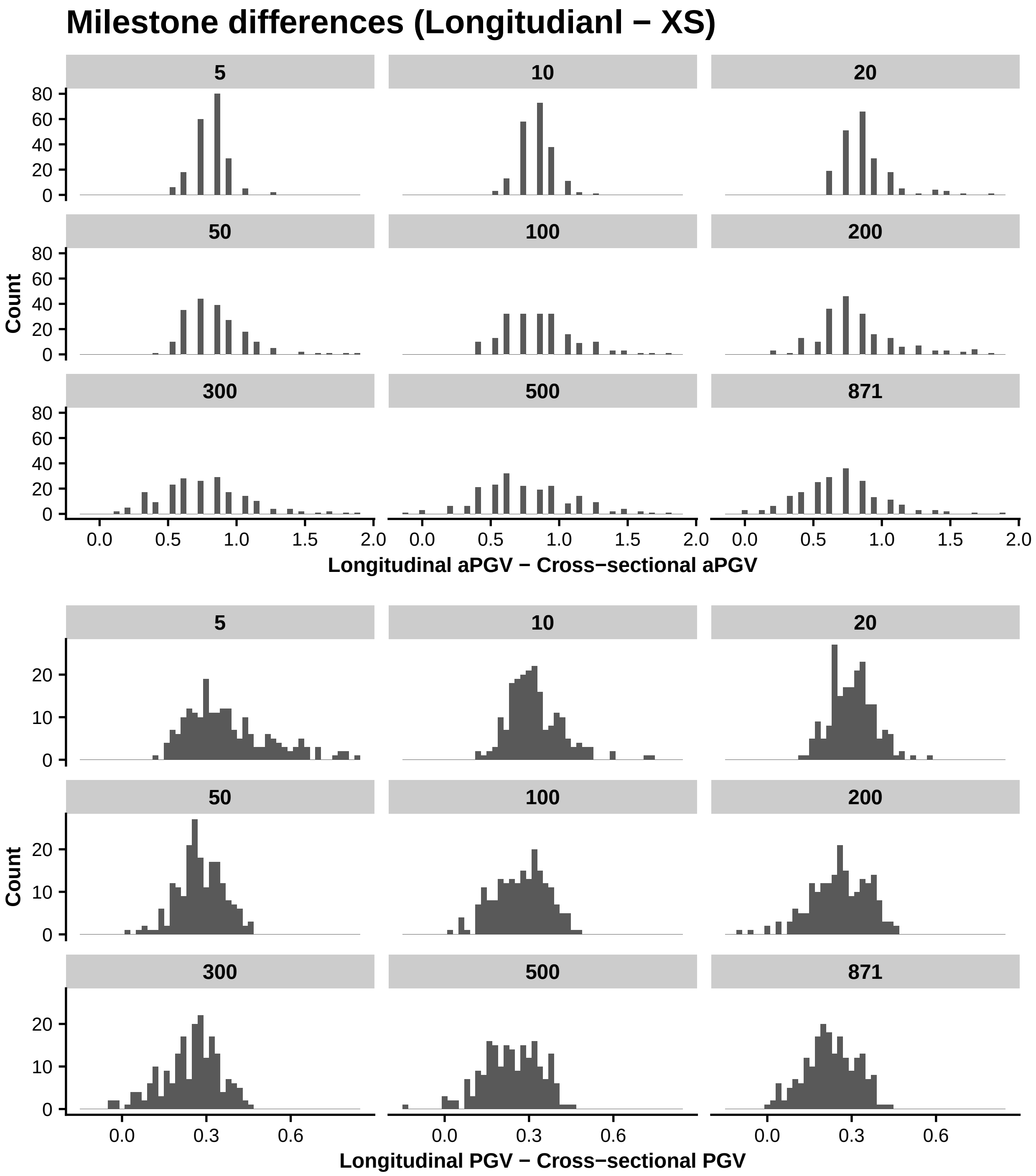
Milestone differences (Longitudianl − XS)
5
10
20
50
100
200
300
500
871
Count
Longitudinal aPGV − Cross−sectional aPGV
Longitudinal PGV − Cross−sectional PGV

6 Female, Condylion-Pogonion

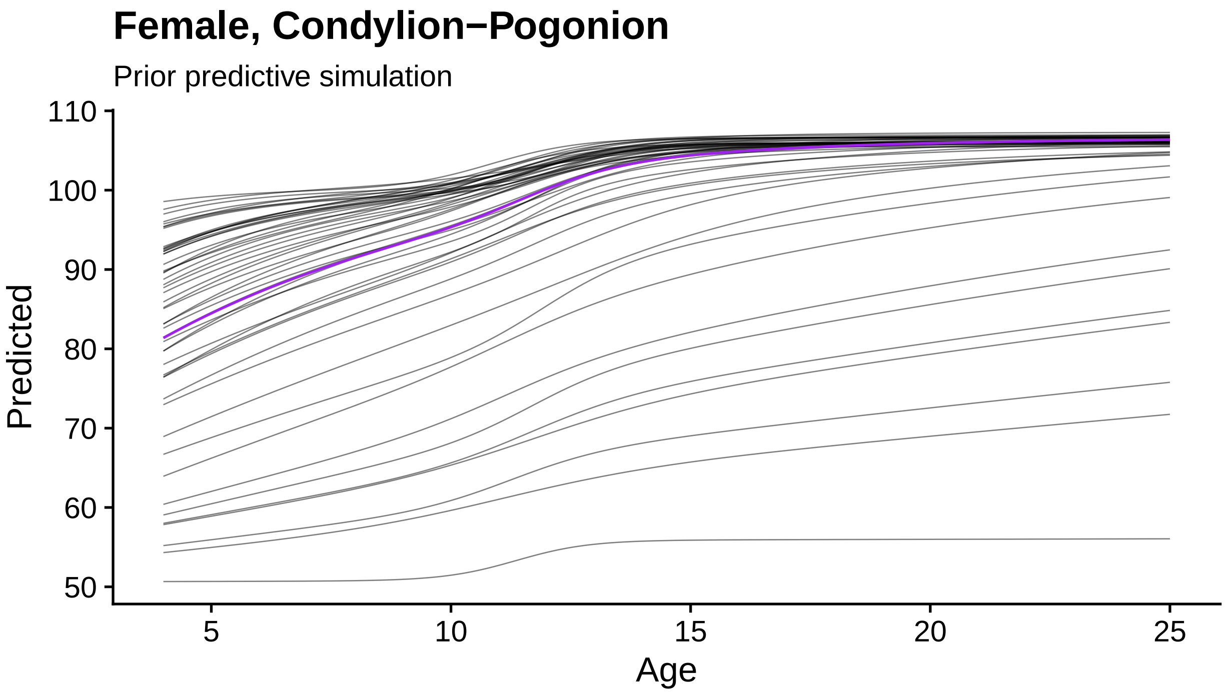

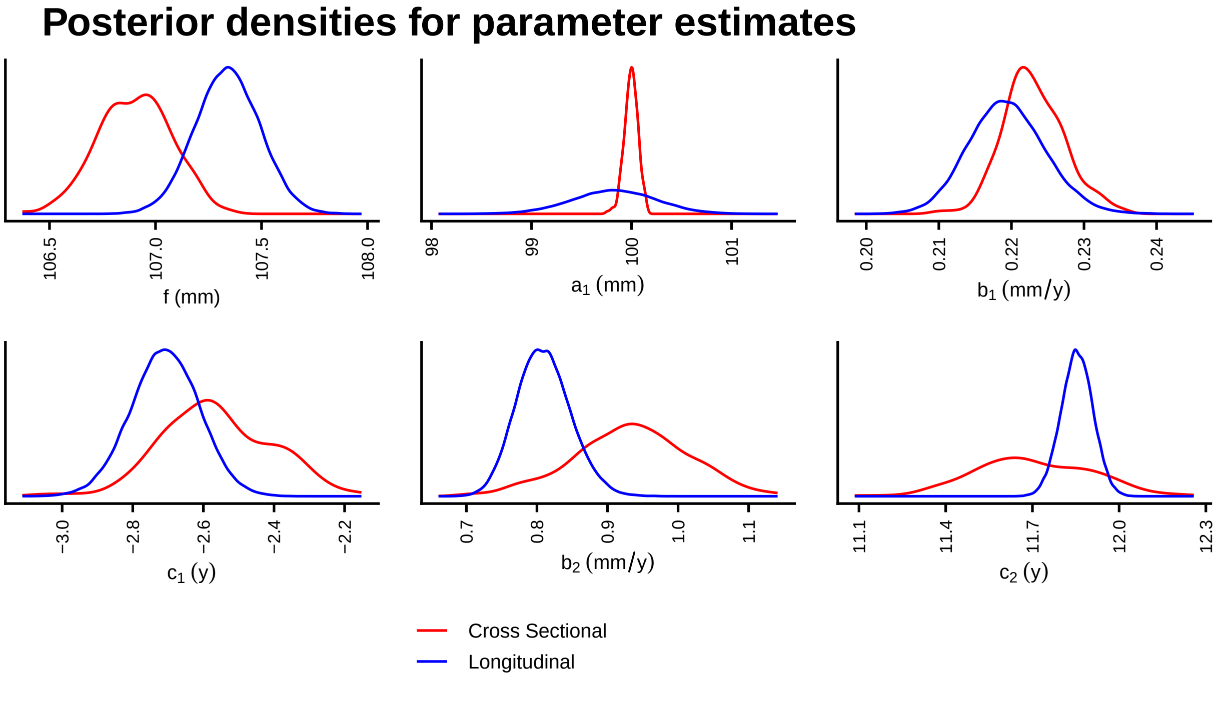

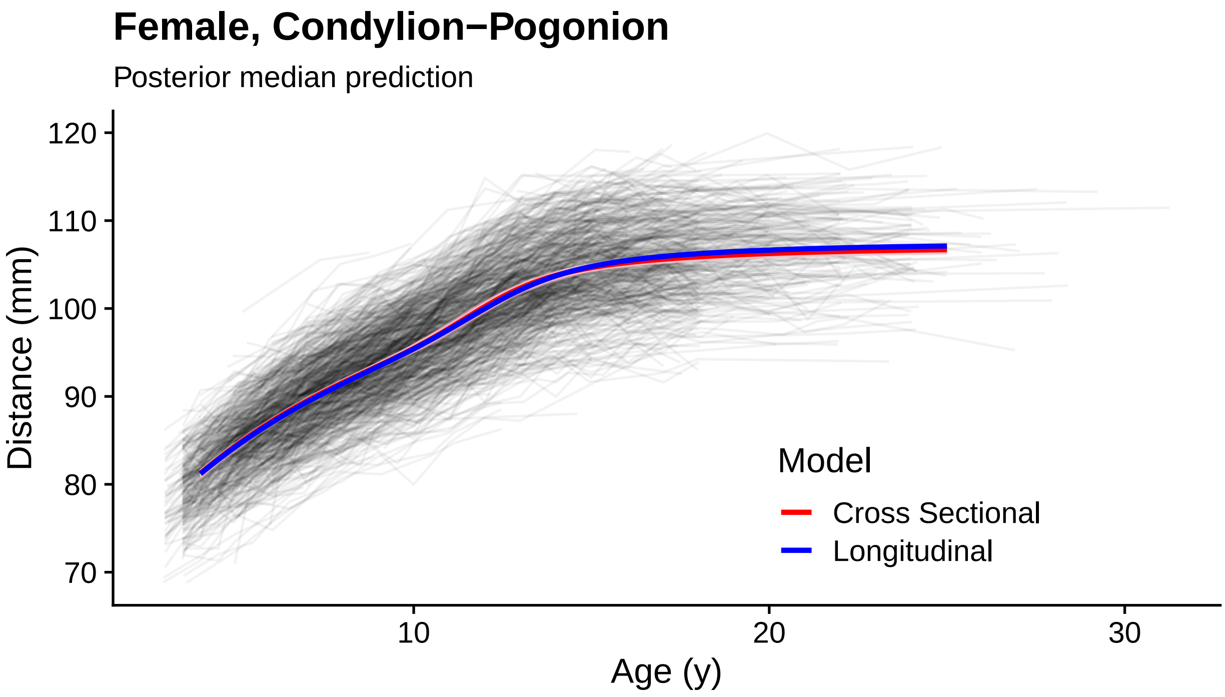

Female, Condylion−Pogonion
Posterior median prediction
Distance (mm)
120
110
100
90
80
70
10
20
30
Age (y)
Model
Cross Sectional
Longitudinal

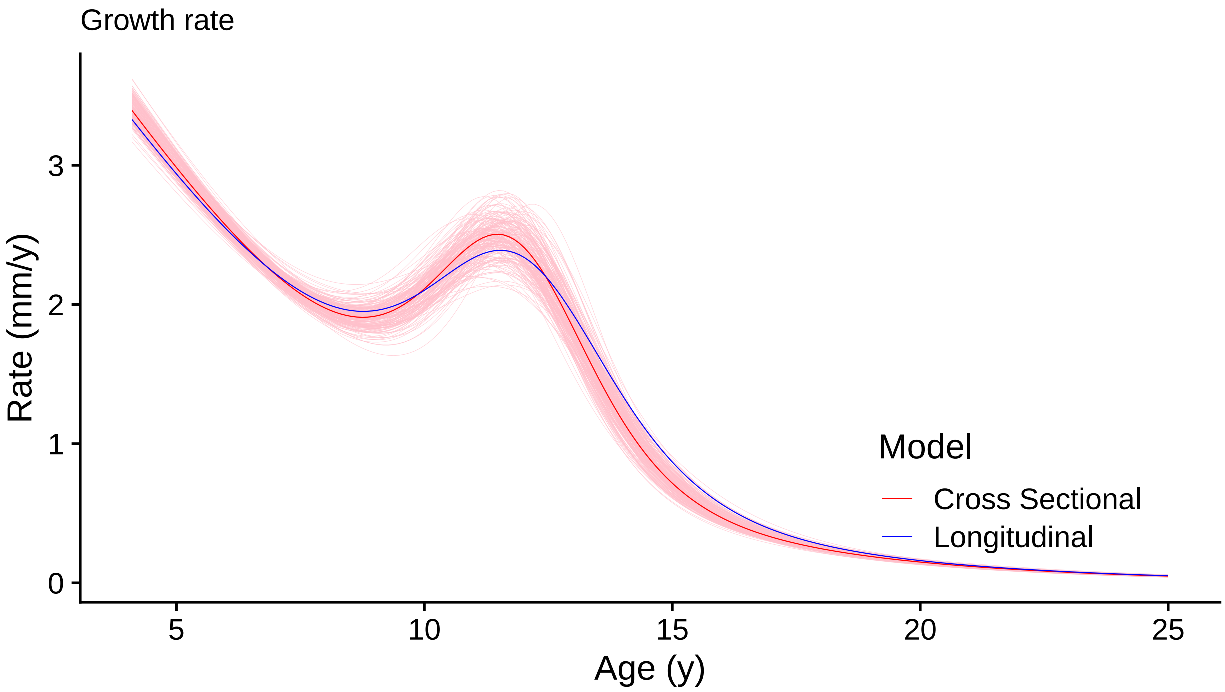

Growth rate
Rate (mm/y)
3
2
1
0
5
10
15
20
25
Age (y)
Model
Cross Sectional
Longitudinal

Table 9: Longitudinal Model Summary

Parameter	Mean	Median	Std. Dev.	MAD	5%	95%	$\hat{r}$	Bulk ESS	Tail ESS
f	107.34	107.34	0.153	0.153	107.09	107.59	1	3103	7824
a1	99.82	99.83	0.419	0.417	99.13	100.50	1	29088	28628
b1	0.22	0.22	0.005	0.005	0.21	0.23	1	19678	26713
c1	-2.71	-2.71	0.097	0.097	-2.87	-2.55	1	34433	30117
b2	0.81	0.81	0.038	0.038	0.75	0.88	1	27407	31109
c2	11.85	11.85	0.055	0.055	11.76	11.94	1	52745	32320
sigma	1.91	1.91	0.016	0.016	1.88	1.93	1	64020	30933
sigma_ID	4.03	4.03	0.096	0.095	3.88	4.19	1	76520	30854

Table 10: Median Coefficients

Model	f	a_1	b_1	c_1	b_2	c_2	σ	σ_{ID}
Longitudinal	107.34	99.83	0.22	-2.71	0.81	11.85	1.91	4.03
Cross Sectional	106.91	100.00	0.22	-2.58	0.93	11.69	4.45	NA

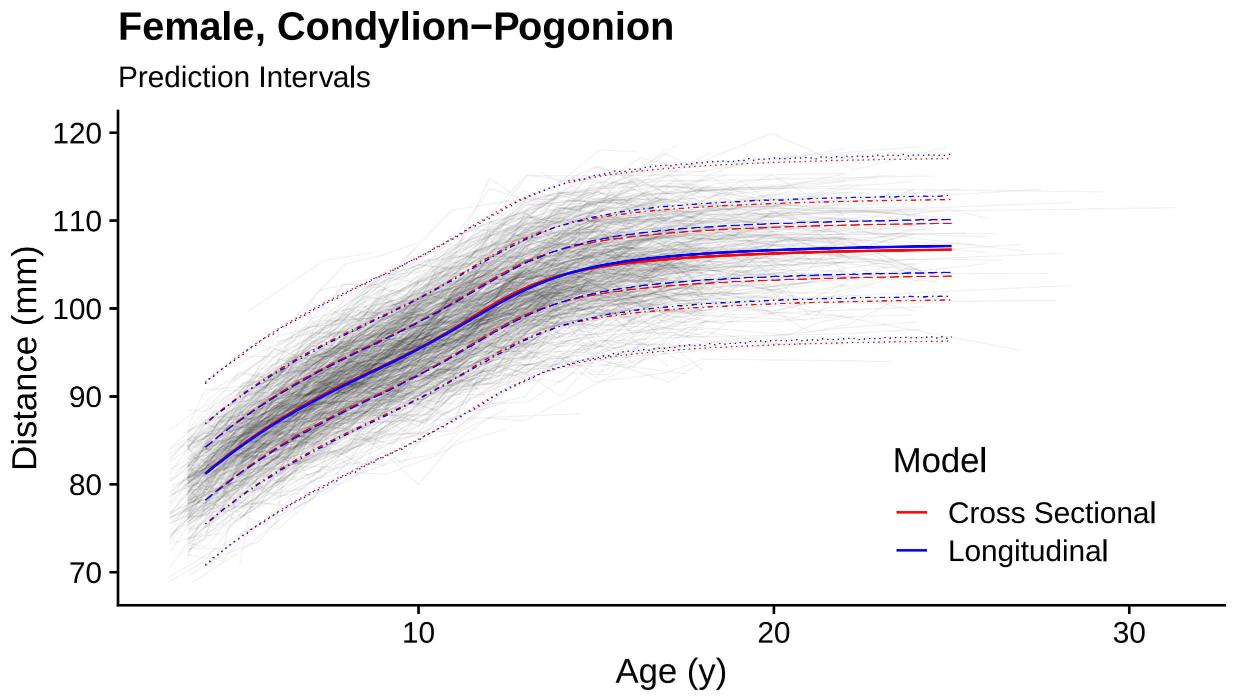
Female, Condylion−Pogonion
Prediction Intervals
Distance (mm)
120
110
100
90
80
70
10
20
30
Age (y)
Model
Cross Sectional
Longitudinal

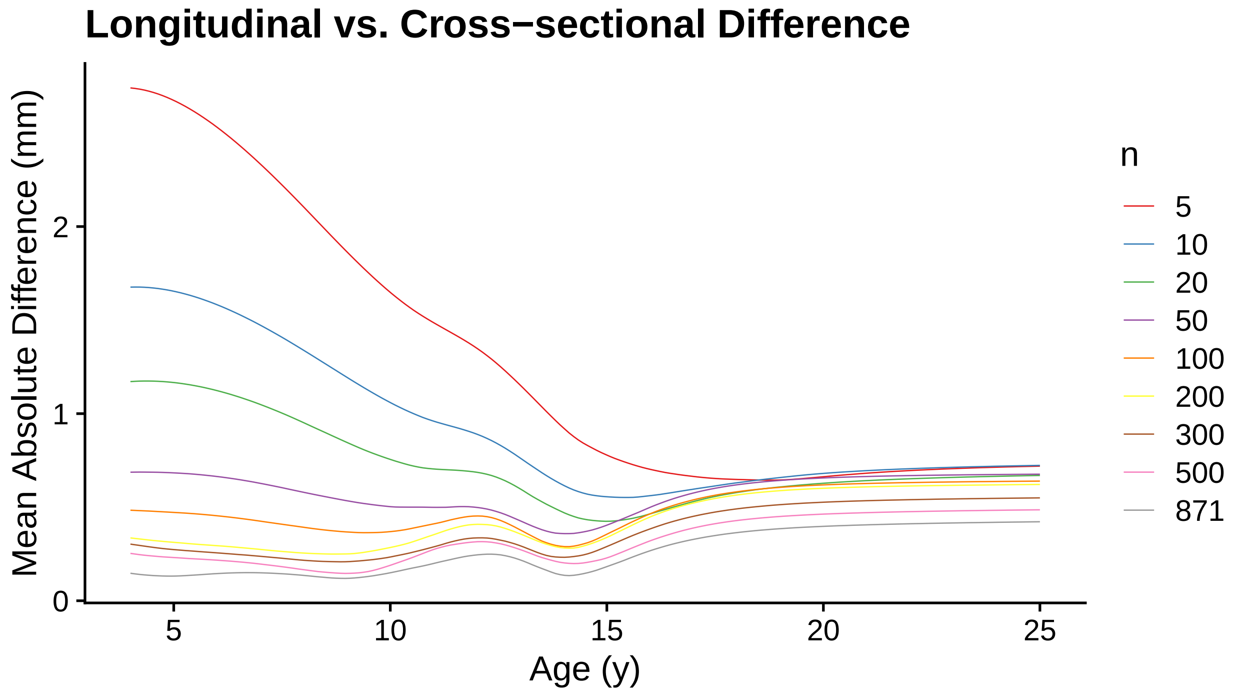
Longitudinal vs. Cross−sectional Difference
Mean Absolute Difference (mm)
2
1
0
5
10
15
20
25
Age (y)
n
5
10
20
50
100
200
300
500
871

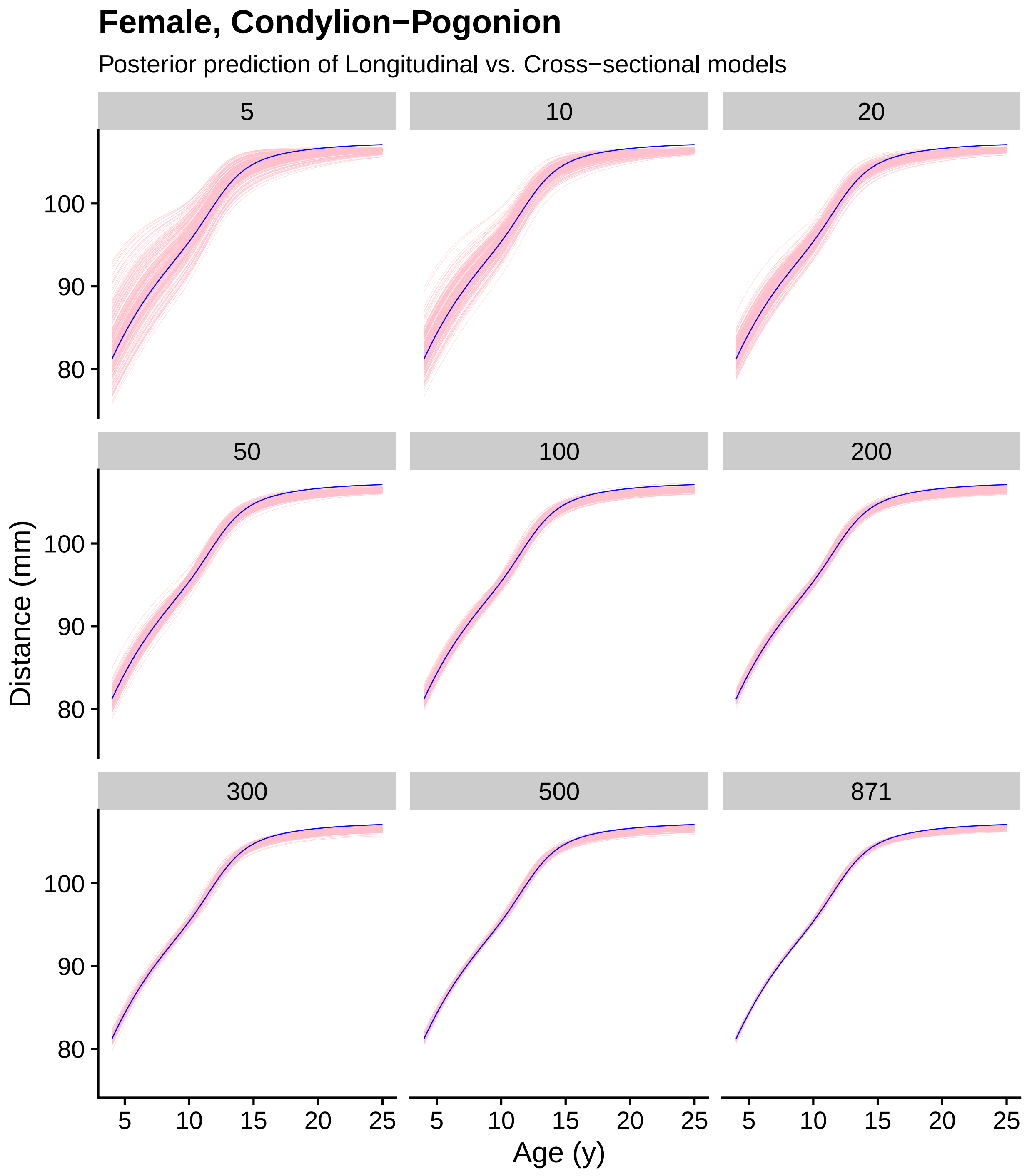

Female, Condylion−Pogonion
Posterior prediction of Longitudinal vs. Cross−sectional models
5
10
20
50
100
200
300
500
871
Distance (mm)
100
90
80
5
10
15
20
25
Age (y)

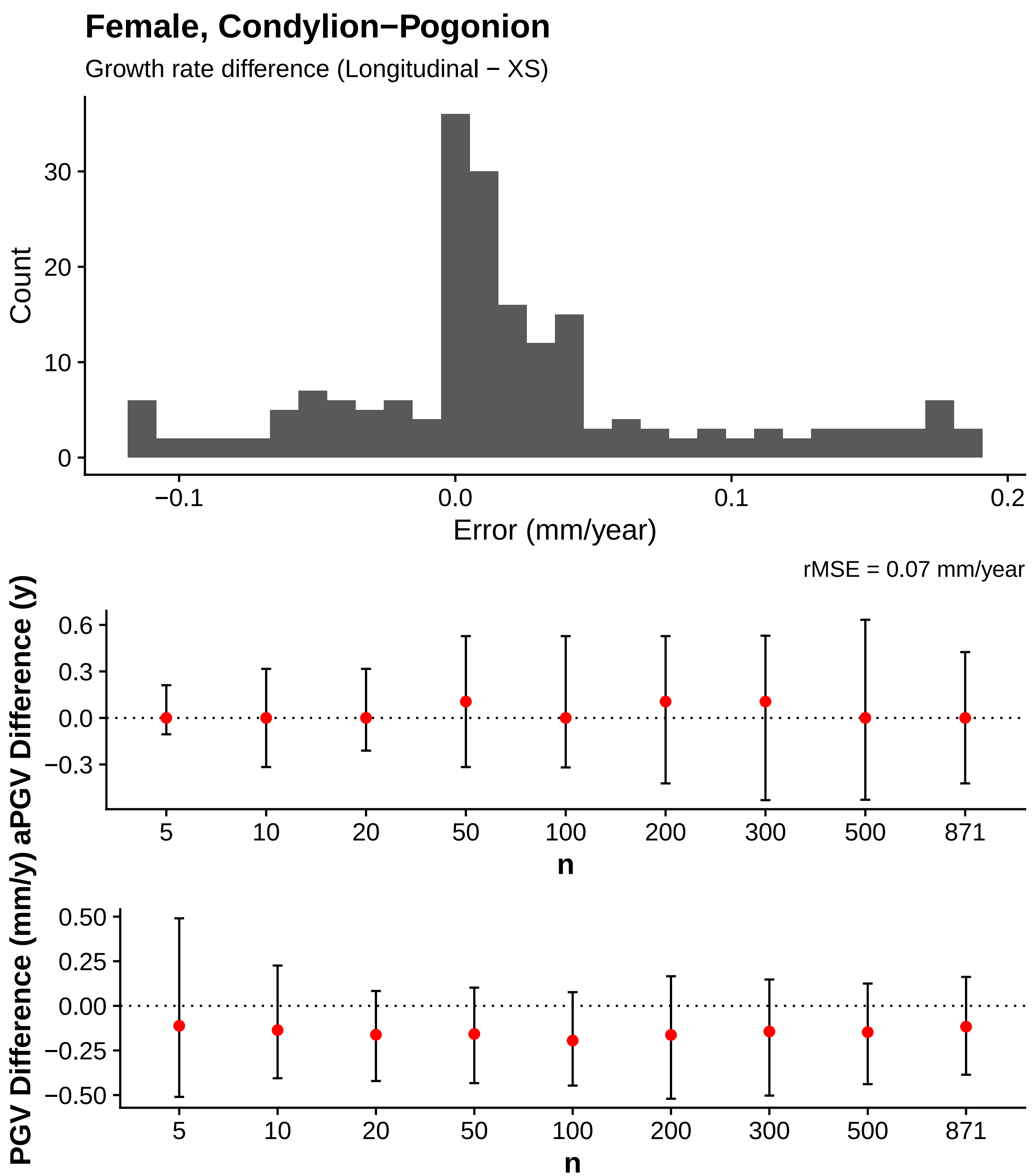
Female, Condylion−Pogonion
Growth rate difference (Longitudinal − XS)
Count
30
20
10
0
−0.1
0.0
0.1
0.2
Error (mm/year)
rMSE = 0.07 mm/year
0.6
0.3
0.0
−0.3
5
10
20
50
100
200
300
500
871
n
0.50
0.25
0.00
−0.25
−0.50
PGV Difference (mm/y) aPGV Difference (y)

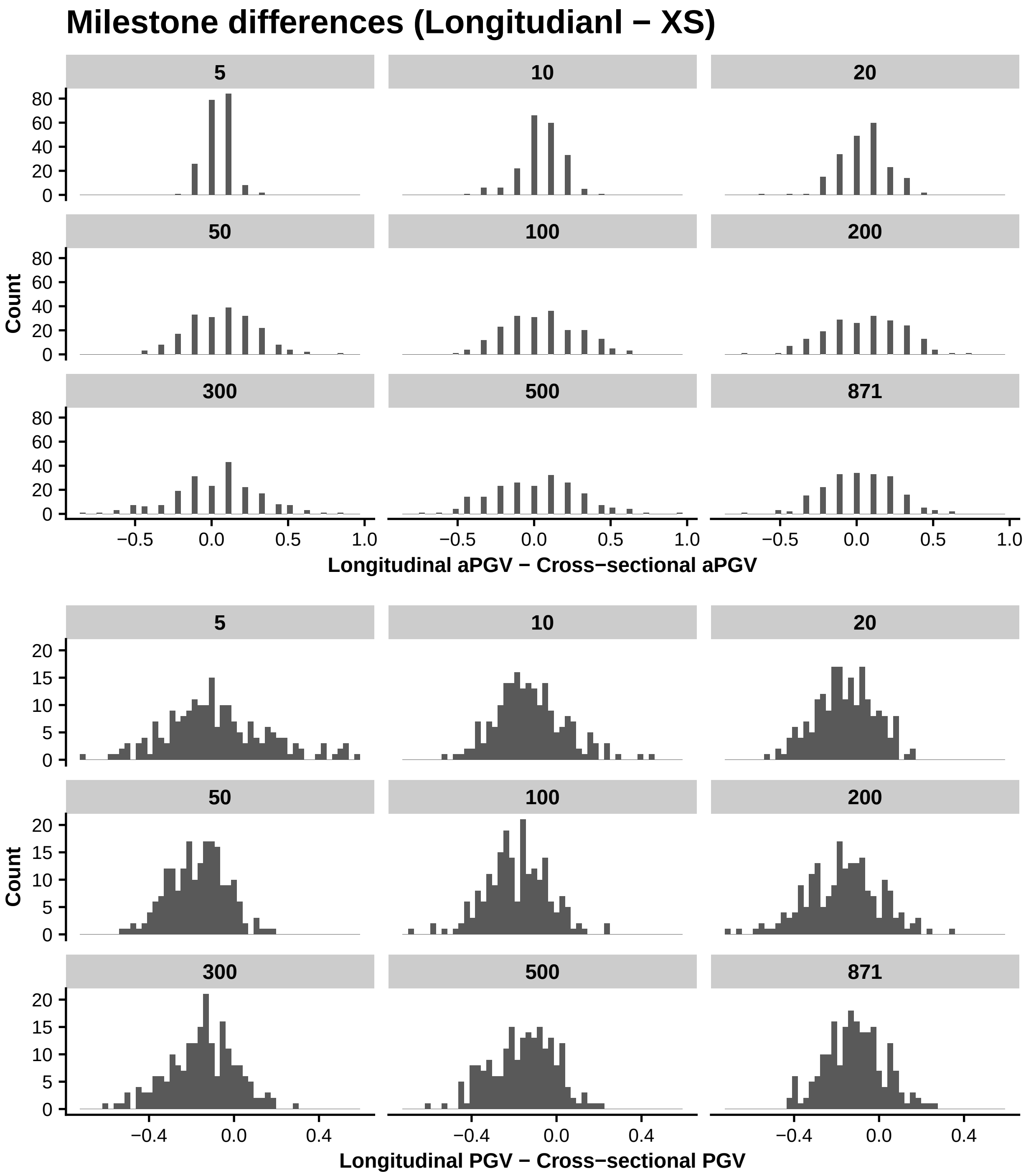
Milestone differences (Longitudianl − XS)
5
10
20
50
100
200
300
500
871
Count
Longitudinal aPGV − Cross−sectional aPGV
Longitudinal PGV − Cross−sectional PGV

7 Female, Gonion-Pogonion

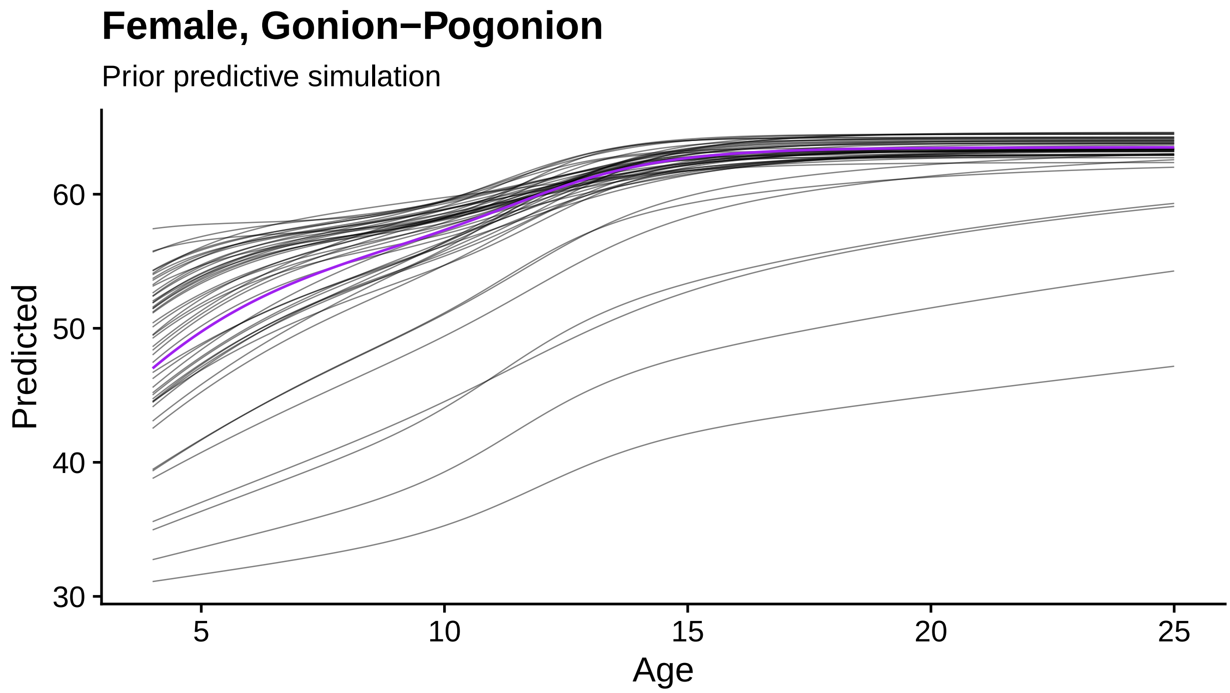

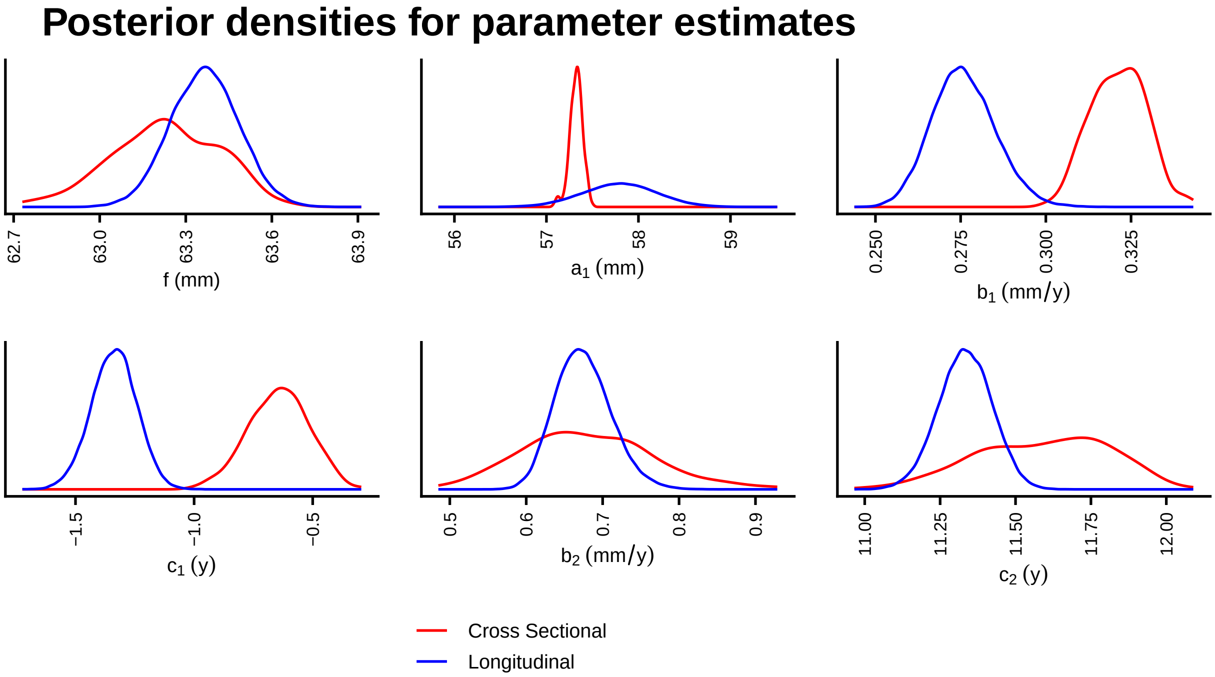

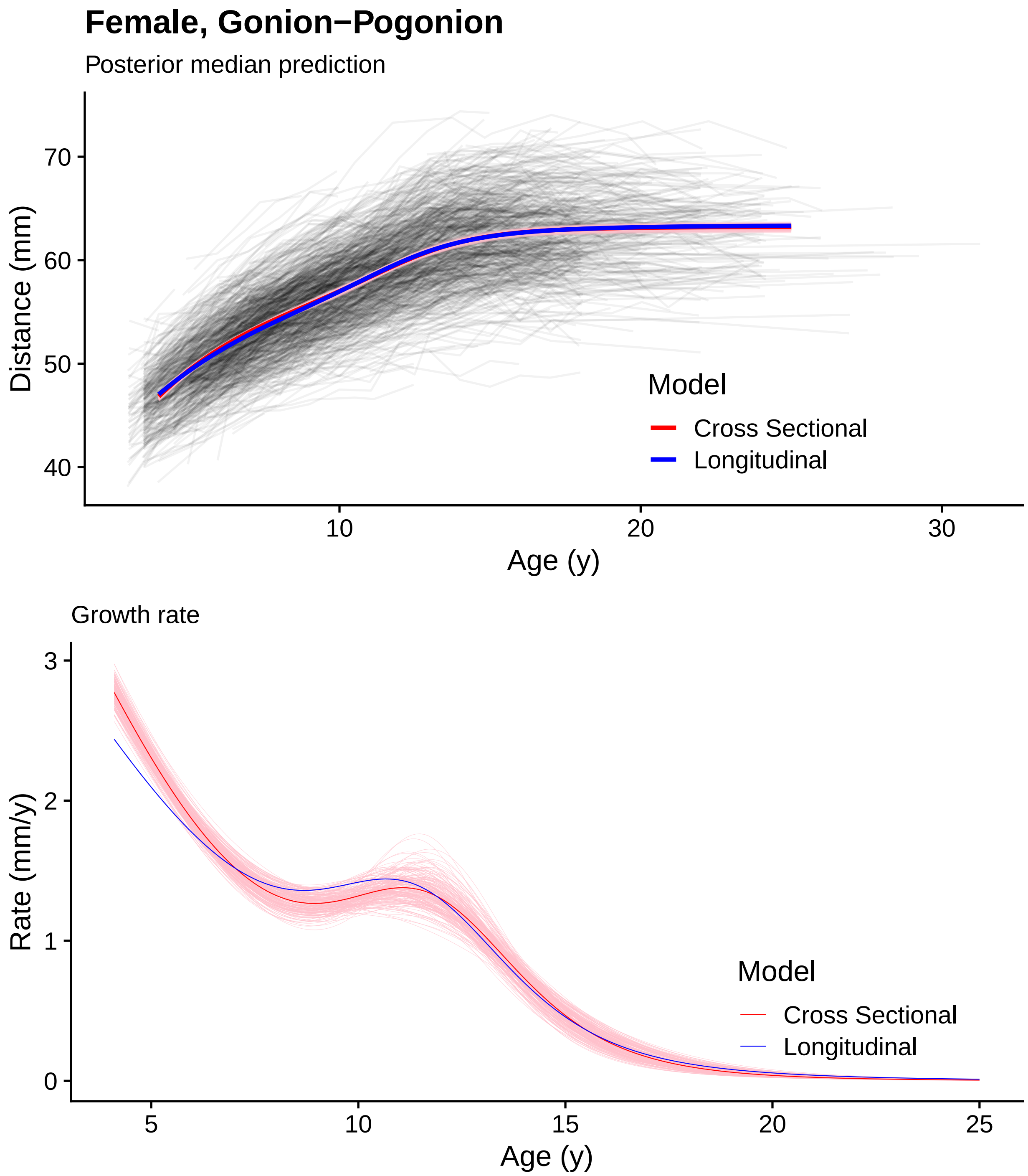
Female, Gonion−Pogonion
Posterior median prediction
Distance (mm)
70
60
50
40
10
20
30
Age (y)
Model
Cross Sectional
Longitudinal
Growth rate
Rate (mm/y)
3
2
1
0
5
10
15
20
25
Age (y)
Model
Cross Sectional
Longitudinal

Table 11: Longitudinal Model Summary

Parameter	Mean	Median	Std. Dev.	MAD	5%	95%	$\hat{r}$	Bulk ESS	Tail ESS
f	63.37	63.37	0.119	0.120	63.17	63.56	1	1017	2923
a1	57.79	57.80	0.405	0.401	57.12	58.45	1	14862	21684
b1	0.28	0.28	0.009	0.009	0.26	0.29	1	12032	21691
c1	-1.34	-1.34	0.096	0.095	-1.50	-1.18	1	28763	28999
b2	0.67	0.67	0.036	0.036	0.62	0.74	1	13008	27548
c2	11.33	11.33	0.089	0.089	11.19	11.48	1	15248	25889
sigma	1.36	1.36	0.011	0.011	1.34	1.37	1	52900	30509
sigma_ID	3.49	3.49	0.081	0.081	3.36	3.63	1	58077	30971

Table 12: Median Coefficients

Model	f	a_1	b_1	c_1	b_2	c_2	σ	σ_{ID}
Longitudinal	63.37	57.80	0.28	-1.34	0.67	11.33	1.36	3.49
Cross Sectional	63.23	57.33	0.32	-0.64	0.67	11.61	3.74	NA

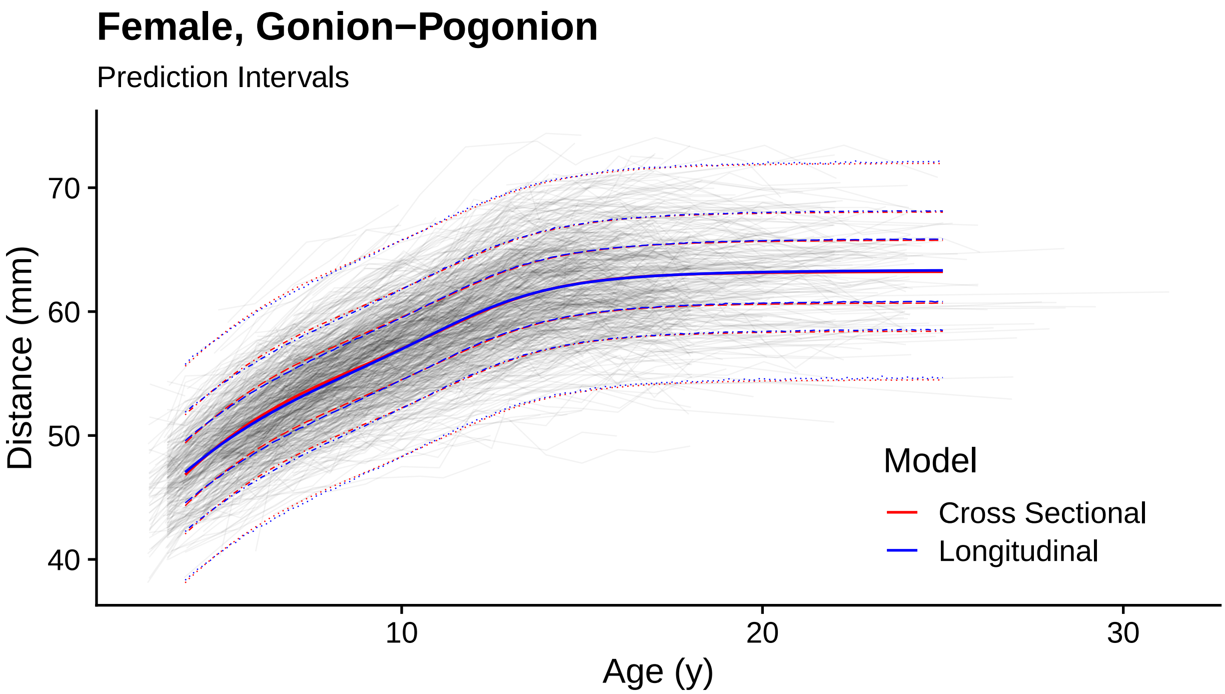
Female, Gonion−Pogonion
Prediction Intervals
Distance (mm)
70
60
50
40
10
20
30
Age (y)
Model
Cross Sectional
Longitudinal

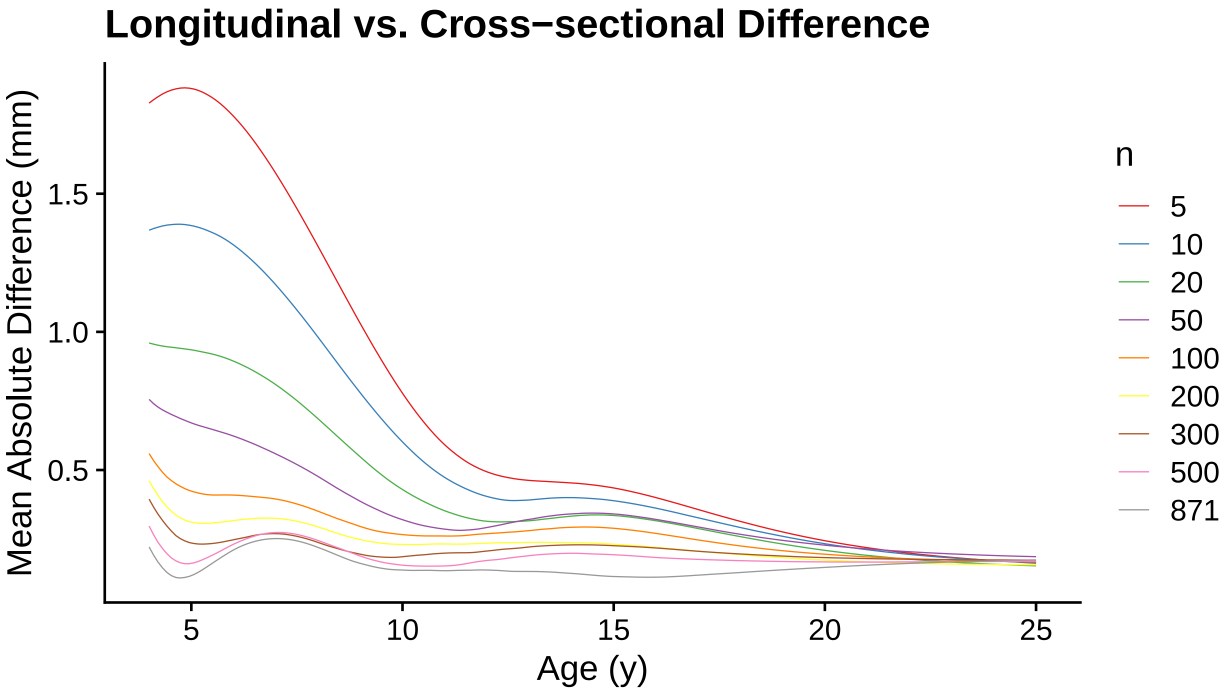
Longitudinal vs. Cross−sectional Difference
Mean Absolute Difference (mm)
1.5
1.0
0.5
5
10
15
20
25
Age (y)
n
5
10
20
50
100
200
300
500
871

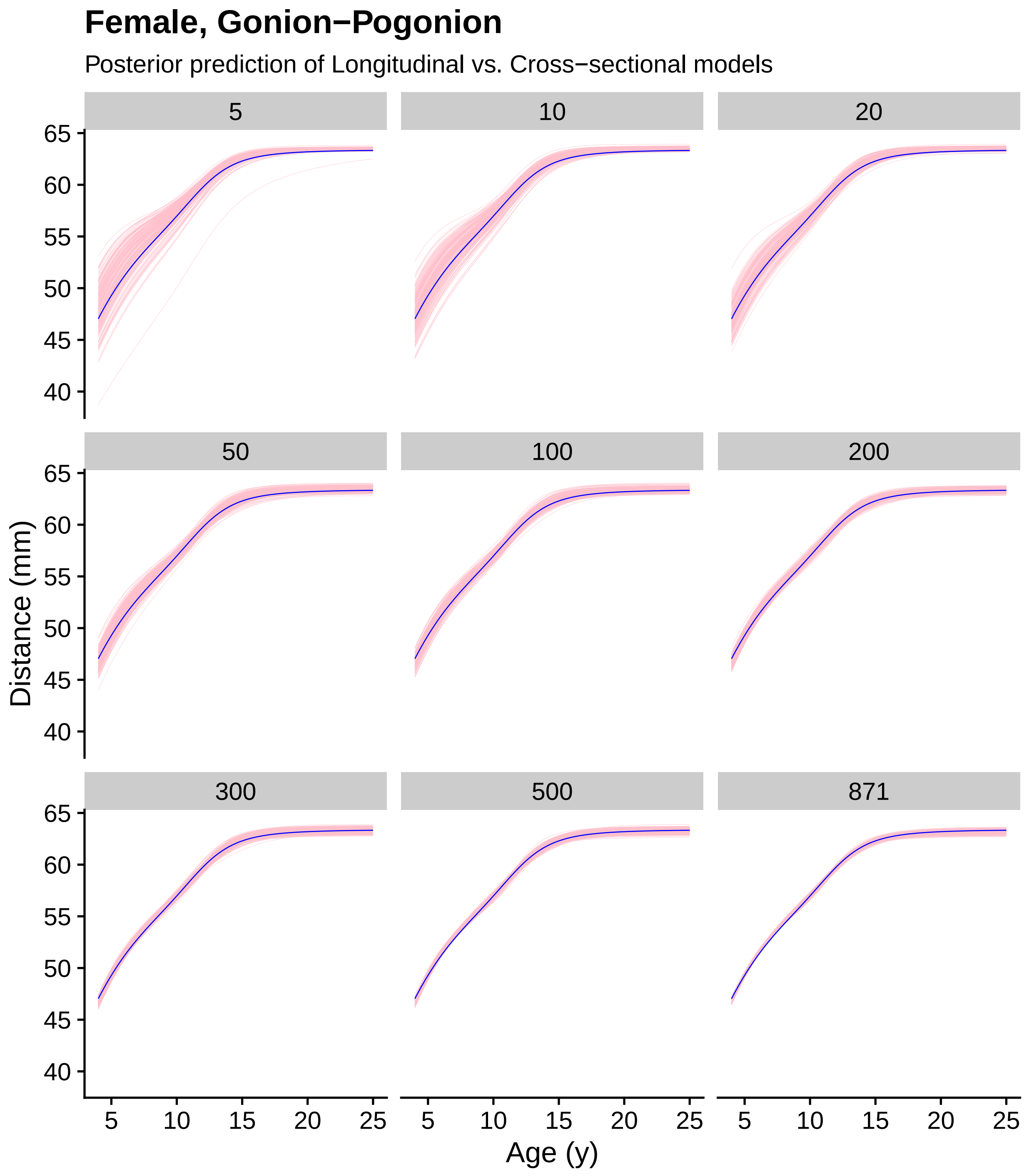
Female, Gonion−Pogonion
Posterior prediction of Longitudinal vs. Cross−sectional models
5
10
20
50
100
200
300
500
871
Distance (mm)
Age (y)
40
45
50
55
60
65
5
10
15
20
25

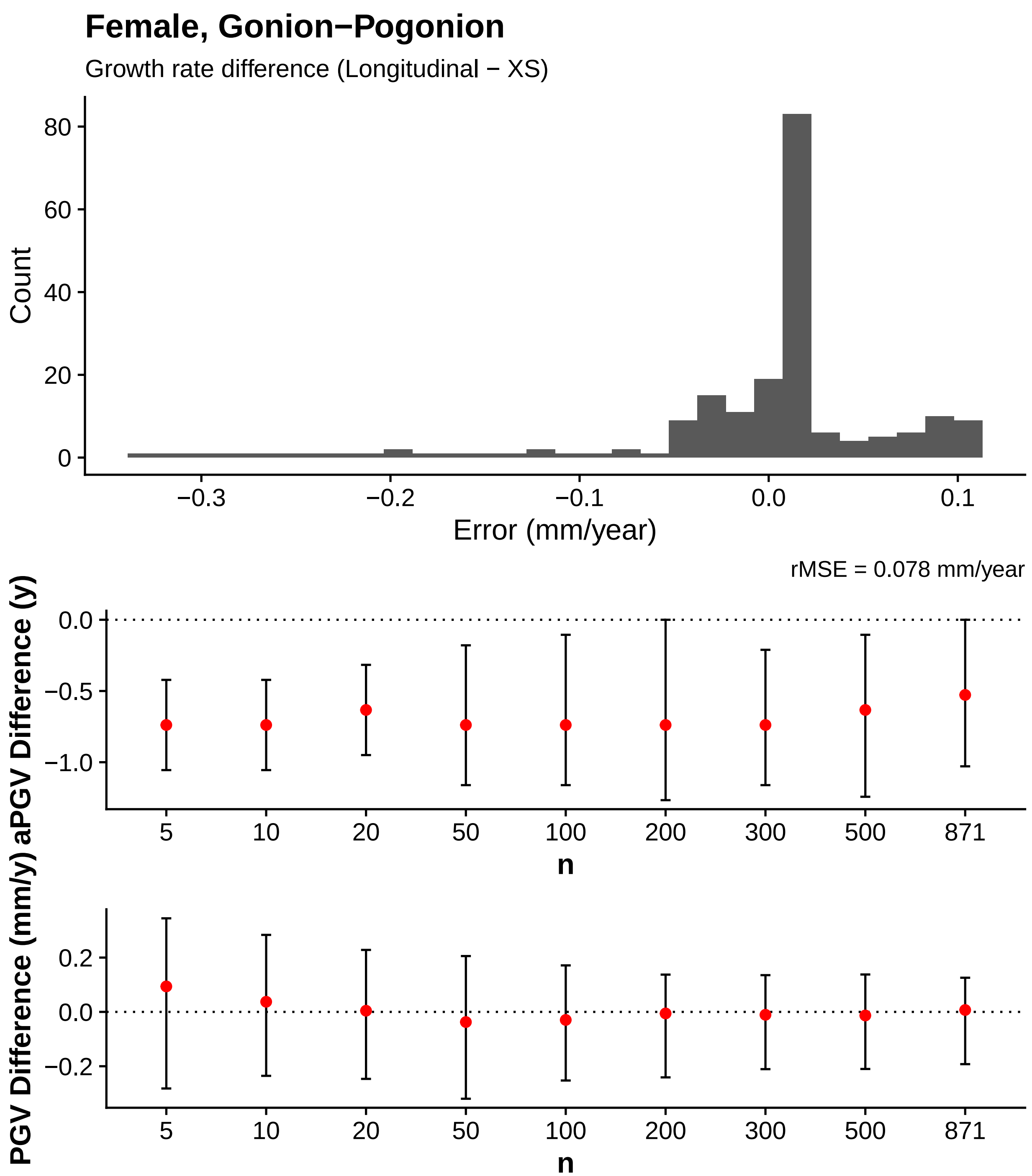
Female, Gonion−Pogonion
Growth rate difference (Longitudinal − XS)
Count
80
60
40
20
0
−0.3
−0.2
−0.1
0.0
0.1
Error (mm/year)
rMSE = 0.078 mm/year
PGV Difference (mm/y) aPGV Difference (y)
0.0
−0.5
−1.0
5
10
20
50
100
200
300
500
871
n
0.2
0.0
−0.2
5
10
20
50
100
200
300
500
871
n

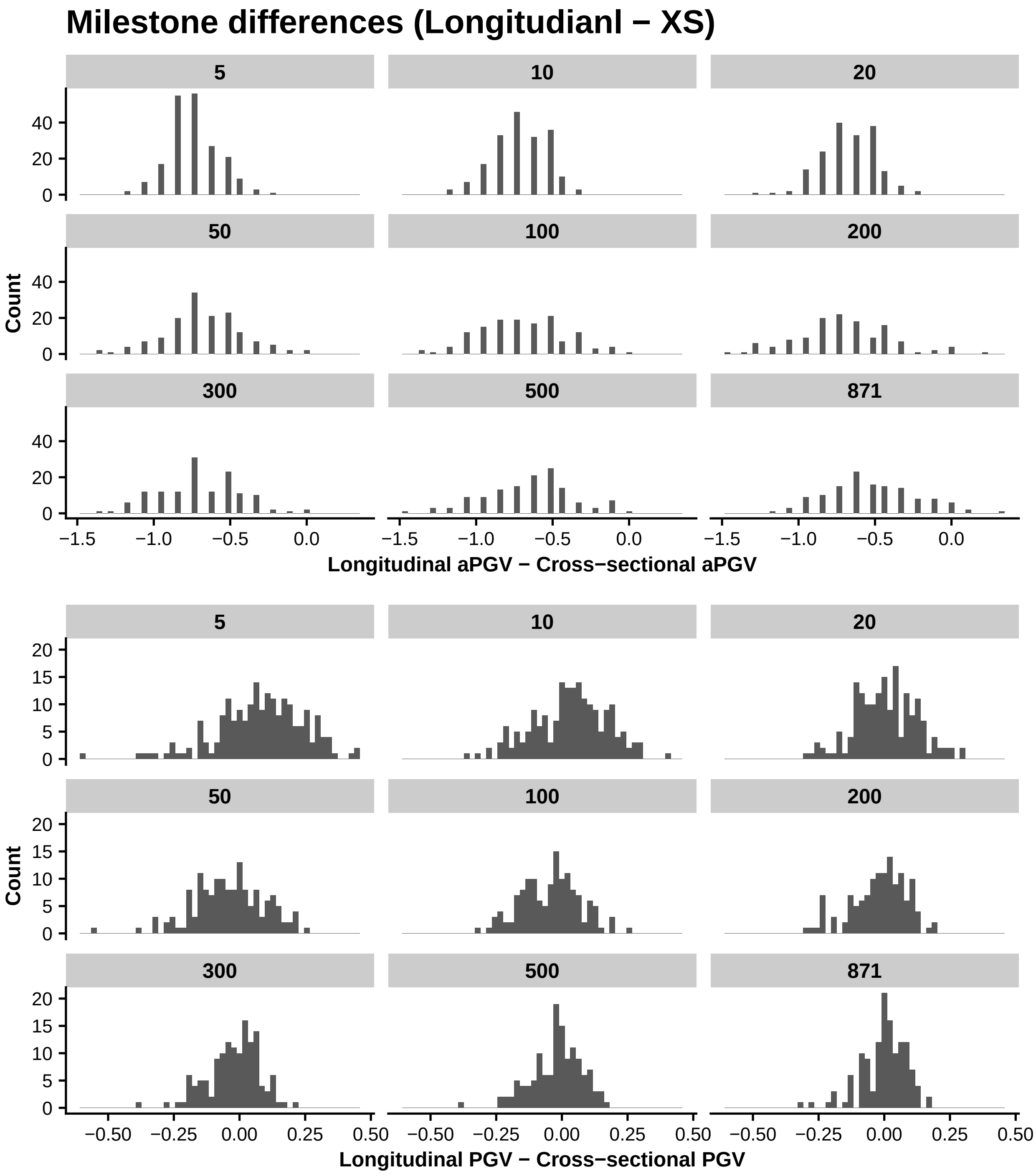
Milestone differences (Longitudianl − XS)
5
10
20
50
100
200
300
500
871
Count
Longitudinal aPGV − Cross−sectional aPGV
Longitudinal PGV − Cross−sectional PGV

8 Female, Menton-ANS

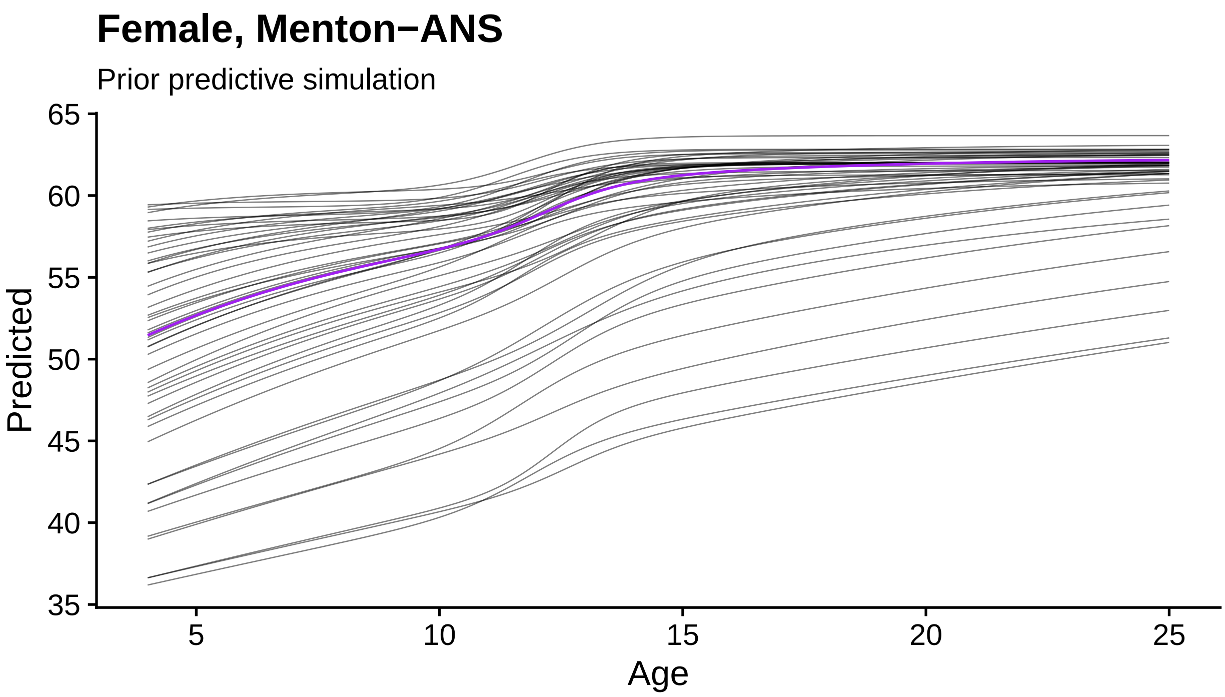

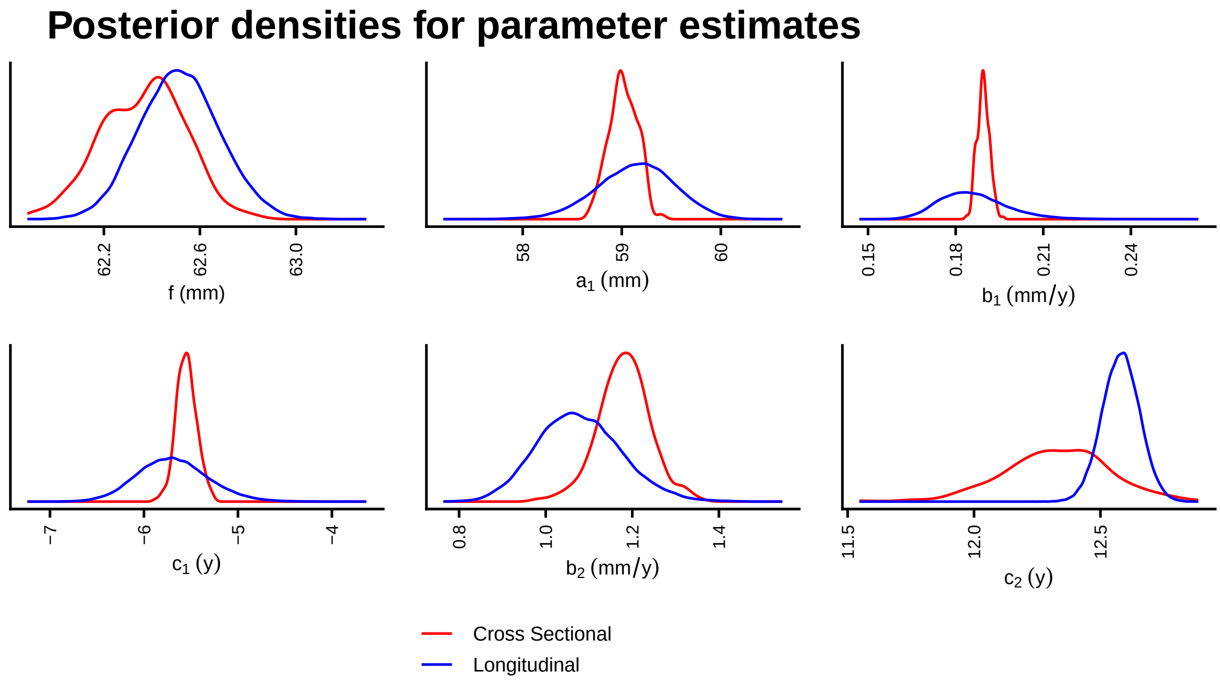

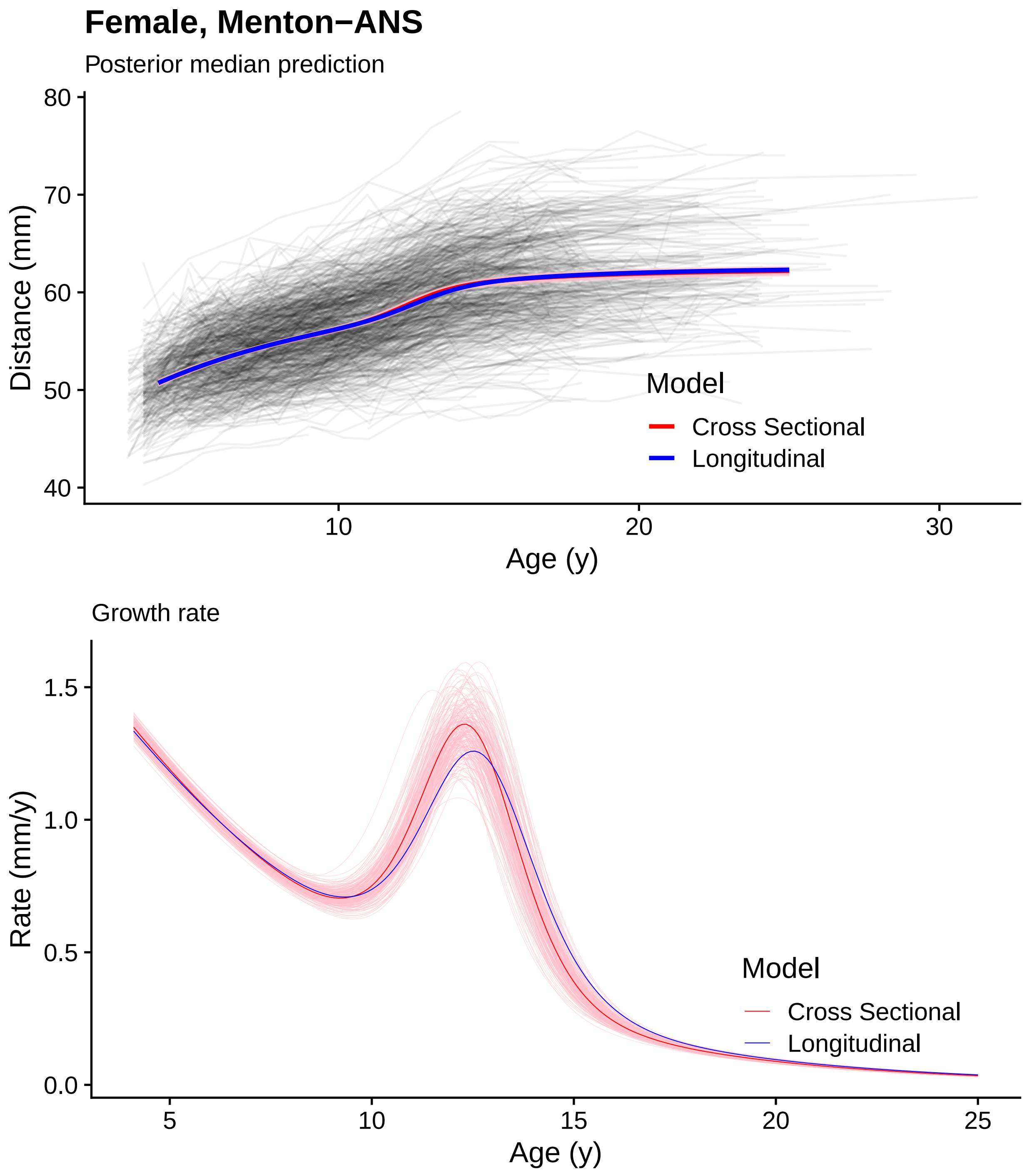

Female, Menton−ANS
Posterior median prediction
Distance (mm)
80
70
60
50
40
10
20
30
Age (y)
Model
Cross Sectional
Longitudinal
Growth rate
Rate (mm/y)
1.5
1.0
0.5
0.0
5
10
15
20
25
Age (y)
Model
Cross Sectional
Longitudinal

Table 13: Longitudinal Model Summary

Parameter	Mean	Median	Std. Dev.	MAD	5%	95%	$\hat{r}$	Bulk ESS	Tail ESS
f	62.52	62.51	0.167	0.167	62.25	62.80	1	6847	15475
a1	59.14	59.15	0.393	0.390	58.47	59.76	1	21568	22393
b1	0.19	0.18	0.012	0.011	0.17	0.21	1	14801	22070
c1	-5.69	-5.70	0.375	0.371	-6.28	-5.05	1	18143	23661
b2	1.08	1.07	0.097	0.097	0.93	1.24	1	23306	29114
c2	12.58	12.58	0.074	0.074	12.46	12.70	1	60284	30143
sigma	1.64	1.64	0.014	0.014	1.62	1.67	1	53979	29824
sigma_ID	3.67	3.66	0.086	0.086	3.53	3.81	1	71711	30139

Table 14: Median Coefficients

Model	f	a_1	b_1	c_1	b_2	c_2	σ	σ_{ID}
Longitudinal	62.51	59.15	0.18	-5.70	1.07	12.58	1.64	3.66
Cross Sectional	62.38	59.01	0.19	-5.55	1.18	12.33	3.98	NA

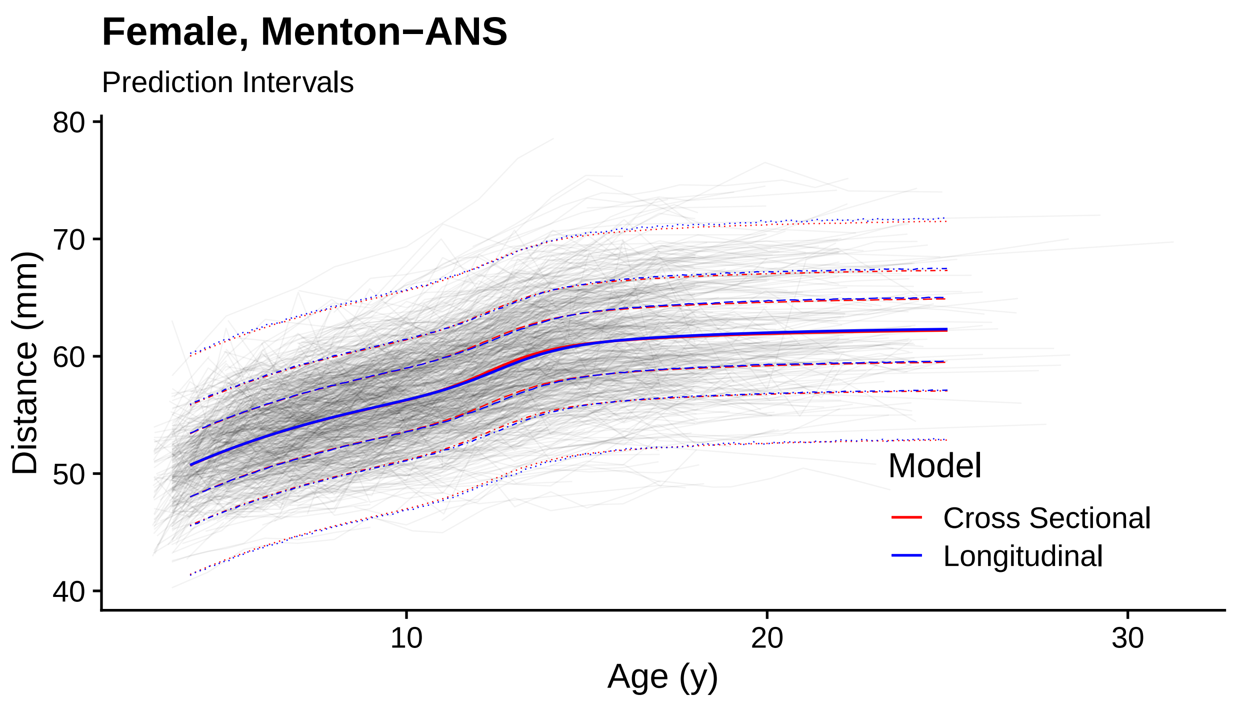
Female, Menton−ANS
Prediction Intervals
Distance (mm)
Age (y)
Model
Cross Sectional
Longitudinal
80
70
60
50
40
10
20
30

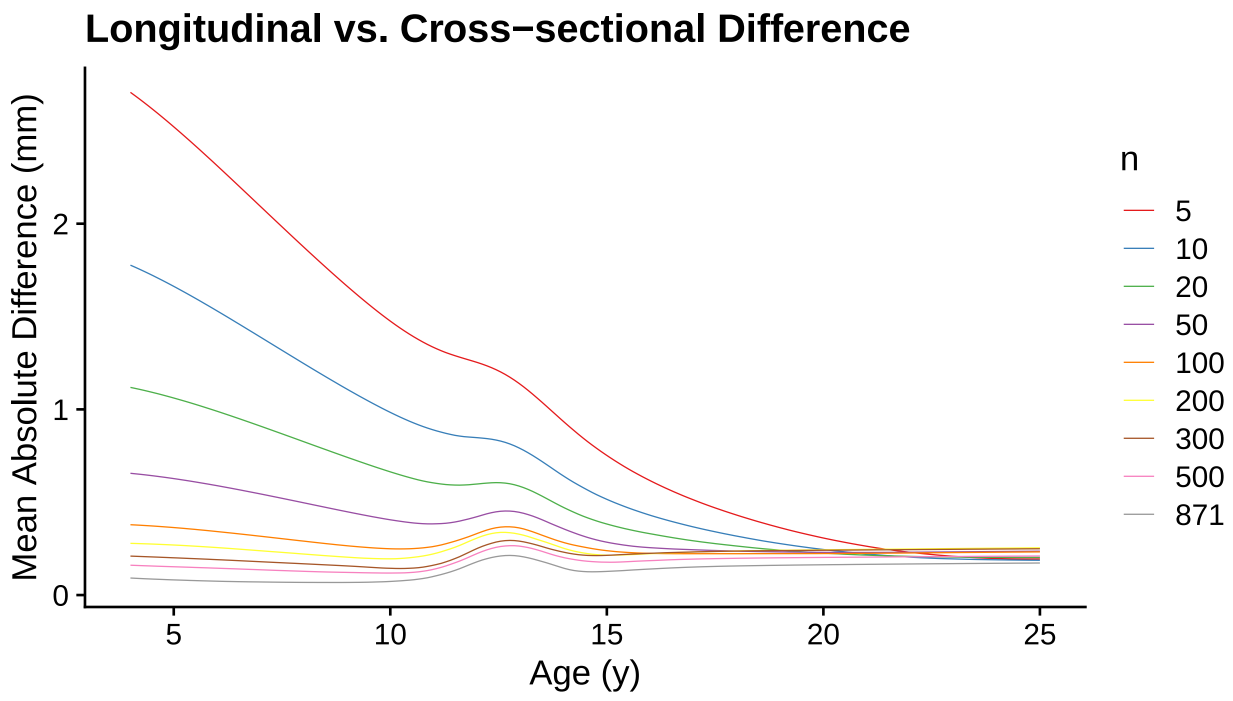
Longitudinal vs. Cross−sectional Difference
Mean Absolute Difference (mm)
Age (y)
n
5
10
20
50
100
200
300
500
871
0
1
2
5
10
15
20
25

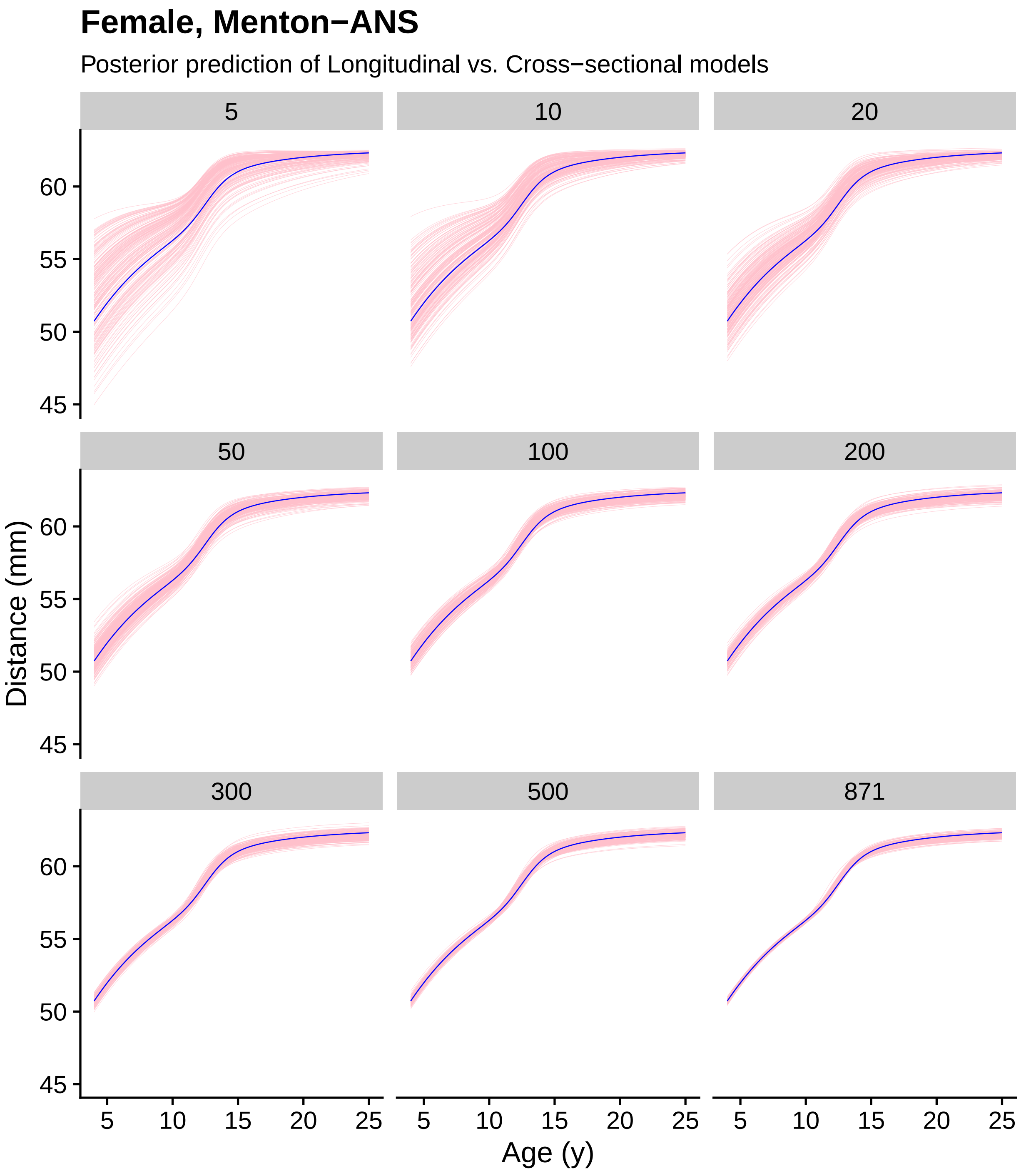

Female, Menton−ANS
Posterior prediction of Longitudinal vs. Cross−sectional models
5
10
20
50
100
200
300
500
871
Distance (mm)
45
50
55
60
Age (y)
5
10
15
20
25

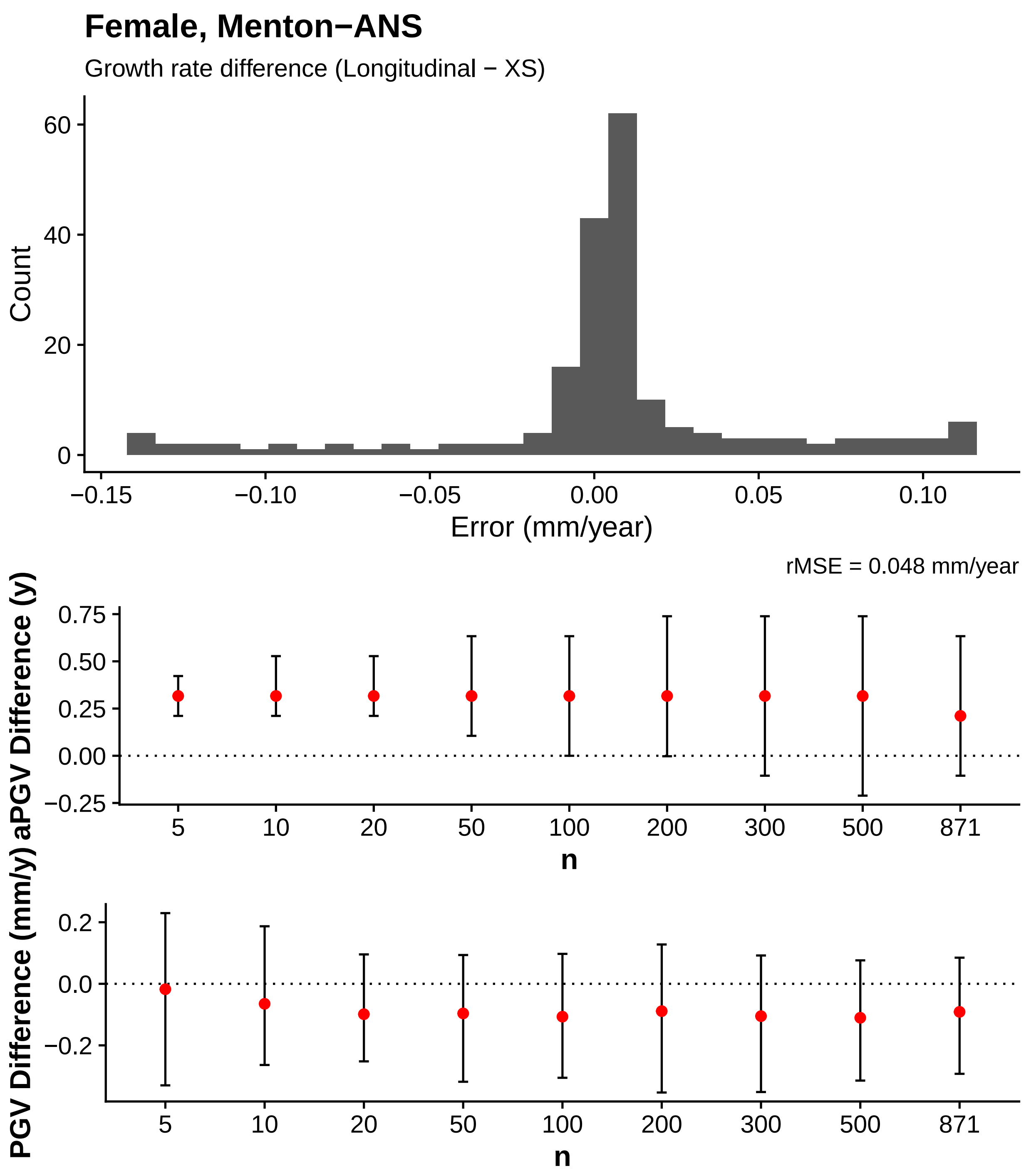
Female, Menton−ANS
Growth rate difference (Longitudinal − XS)
Count
60
40
20
0
−0.15
−0.10
−0.05
0.00
0.05
0.10
Error (mm/year)
rMSE = 0.048 mm/year
PGV Difference (mm/y) aPGV Difference (y)
0.75
0.50
0.25
0.00
−0.25
5
10
20
50
100
200
300
500
871
n
0.2
0.0
−0.2
5
10
20
50
100
200
300
500
871
n

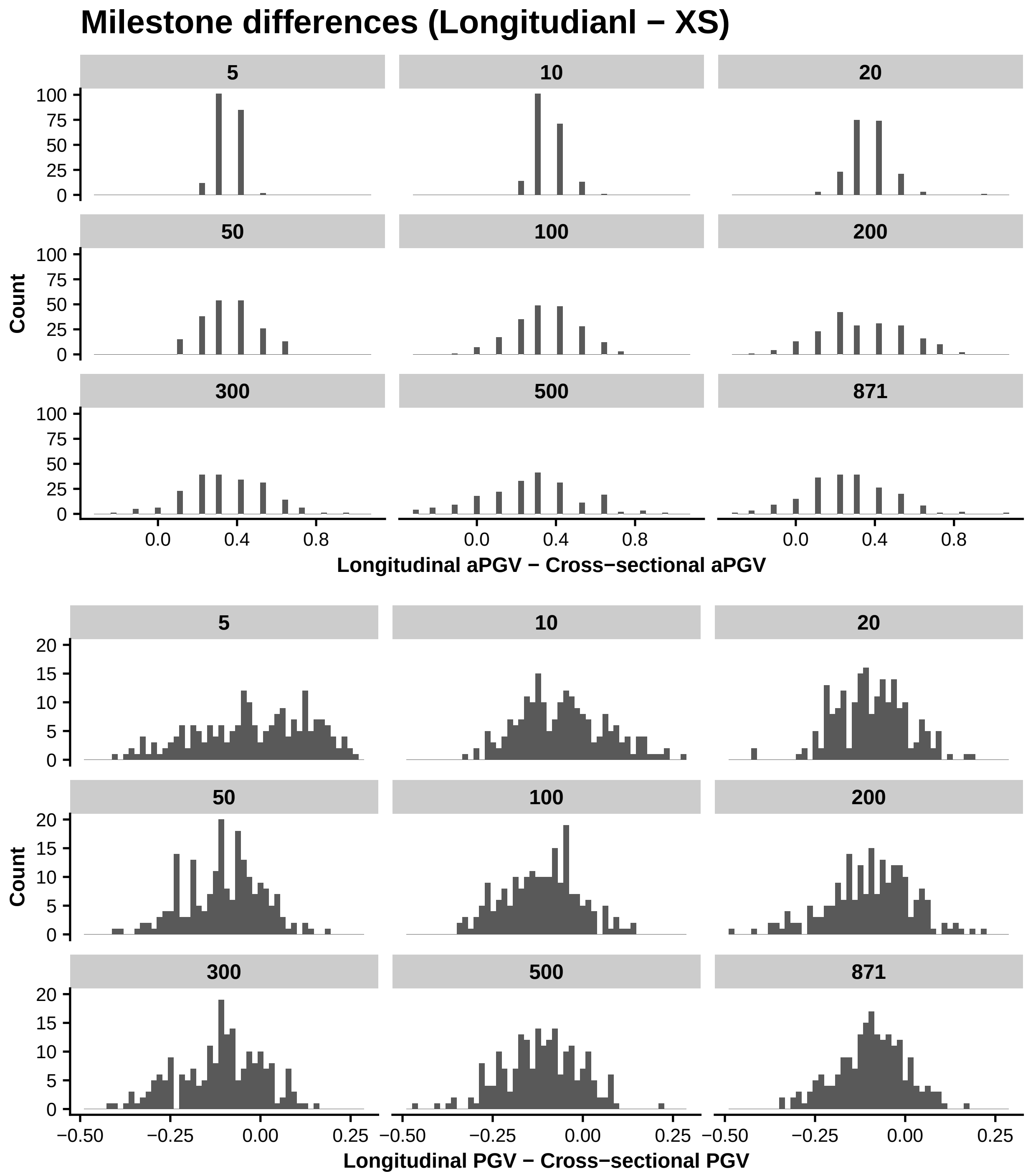
Milestone differences (Longitudianl − XS)
5
10
20
50
100
200
300
500
871
Count
Longitudinal aPGV − Cross−sectional aPGV
Count
Longitudinal PGV − Cross−sectional PGV

9 Female, Nasion-ANS

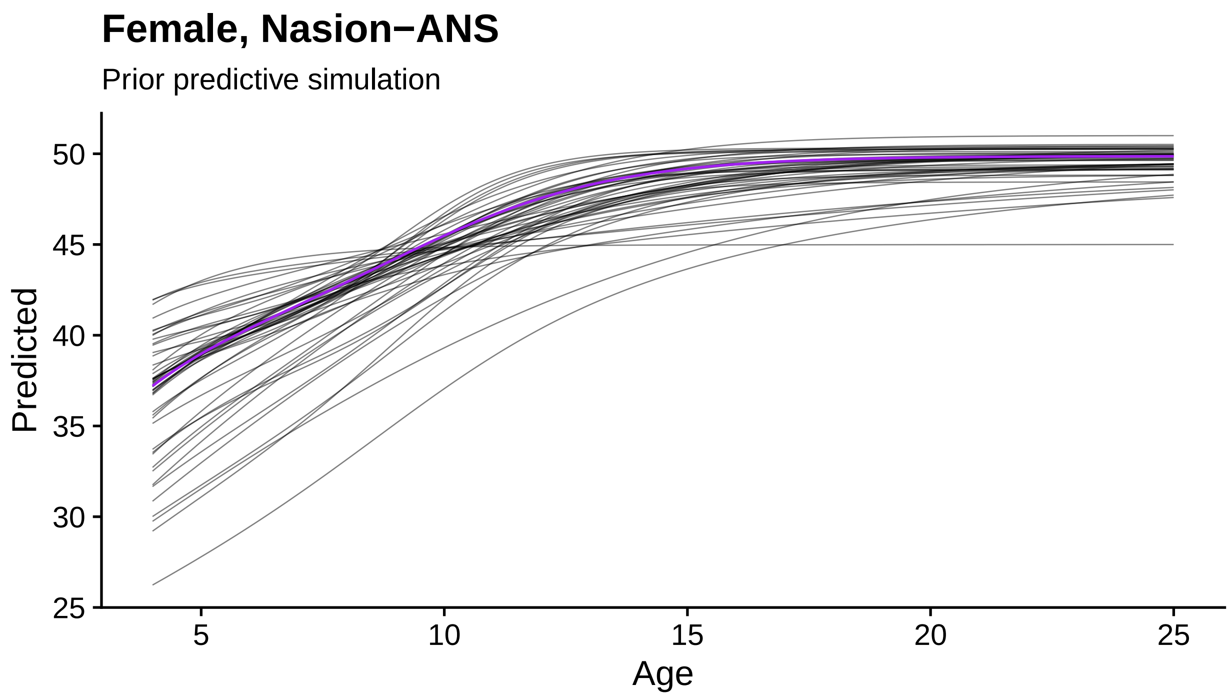

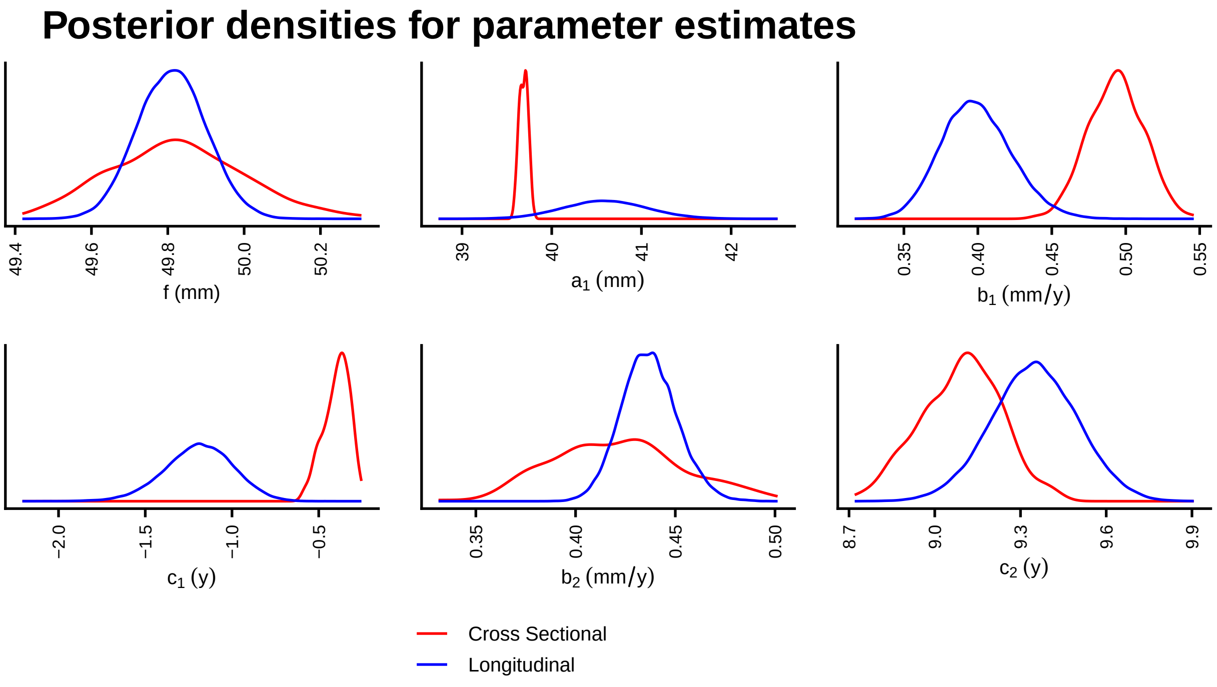

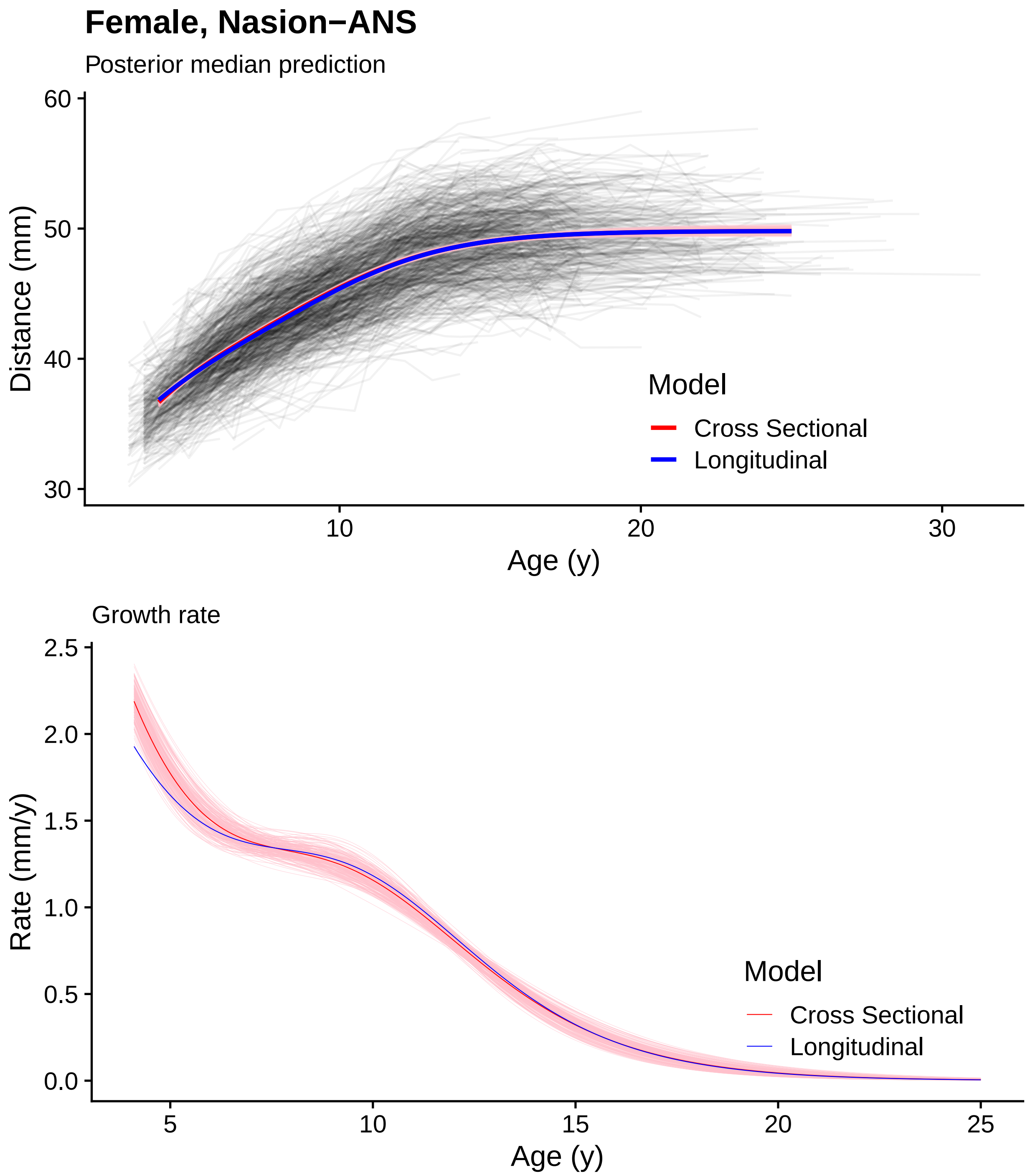
Female, Nasion−ANS
Posterior median prediction
Distance (mm)
60
50
40
30
10
20
30
Age (y)
Model
Cross Sectional
Longitudinal
Growth rate
Rate (mm/y)
2.5
2.0
1.5
1.0
0.5
0.0
5
10
15
20
25
Age (y)
Model
Cross Sectional
Longitudinal

Table 15: Longitudinal Model Summary

Parameter	Mean	Median	Std. Dev.	MAD	5%	95%	$\hat{r}$	Bulk ESS	Tail ESS
f	49.81	49.81	0.090	0.091	49.66	49.96	1.01	1285	3559
a1	40.60	40.59	0.467	0.466	39.83	41.37	1.00	18727	24106
b1	0.40	0.40	0.024	0.024	0.36	0.44	1.00	21292	27334
c1	-1.19	-1.19	0.198	0.195	-1.53	-0.88	1.00	25383	25992
b2	0.44	0.44	0.014	0.014	0.41	0.46	1.00	19352	26400
c2	9.35	9.35	0.151	0.151	9.10	9.60	1.00	17125	24215
sigma	1.12	1.12	0.009	0.009	1.11	1.14	1.00	49471	31223
sigma_ID	2.37	2.37	0.056	0.055	2.28	2.46	1.00	53588	29406

Table 16: Median Coefficients

Model	f	a_1	b_1	c_1	b_2	c_2	σ	σ_{ID}
Longitudinal	49.81	40.59	0.40	-1.19	0.44	9.35	1.12	2.37
Cross Sectional	49.82	39.69	0.49	-0.39	0.42	9.10	2.61	NA

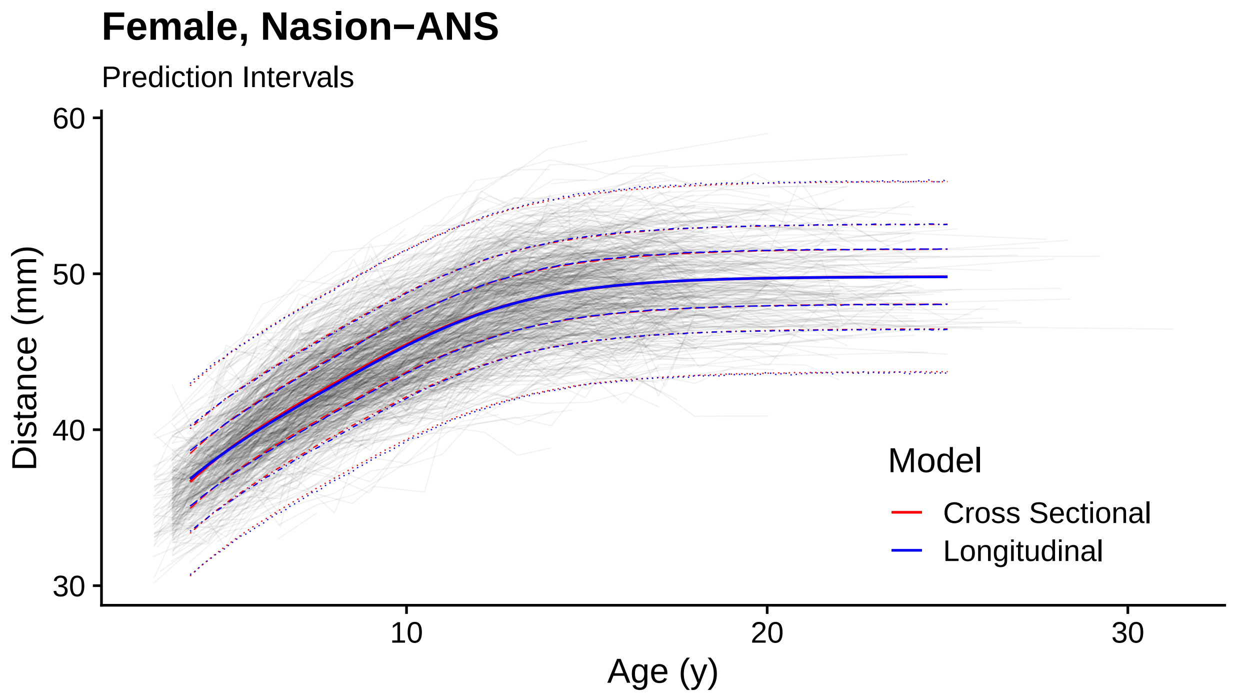
Female, Nasion−ANS
Prediction Intervals
Distance (mm)
60
50
40
30
10
20
30
Age (y)
Model
Cross Sectional
Longitudinal

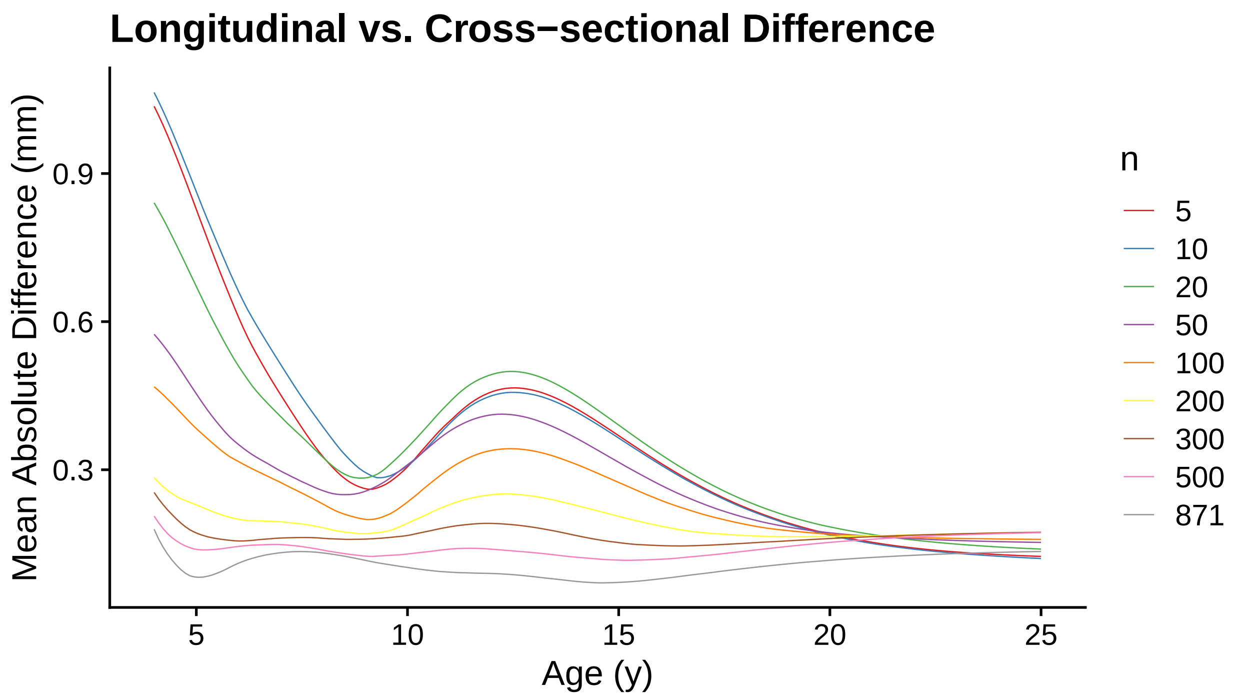
Longitudinal vs. Cross−sectional Difference
Mean Absolute Difference (mm)
0.9
0.6
0.3
5
10
15
20
25
Age (y)
n
5
10
20
50
100
200
300
500
871

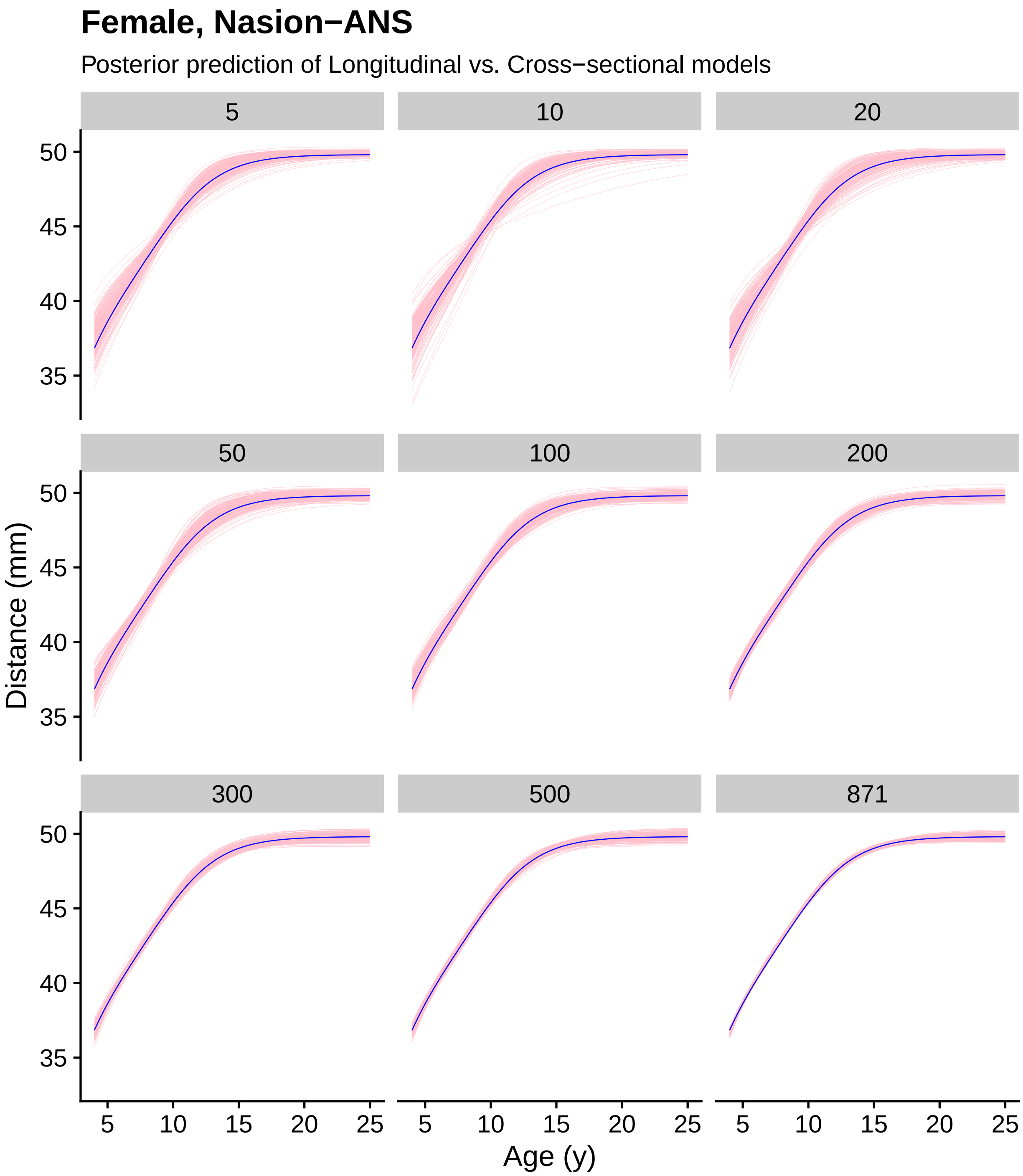
Female, Nasion−ANS
Posterior prediction of Longitudinal vs. Cross−sectional models
5
10
20
50
100
200
300
500
871
50
45
40
35
Distance (mm)
5
10
15
20
25
Age (y)

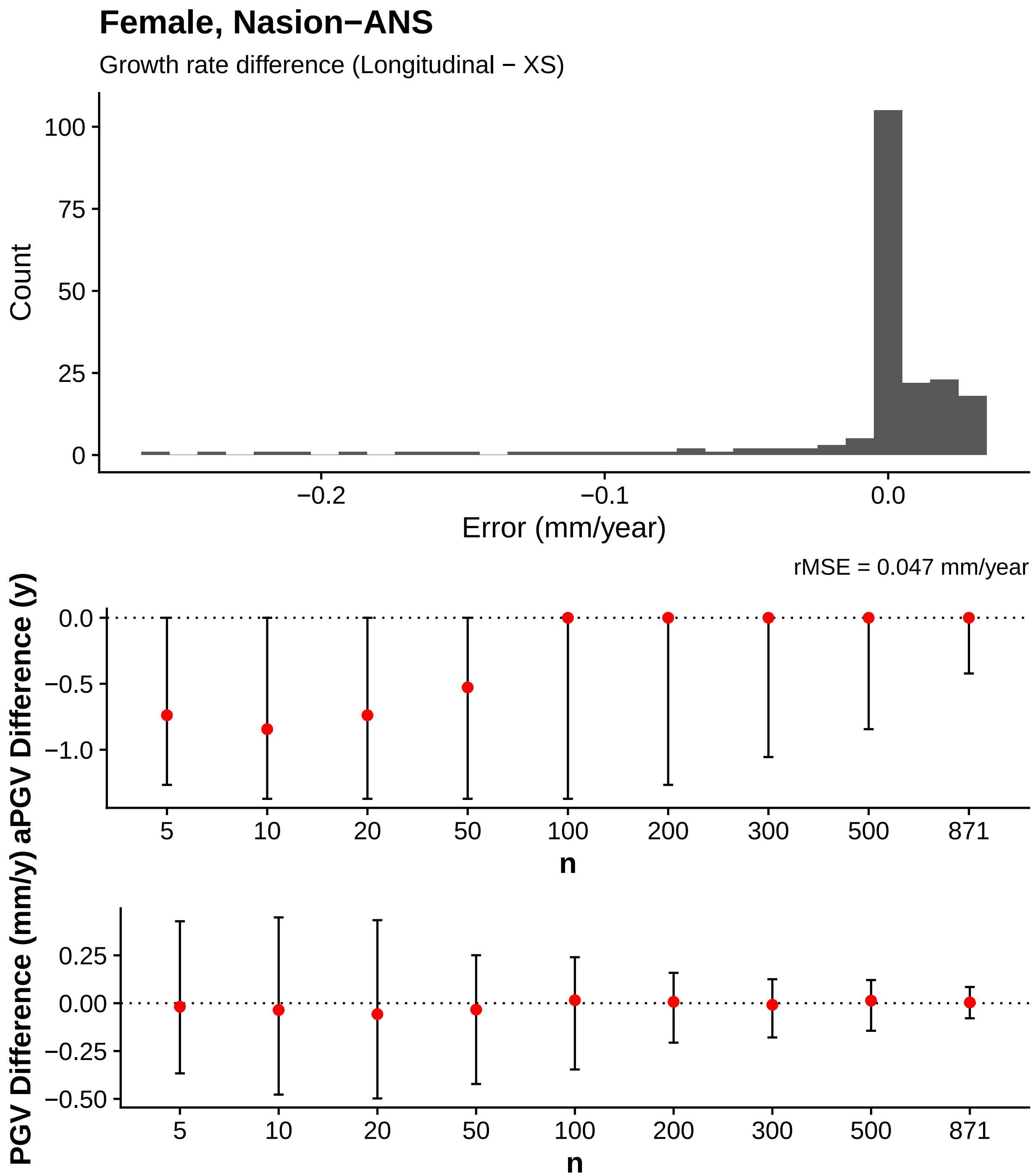

Female, Nasion−ANS
Growth rate difference (Longitudinal − XS)
Count
100
75
50
25
0
−0.2
−0.1
0.0
Error (mm/year)
rMSE = 0.047 mm/year
aPGV Difference (y)
0.0
−0.5
−1.0
5
10
20
50
100
200
300
500
871
n
PGV Difference (mm/y)
0.25
0.00
−0.25
−0.50
5
10
20
50
100
200
300
500
871
n

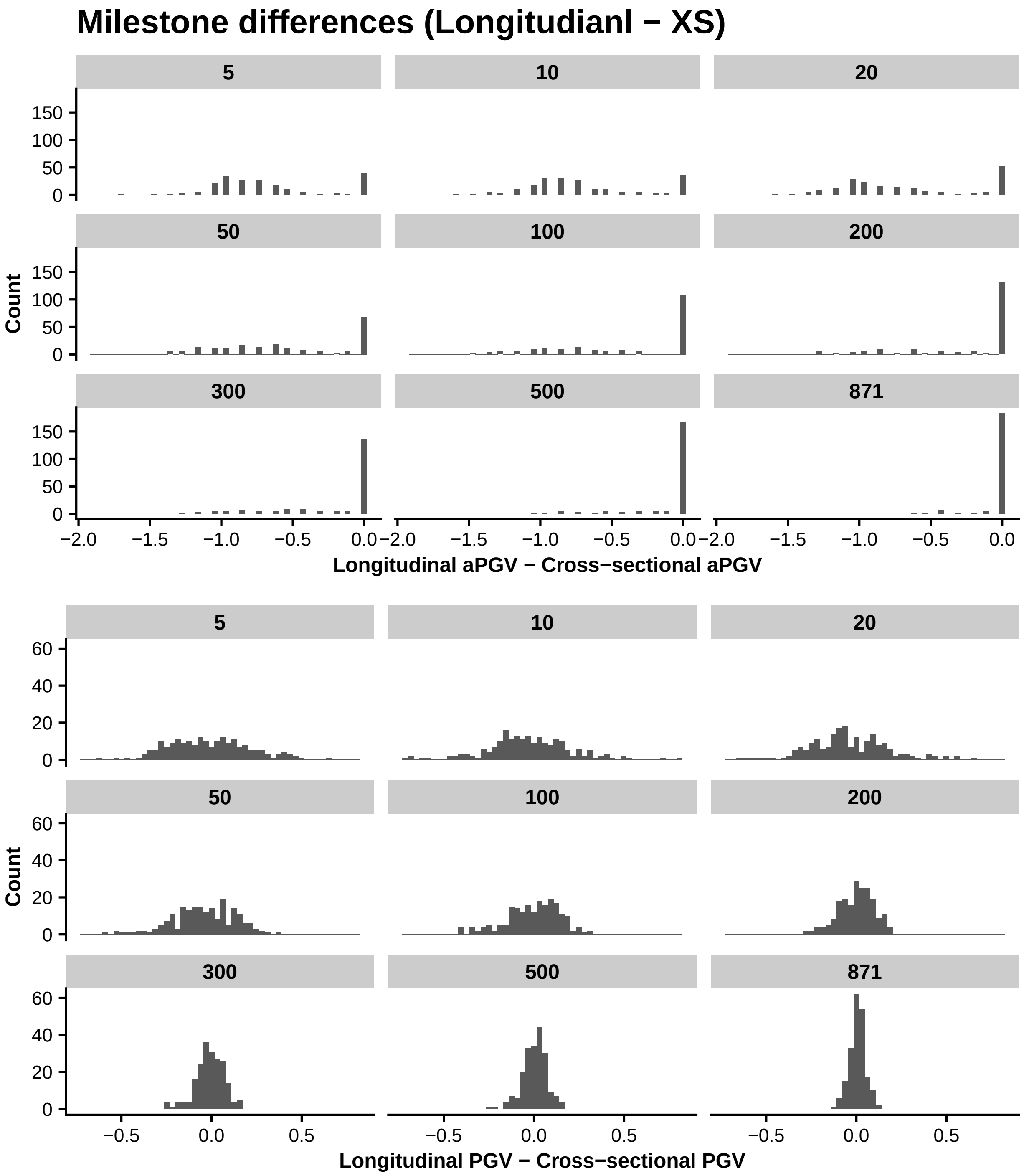
Milestone differences (Longitudianl − XS)
5
10
20
50
100
200
300
500
871
Count
150
100
50
0
−2.0
−1.5
−1.0
−0.5
0.0
Longitudinal aPGV − Cross−sectional aPGV
60
40
20
−0.5
0.5
Longitudinal PGV − Cross−sectional PGV

10 Female, Nasion-Basion

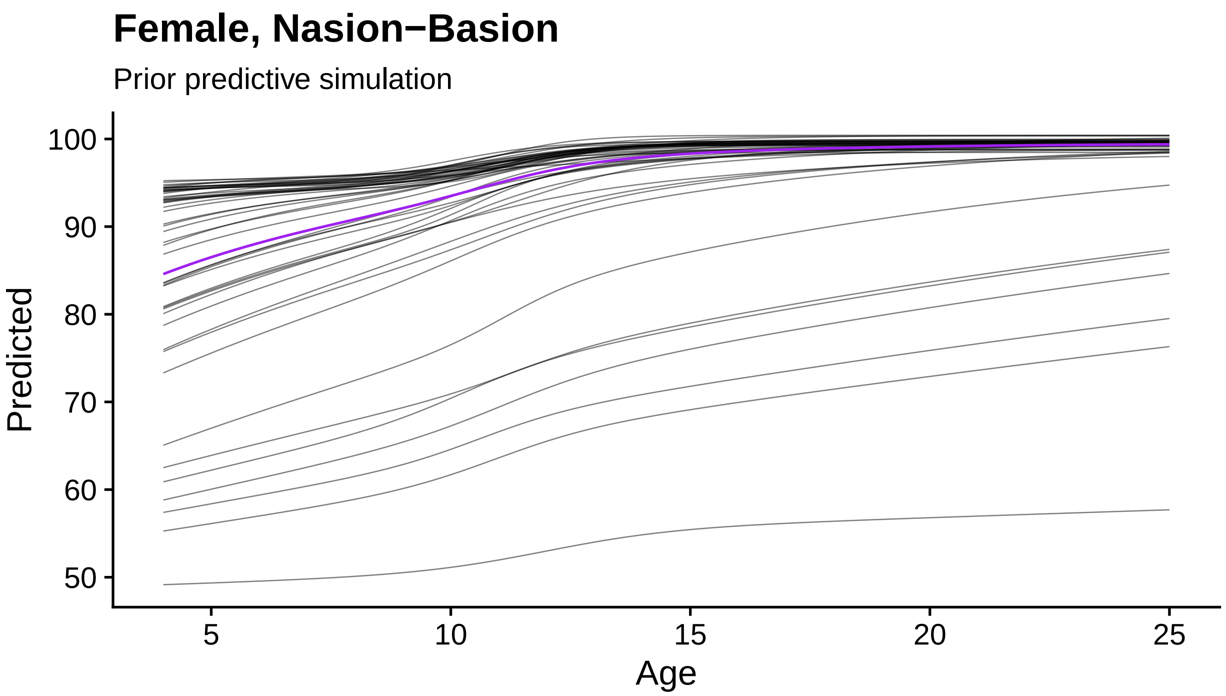

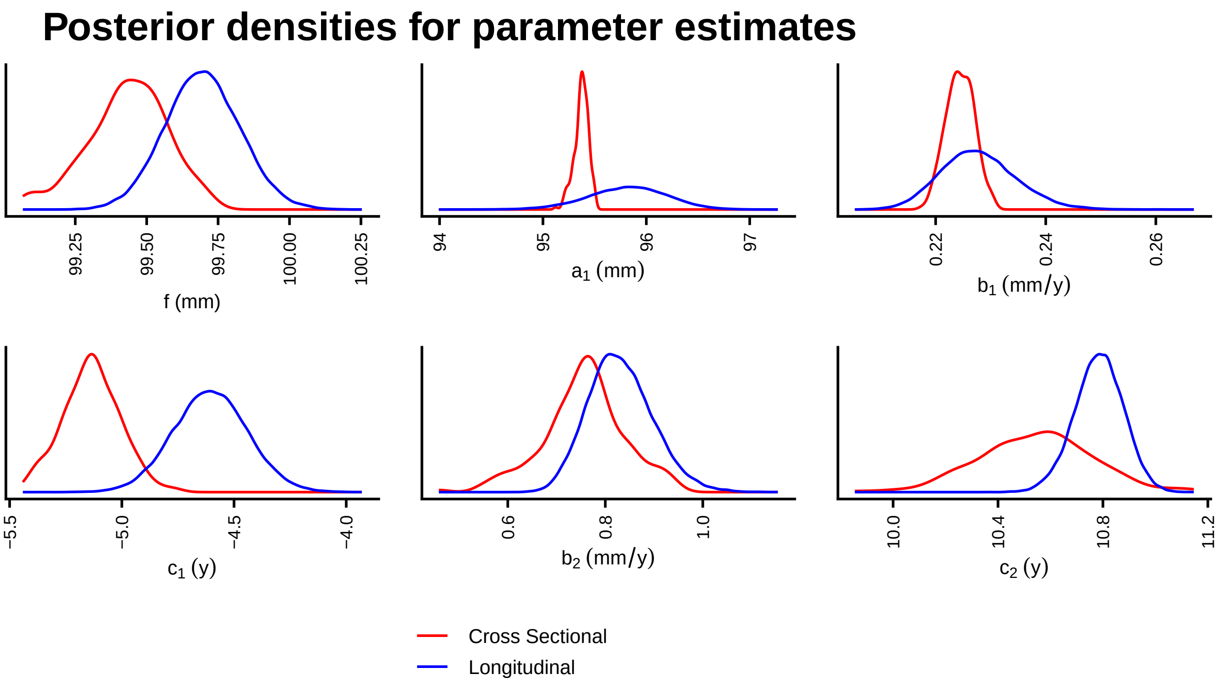

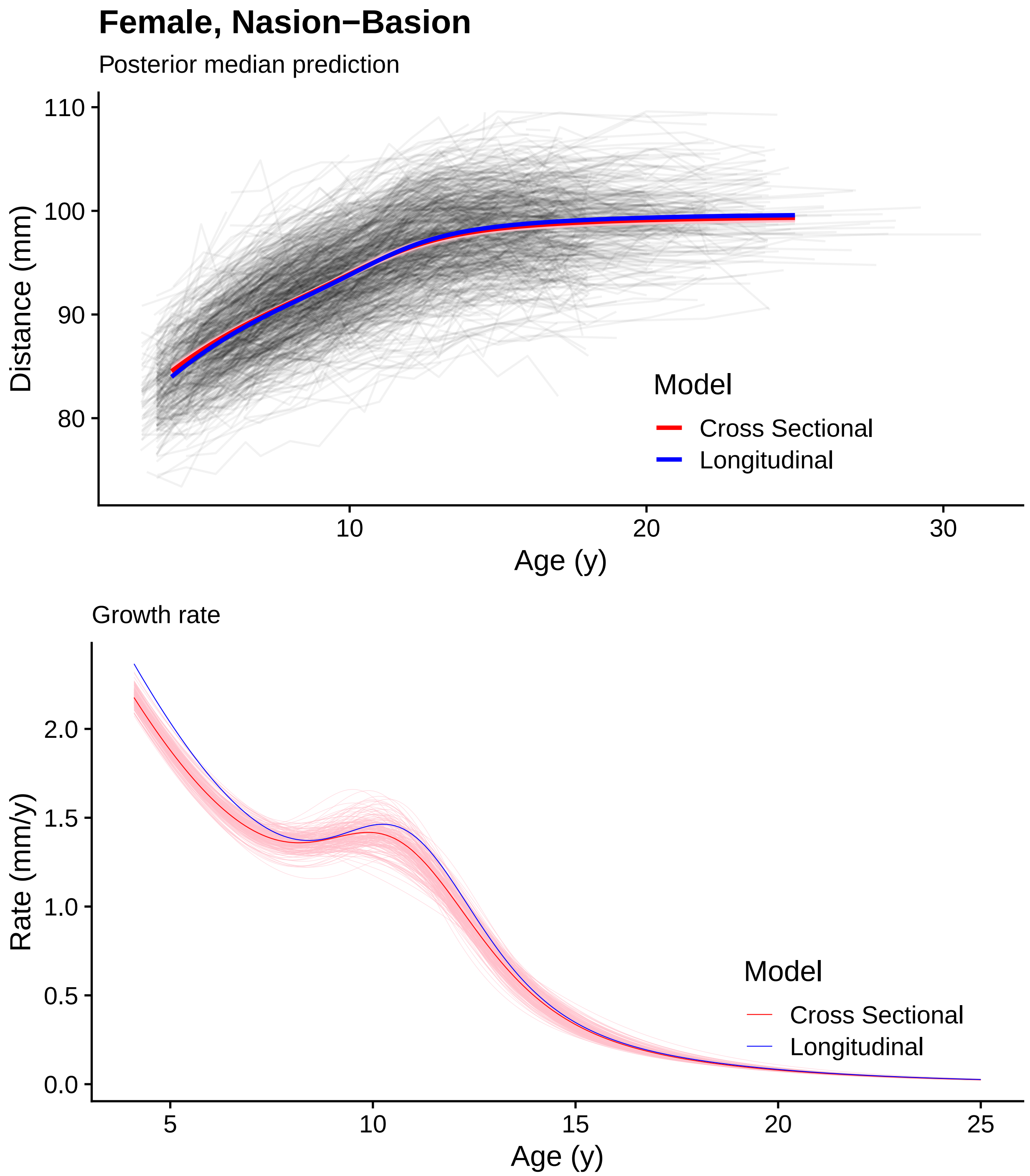
Female, Nasion−Basion
Posterior median prediction
Distance (mm)
Age (y)
Model
Cross Sectional
Longitudinal
Growth rate
Rate (mm/y)
Age (y)
Model
Cross Sectional
Longitudinal

Table 17: Longitudinal Model Summary

Parameter	Mean	Median	Std. Dev.	MAD	5%	95%	$\hat{r}$	Bulk ESS	Tail ESS
f	99.70	99.69	0.132	0.131	99.48	99.91	1	1390	3855
a1	95.82	95.83	0.380	0.378	95.17	96.42	1	17127	22913
b1	0.23	0.23	0.007	0.007	0.22	0.24	1	14049	20349
c1	-4.61	-4.60	0.166	0.164	-4.88	-4.33	1	21457	25617
b2	0.83	0.83	0.067	0.066	0.73	0.95	1	18596	28125
c2	10.78	10.79	0.093	0.093	10.63	10.93	1	31628	29372
sigma	1.45	1.45	0.012	0.012	1.43	1.47	1	53626	31817
sigma_ID	3.68	3.68	0.086	0.086	3.55	3.83	1	65398	30043

Table 18: Median Coefficients

Model	f	a_1	b_1	c_1	b_2	c_2	σ	σ_{ID}
Longitudinal	99.69	95.83	0.23	-4.60	0.83	10.79	1.45	3.68
Cross Sectional	99.44	95.38	0.22	-5.13	0.76	10.56	3.94	NA

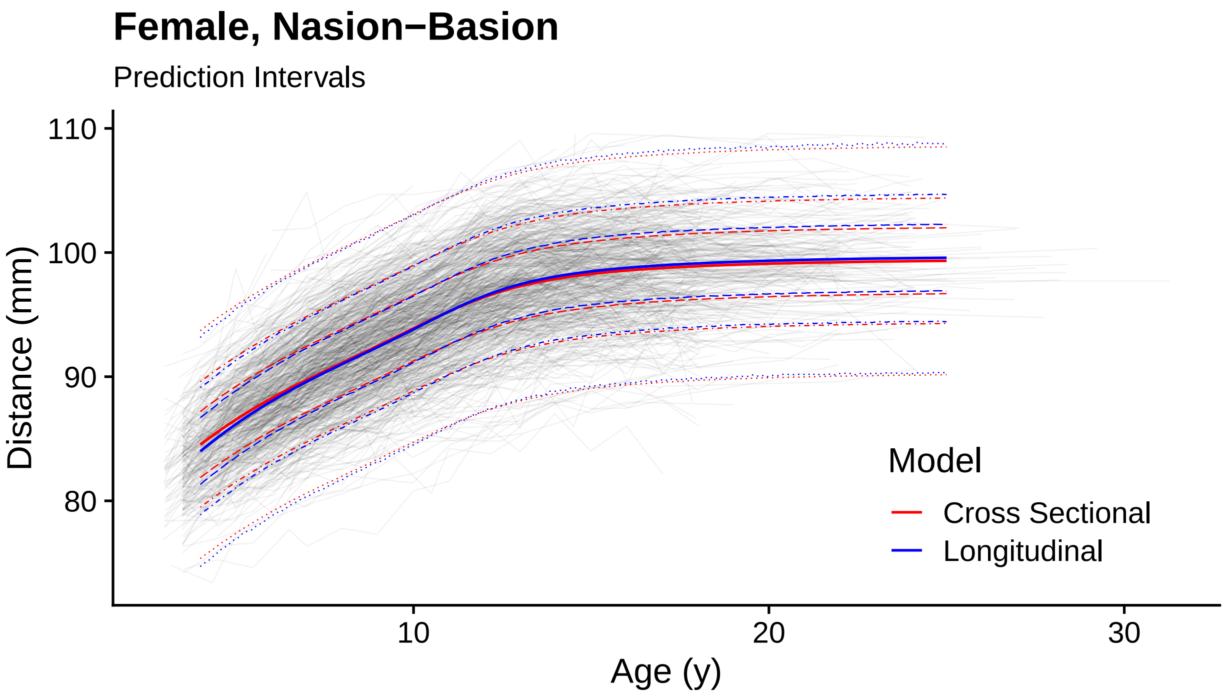
Female, Nasion−Basion
Prediction Intervals
Distance (mm)
110
100
90
80
10
20
30
Age (y)
Model
Cross Sectional
Longitudinal

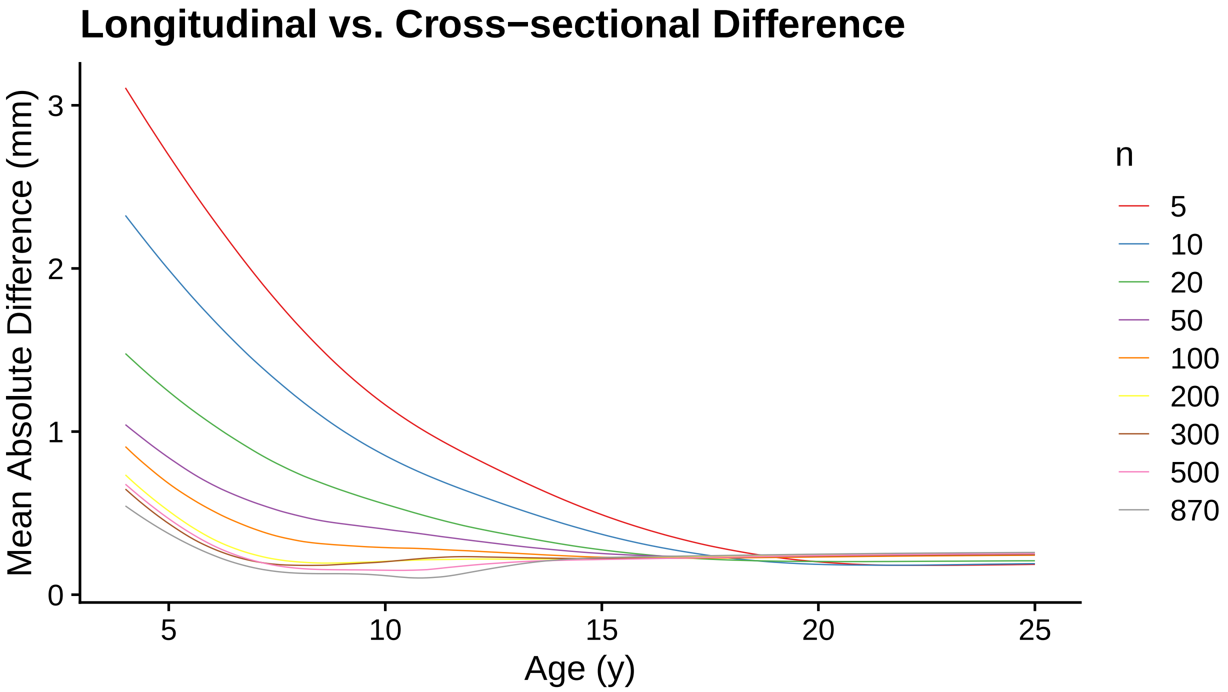
Longitudinal vs. Cross−sectional Difference
Mean Absolute Difference (mm)
3
2
1
0
5
10
15
20
25
Age (y)
n
5
10
20
50
100
200
300
500
870

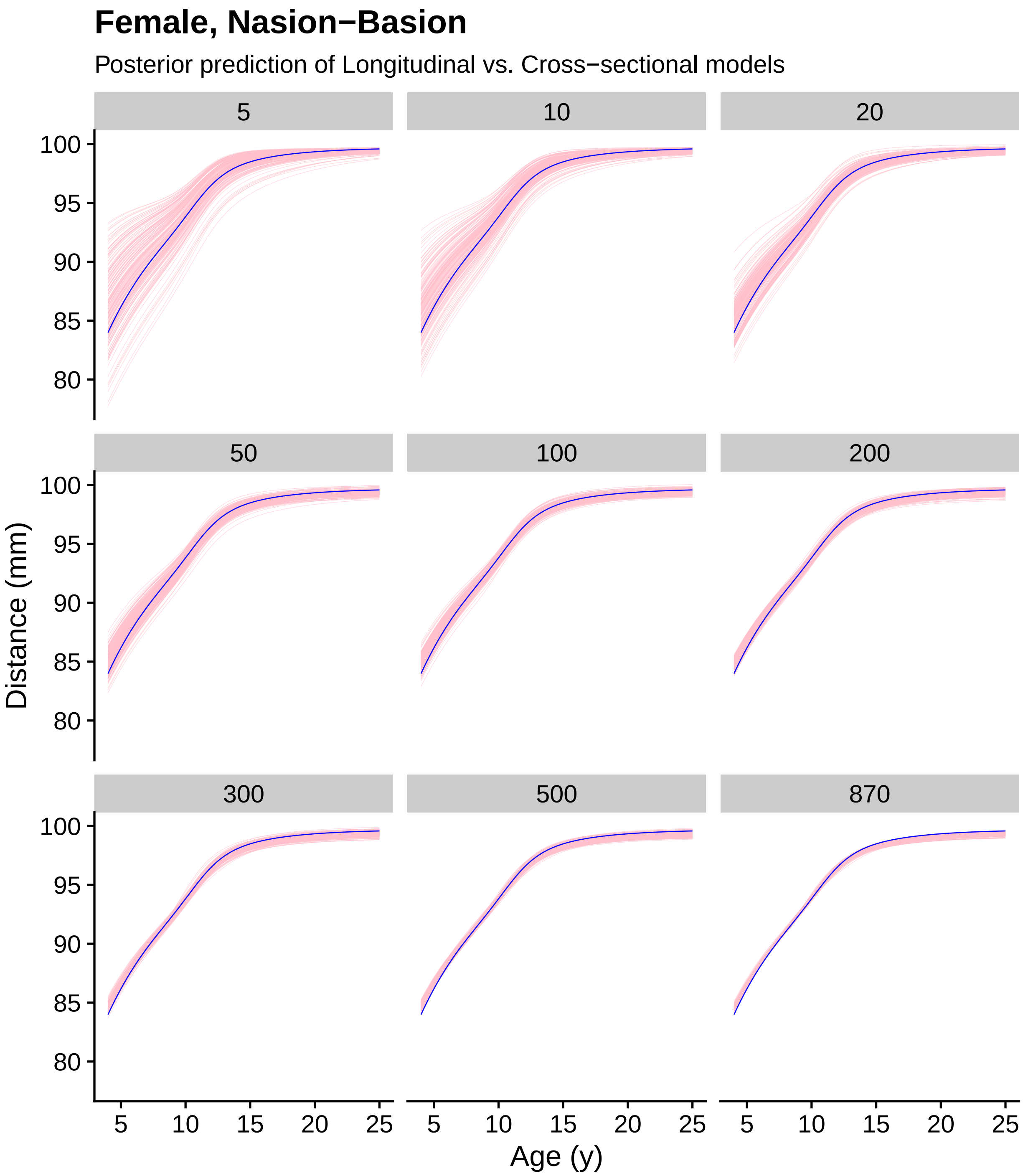

Female, Nasion−Basion
Posterior prediction of Longitudinal vs. Cross−sectional models
5
10
20
50
100
200
300
500
870
Distance (mm)
Age (y)
100
95
90
85
80
5
10
15
20
25

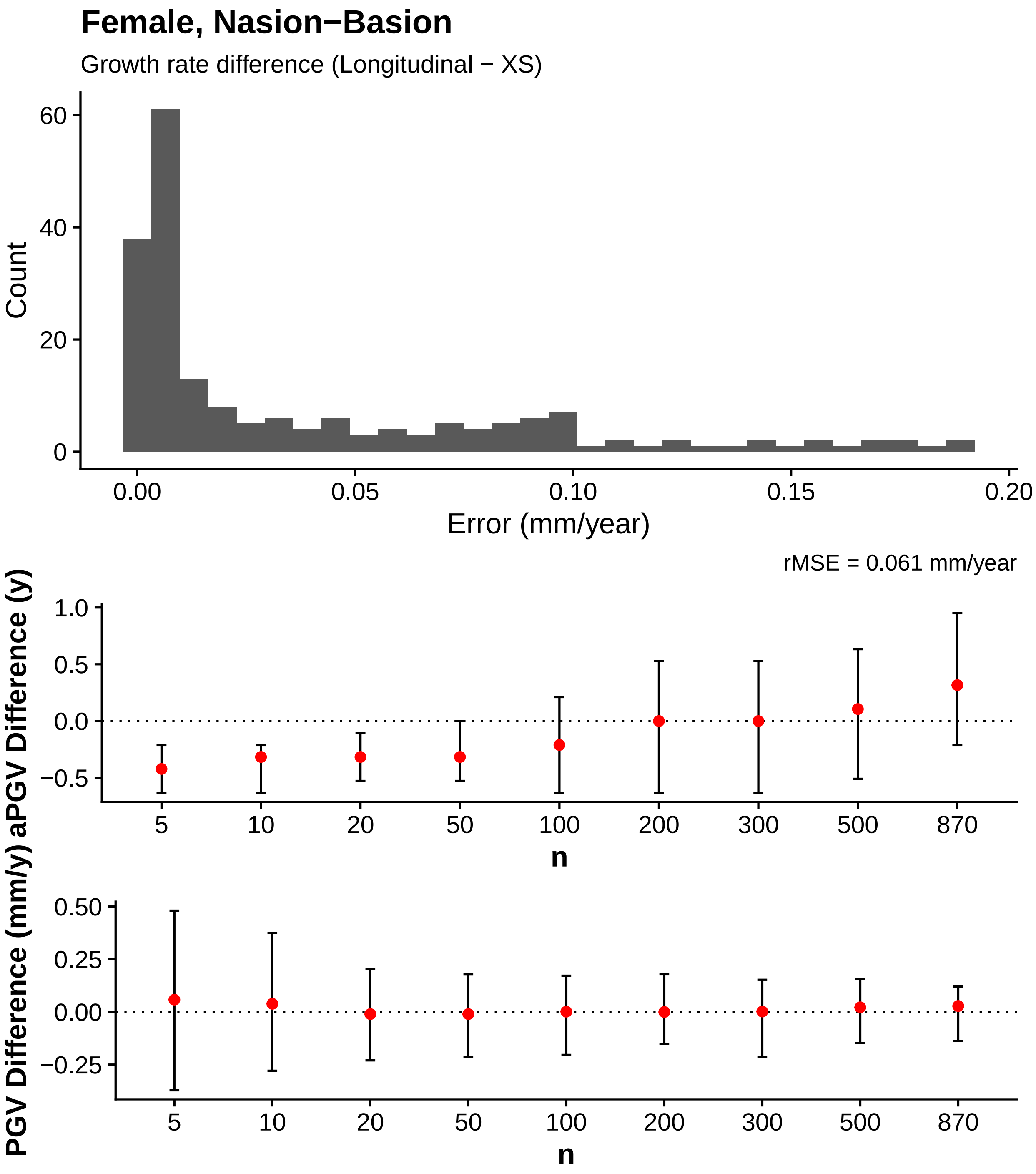
Female, Nasion−Basion
Growth rate difference (Longitudinal − XS)
Count
60
40
20
0
0.00
0.05
0.10
0.15
0.20
Error (mm/year)
rMSE = 0.061 mm/year
aPGV Difference (y)
1.0
0.5
0.0
−0.5
5
10
20
50
100
200
300
500
870
n
PGV Difference (mm/y)
0.50
0.25
0.00
−0.25
5
10
20
50
100
200
300
500
870
n

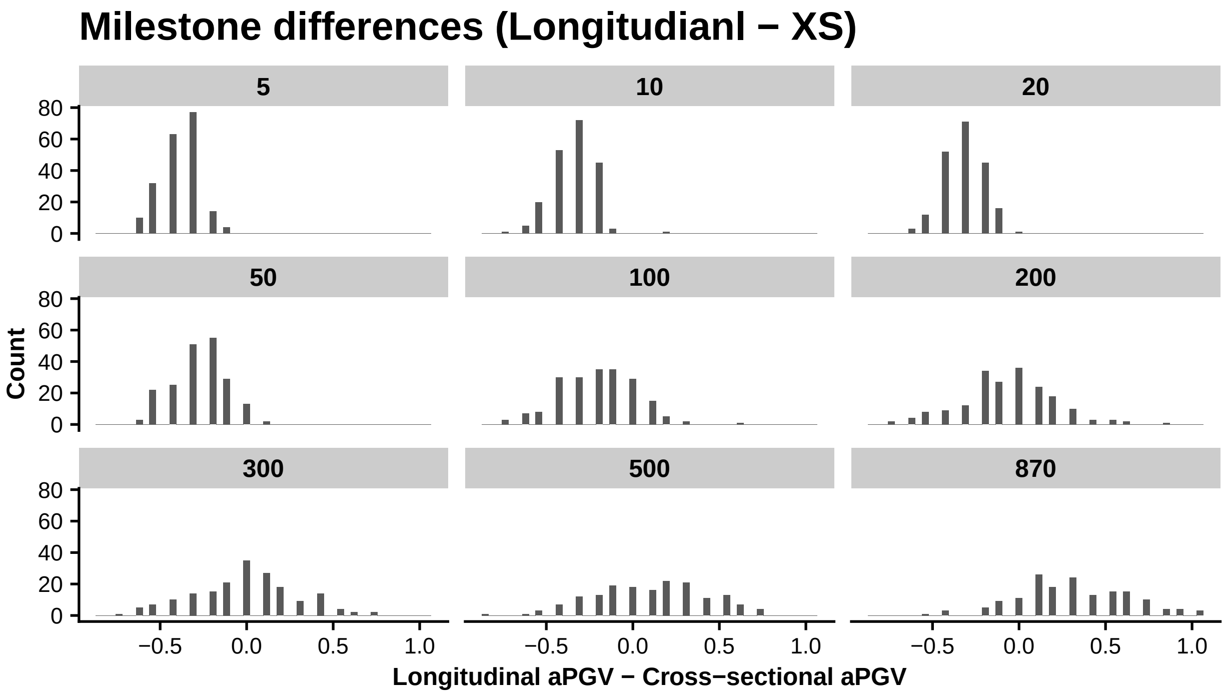

Milestone differences (Longitudianl − XS)
5
10
20
50
100
200
300
500
870
Count
80
60
40
20
0
−0.5
0.0
0.5
1.0
Longitudinal aPGV − Cross−sectional aPGV

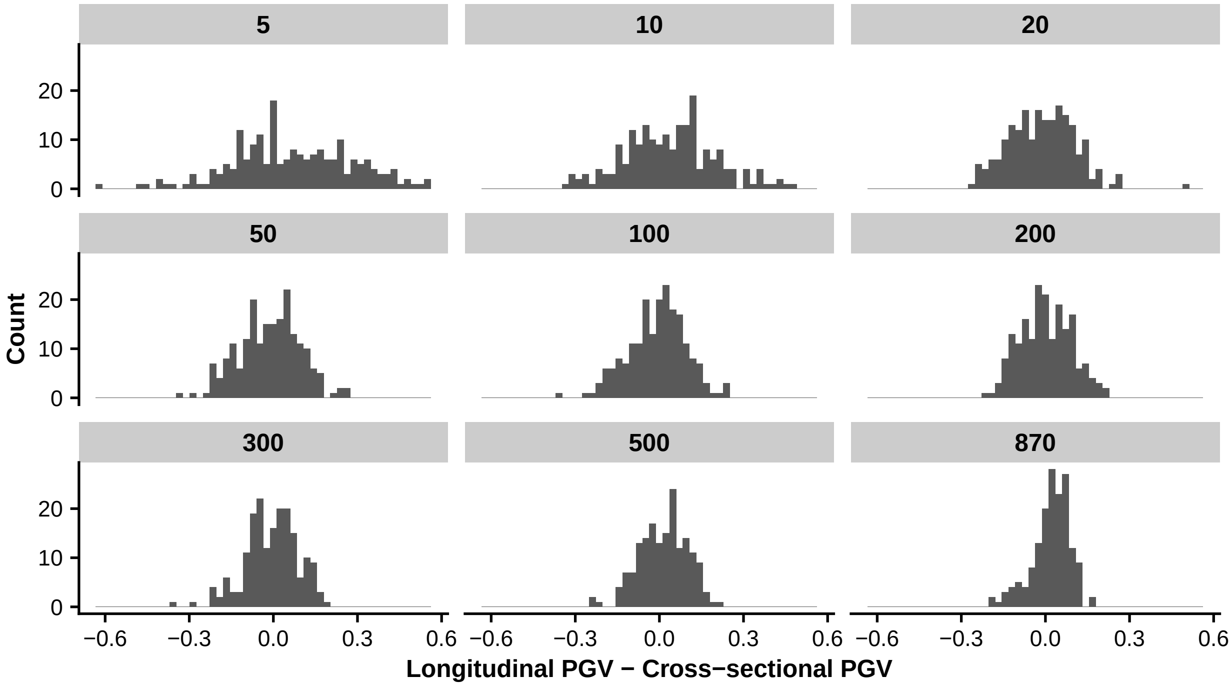

5
10
20
50
100
200
300
500
870
Count
20
10
0
−0.6
−0.3
0.0
0.3
0.6
Longitudinal PGV − Cross−sectional PGV

11 Female, Nasion-Menton

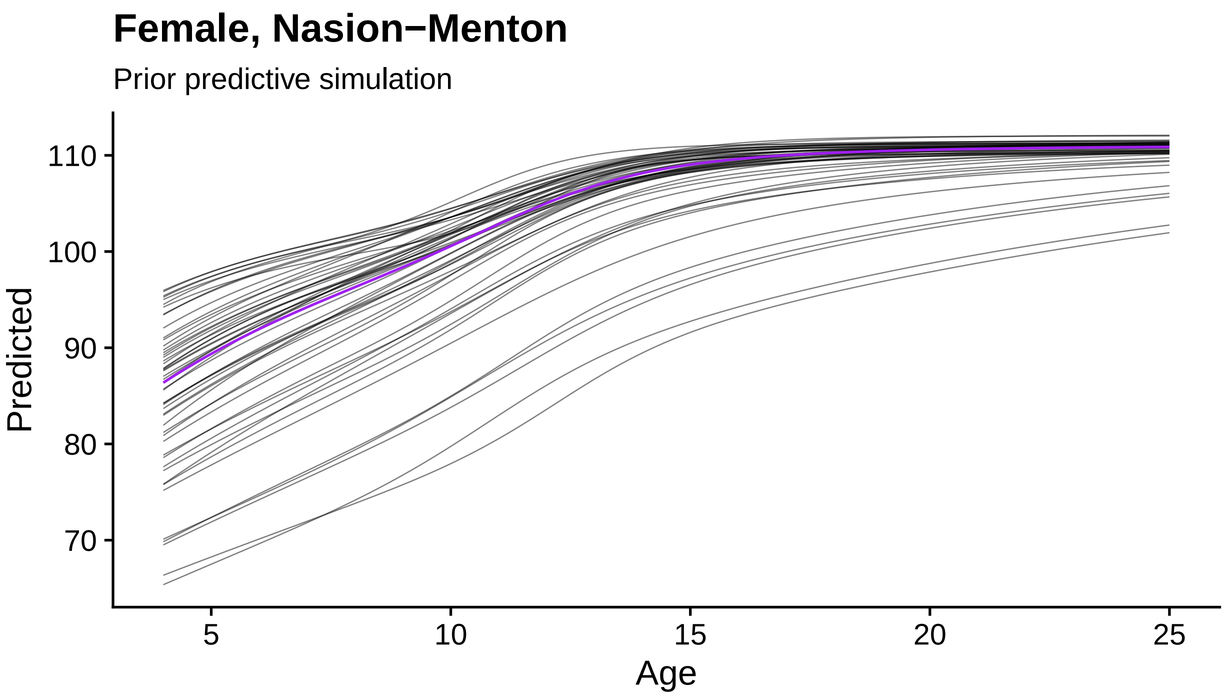

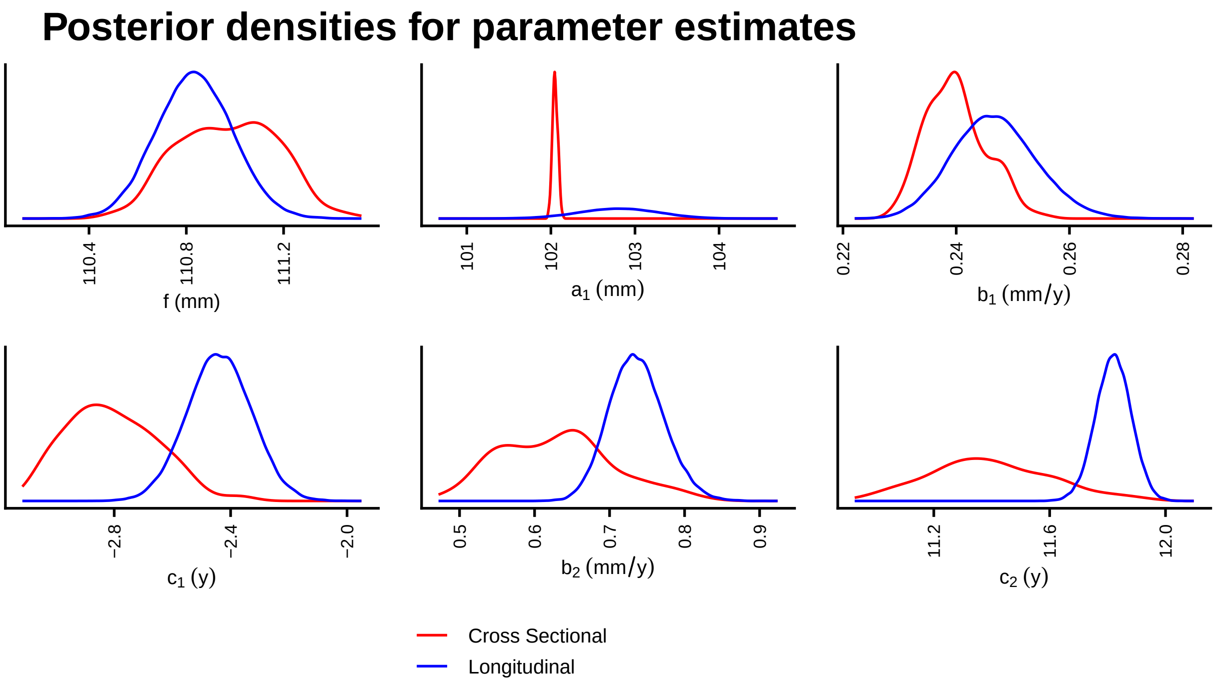

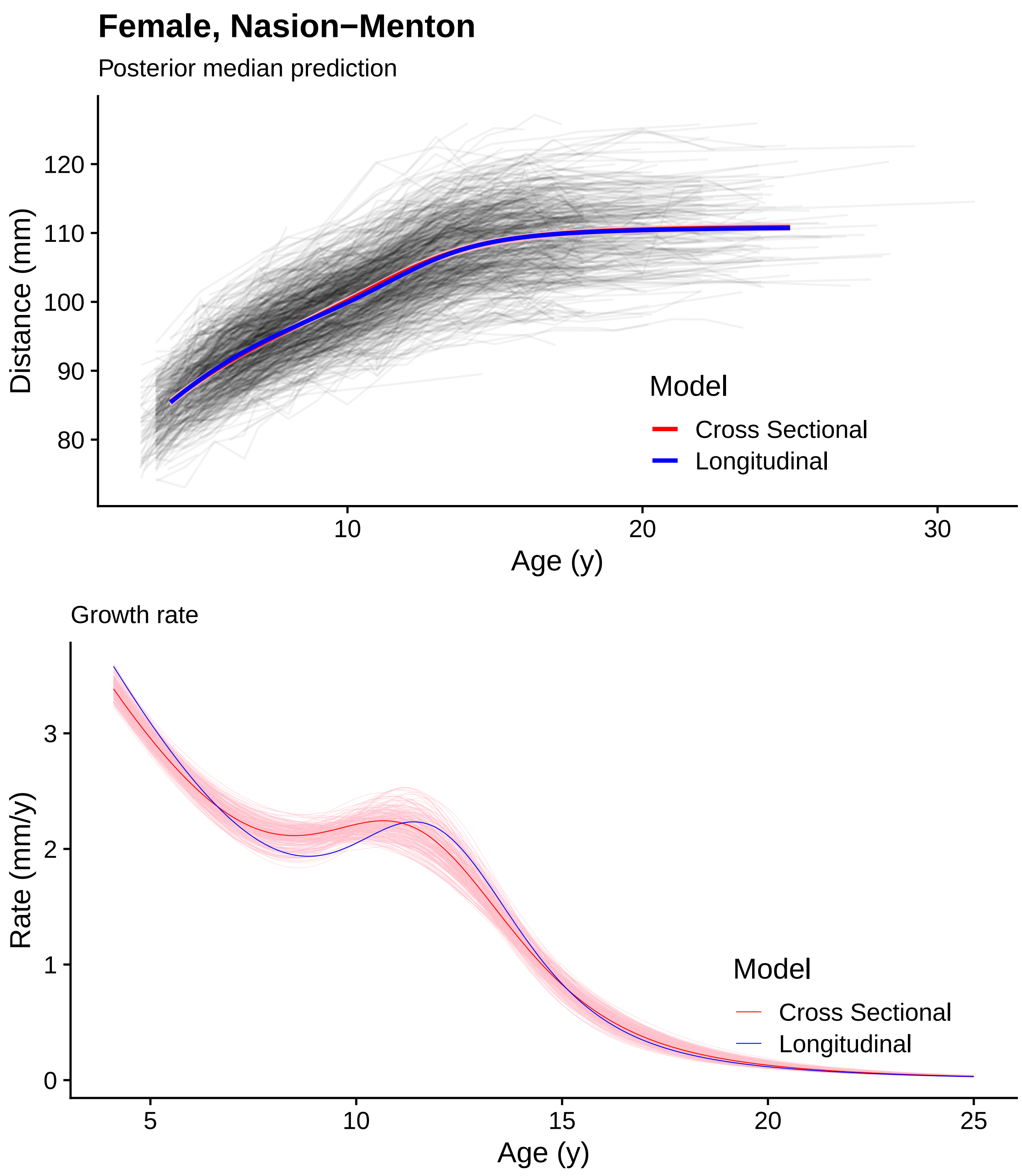
Female, Nasion−Menton
Posterior median prediction
Distance (mm)
120
110
100
90
80
10
20
30
Age (y)
Model
Cross Sectional
Longitudinal
Growth rate
Rate (mm/y)
3
2
1
0
5
10
15
20
25
Age (y)
Model
Cross Sectional
Longitudinal

Table 19: Longitudinal Model Summary

Parameter	Mean	Median	Std. Dev.	MAD	5%	95%	$\hat{r}$	Bulk ESS	Tail ESS
f	110.83	110.83	0.157	0.157	110.58	111.09	1	2114	5545
a1	102.80	102.81	0.474	0.474	102.02	103.57	1	24428	28430
b1	0.25	0.25	0.007	0.007	0.24	0.26	1	12498	23923
c1	-2.44	-2.44	0.110	0.110	-2.62	-2.26	1	27760	29070
b2	0.74	0.73	0.037	0.037	0.68	0.80	1	19657	28614
c2	11.82	11.82	0.061	0.061	11.72	11.92	1	36370	32217
sigma	1.97	1.97	0.016	0.016	1.94	1.99	1	63544	30834
sigma_ID	4.56	4.56	0.107	0.107	4.39	4.74	1	74290	30302

Table 20: Median Coefficients

Model	f	a_1	b_1	c_1	b_2	c_2	σ	σ_{ID}
Longitudinal	110.83	102.81	0.25	-2.44	0.73	11.82	1.97	4.56
Cross Sectional	110.97	102.05	0.24	-2.82	0.63	11.38	4.92	NA

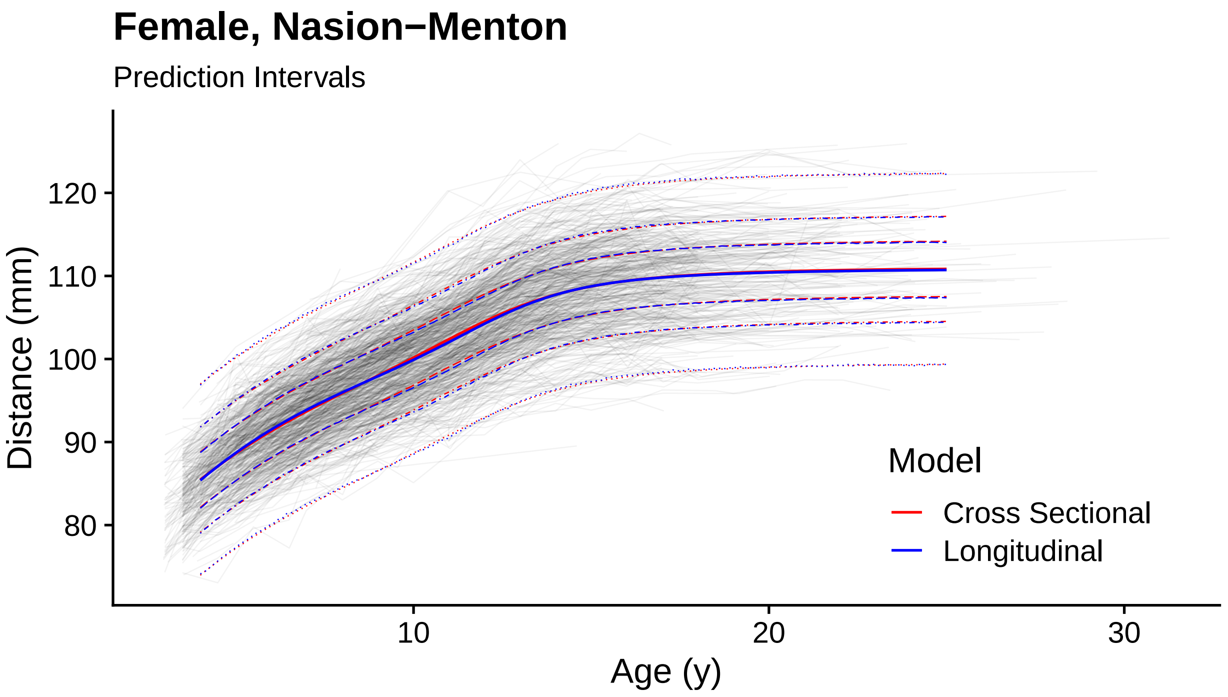
Female, Nasion−Menton
Prediction Intervals
Distance (mm)
120
110
100
90
80
10
20
30
Age (y)
Model
Cross Sectional
Longitudinal

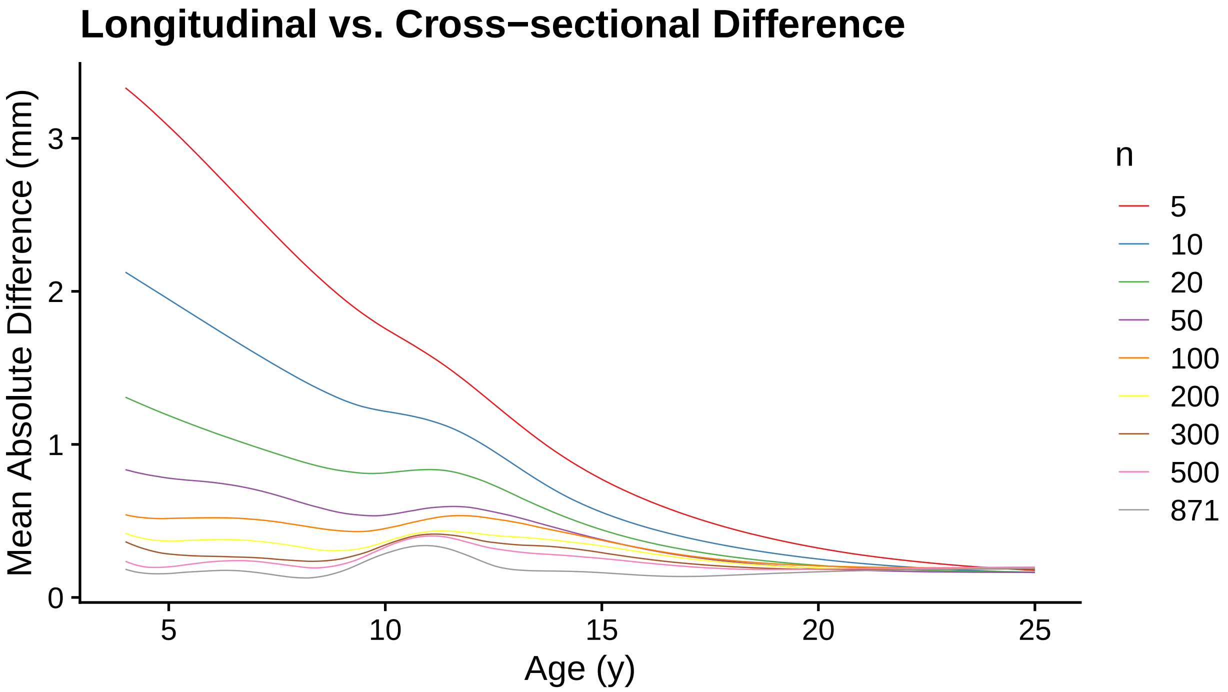
Longitudinal vs. Cross−sectional Difference
Mean Absolute Difference (mm)
3
2
1
0
5
10
15
20
25
Age (y)
n
5
10
20
50
100
200
300
500
871

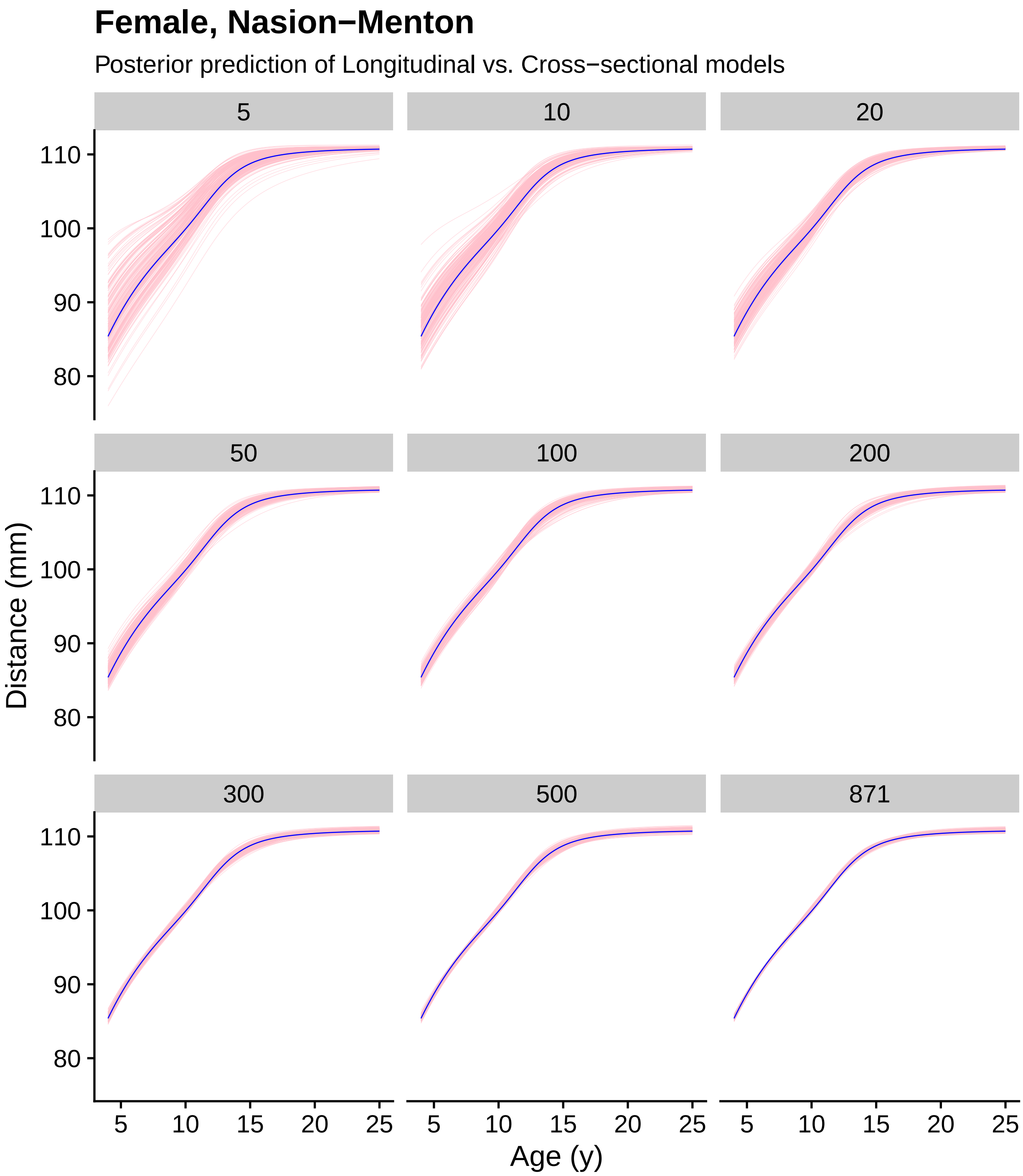

Female, Nasion−Menton
Posterior prediction of Longitudinal vs. Cross−sectional models
5
10
20
50
100
200
300
500
871
Distance (mm)
110
100
90
80
5
10
15
20
25
Age (y)

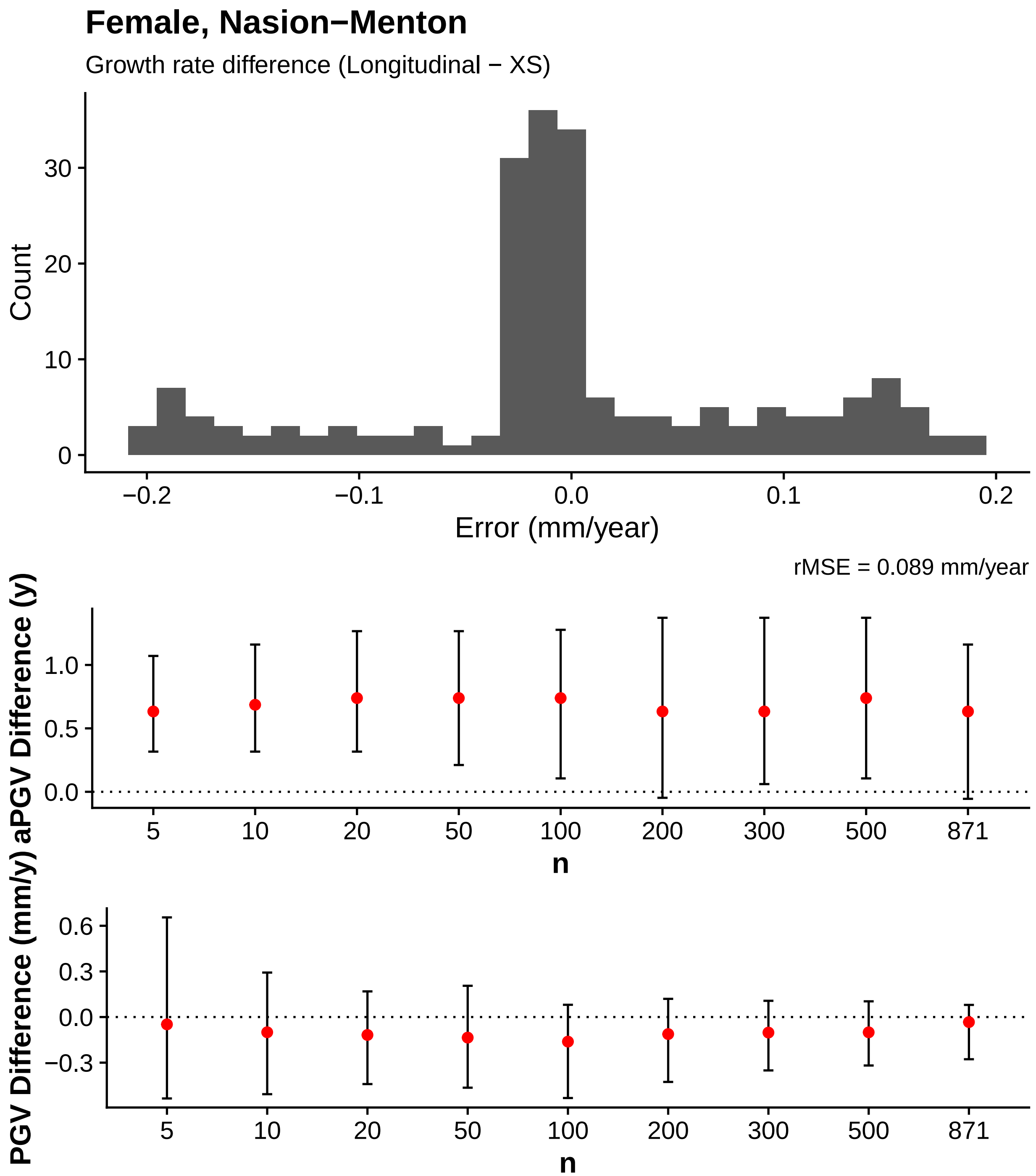
Female, Nasion−Menton
Growth rate difference (Longitudinal − XS)
Count
Error (mm/year)
rMSE = 0.089 mm/year
aPGV Difference (y)
PGV Difference (mm/y)
n
5
10
20
50
100
200
300
500
871

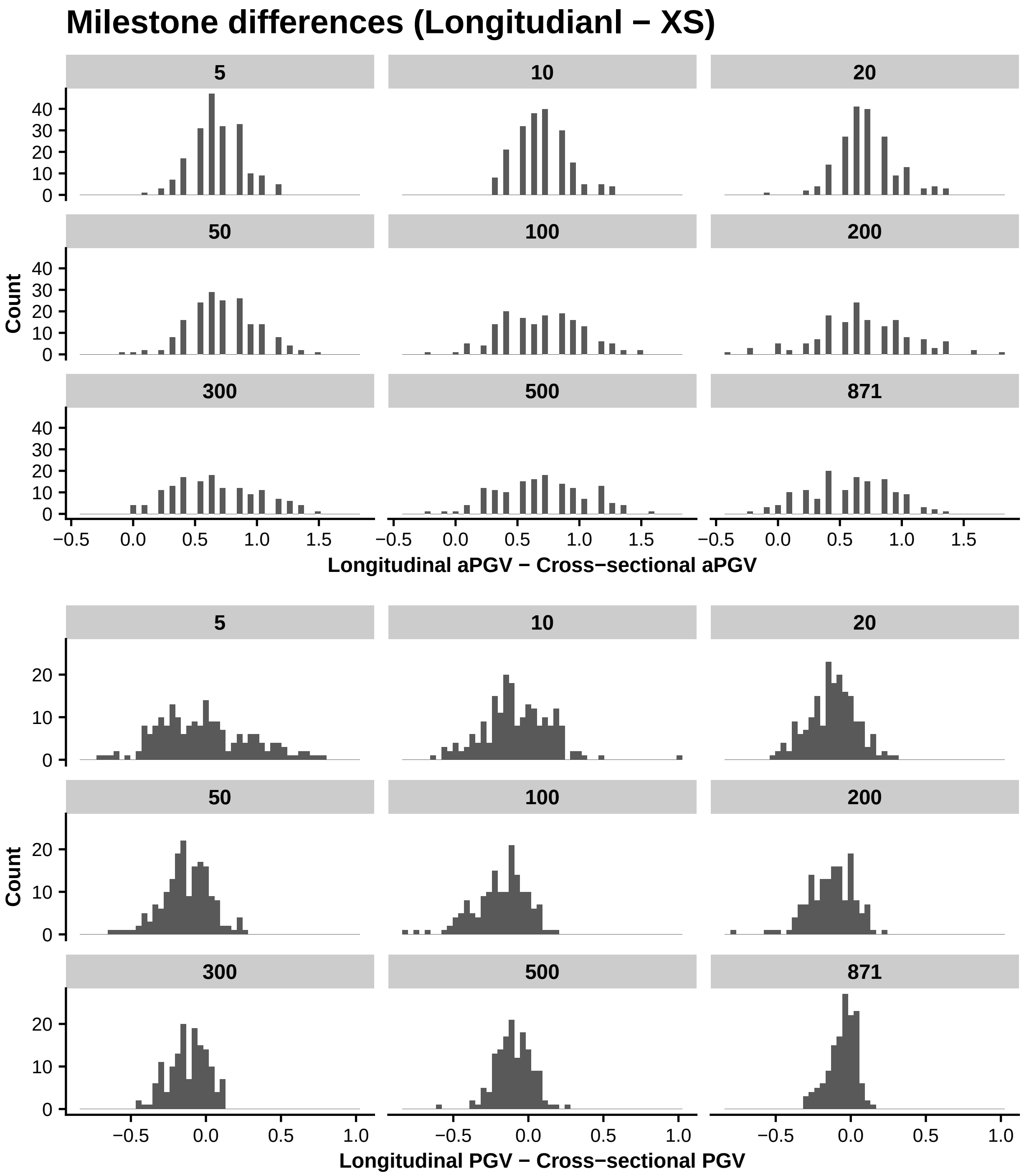
Milestone differences (Longitudianl − XS)
5
10
20
50
100
200
300
500
871
Count
Longitudinal aPGV − Cross−sectional aPGV
Longitudinal PGV − Cross−sectional PGV

12 Female, Sella-Basion

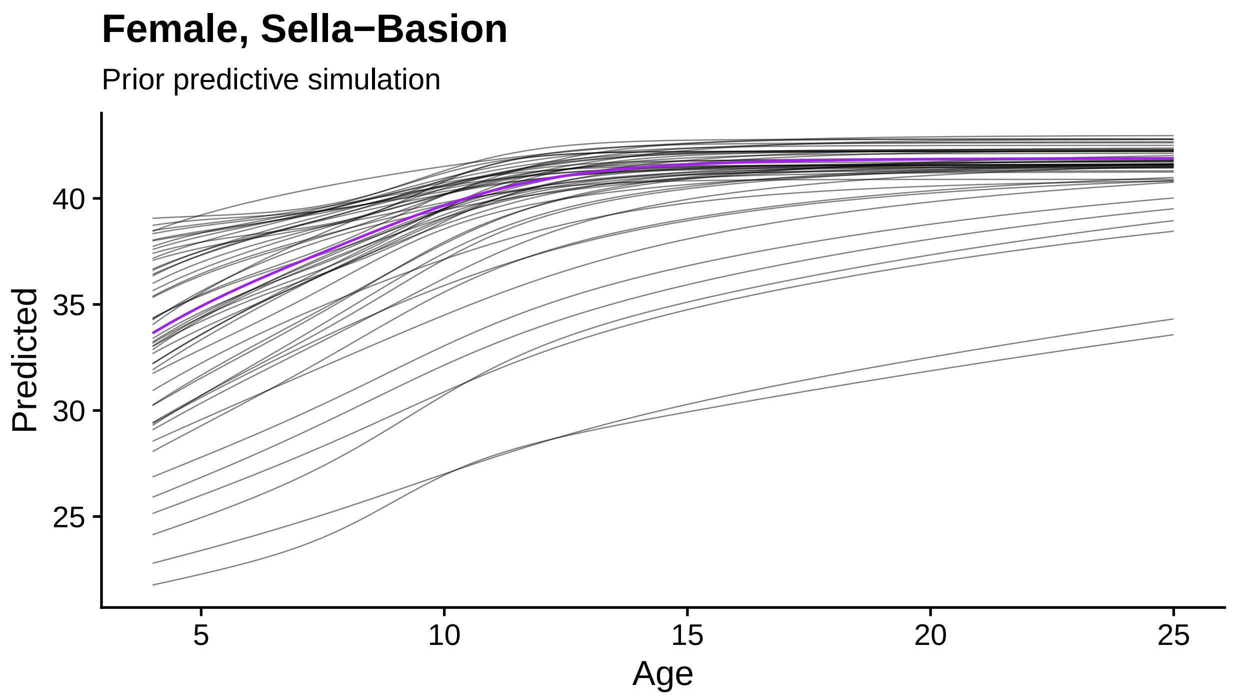

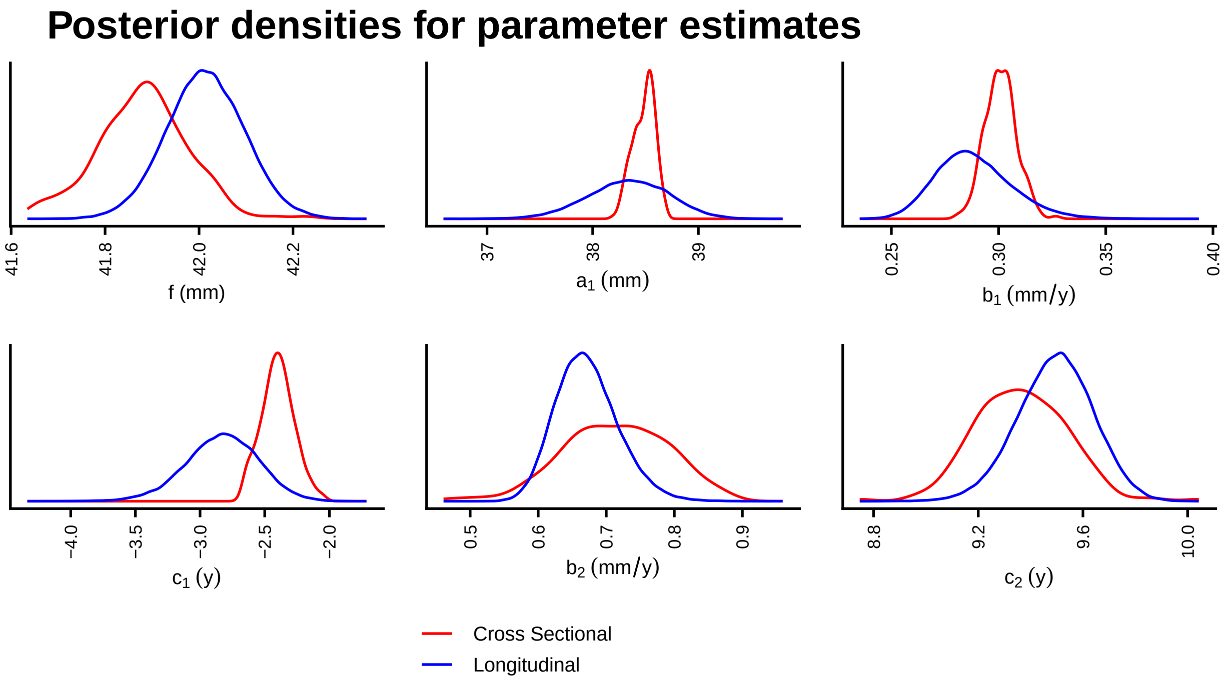

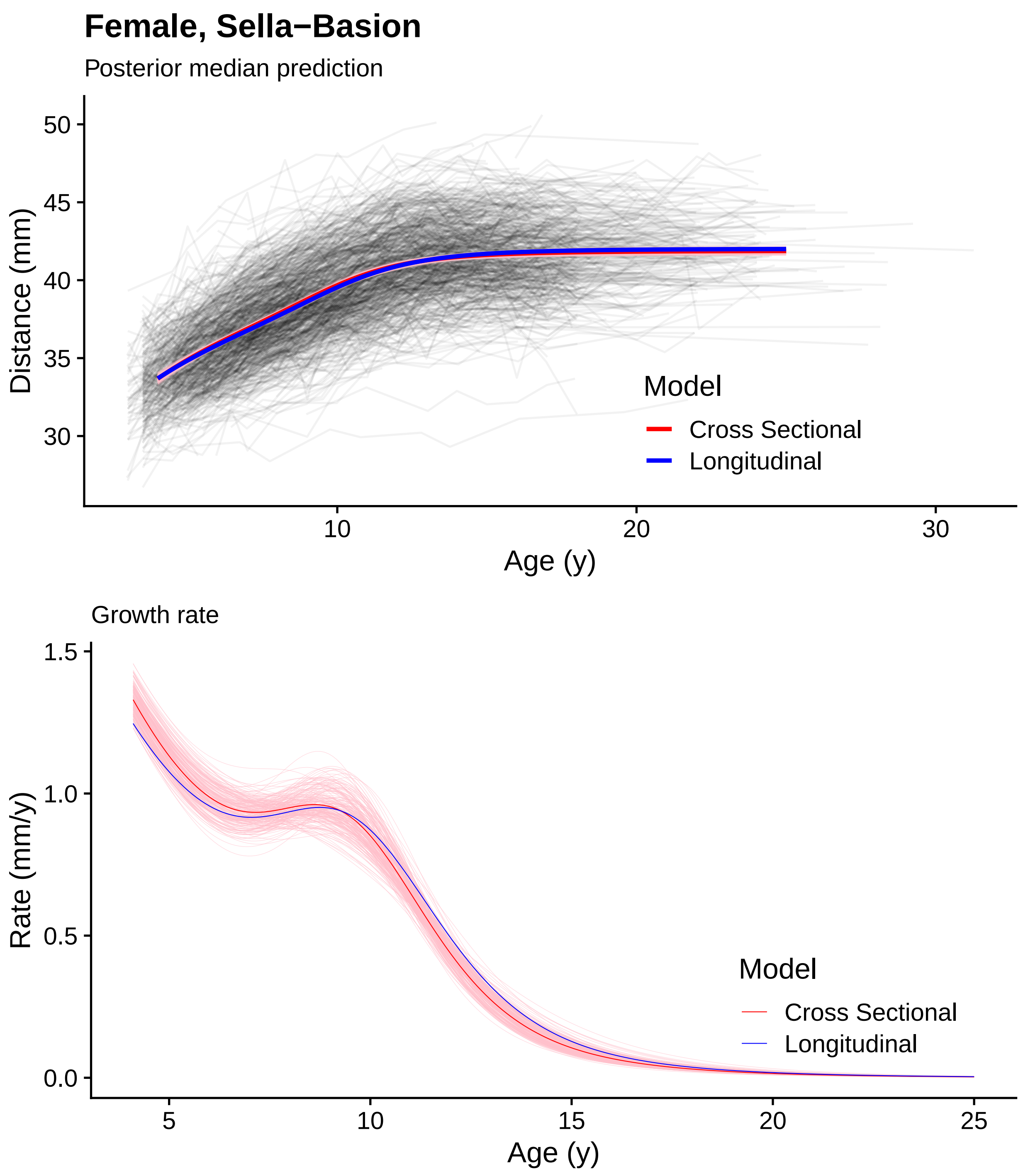
Female, Sella−Basion
Posterior median prediction
Distance (mm)
50
45
40
35
30
10
20
30
Age (y)
Model
Cross Sectional
Longitudinal
Growth rate
Rate (mm/y)
1.5
1.0
0.5
0.0
5
10
15
20
25
Age (y)
Model
Cross Sectional
Longitudinal

Table 21: Longitudinal Model Summary

Parameter	Mean	Median	Std. Dev.	MAD	5%	95%	$\hat{r}$	Bulk ESS	Tail ESS
f	42.01	42.01	0.083	0.083	41.88	42.15	1	1912	4465
a1	38.34	38.35	0.382	0.387	37.69	38.95	1	16367	21749
b1	0.29	0.29	0.018	0.017	0.26	0.32	1	17850	21223
c1	-2.83	-2.82	0.282	0.278	-3.30	-2.38	1	20595	23668
b2	0.67	0.67	0.046	0.045	0.60	0.75	1	18211	26124
c2	9.49	9.50	0.145	0.144	9.25	9.73	1	19794	24150
sigma	1.18	1.18	0.010	0.010	1.16	1.19	1	45235	30712
sigma_ID	2.22	2.22	0.053	0.054	2.13	2.31	1	50332	31358

Table 22: Median Coefficients

Model	f	a_1	b_1	c_1	b_2	c_2	σ	σ_{ID}
Longitudinal	42.01	38.35	0.29	-2.82	0.67	9.50	1.18	2.22
Cross Sectional	41.88	38.51	0.30	-2.40	0.72	9.36	2.49	NA

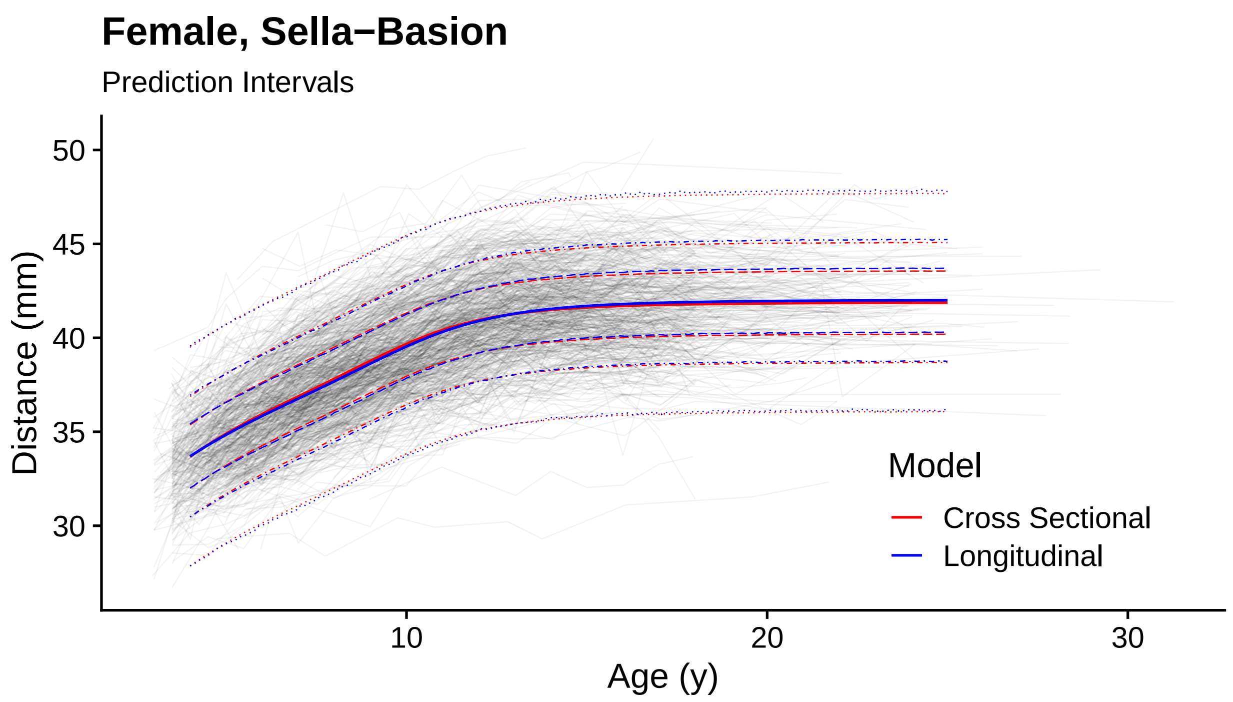
Female, Sella−Basion
Prediction Intervals
Distance (mm)
50
45
40
35
30
10
20
30
Age (y)
Model
Cross Sectional
Longitudinal

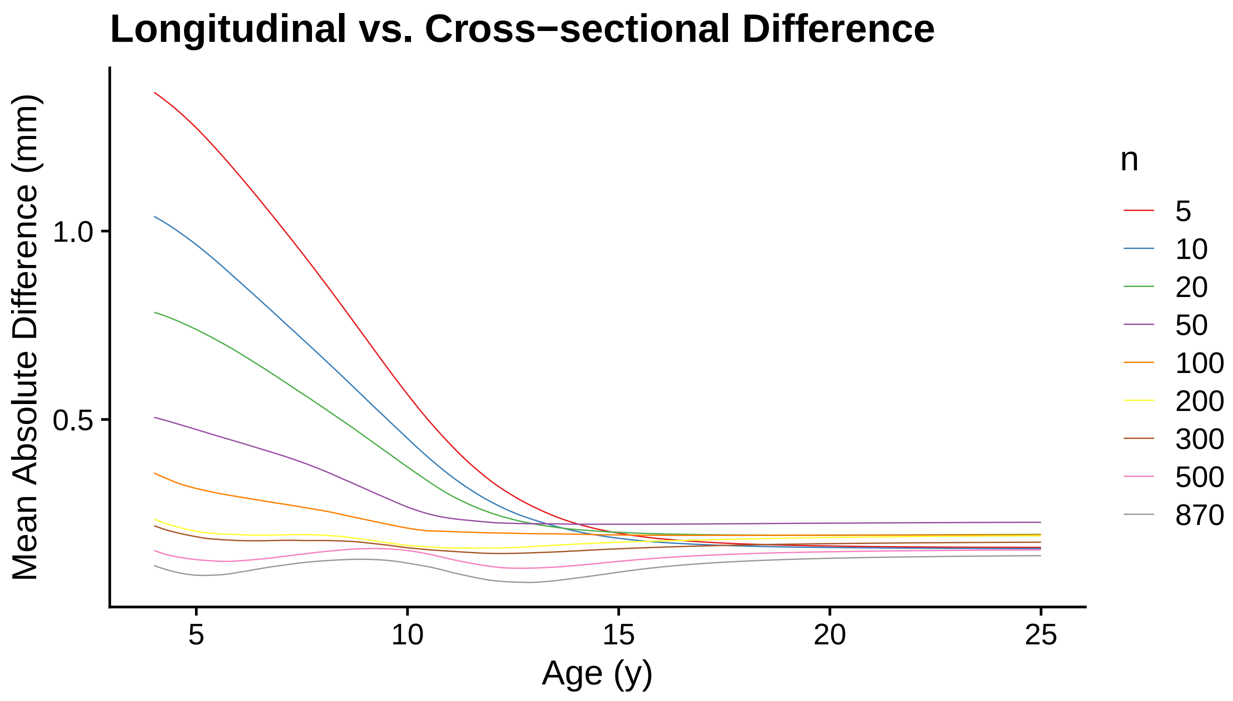
Longitudinal vs. Cross−sectional Difference
Mean Absolute Difference (mm)
1.0
0.5
5
10
15
20
25
Age (y)
n
5
10
20
50
100
200
300
500
870

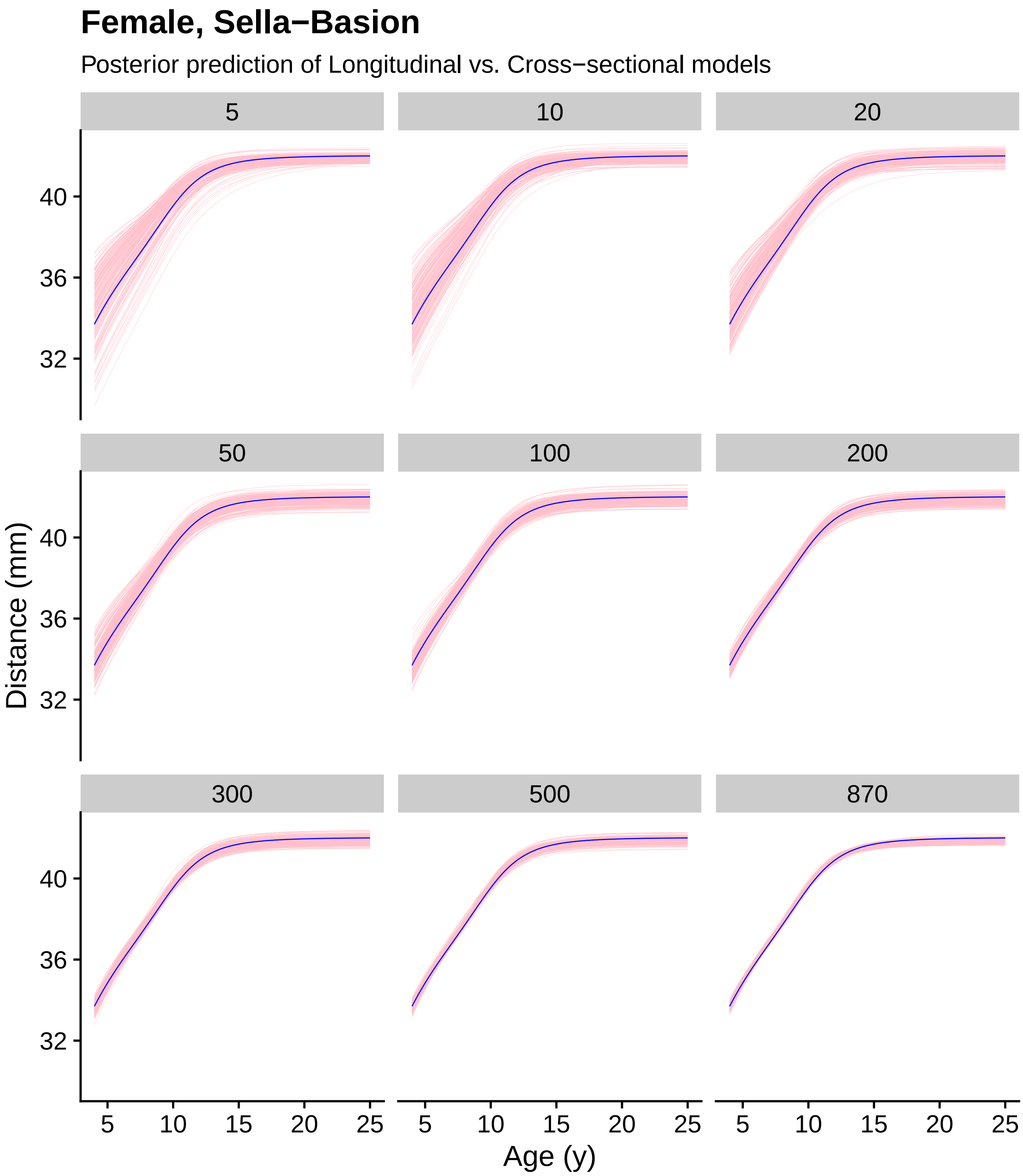

Female, Sella−Basion
Posterior prediction of Longitudinal vs. Cross−sectional models
5
10
20
50
100
200
300
500
870
Distance (mm)
40
36
32
5
10
15
20
25
Age (y)

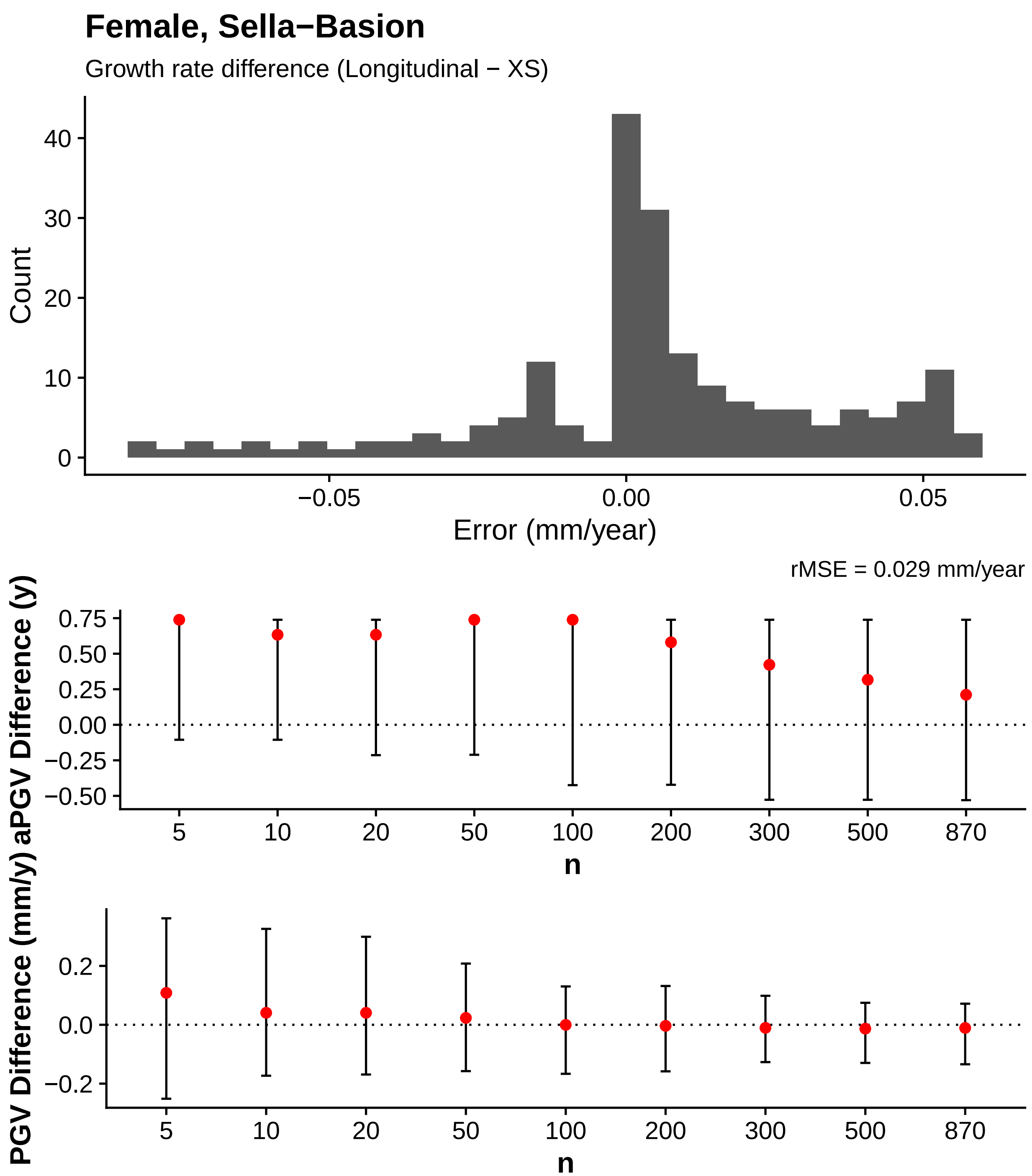
Female, Sella−Basion
Growth rate difference (Longitudinal − XS)
Count
0
10
20
30
40
−0.05
0.00
0.05
Error (mm/year)
rMSE = 0.029 mm/year
aPGV Difference (y)
0.75
0.50
0.25
0.00
−0.25
−0.50
5
10
20
50
100
200
300
500
870
n
PGV Difference (mm/y)
0.2
0.0
−0.2
5
10
20
50
100
200
300
500
870
n

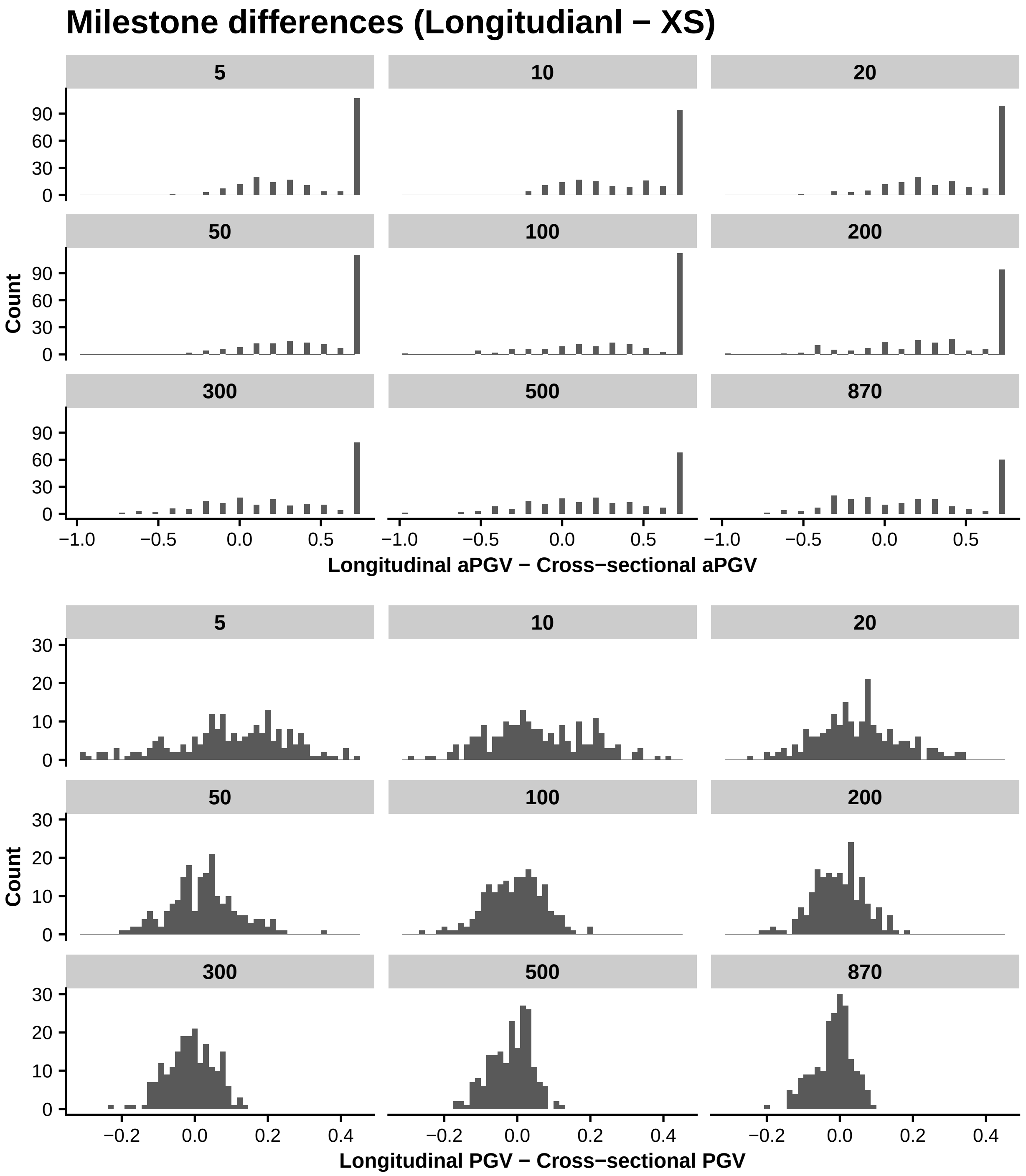
Milestone differences (Longitudianl − XS)
5
10
20
50
100
200
300
500
870
Count
0
30
60
90
−1.0
−0.5
0.0
0.5
Longitudinal aPGV − Cross−sectional aPGV
0
10
20
30
−0.2
0.0
0.2
0.4
Longitudinal PGV − Cross−sectional PGV

13 Female, Sella-Gonion

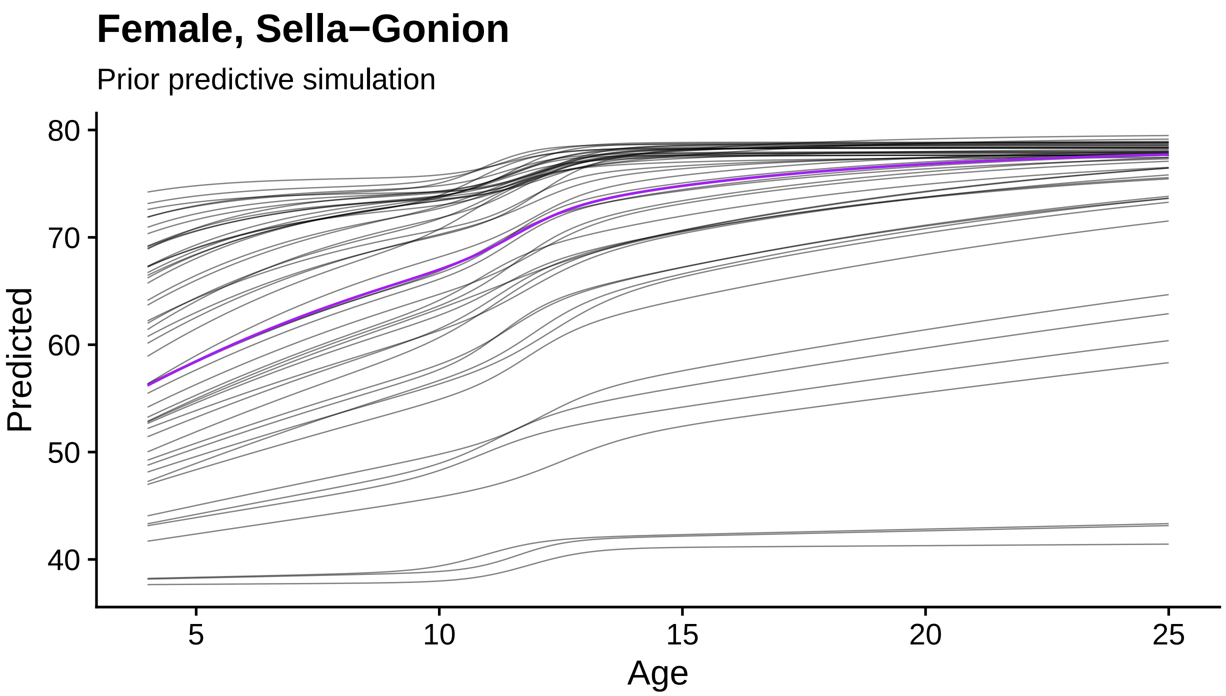

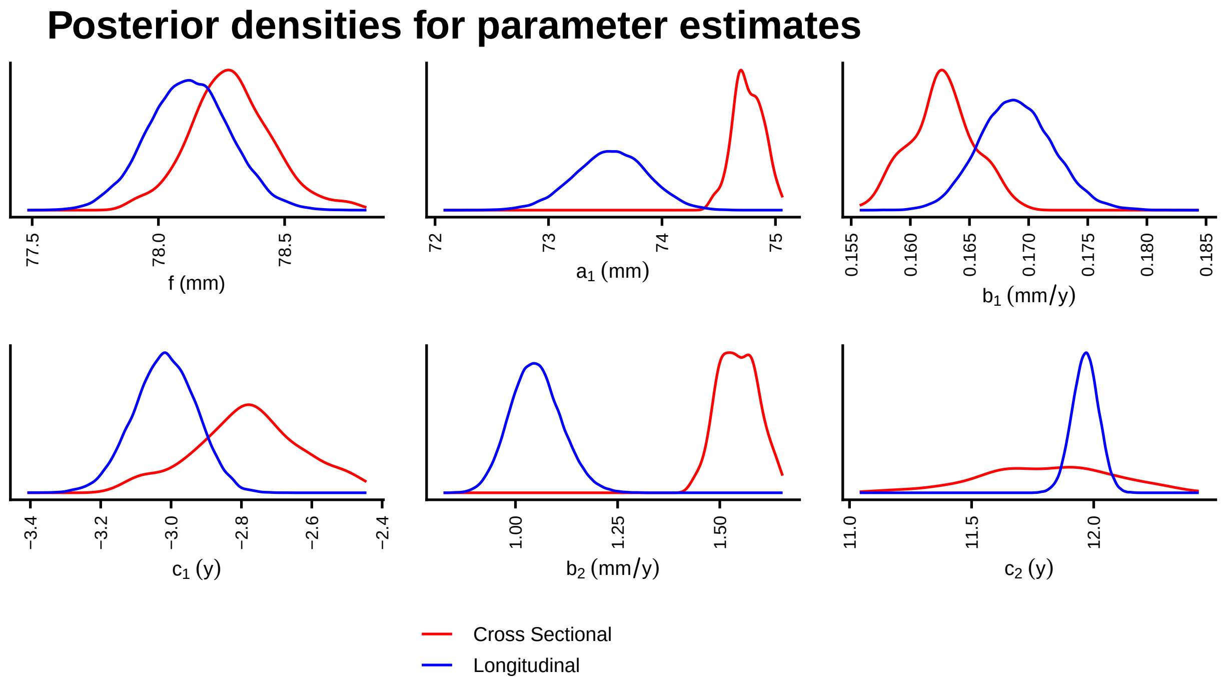

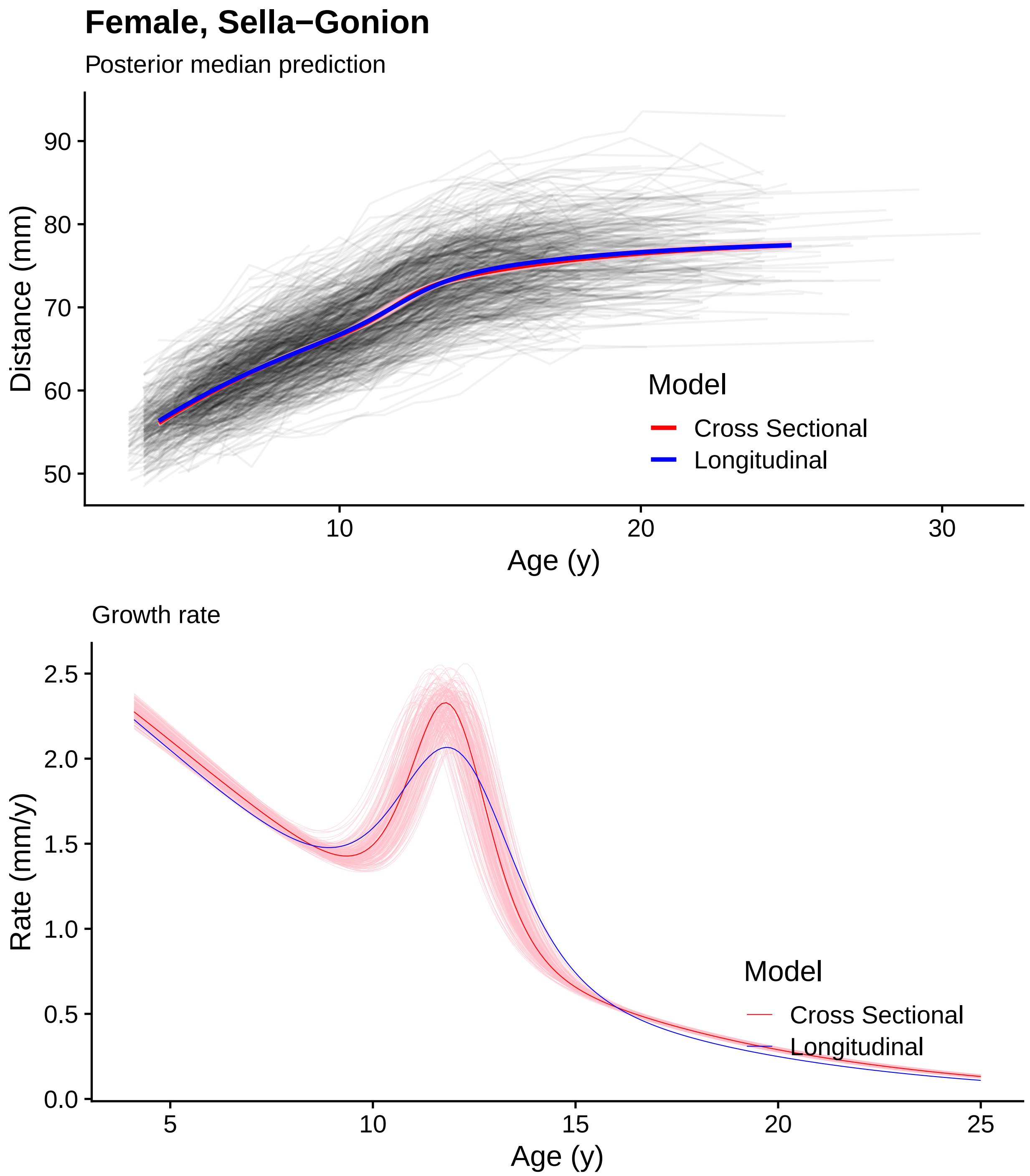
Female, Sella−Gonion
Posterior median prediction
Distance (mm)
90
80
70
60
50
10
20
30
Age (y)
Model
Cross Sectional
Longitudinal
Growth rate
Rate (mm/y)
2.5
2.0
1.5
1.0
0.5
0.0
5
10
15
20
25
Age (y)
Model
Cross Sectional
Longitudinal

Table 23: Longitudinal Model Summary

Parameter	Mean	Median	Std. Dev.	MAD	5%	95%	$\hat{r}$	Bulk ESS	Tail ESS
f	78.12	78.12	0.162	0.163	77.85	78.39	1	2194	5266
a1	73.55	73.55	0.322	0.322	73.02	74.07	1	13159	22584
b1	0.17	0.17	0.003	0.003	0.16	0.17	1	14350	21612
c1	-3.02	-3.02	0.090	0.090	-3.17	-2.87	1	17789	25886
b2	1.05	1.05	0.062	0.062	0.95	1.16	1	21926	27778
c2	11.97	11.97	0.054	0.054	11.88	12.05	1	34810	32073
sigma	1.49	1.49	0.012	0.012	1.47	1.51	1	50396	29642
sigma_ID	3.61	3.61	0.085	0.085	3.48	3.76	1	56036	29809

Table 24: Median Coefficients

Model	f	a_1	b_1	c_1	b_2	c_2	σ	σ_{ID}
Longitudinal	78.12	73.55	0.17	-3.02	1.05	11.97	1.49	3.61
Cross Sectional	78.28	74.74	0.16	-2.78	1.54	11.81	3.90	NA

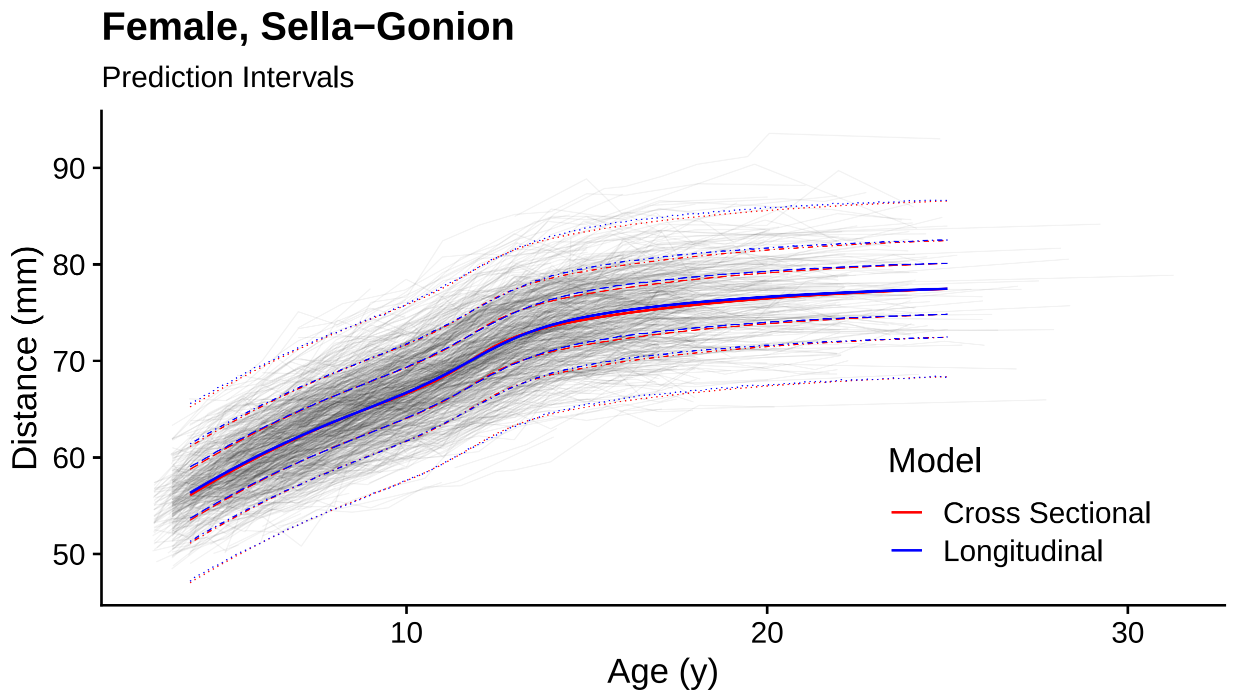

Female, Sella−Gonion
Prediction Intervals
Distance (mm)
90
80
70
60
50
10
20
30
Age (y)
Model
Cross Sectional
Longitudinal

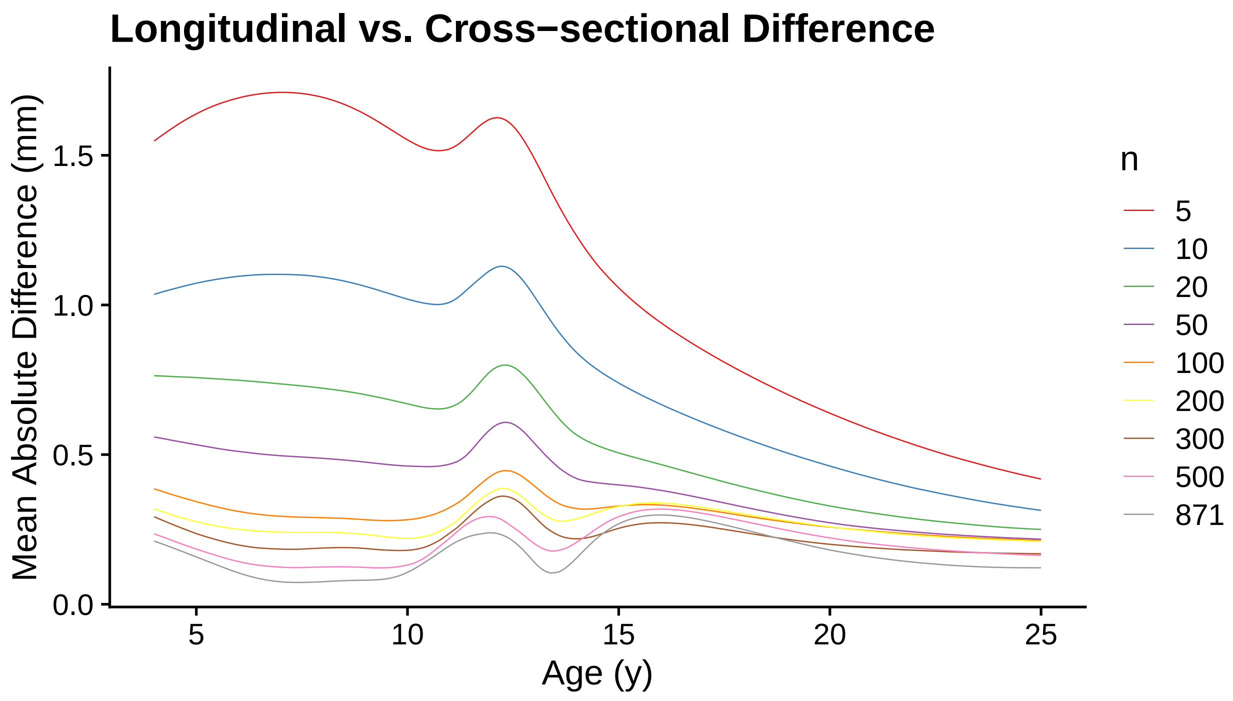

Longitudinal vs. Cross−sectional Difference
Mean Absolute Difference (mm)
1.5
1.0
0.5
0.0
5
10
15
20
25
Age (y)
n
5
10
20
50
100
200
300
500
871

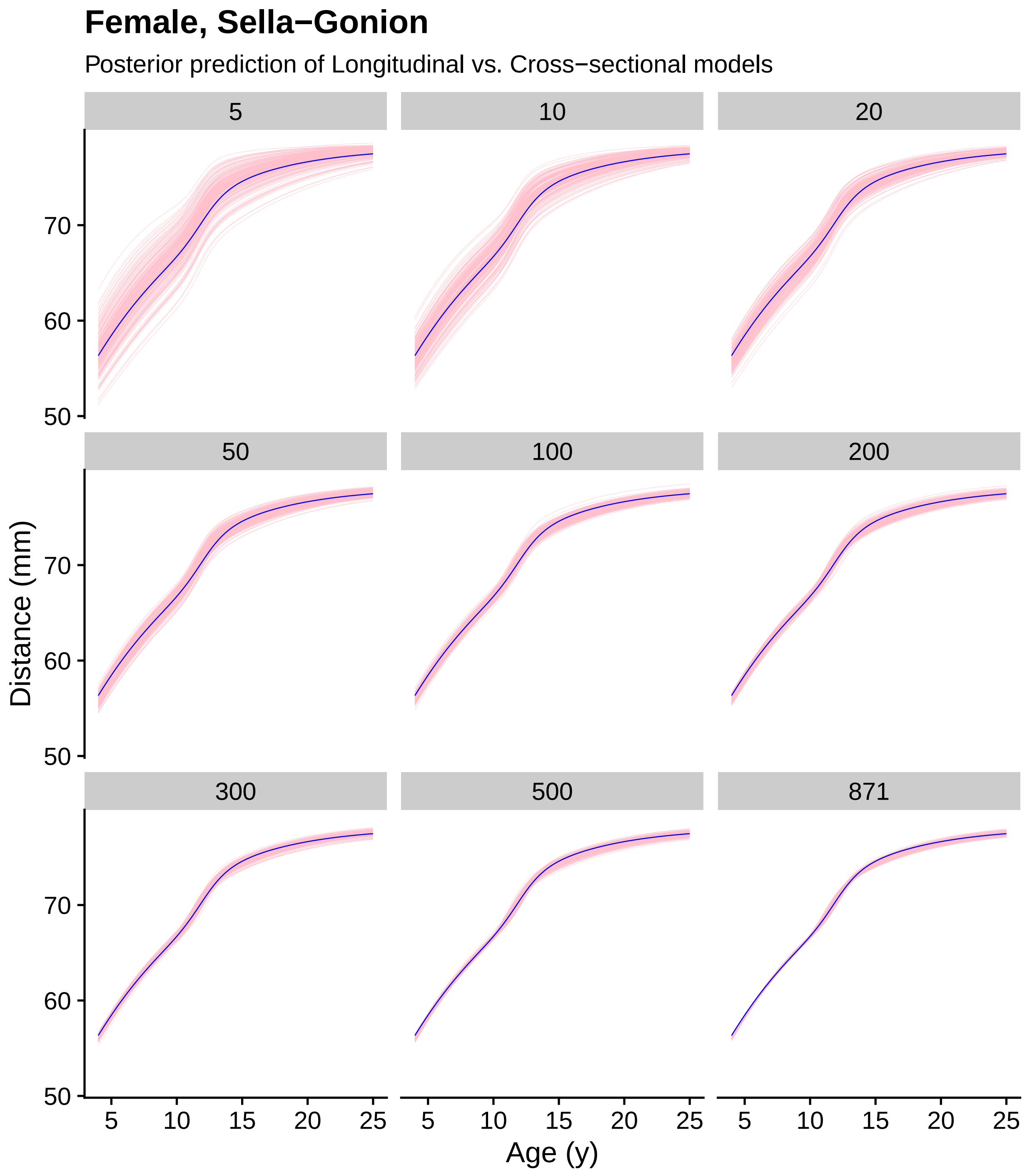
Female, Sella−Gonion
Posterior prediction of Longitudinal vs. Cross−sectional models
5
10
20
50
100
200
300
500
871
Distance (mm)
Age (y)
50
60
70
5
10
15
20
25

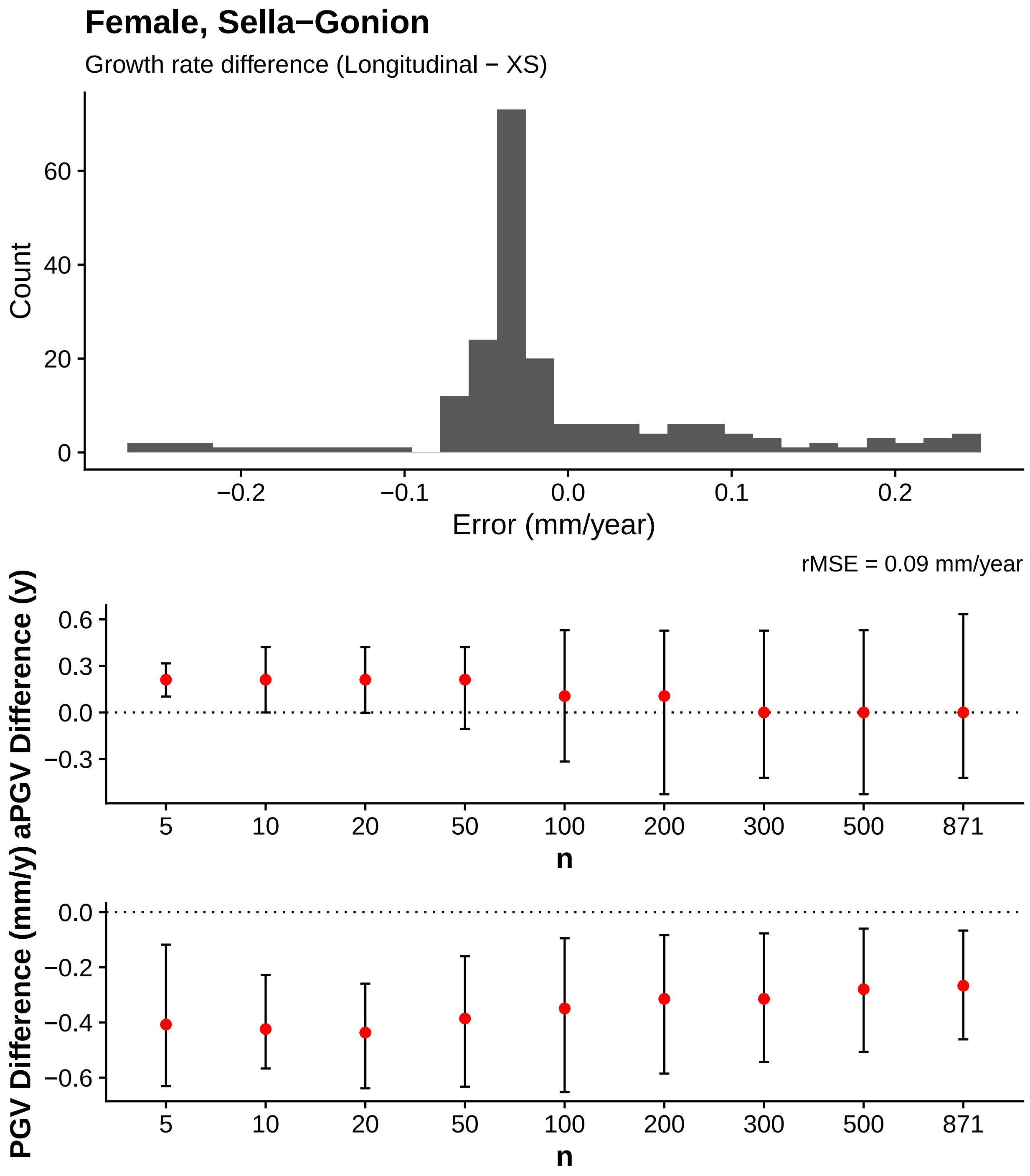

Female, Sella−Gonion
Growth rate difference (Longitudinal − XS)
Count
Error (mm/year)
rMSE = 0.09 mm/year
PGV Difference (mm/y) aPGV Difference (y)
n

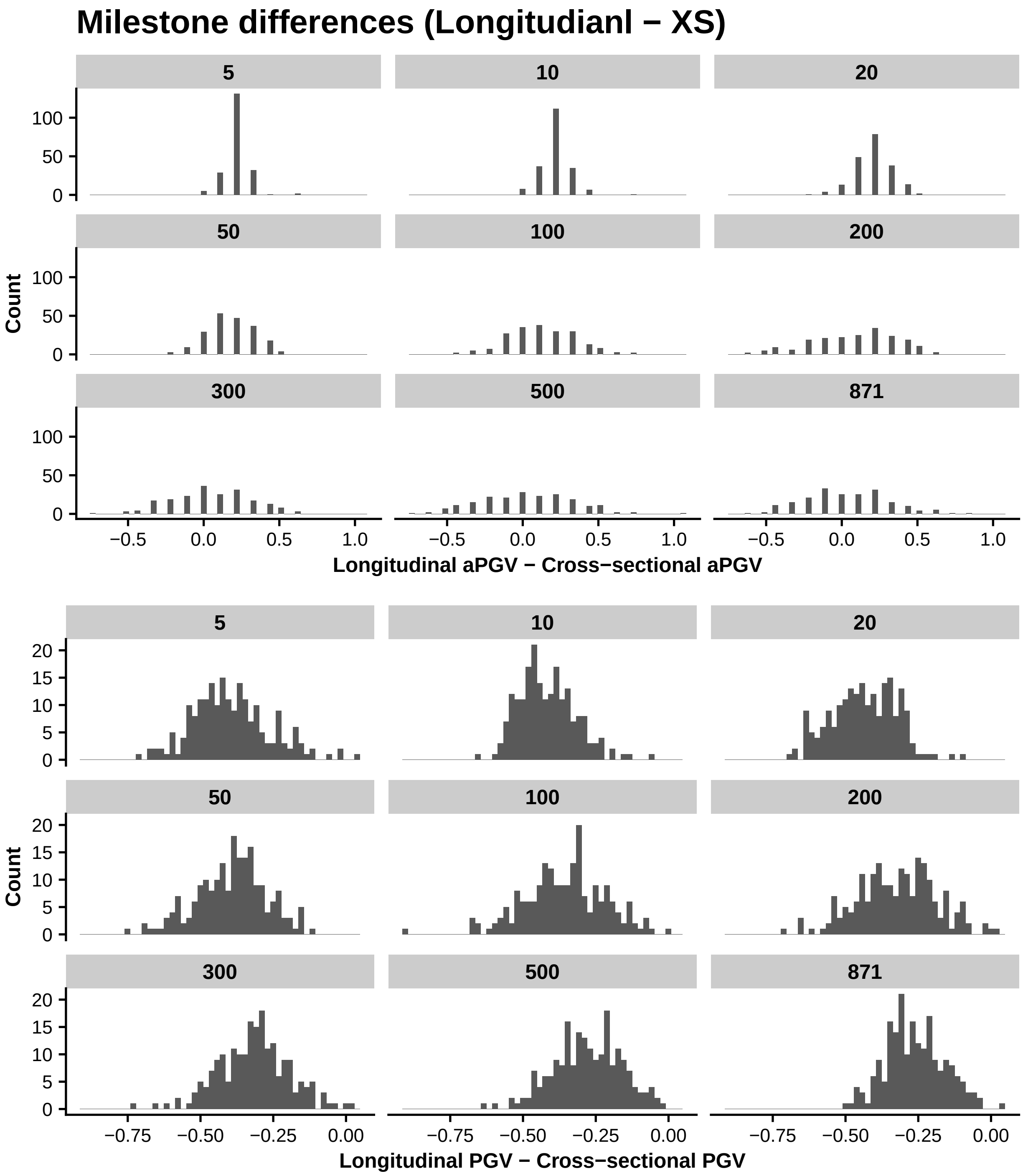
Milestone differences (Longitudianl − XS)
5
10
20
50
100
200
300
500
871
Count
100
50
0
−0.5
0.0
0.5
1.0
Longitudinal aPGV − Cross−sectional aPGV
20
15
10
5
−0.75
−0.50
−0.25
0.00
Longitudinal PGV − Cross−sectional PGV

14 Female, Sella-Nasion

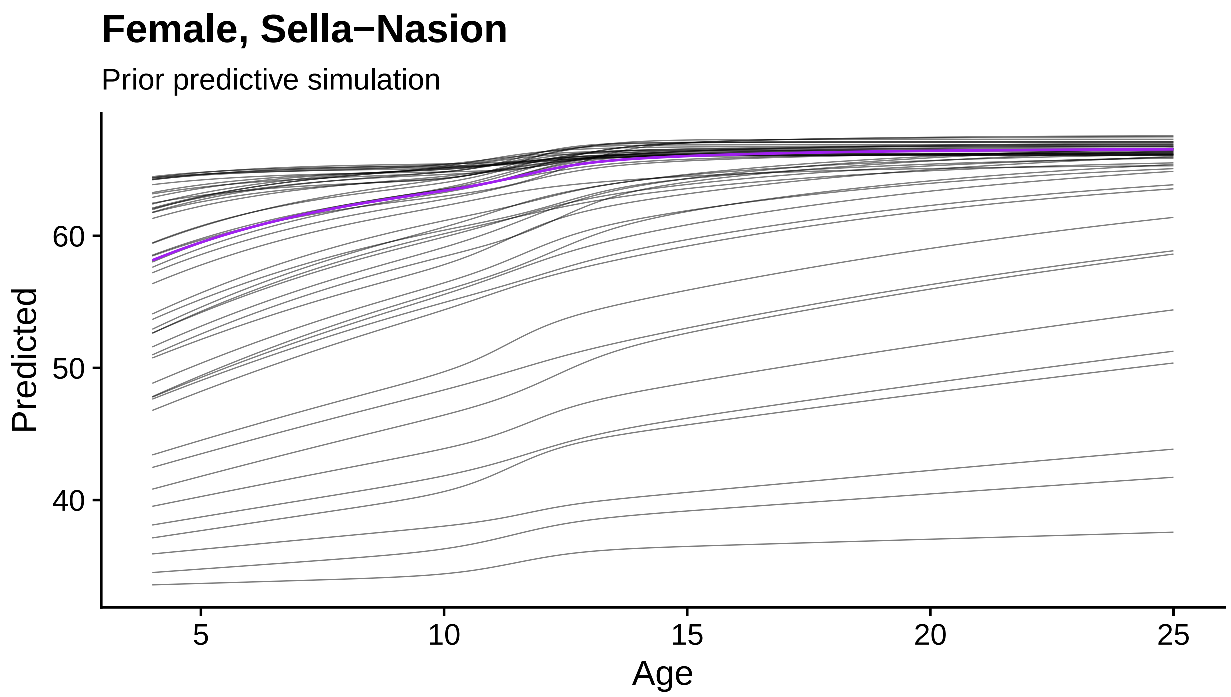

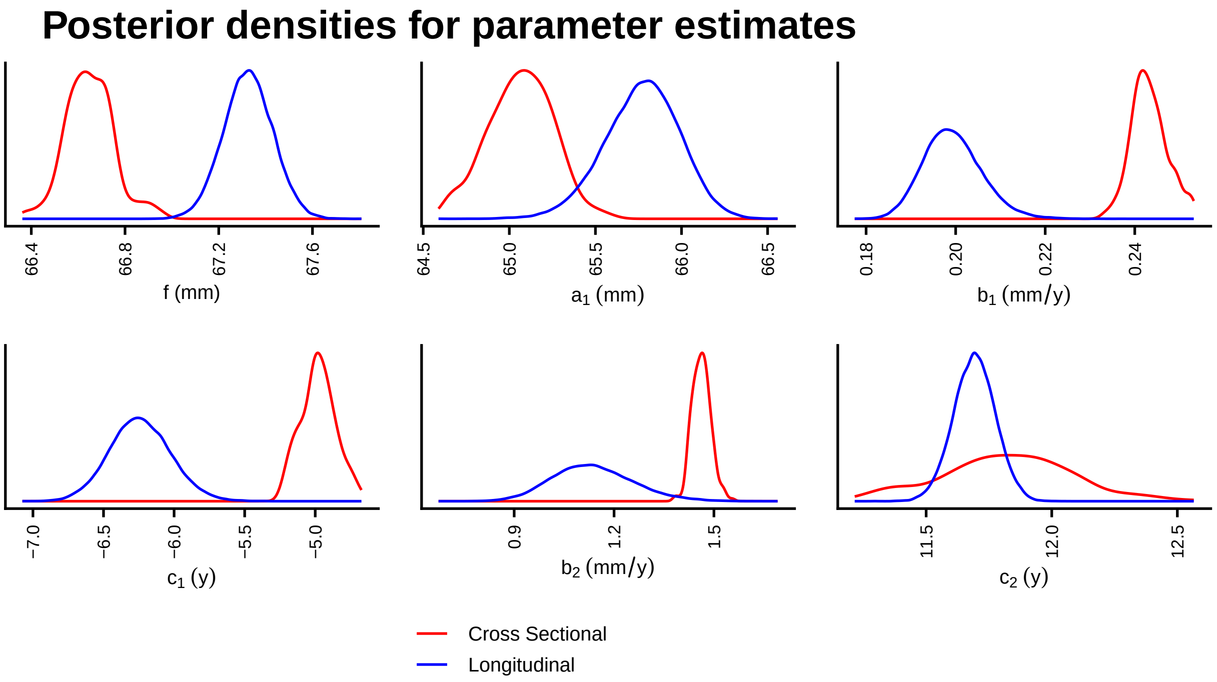

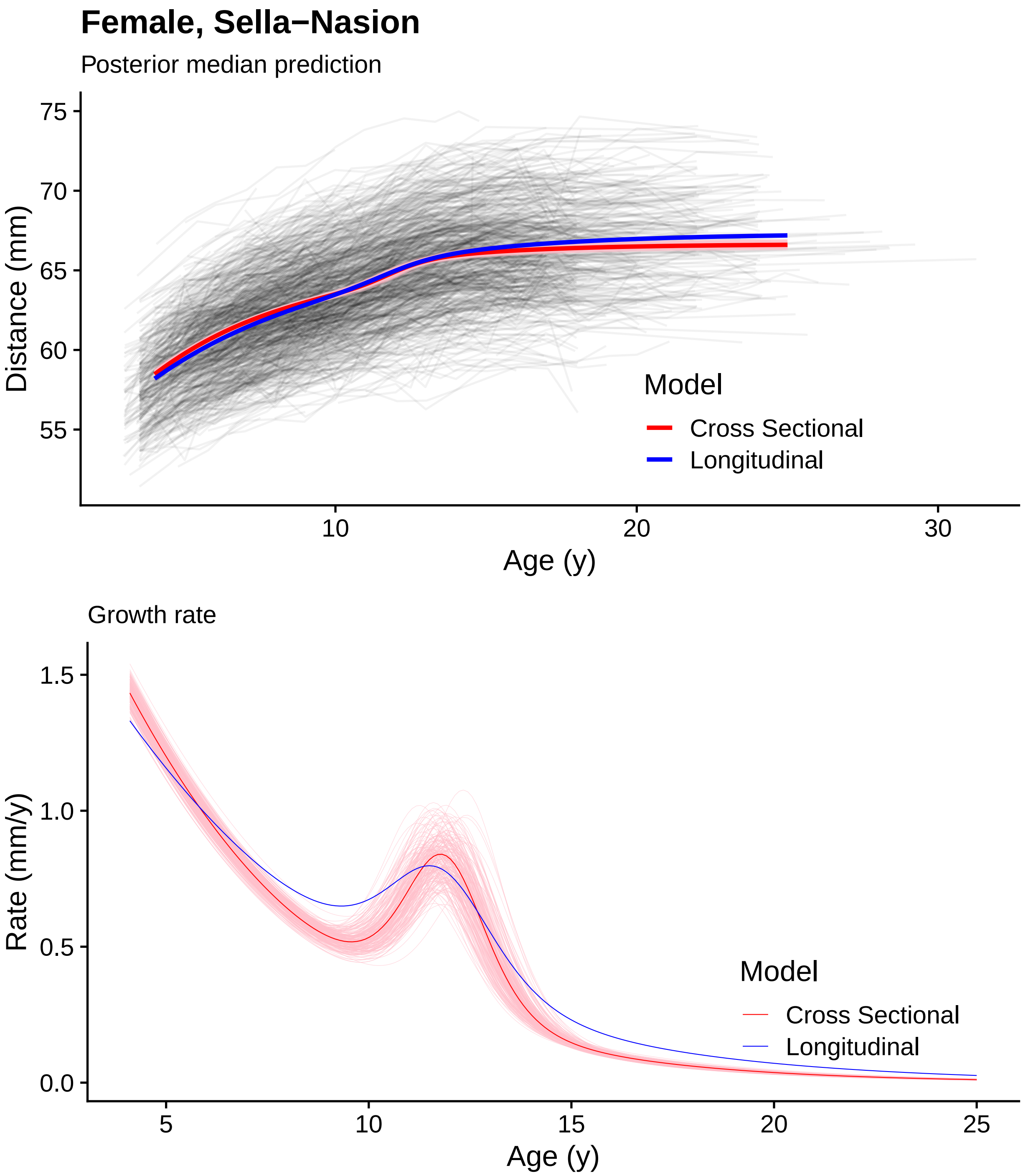
Female, Sella−Nasion
Posterior median prediction
Distance (mm)
75
70
65
60
55
10
20
30
Age (y)
Model
Cross Sectional
Longitudinal
Growth rate
Rate (mm/y)
1.5
1.0
0.5
0.0
5
10
15
20
25
Age (y)
Model
Cross Sectional
Longitudinal

Table 25: Longitudinal Model Summary

Parameter	Mean	Median	Std. Dev.	MAD	5%	95%	$\hat{r}$	Bulk ESS	Tail ESS
f	67.32	67.33	0.105	0.105	67.15	67.50	1	1534	4152
a1	65.77	65.78	0.219	0.217	65.40	66.11	1	6925	15924
b1	0.20	0.20	0.007	0.006	0.19	0.21	1	16325	20530
c1	-6.24	-6.24	0.216	0.214	-6.59	-5.88	1	19100	23353
b2	1.14	1.13	0.121	0.120	0.95	1.35	1	23532	26942
c2	11.69	11.69	0.083	0.083	11.55	11.83	1	58460	29575
sigma	0.87	0.87	0.007	0.007	0.86	0.89	1	55458	30391
sigma_ID	2.76	2.76	0.065	0.065	2.66	2.87	1	72140	29861

Table 26: Median Coefficients

Model	f	a_1	b_1	c_1	b_2	c_2	σ	σ_{ID}
Longitudinal	67.33	65.78	0.20	-6.24	1.13	11.69	0.87	2.76
Cross Sectional	66.65	65.07	0.24	-4.98	1.46	11.82	2.88	NA

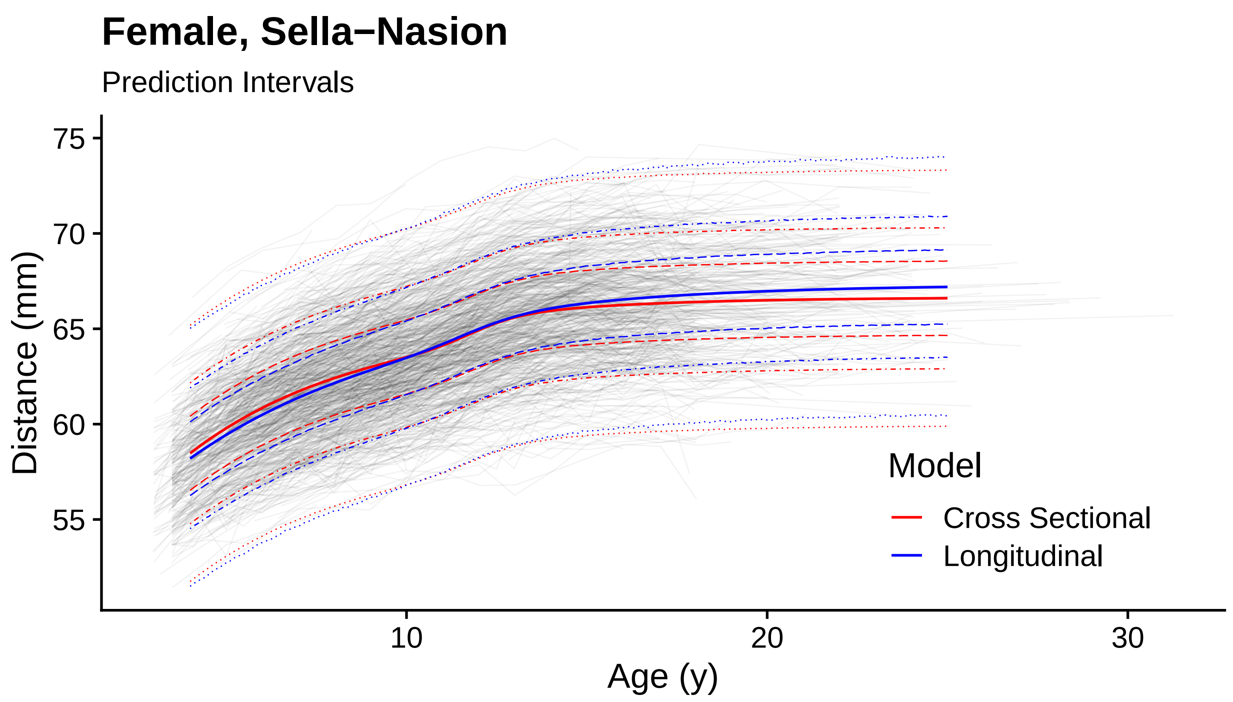
Female, Sella−Nasion
Prediction Intervals
Distance (mm)
75
70
65
60
55
10
20
30
Age (y)
Model
Cross Sectional
Longitudinal

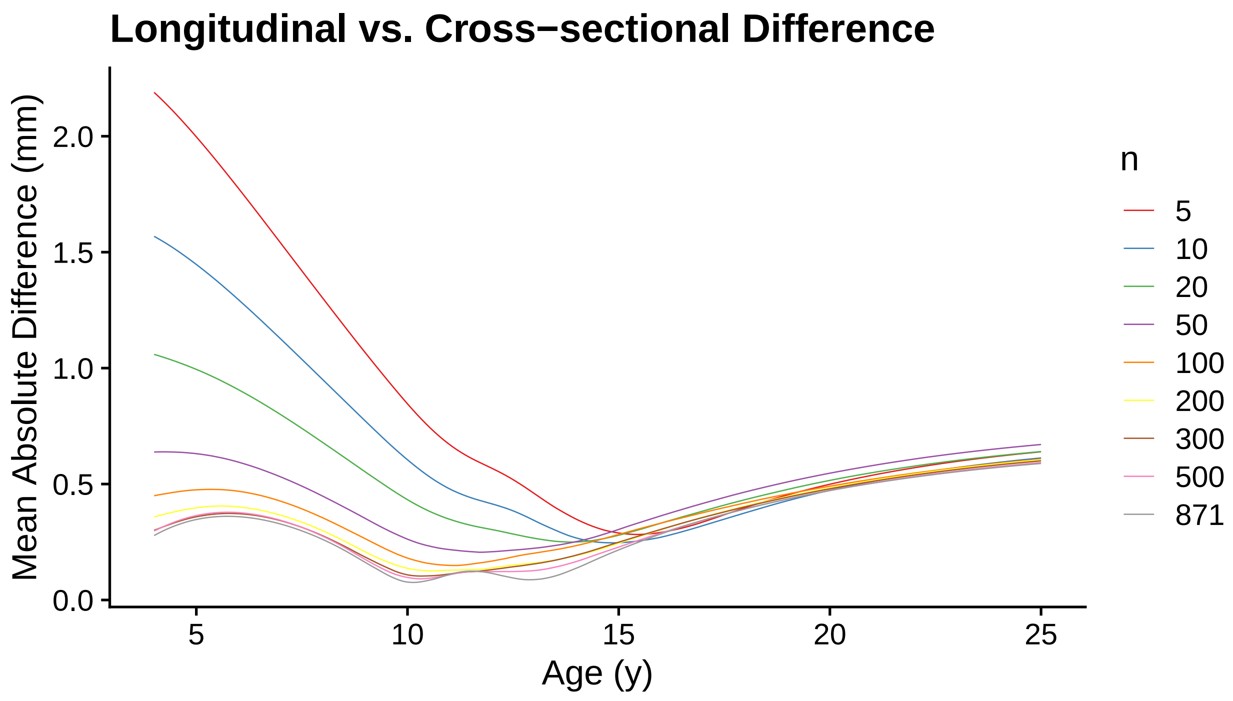
Longitudinal vs. Cross−sectional Difference
Mean Absolute Difference (mm)
2.0
1.5
1.0
0.5
0.0
5
10
15
20
25
Age (y)
n
5
10
20
50
100
200
300
500
871

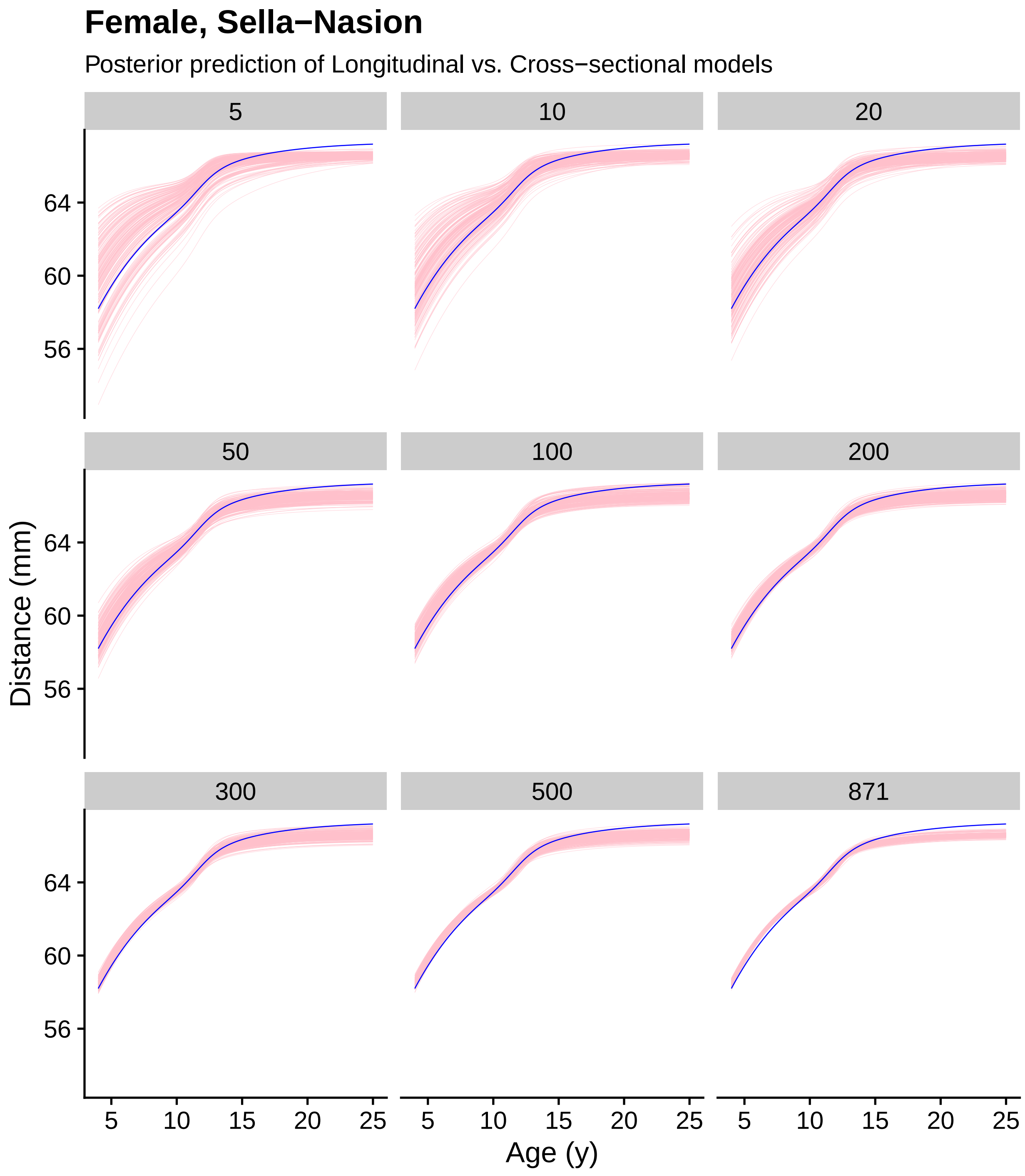
Female, Sella−Nasion
Posterior prediction of Longitudinal vs. Cross−sectional models
5
10
20
50
100
200
300
500
871
64
60
56
Distance (mm)
5
10
15
20
25
Age (y)

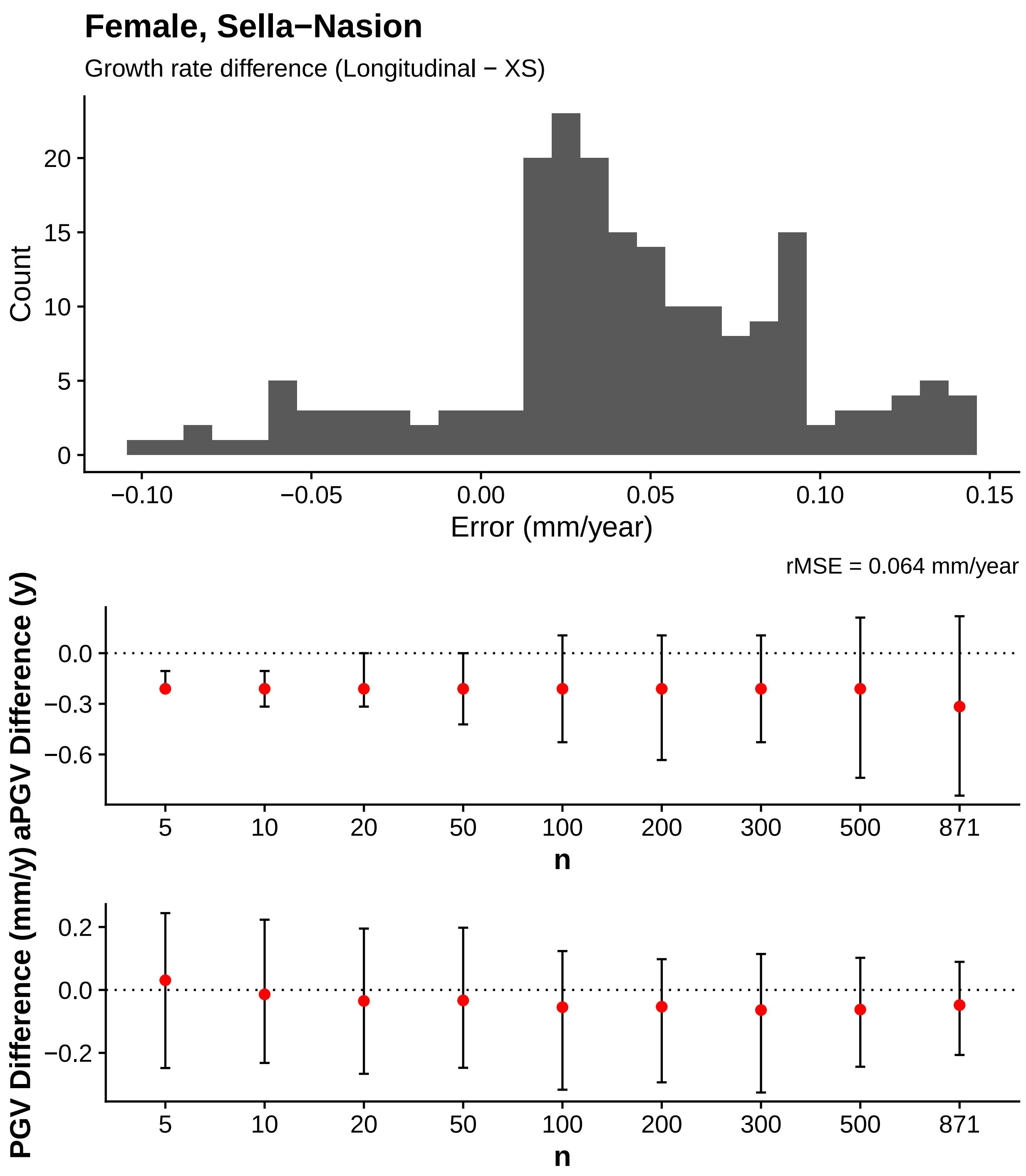

Female, Sella−Nasion
Growth rate difference (Longitudinal − XS)
Count
Error (mm/year)
rMSE = 0.064 mm/year
PGV Difference (mm/y) aPGV Difference (y)
n
n

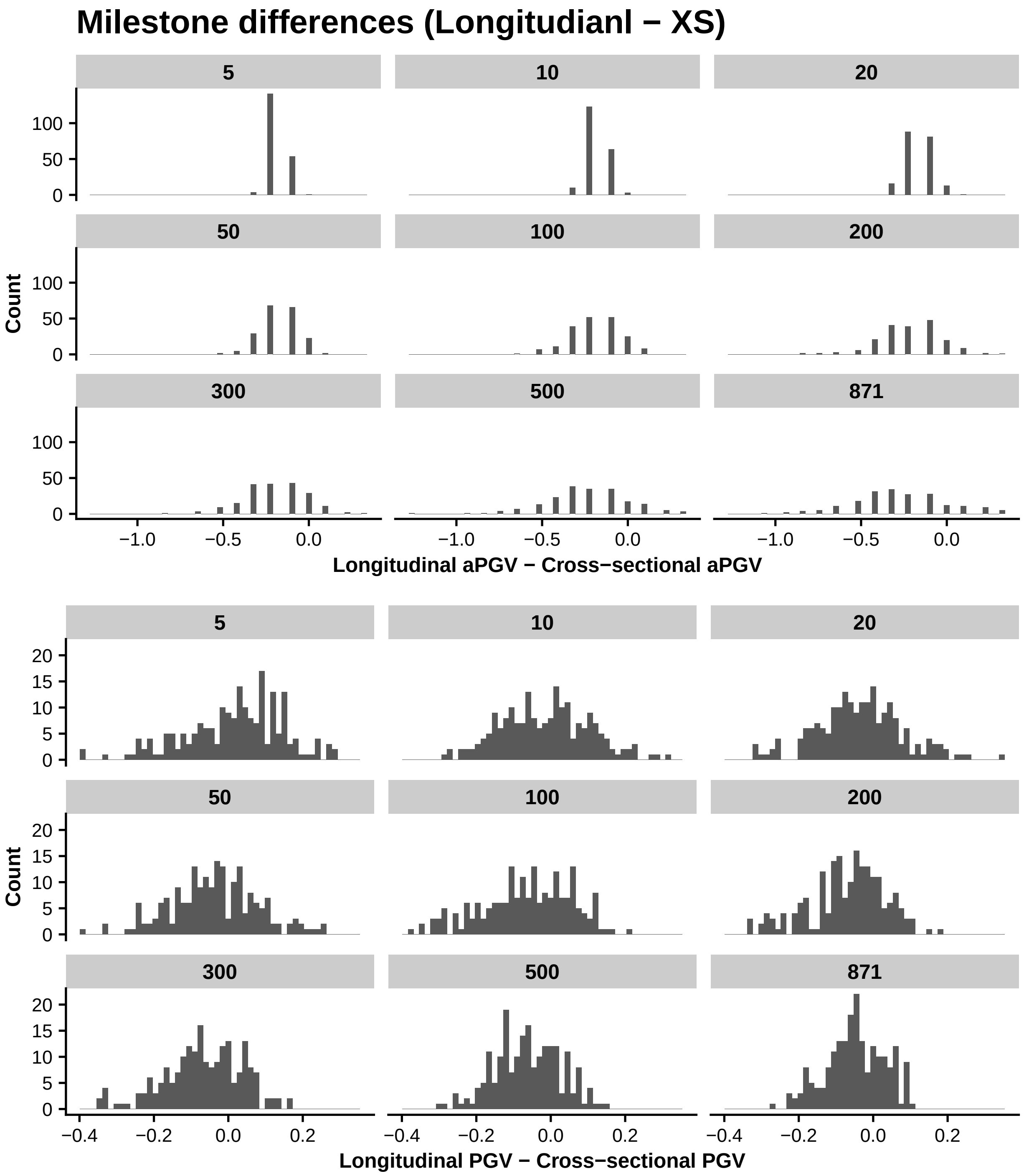
Milestone differences (Longitudianl − XS)
5
10
20
50
100
200
300
500
871
Count
Longitudinal aPGV − Cross−sectional aPGV
Longitudinal PGV − Cross−sectional PGV

15 Male, ANS-PNS

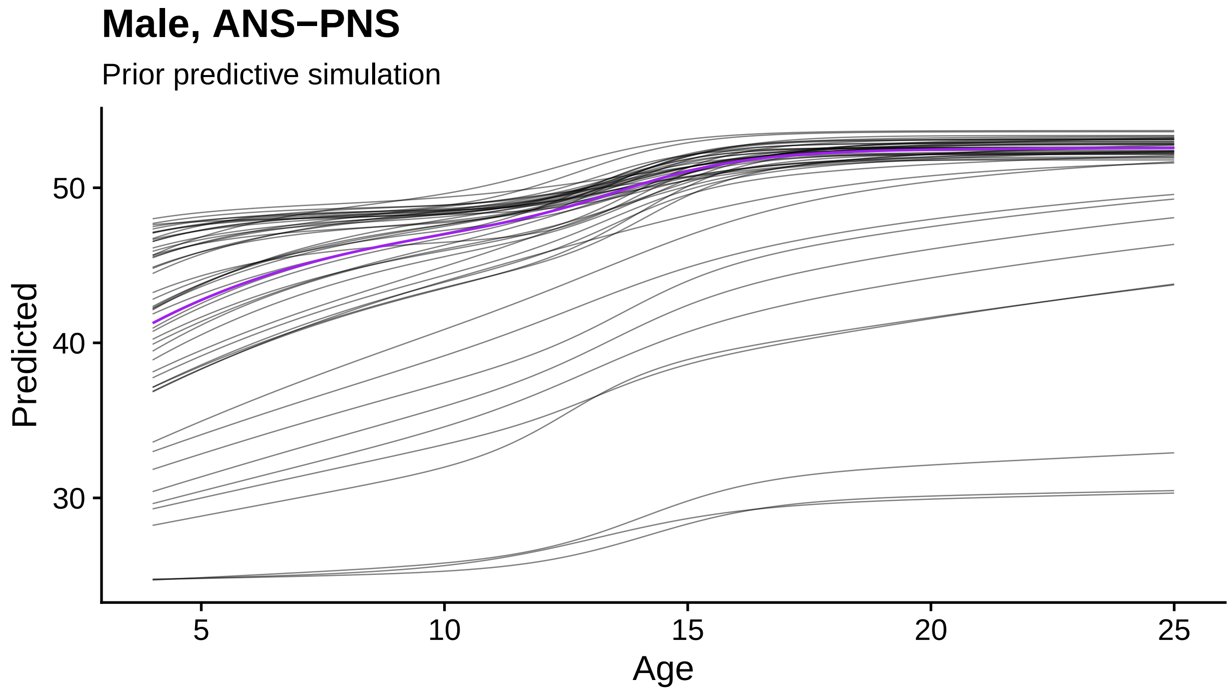

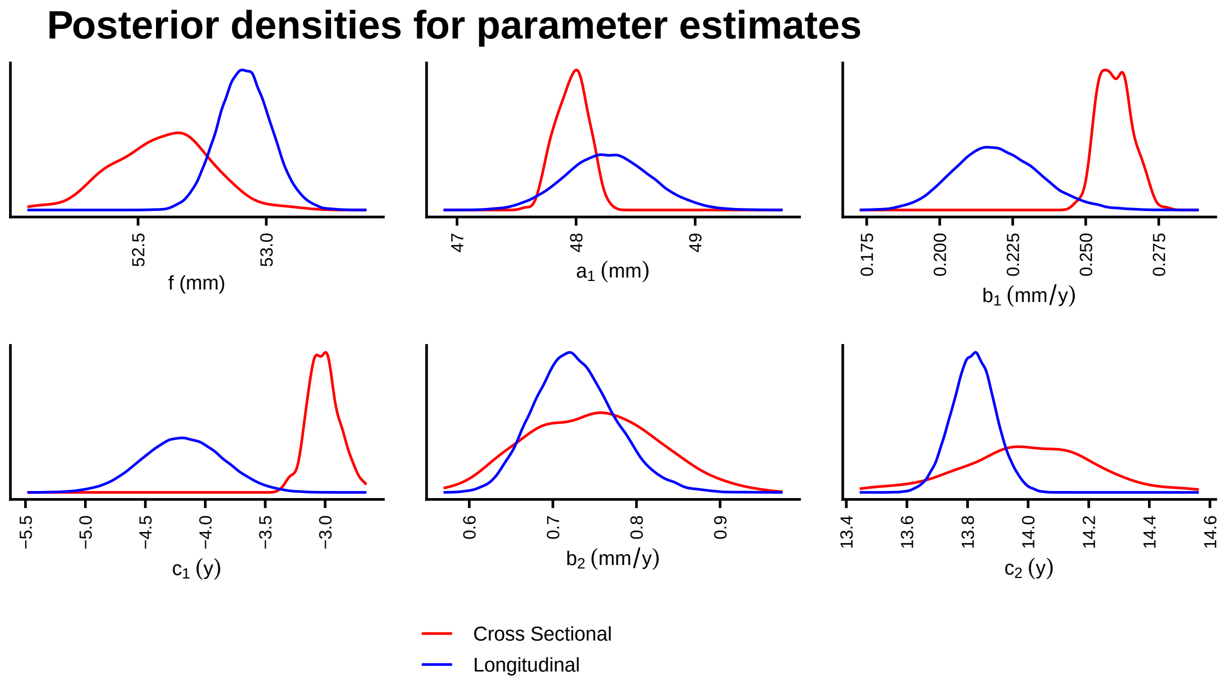

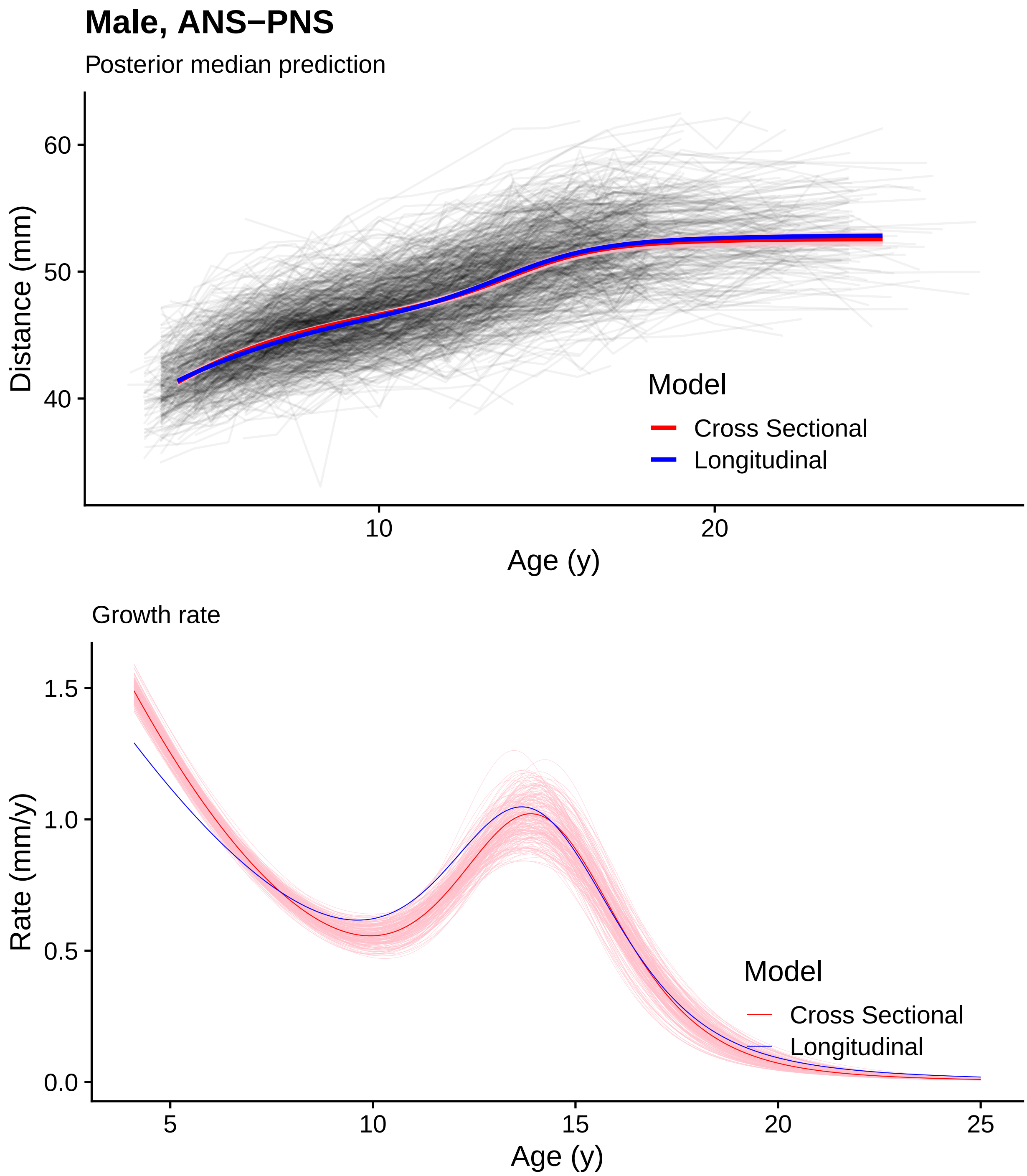

Male, ANS−PNS
Posterior median prediction
Distance (mm)
60
50
40
10
20
Age (y)
Model
Cross Sectional
Longitudinal
Growth rate
Rate (mm/y)
1.5
1.0
0.5
0.0
5
10
15
20
25
Age (y)
Model
Cross Sectional
Longitudinal

Table 27: Longitudinal Model Summary

Parameter	Mean	Median	Std. Dev.	MAD	5%	95%	$\hat{r}$	Bulk ESS	Tail ESS
f	52.92	52.91	0.104	0.103	52.75	53.09	1	4868	12021
a1	48.27	48.27	0.355	0.356	47.69	48.86	1	18443	23104
b1	0.22	0.22	0.015	0.015	0.20	0.24	1	18786	23018
c1	-4.19	-4.19	0.337	0.339	-4.74	-3.63	1	21237	25306
b2	0.73	0.72	0.048	0.047	0.65	0.81	1	26244	30035
c2	13.82	13.82	0.072	0.071	13.70	13.94	1	40290	31024
sigma	1.36	1.36	0.011	0.011	1.34	1.38	1	54728	30788
sigma_ID	2.32	2.32	0.055	0.055	2.24	2.42	1	67630	31766

Table 28: Median Coefficients

Model	f	a_1	b_1	c_1	b_2	c_2	σ	σ_{ID}
Longitudinal	52.91	48.27	0.22	-4.19	0.72	13.82	1.36	2.32
Cross Sectional	52.60	47.97	0.26	-3.01	0.75	14.01	2.67	NA

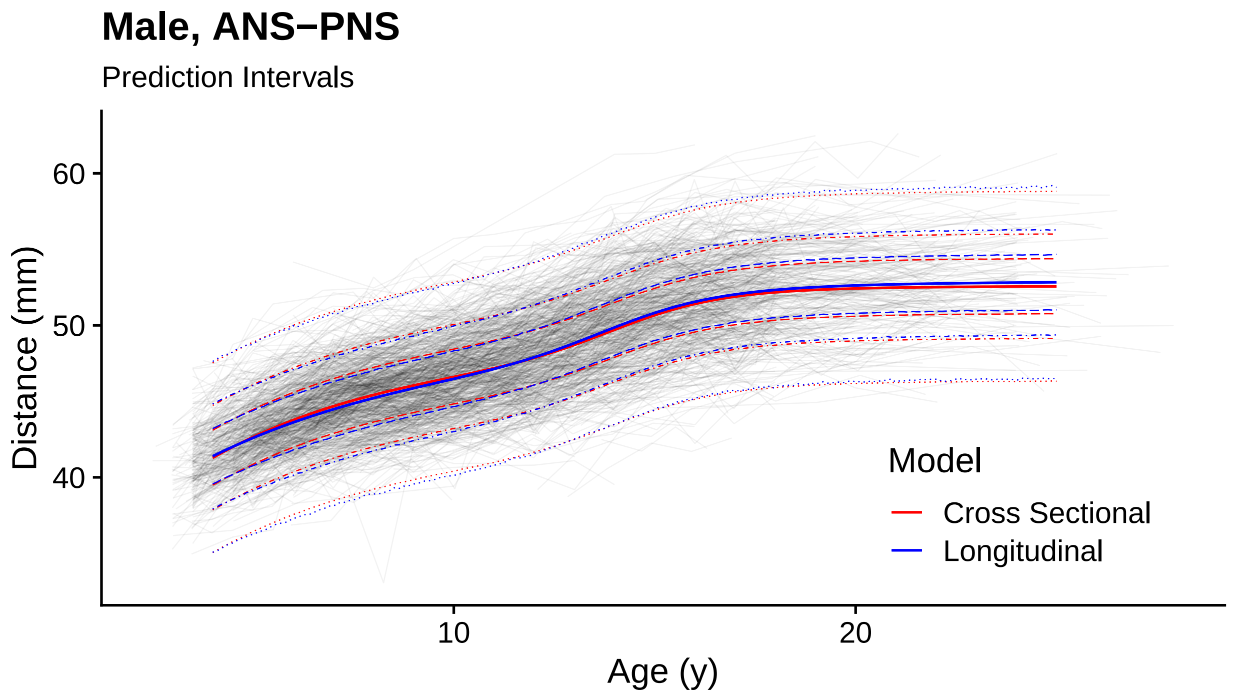
Male, ANS−PNS
Prediction Intervals
Distance (mm)
60
50
40
10
20
Age (y)
Model
Cross Sectional
Longitudinal

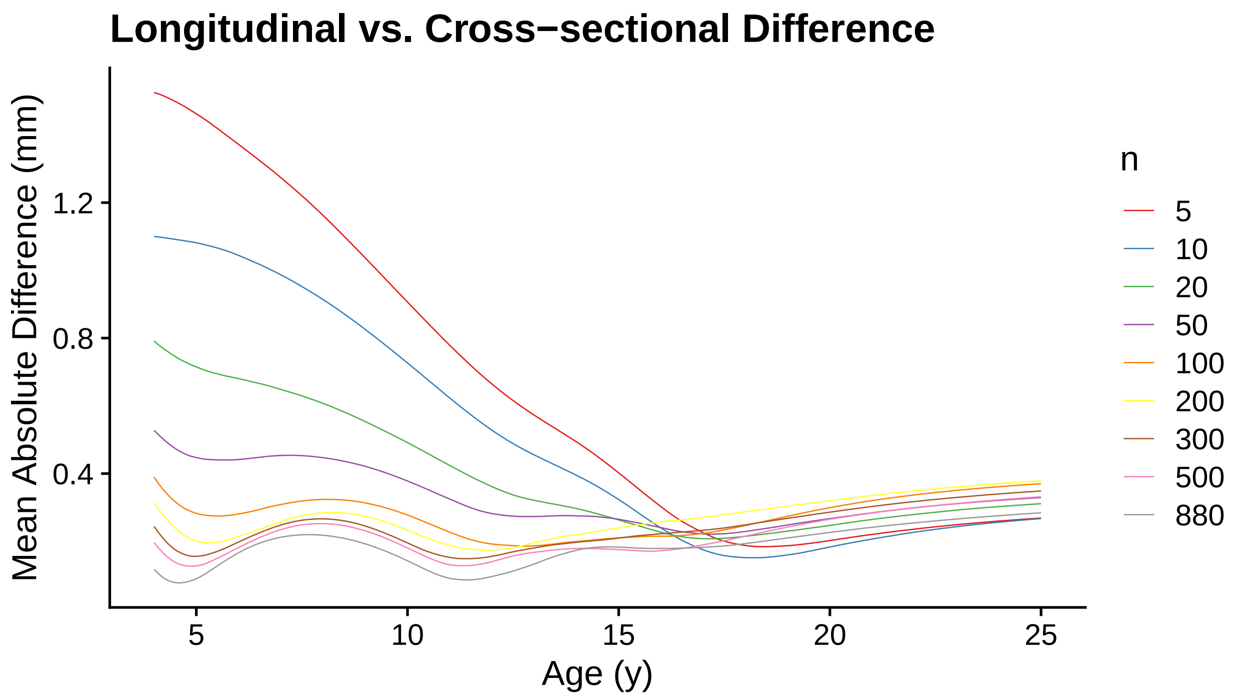
Longitudinal vs. Cross−sectional Difference
Mean Absolute Difference (mm)
1.2
0.8
0.4
5
10
15
20
25
Age (y)
n
5
10
20
50
100
200
300
500
880

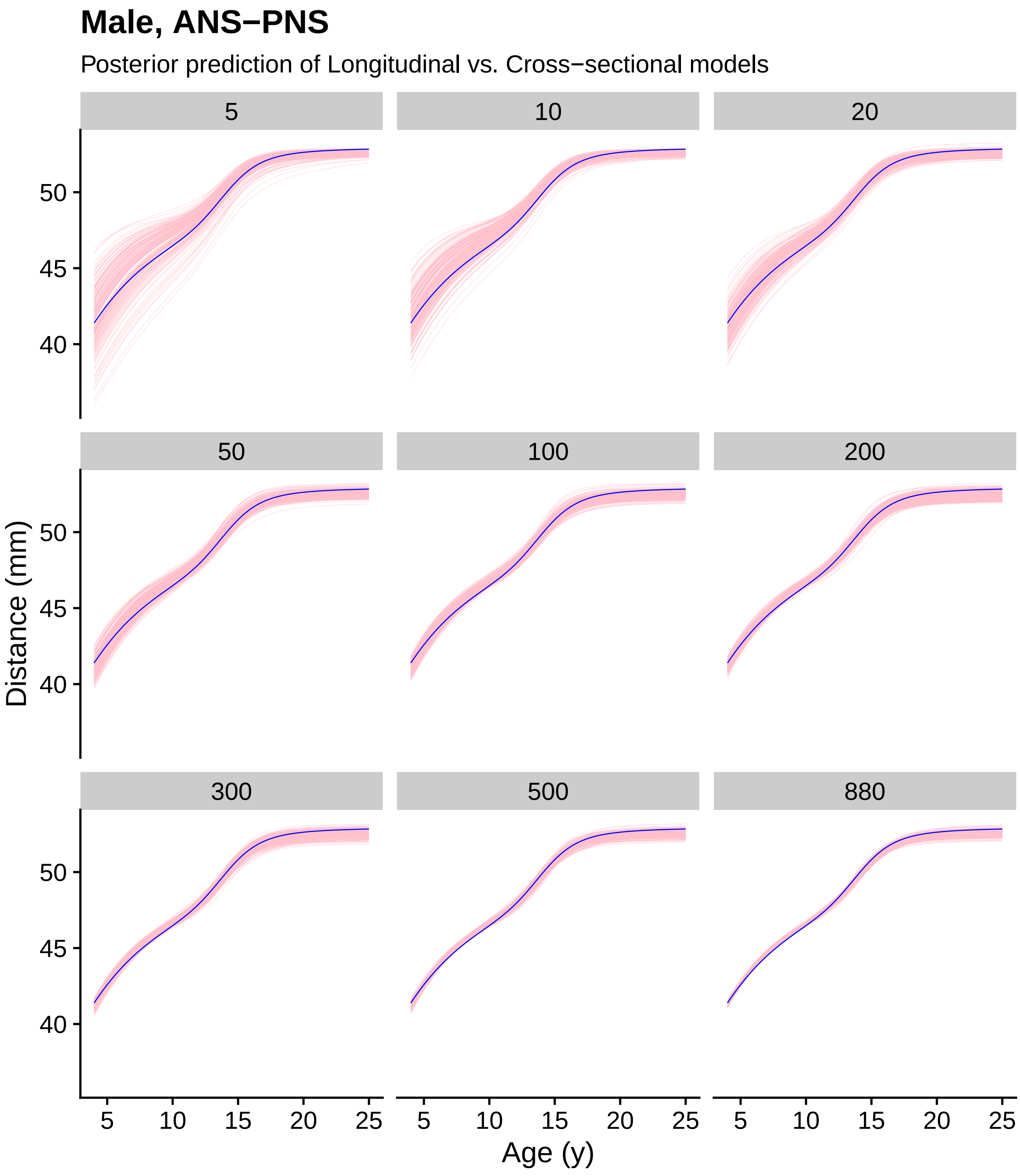
Male, ANS−PNS
Posterior prediction of Longitudinal vs. Cross−sectional models
5
10
20
50
100
200
300
500
880
Distance (mm)
Age (y)
40
45
50
5
10
15
20
25

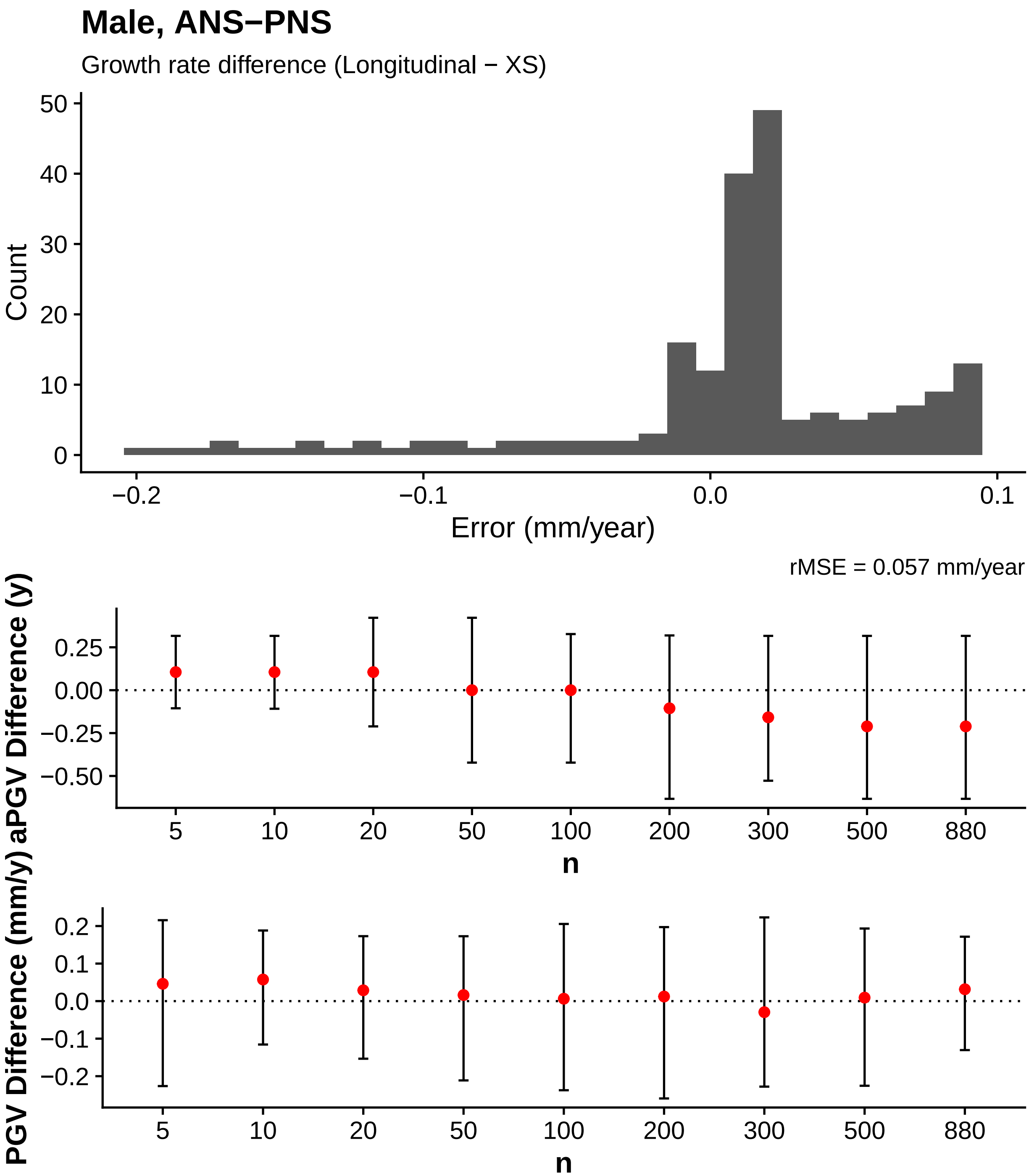
Male, ANS−PNS
Growth rate difference (Longitudinal − XS)
Count
Error (mm/year)
rMSE = 0.057 mm/year
PGV Difference (mm/y) aPGV Difference (y)
n
n

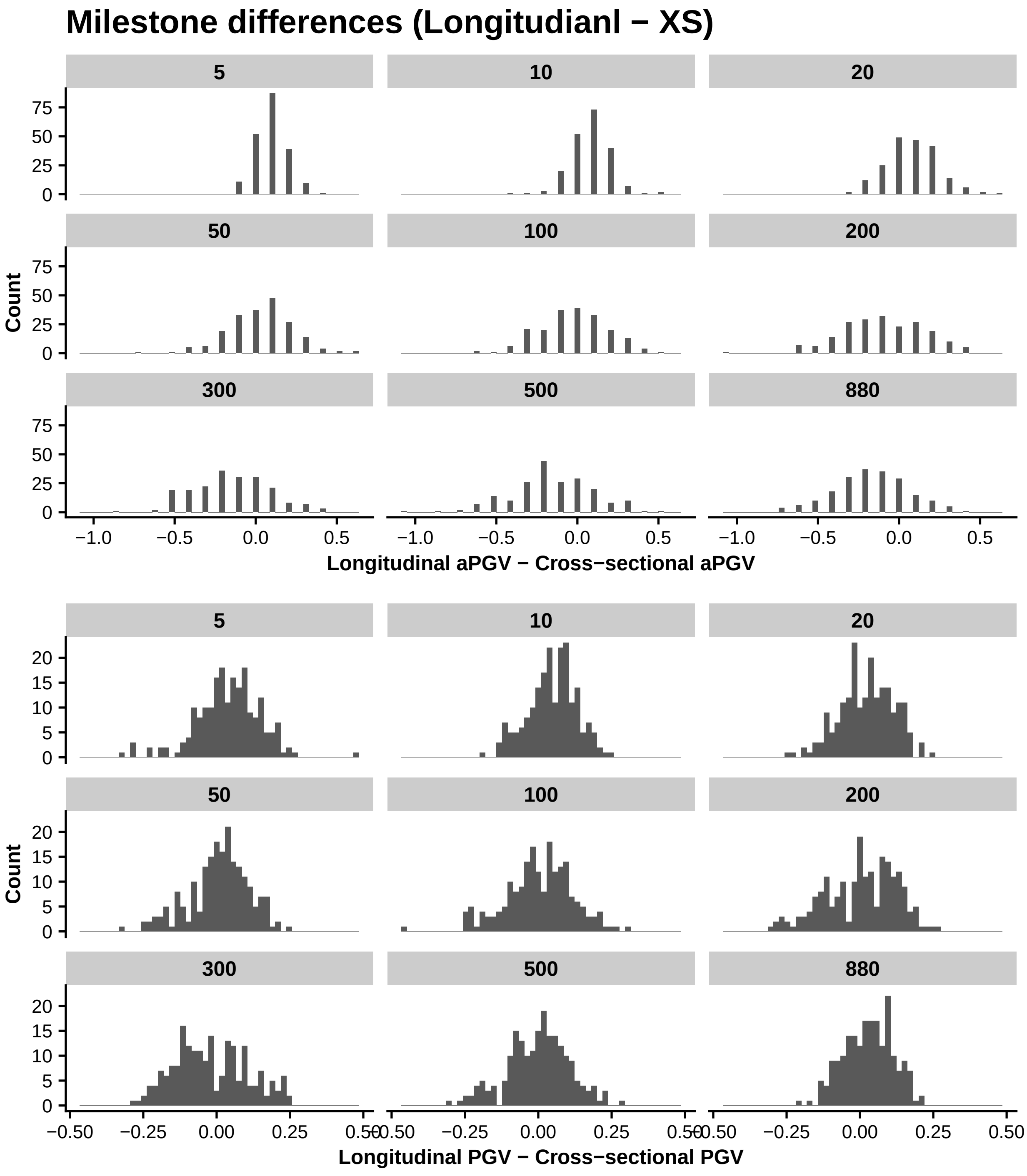
Milestone differences (Longitudianl − XS)
5
10
20
50
100
200
300
500
880
Count
0
25
50
75
−1.0
−0.5
0.0
0.5
Longitudinal aPGV − Cross−sectional aPGV
0
5
10
15
20
−0.50
−0.25
0.00
0.25
0.50
Longitudinal PGV − Cross−sectional PGV

16 Male, Articulare-Pogonion

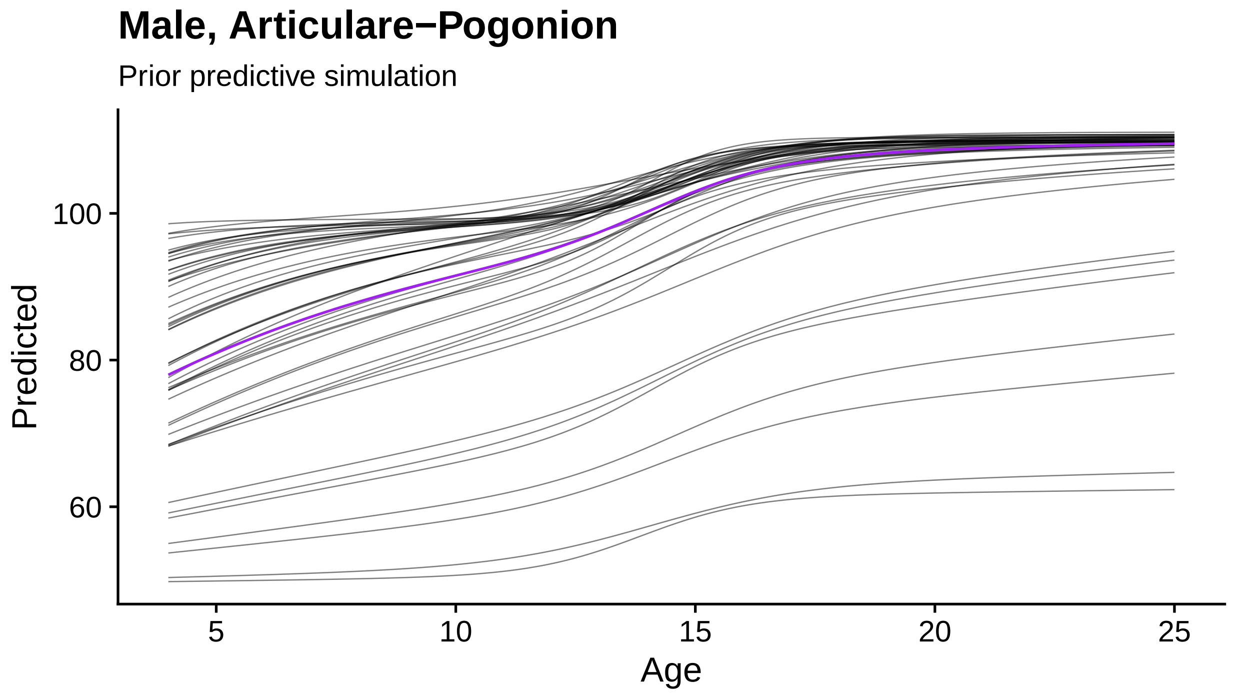

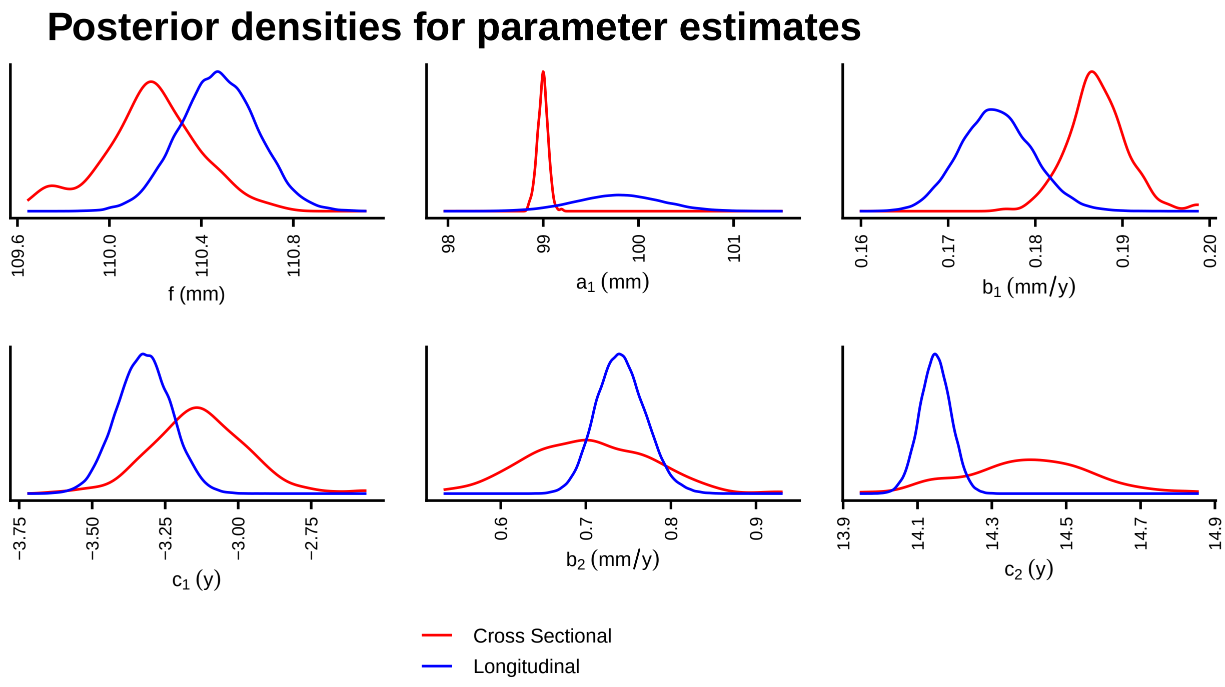

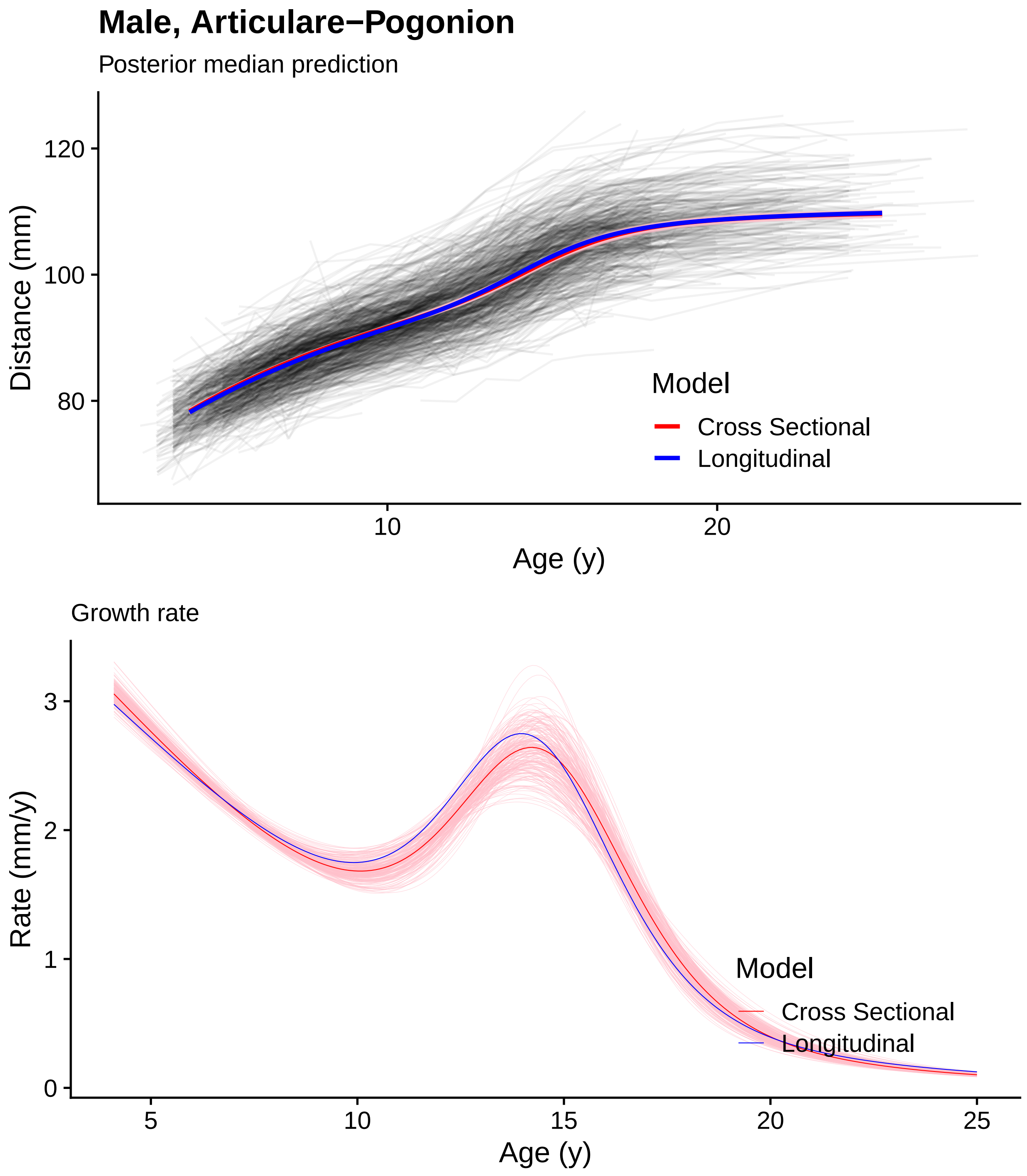
Male, Articulare−Pogonion
Posterior median prediction
120
100
80
Distance (mm)
10
20
Age (y)
Model
Cross Sectional
Longitudinal
Growth rate
3
2
1
0
Rate (mm/y)
5
10
15
20
25
Age (y)
Model
Cross Sectional
Longitudinal

Table 29: Longitudinal Model Summary

Parameter	Mean	Median	Std. Dev.	MAD	5%	95%	$\hat{r}$	Bulk ESS	Tail ESS
f	110.47	110.47	0.170	0.170	110.19	110.75	1	5438	14000
a1	99.79	99.79	0.448	0.447	99.05	100.52	1	26102	28742
b1	0.18	0.18	0.004	0.004	0.17	0.18	1	14304	24909
c1	-3.32	-3.32	0.093	0.094	-3.48	-3.17	1	33922	31734
b2	0.74	0.74	0.029	0.029	0.70	0.79	1	26492	30473
c2	14.15	14.15	0.042	0.042	14.08	14.22	1	59638	31641
sigma	1.96	1.96	0.016	0.016	1.93	1.99	1	65755	29466
sigma_ID	3.89	3.89	0.091	0.091	3.74	4.04	1	79655	29743

Table 30: Median Coefficients

Model	f	a_1	b_1	c_1	b_2	c_2	σ	σ_{ID}
Longitudinal	110.47	99.79	0.18	-3.32	0.74	14.15	1.96	3.89
Cross Sectional	110.18	99.00	0.19	-3.14	0.70	14.40	4.32	NA

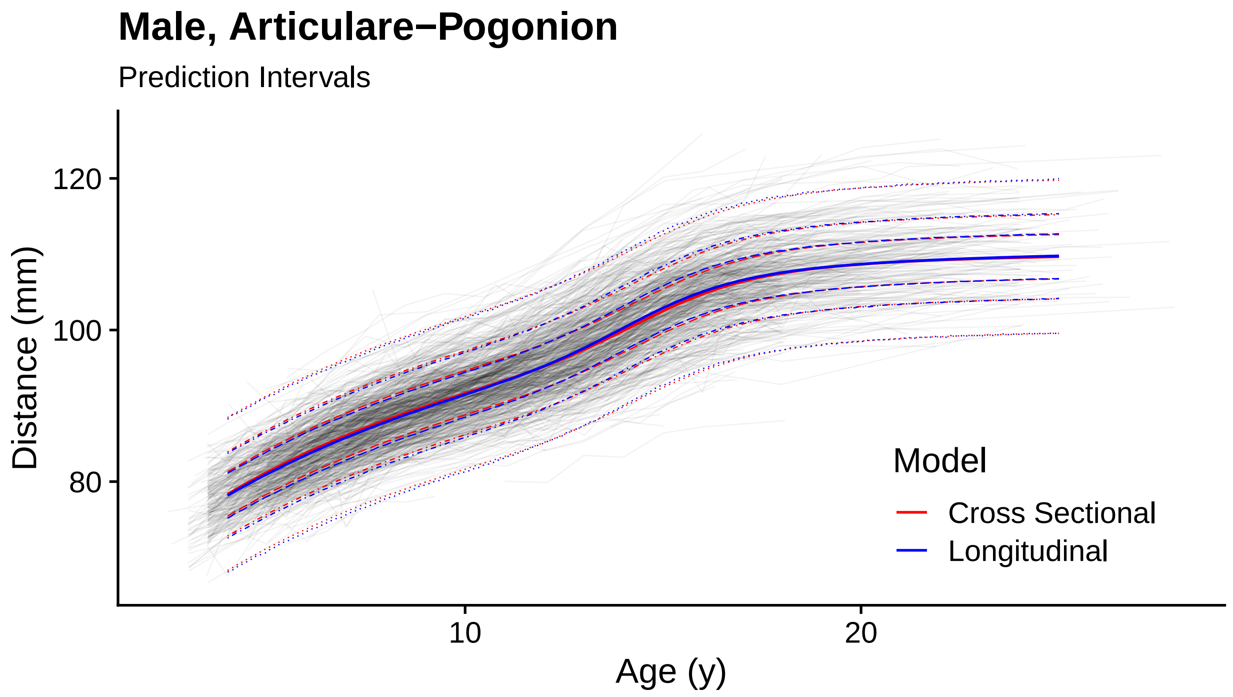
Male, Articulare−Pogonion
Prediction Intervals
Distance (mm)
120
100
80
10
20
Age (y)
Model
Cross Sectional
Longitudinal

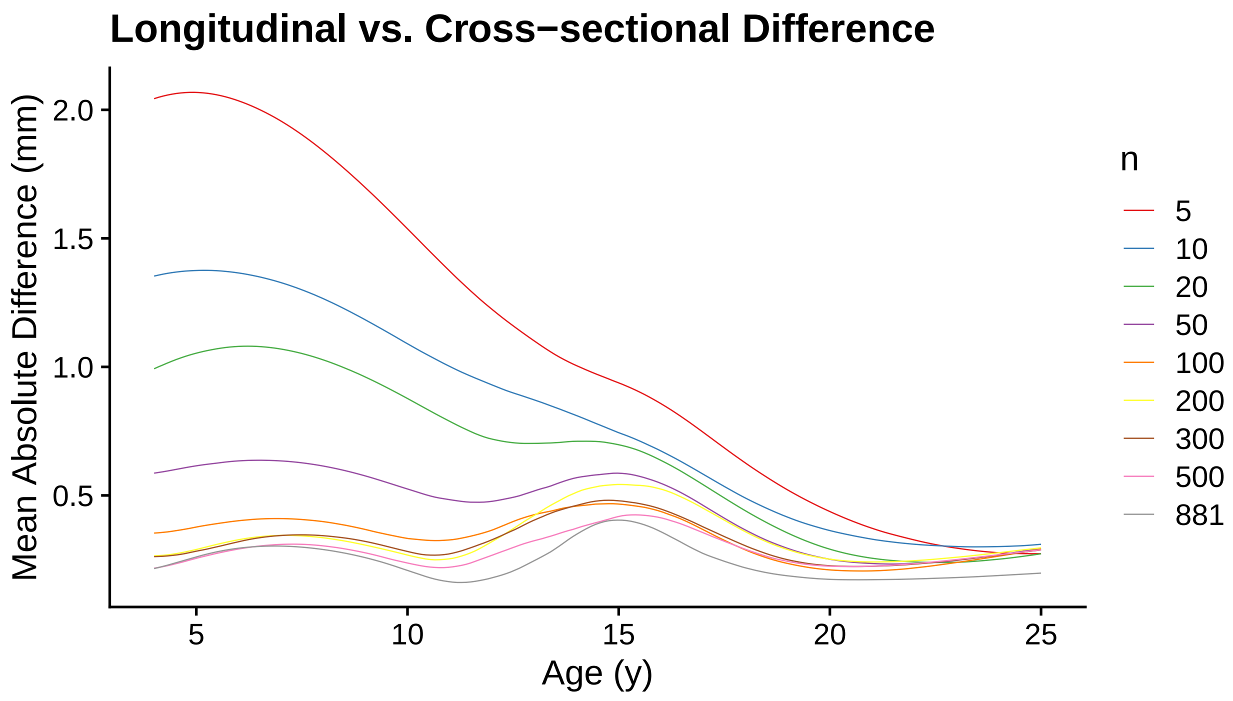
Longitudinal vs. Cross−sectional Difference
Mean Absolute Difference (mm)
2.0
1.5
1.0
0.5
5
10
15
20
25
Age (y)
n
5
10
20
50
100
200
300
500
881

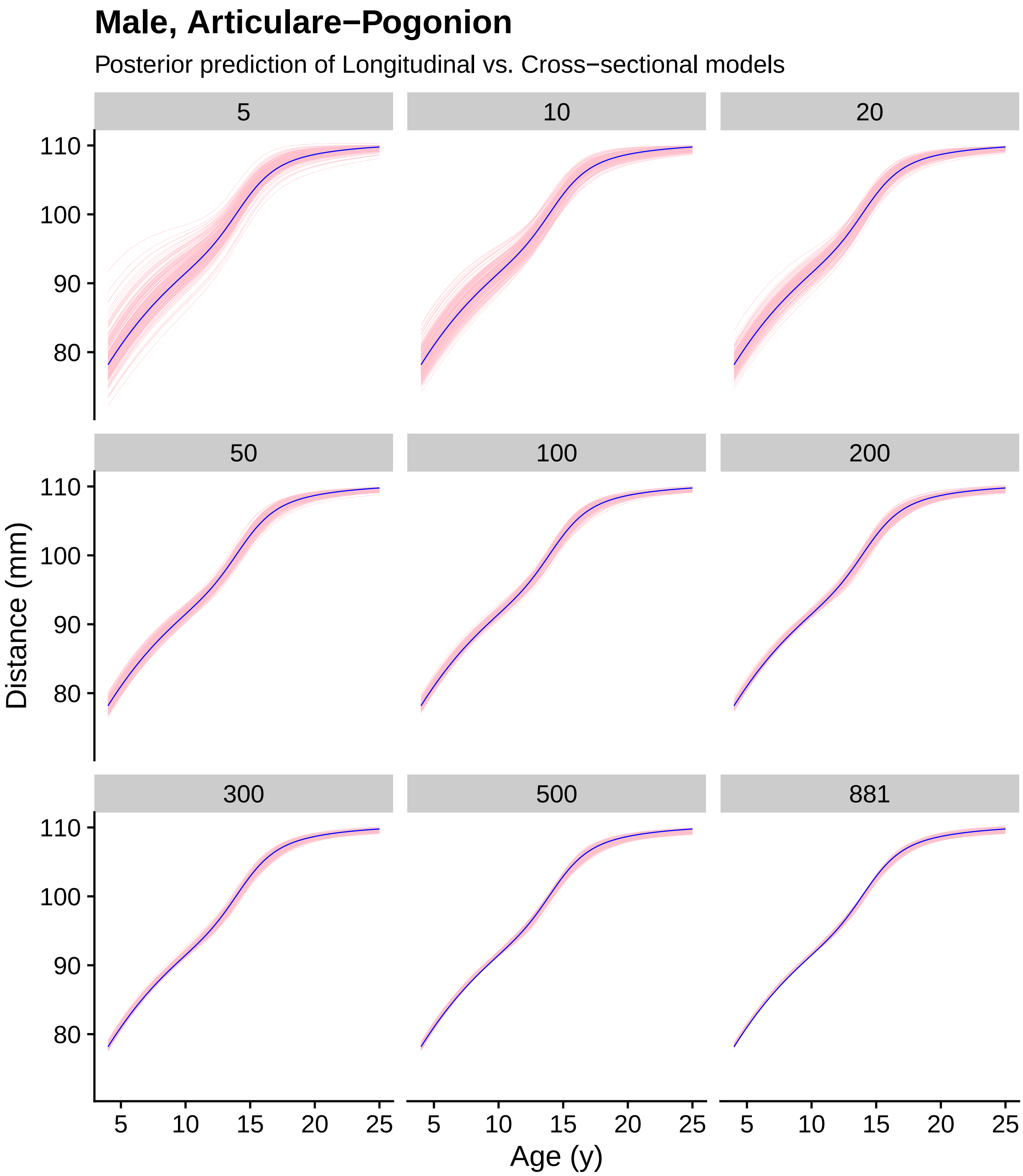

Male, Articulare−Pogonion
Posterior prediction of Longitudinal vs. Cross−sectional models
5
10
20
50
100
200
300
500
881
110
100
90
80
Distance (mm)
5
10
15
20
25
Age (y)

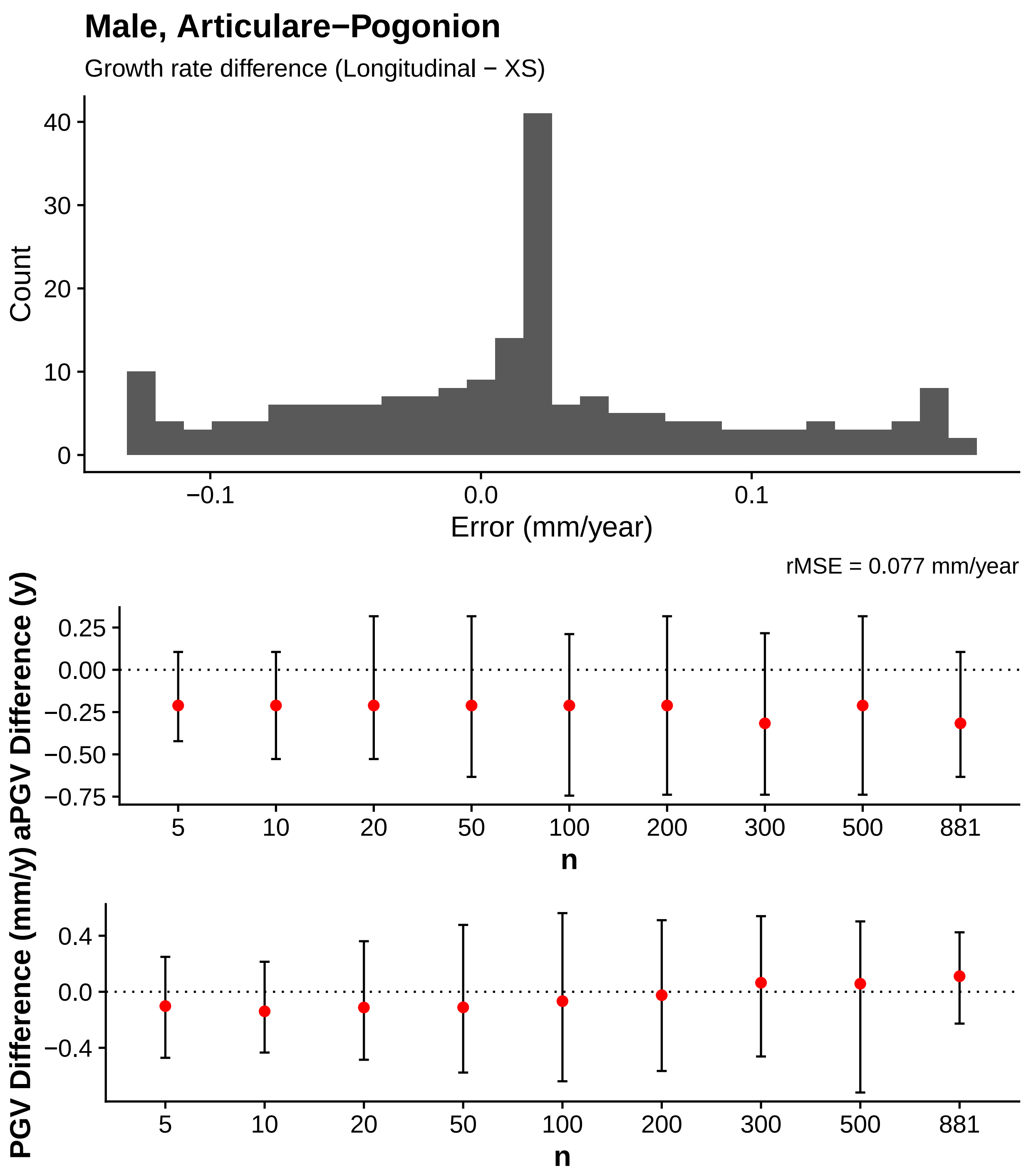
Male, Articulare−Pogonion
Growth rate difference (Longitudinal − XS)
Count
Error (mm/year)
rMSE = 0.077 mm/year
PGV Difference (mm/y) aPGV Difference (y)
n
n

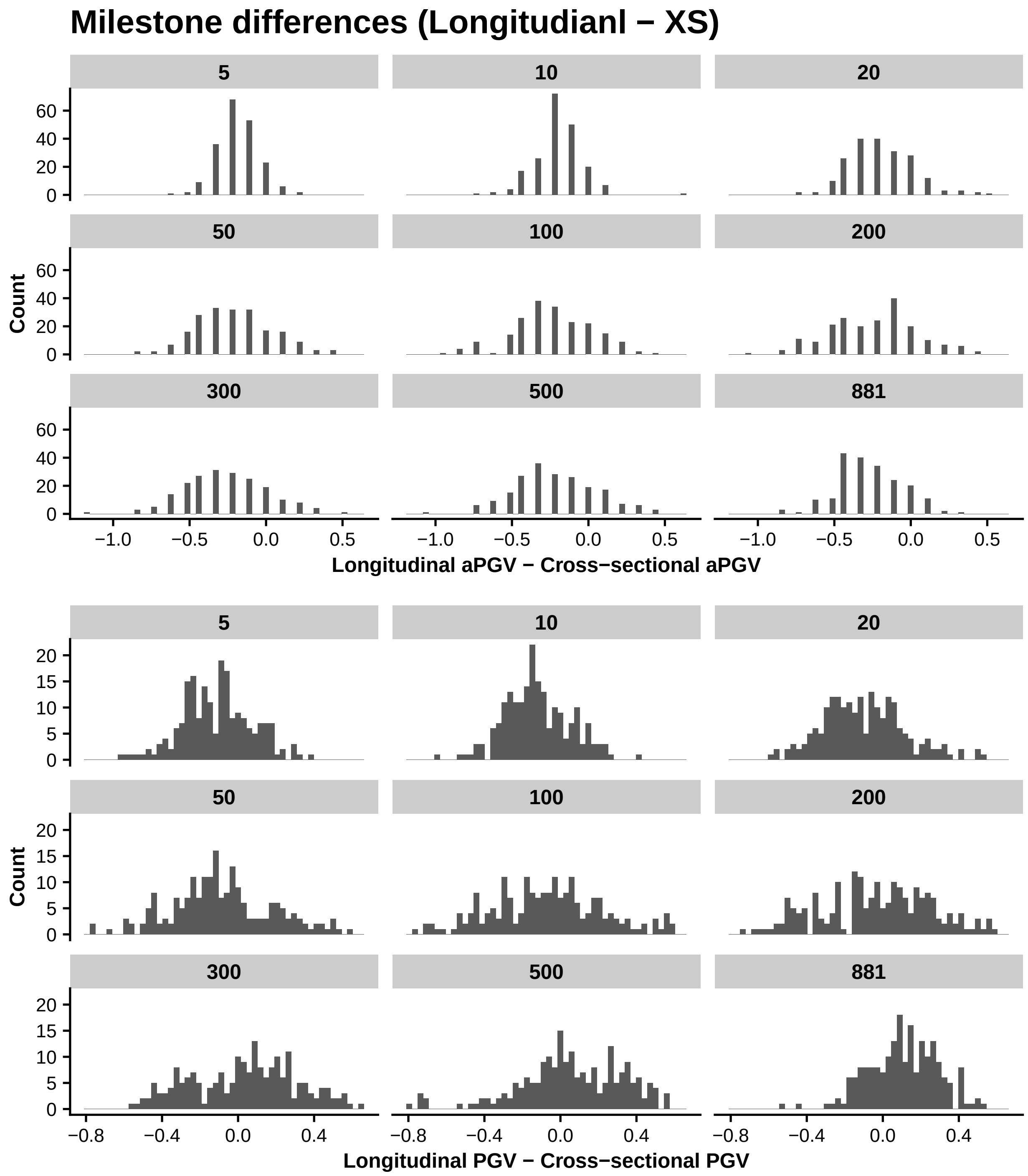
Milestone differences (Longitudianl − XS)
5
10
20
50
100
200
300
500
881
Count
Longitudinal aPGV − Cross−sectional aPGV
Longitudinal PGV − Cross−sectional PGV

17 Male, Condylion-Gonion

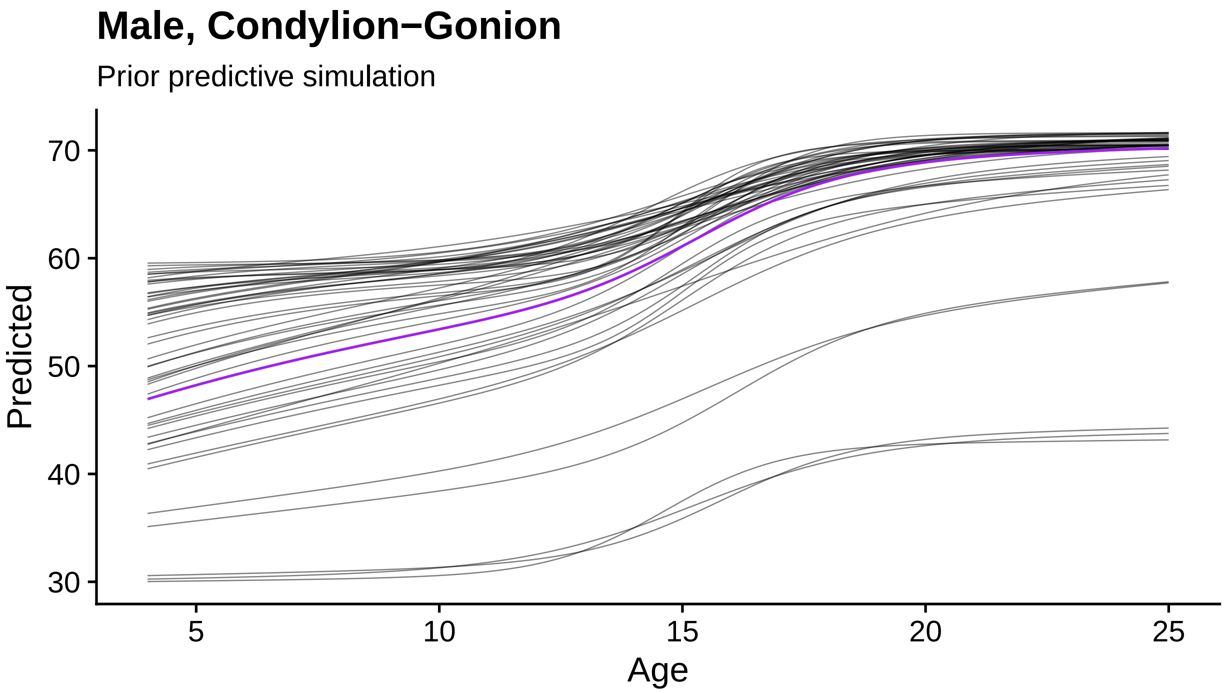

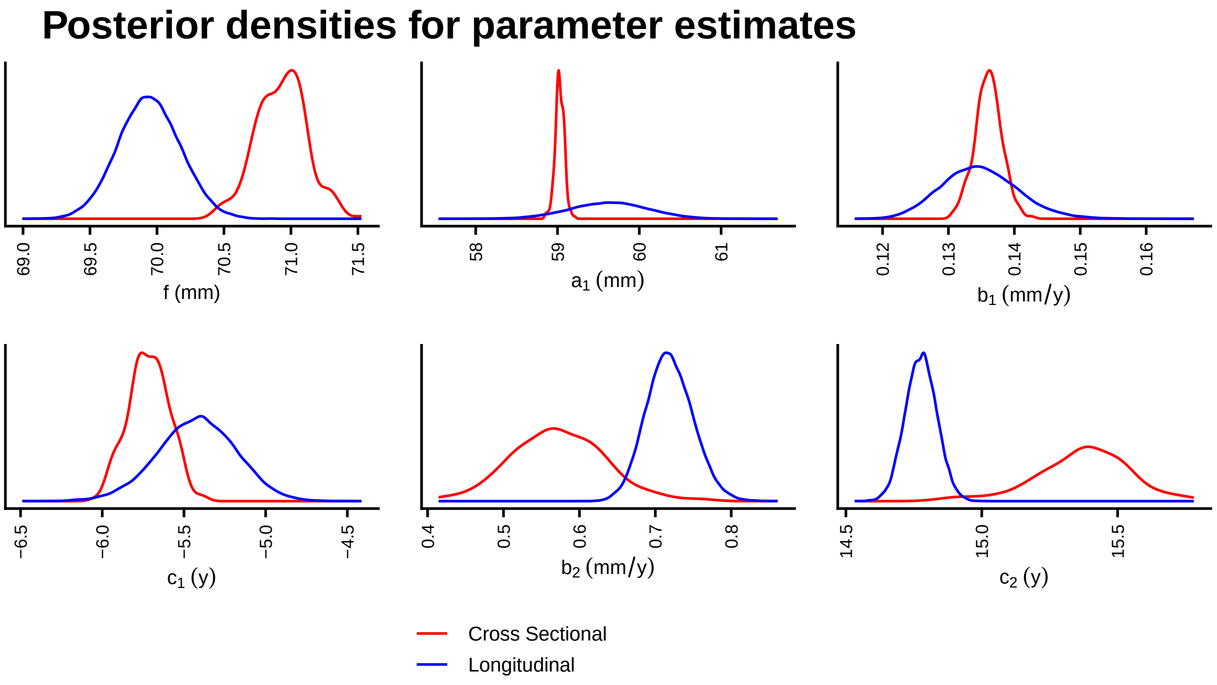

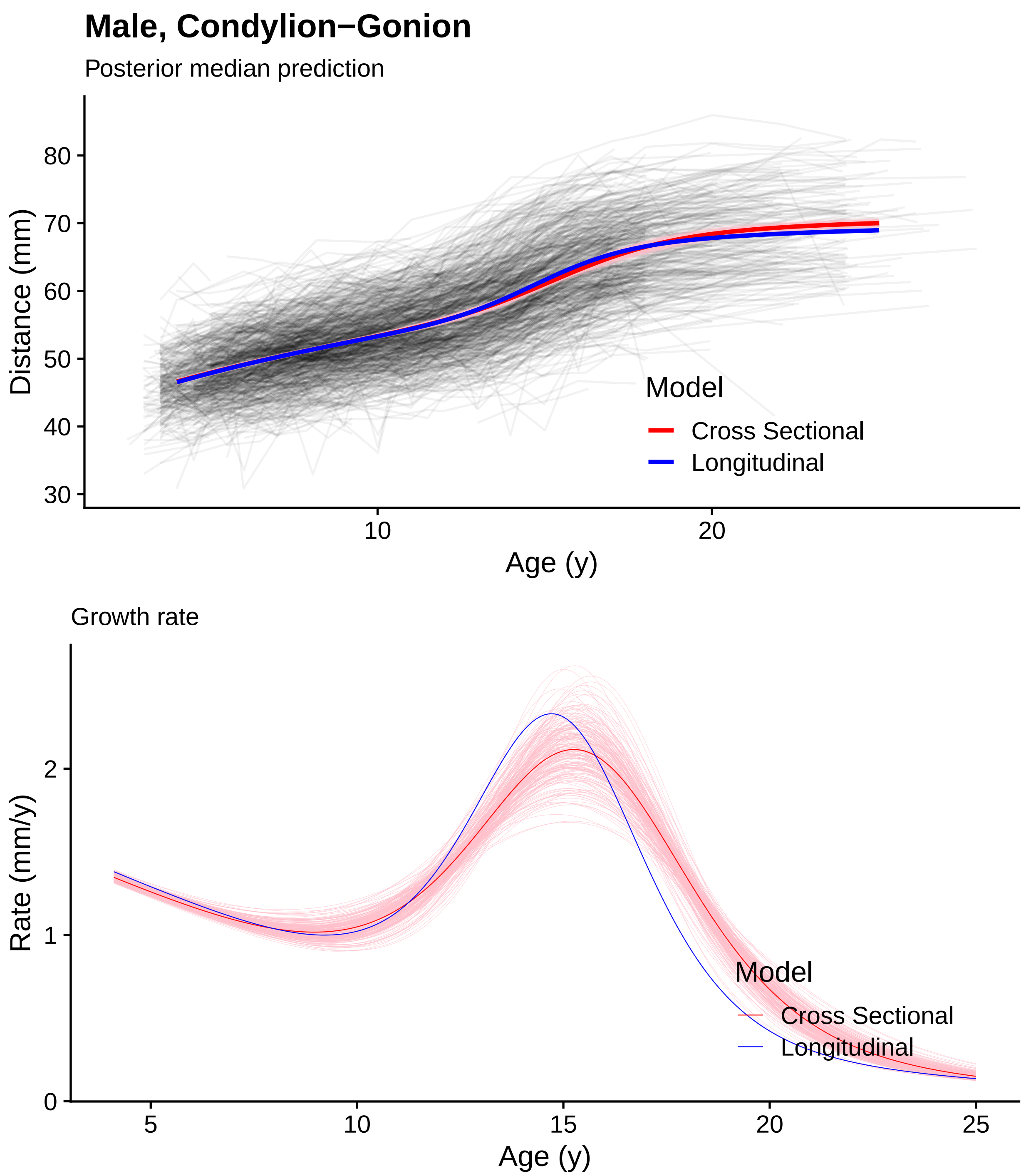
Male, Condylion−Gonion
Posterior median prediction
Distance (mm)
80
70
60
50
40
30
10
20
Age (y)
Model
Cross Sectional
Longitudinal
Growth rate
Rate (mm/y)
2
1
0
5
10
15
20
25
Age (y)
Model
Cross Sectional
Longitudinal

Table 31: Longitudinal Model Summary

Parameter	Mean	Median	Std. Dev.	MAD	5%	95%	$\hat{r}$	Bulk ESS	Tail ESS
f	69.94	69.94	0.232	0.234	69.56	70.33	1	10070	19341
a1	59.63	59.63	0.473	0.475	58.84	60.40	1	22458	26550
b1	0.13	0.13	0.006	0.006	0.13	0.14	1	9732	19146
c1	-5.42	-5.42	0.241	0.239	-5.83	-5.04	1	28221	28984
b2	0.72	0.72	0.031	0.031	0.67	0.77	1	27264	29306
c2	14.78	14.78	0.058	0.058	14.68	14.87	1	45664	31498
sigma	2.32	2.32	0.019	0.019	2.29	2.35	1	58799	29840
sigma_ID	4.62	4.62	0.108	0.108	4.45	4.81	1	69562	31147

Table 32: Median Coefficients

Model	f	a_1	b_1	c_1	b_2	c_2	σ	σ_{ID}
Longitudinal	69.94	59.63	0.13	-5.42	0.72	14.78	2.32	4.62
Cross Sectional	70.95	59.02	0.14	-5.72	0.57	15.39	5.14	NA

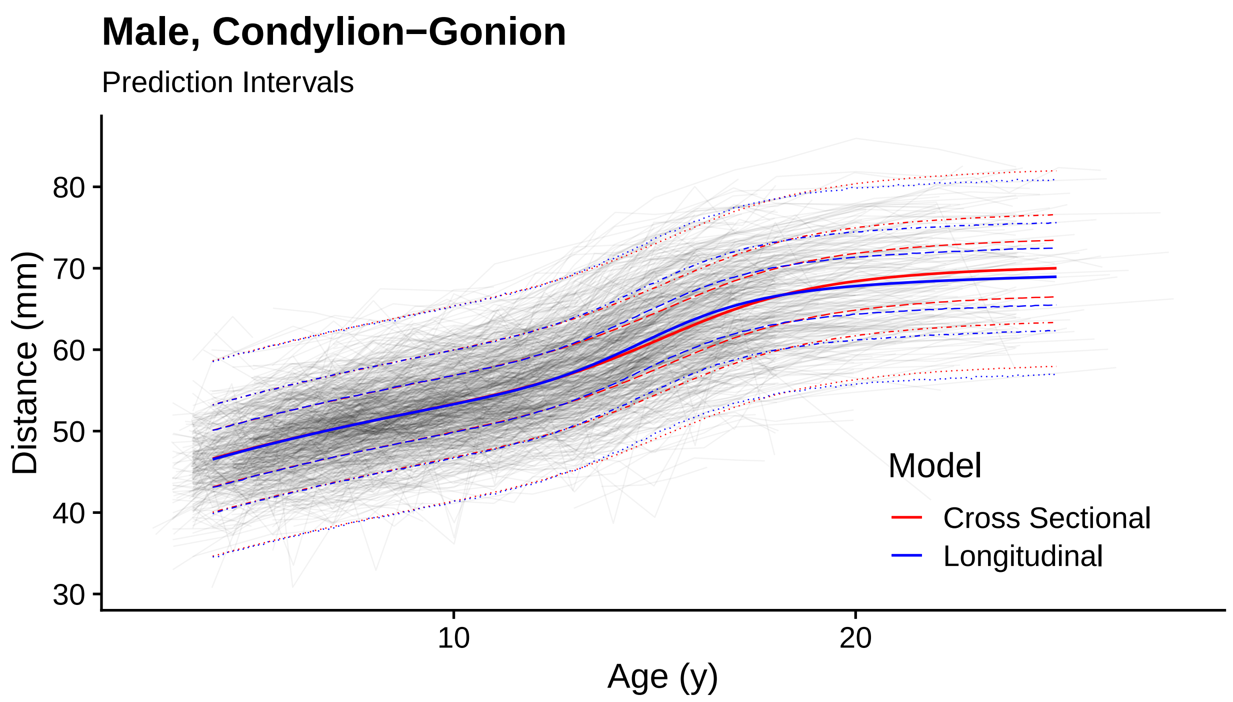

Male, Condylion−Gonion
Prediction Intervals
Distance (mm)
30
40
50
60
70
80
10
20
Age (y)
Model
Cross Sectional
Longitudinal

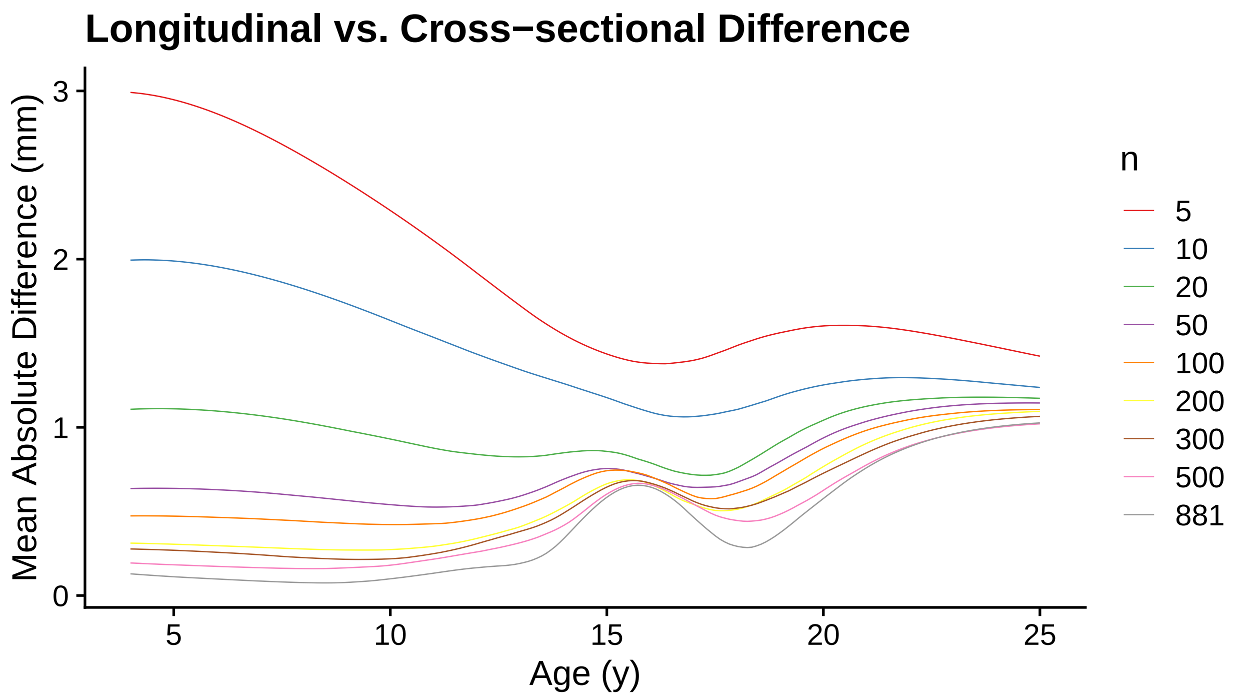

Longitudinal vs. Cross−sectional Difference
Mean Absolute Difference (mm)
0
1
2
3
5
10
15
20
25
Age (y)
n
5
10
20
50
100
200
300
500
881

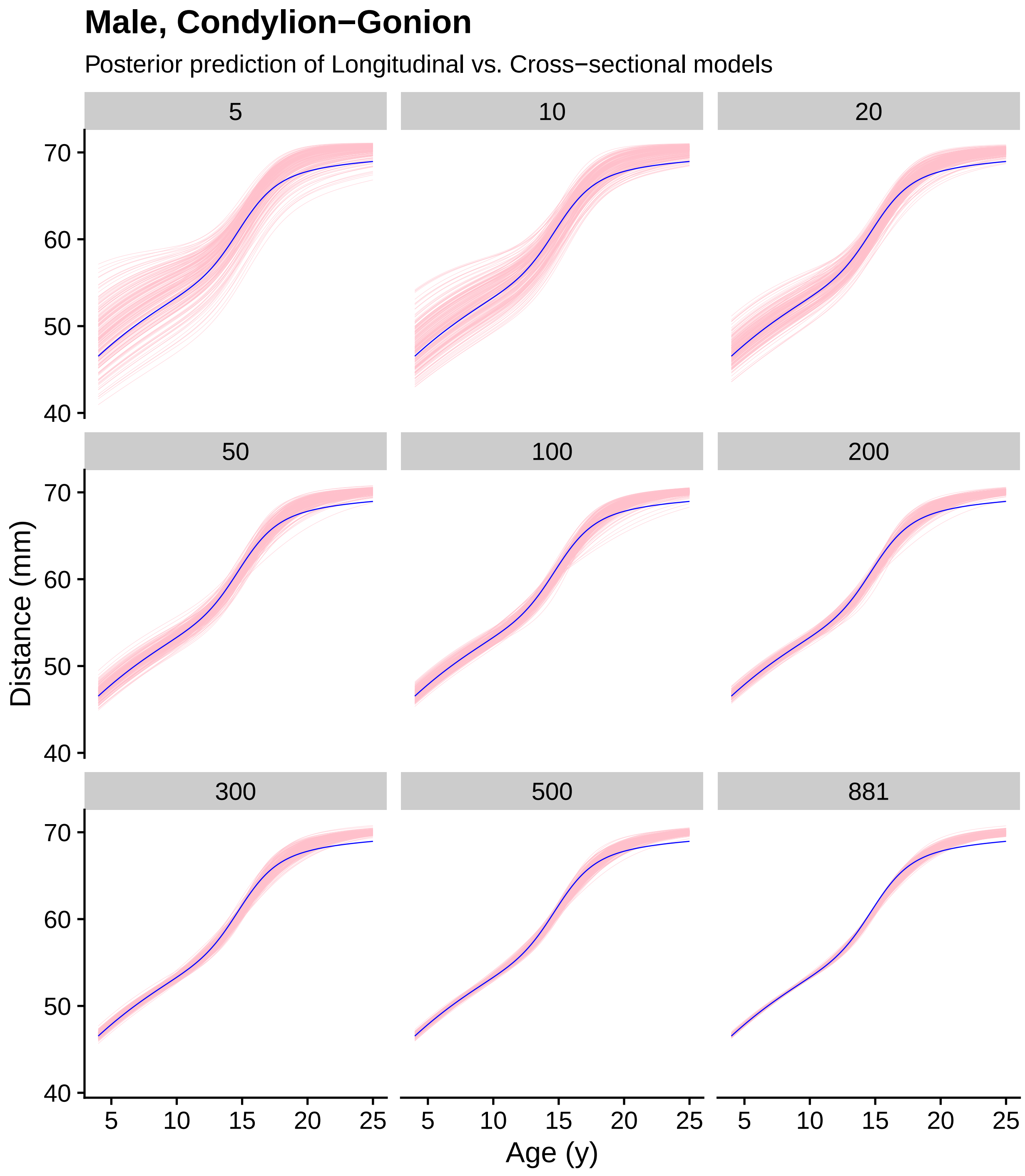
Male, Condylion−Gonion
Posterior prediction of Longitudinal vs. Cross−sectional models
5
10
20
50
100
200
300
500
881
Distance (mm)
Age (y)
70
60
50
40
5
10
15
20
25

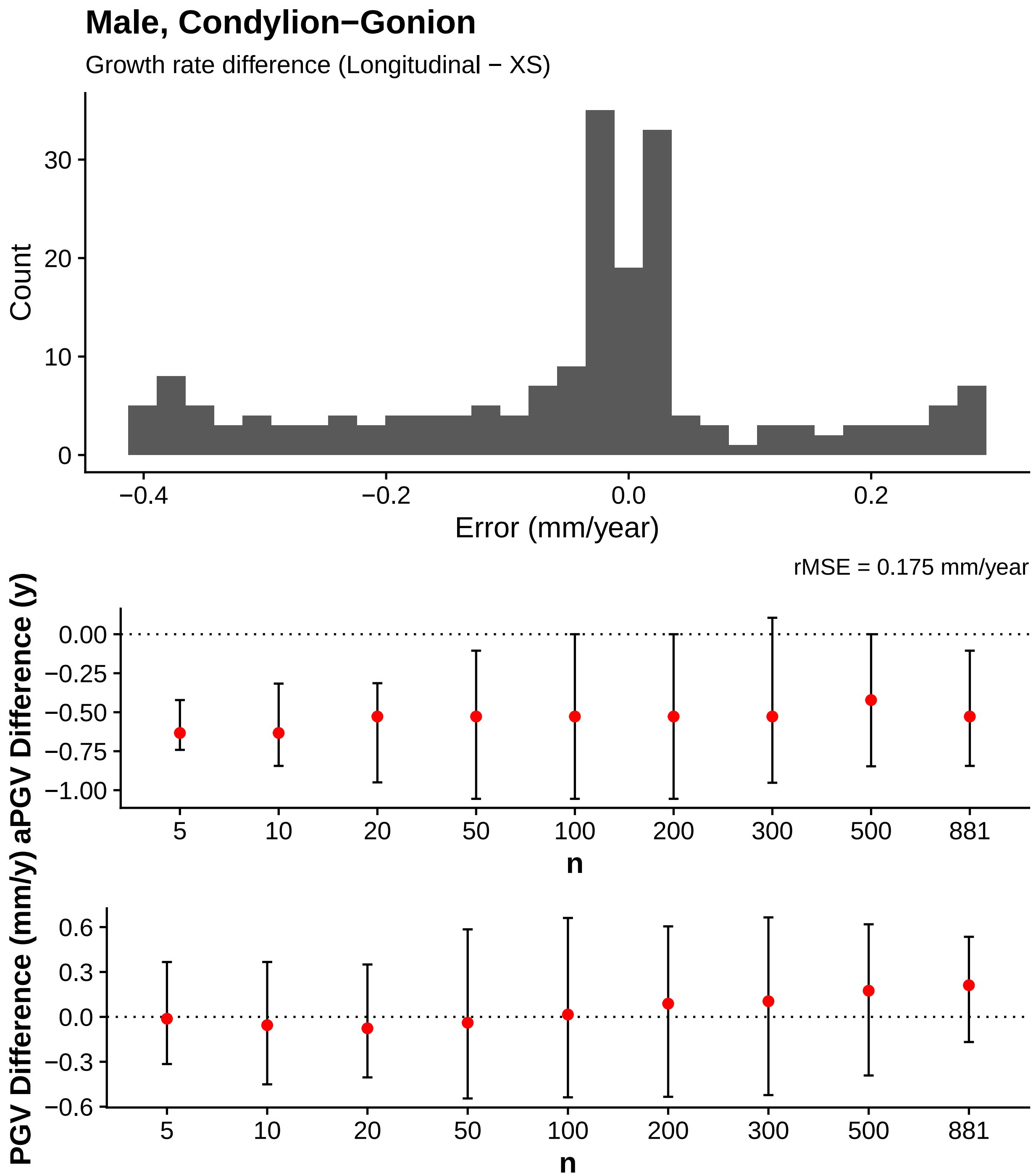

Male, Condylion−Gonion
Growth rate difference (Longitudinal − XS)
Count
30
20
10
0
−0.4
−0.2
0.0
0.2
Error (mm/year)
rMSE = 0.175 mm/year
PGV Difference (mm/y) aPGV Difference (y)
0.00
−0.25
−0.50
−0.75
−1.00
5
10
20
50
100
200
300
500
881
n
0.6
0.3
0.0
−0.3
−0.6
5
10
20
50
100
200
300
500
881
n

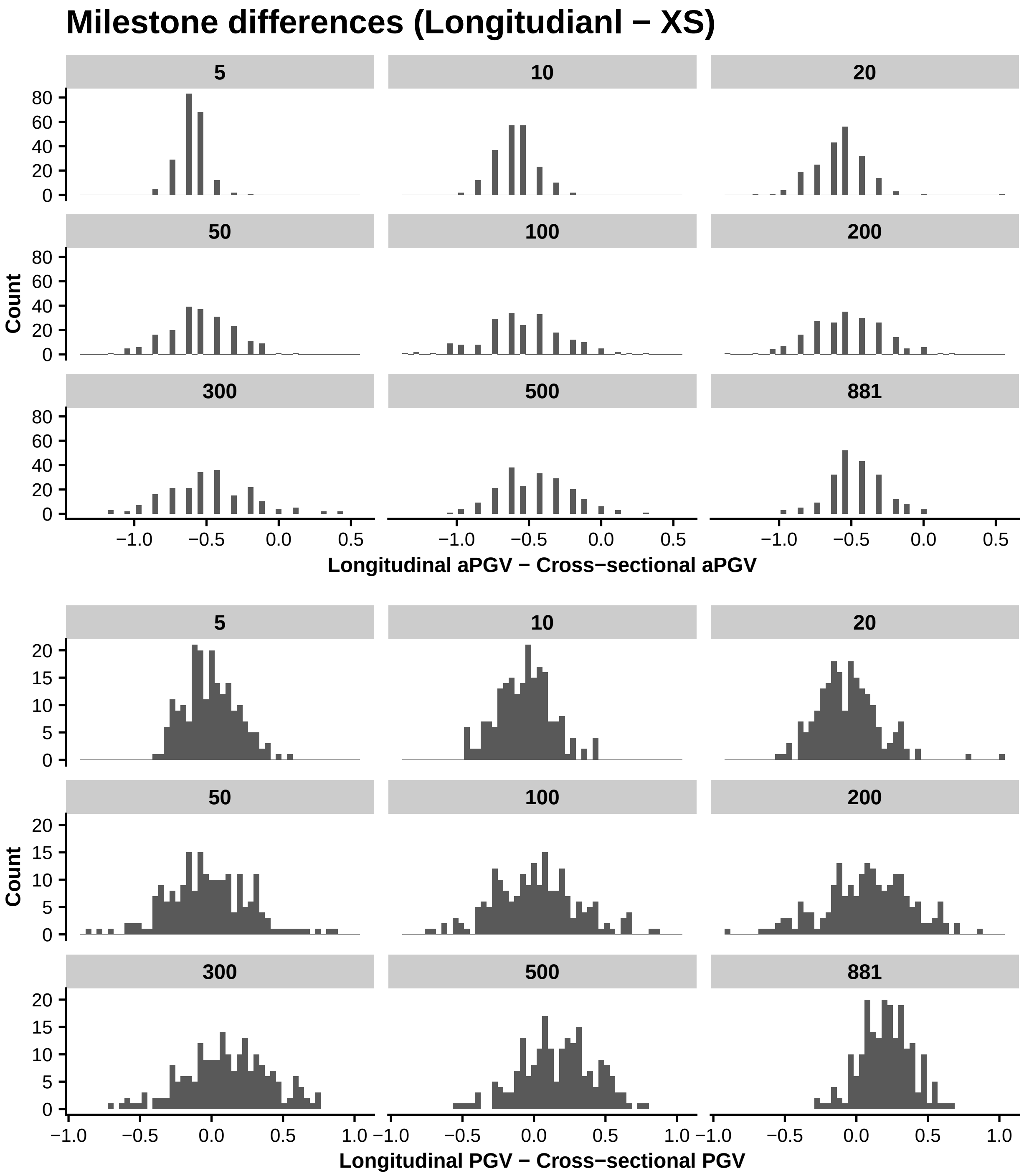

Milestone differences (Longitudianl − XS)
5
10
20
50
100
200
300
500
881
80
60
40
20
0
Count
−1.0
−0.5
0.0
0.5
Longitudinal aPGV − Cross−sectional aPGV
20
15
10
5
0
−1.0
−0.5
0.0
0.5
1.0
Longitudinal PGV − Cross−sectional PGV

18 Male, Condylion-Pogonion

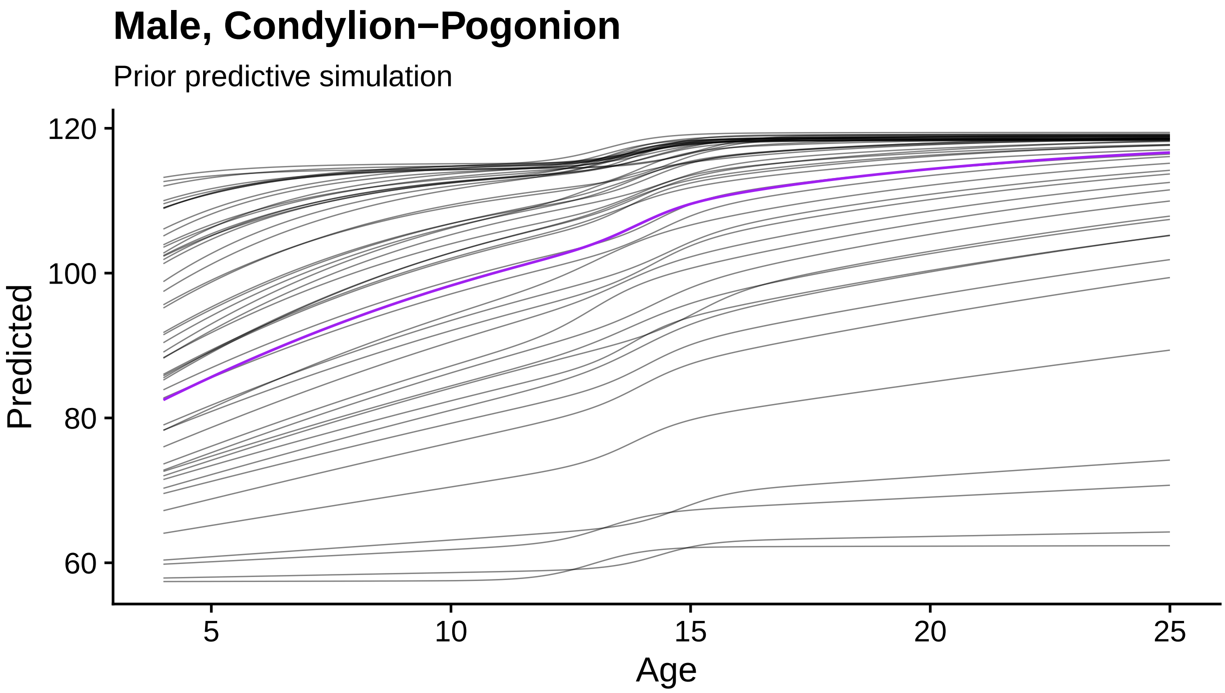

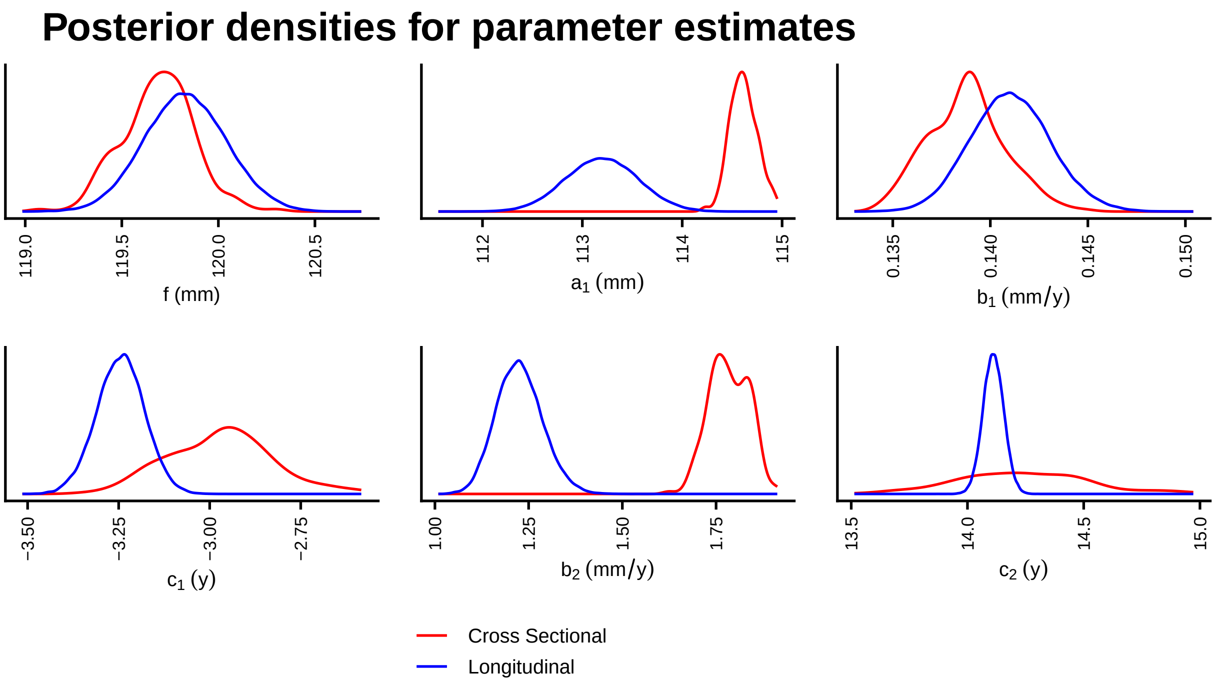

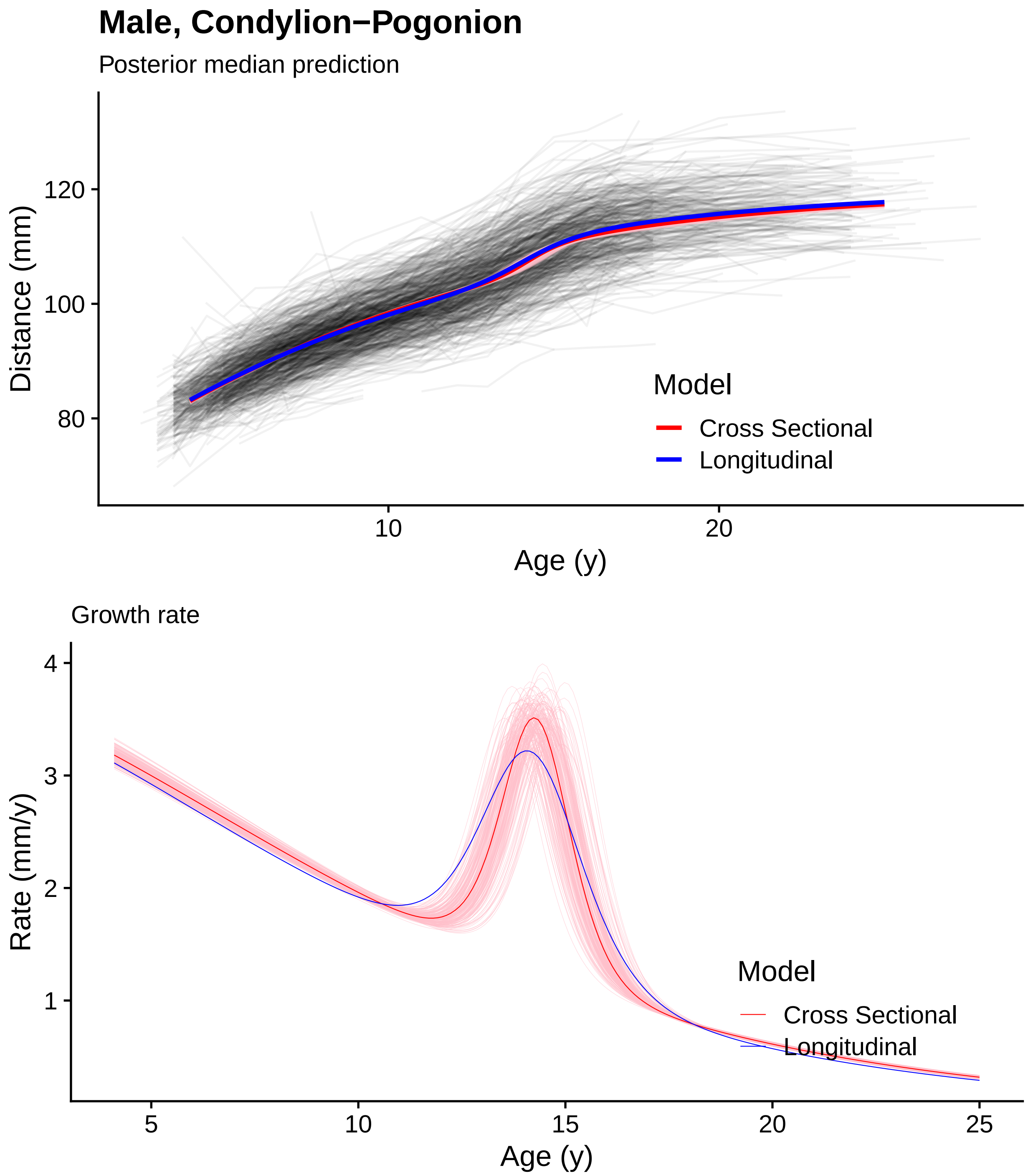
Male, Condylion−Pogonion
Posterior median prediction
Distance (mm)
120
100
80
10
20
Age (y)
Model
Cross Sectional
Longitudinal
Growth rate
Rate (mm/y)
4
3
2
1
5
10
15
20
25
Age (y)
Model
Cross Sectional
Longitudinal

Table 33: Longitudinal Model Summary

Parameter	Mean	Median	Std. Dev.	MAD	5%	95%	$\hat{r}$	Bulk ESS	Tail ESS
f	119.83	119.83	0.213	0.213	119.48	120.18	1	8985	21424
a1	113.20	113.20	0.361	0.365	112.60	113.79	1	22480	26952
b1	0.14	0.14	0.002	0.002	0.14	0.14	1	15124	23635
c1	-3.24	-3.24	0.066	0.066	-3.35	-3.14	1	30881	30745
b2	1.23	1.22	0.062	0.062	1.13	1.33	1	28950	30203
c2	14.11	14.11	0.045	0.045	14.04	14.18	1	53897	31683
sigma	2.06	2.06	0.017	0.017	2.04	2.09	1	52580	30339
sigma_ID	4.17	4.17	0.098	0.097	4.02	4.34	1	63497	30818

Table 34: Median Coefficients

Model	f	a_1	b_1	c_1	b_2	c_2	σ	σ_{ID}
Longitudinal	119.83	113.2	0.14	-3.24	1.22	14.11	2.06	4.17
Cross Sectional	119.70	114.6	0.14	-2.97	1.78	14.23	4.64	NA

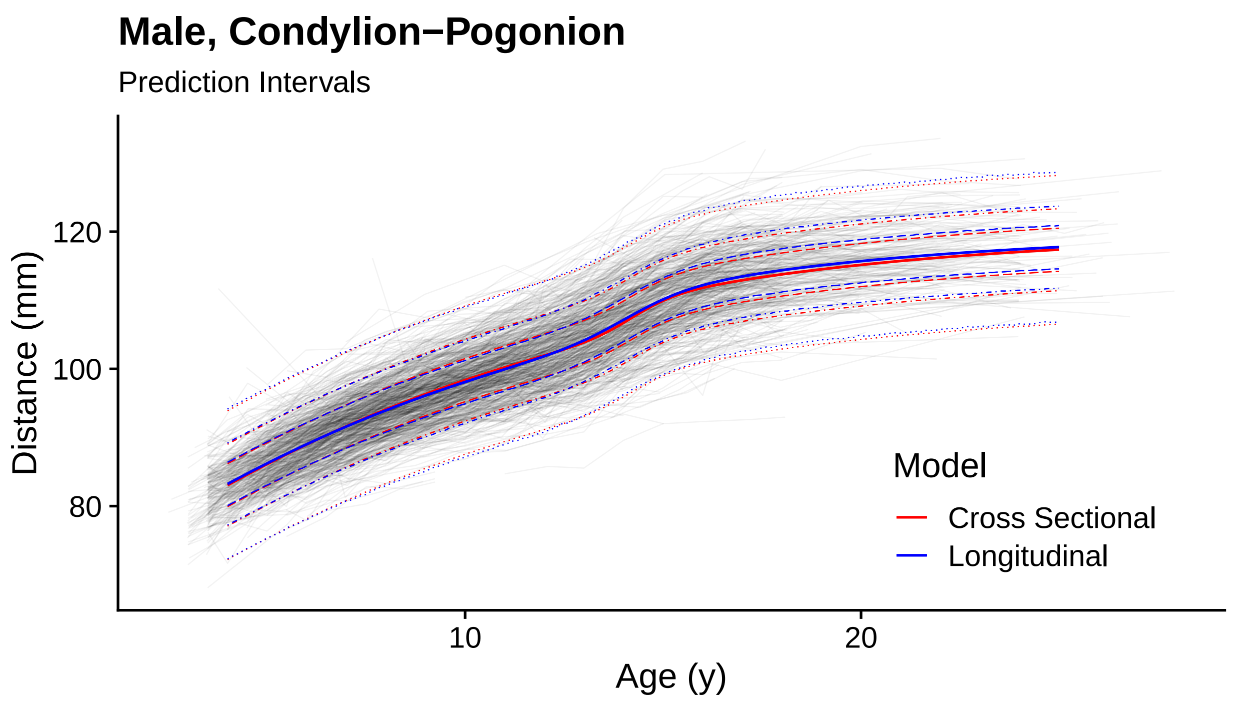
Male, Condylion−Pogonion
Prediction Intervals
Distance (mm)
120
100
80
10
20
Age (y)
Model
Cross Sectional
Longitudinal

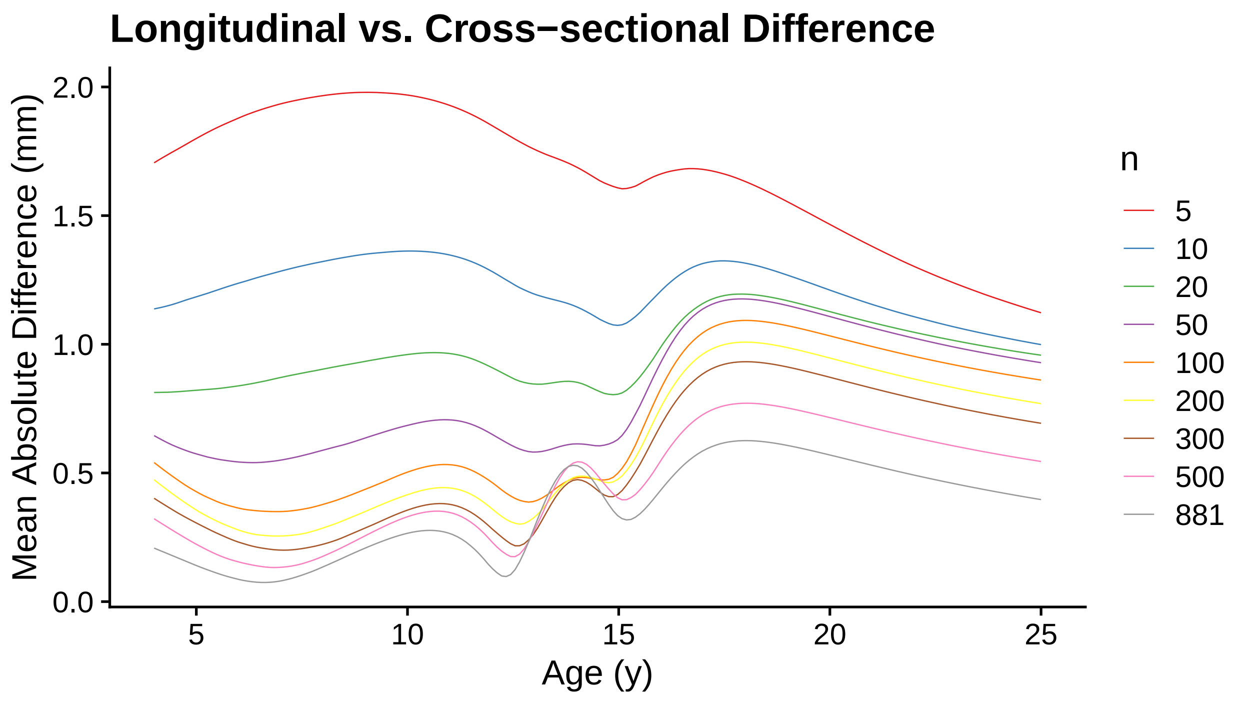
Longitudinal vs. Cross−sectional Difference
Mean Absolute Difference (mm)
2.0
1.5
1.0
0.5
0.0
5
10
15
20
25
Age (y)
n
5
10
20
50
100
200
300
500
881

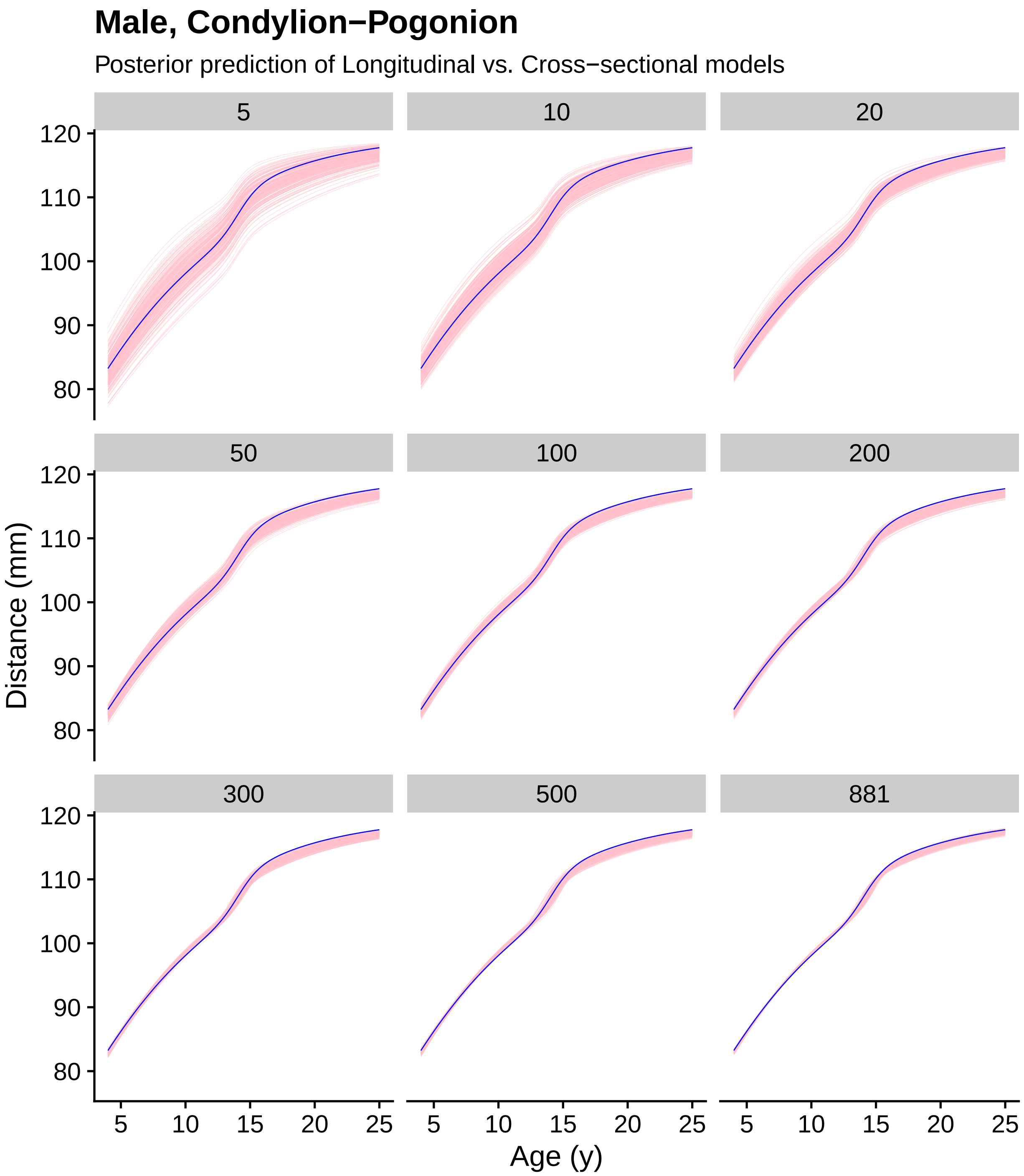

Male, Condylion−Pogonion
Posterior prediction of Longitudinal vs. Cross−sectional models
5
10
20
50
100
200
300
500
881
Distance (mm)
Age (y)
80
90
100
110
120
5
10
15
20
25

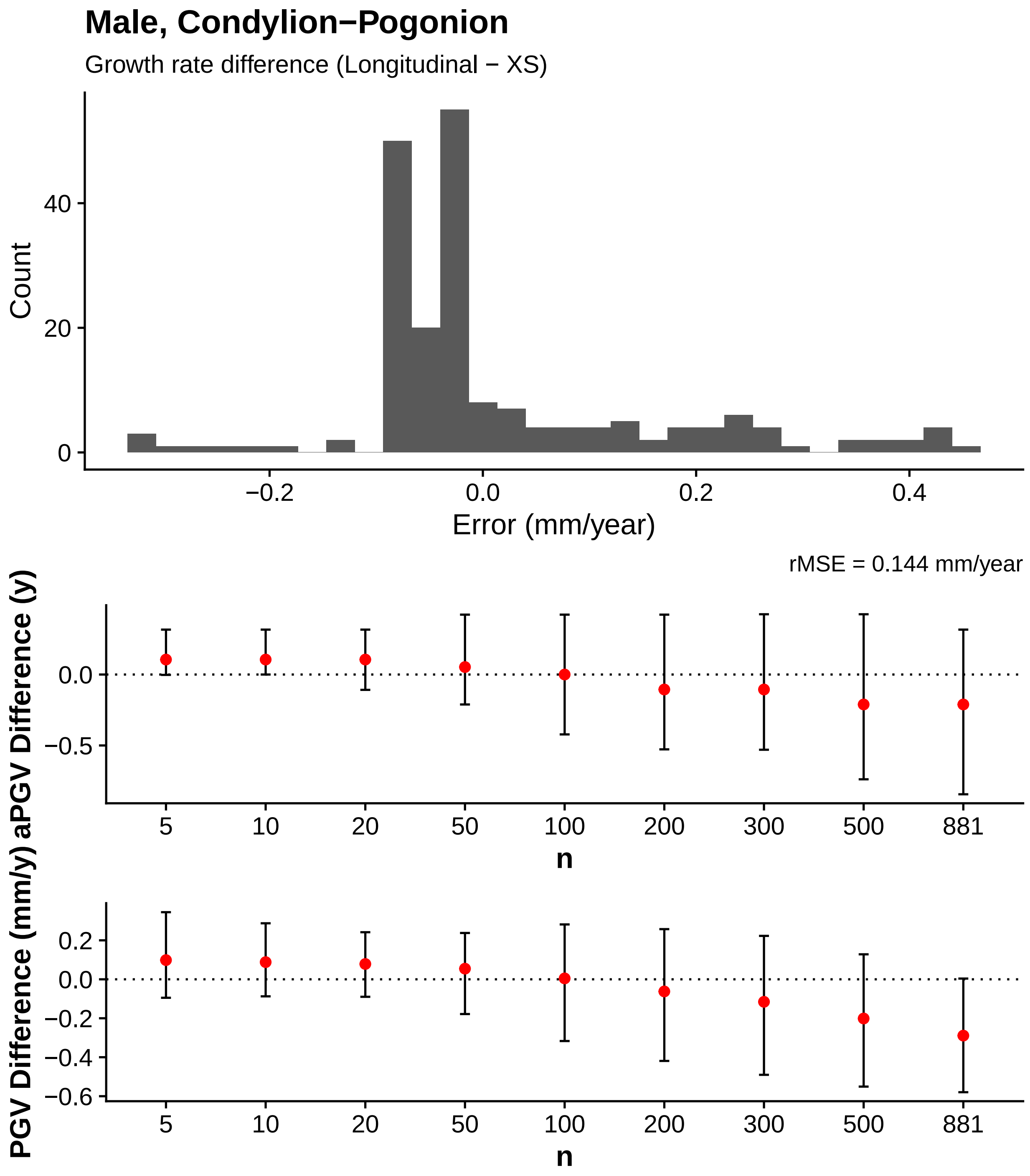
Male, Condylion−Pogonion
Growth rate difference (Longitudinal − XS)
Count
0
20
40
−0.2
0.0
0.2
0.4
Error (mm/year)
rMSE = 0.144 mm/year
PGV Difference (mm/y) aPGV Difference (y)
0.0
−0.5
5
10
20
50
100
200
300
500
881
n
0.2
0.0
−0.2
−0.4
−0.6
5
10
20
50
100
200
300
500
881
n

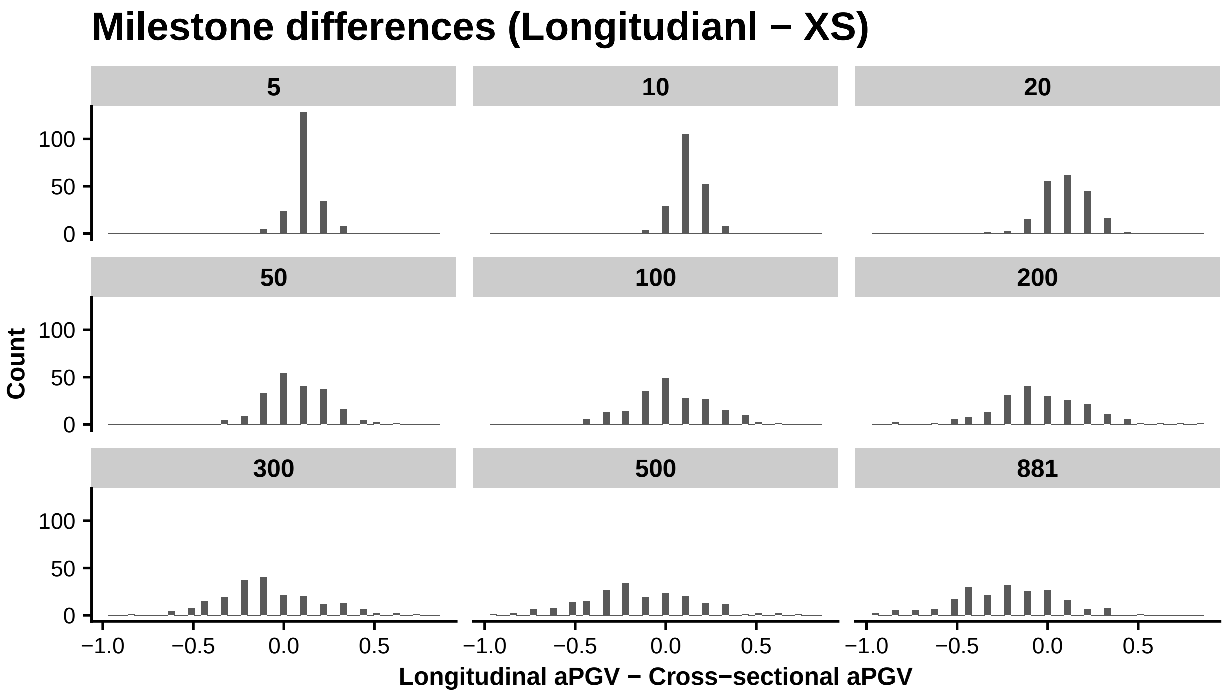
Milestone differences (Longitudianl − XS)
5
10
20
50
100
200
300
500
881
Count
100
50
0
−1.0
−0.5
0.0
0.5
Longitudinal aPGV − Cross−sectional aPGV

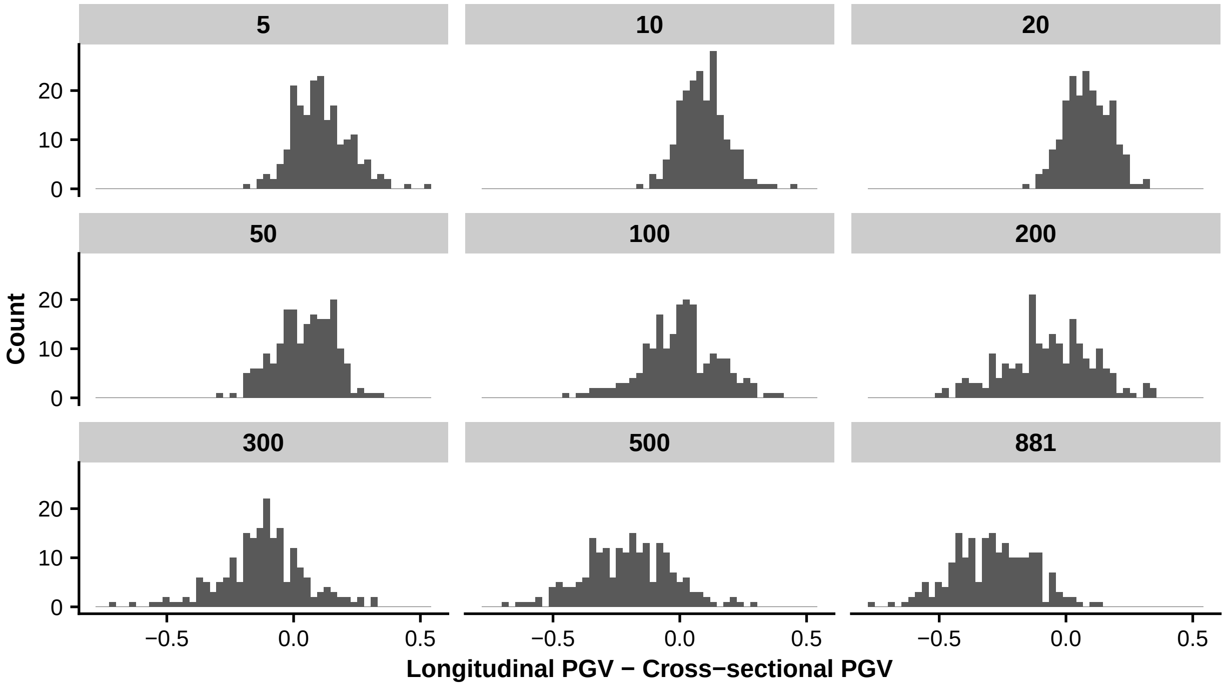
5
10
20
50
100
200
300
500
881
Count
20
10
0
−0.5
0.0
0.5
Longitudinal PGV − Cross−sectional PGV

19 Male, Gonion-Pogonion

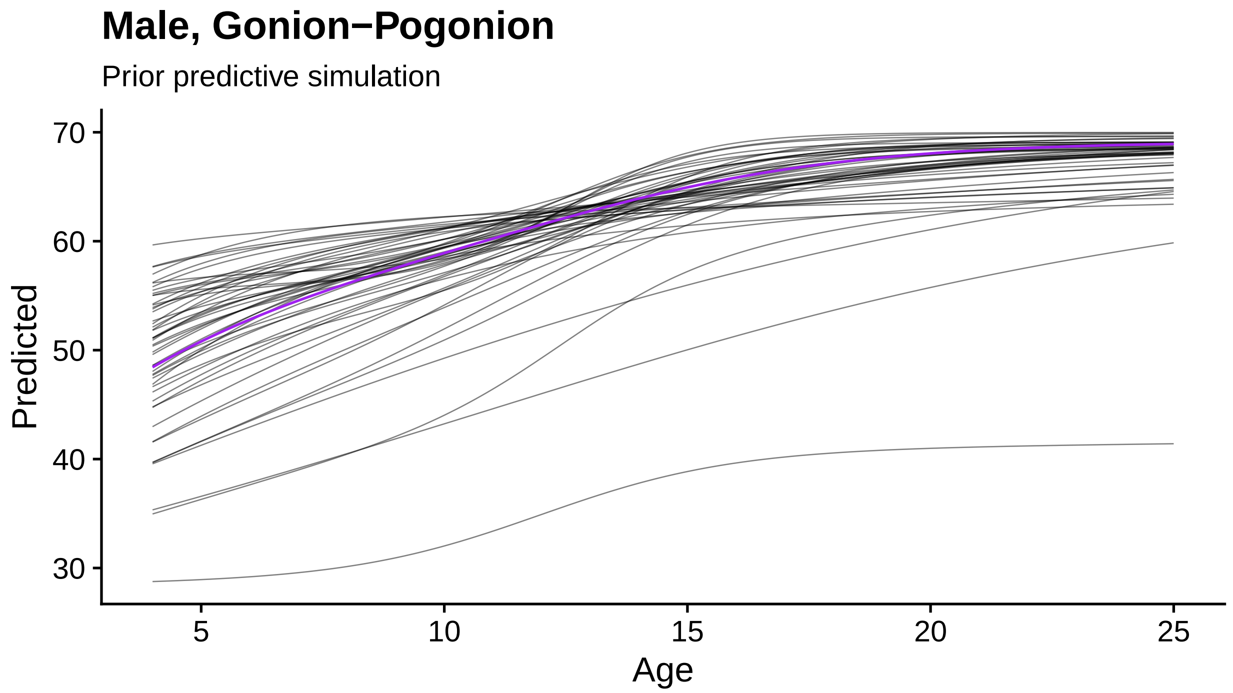

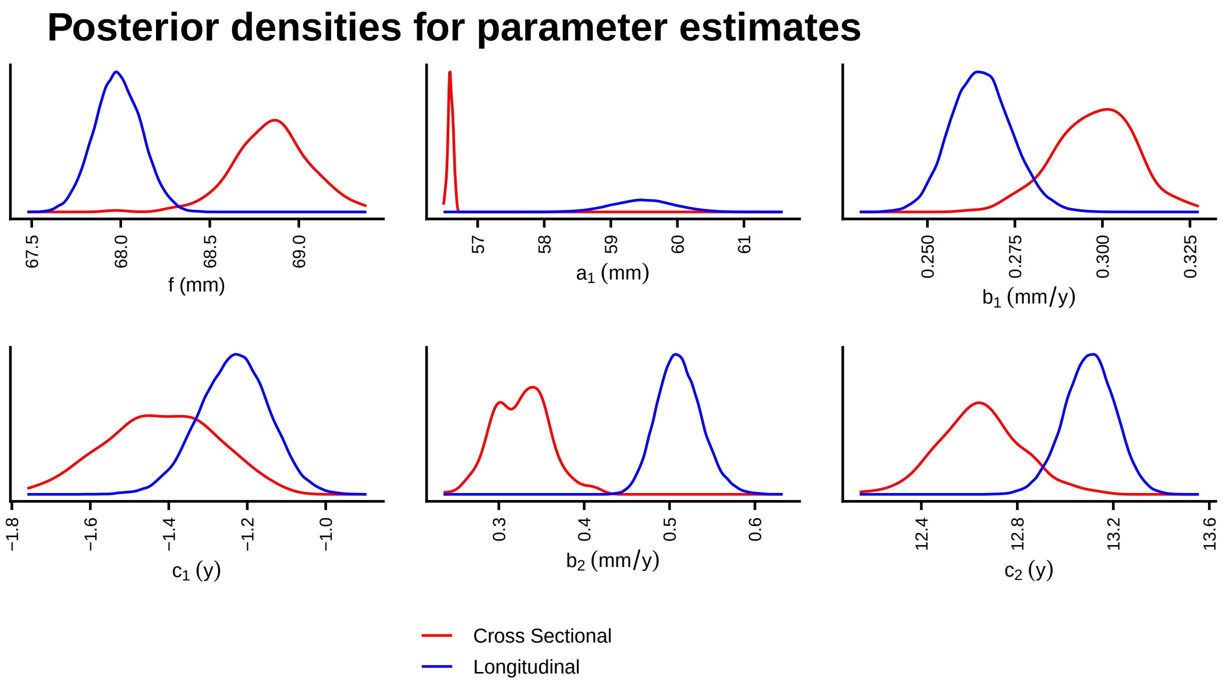

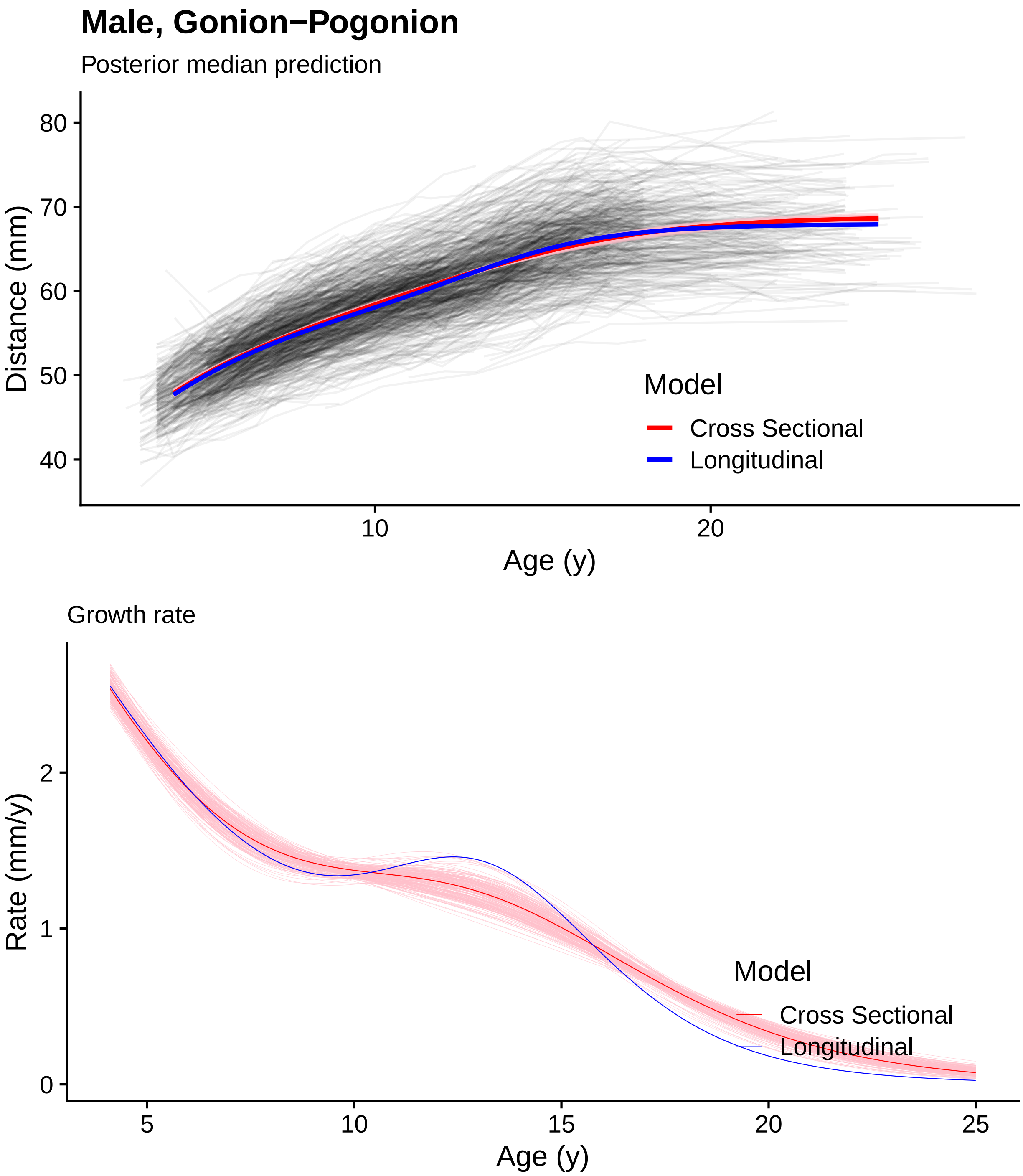
Male, Gonion−Pogonion
Posterior median prediction
Distance (mm)
80
70
60
50
40
10
20
Age (y)
Model
Cross Sectional
Longitudinal
Growth rate
Rate (mm/y)
2
1
0
5
10
15
20
25
Age (y)
Model
Cross Sectional
Longitudinal

Table 35: Longitudinal Model Summary

Parameter	Mean	Median	Std. Dev.	MAD	5%	95%	$\hat{r}$	Bulk ESS	Tail ESS
f	67.99	67.98	0.133	0.134	67.77	68.21	1	1529	4353
a1	59.49	59.49	0.466	0.462	58.72	60.26	1	18603	23870
b1	0.27	0.27	0.009	0.009	0.25	0.28	1	15633	24120
c1	-1.23	-1.23	0.089	0.089	-1.38	-1.09	1	36948	30355
b2	0.51	0.51	0.026	0.026	0.47	0.56	1	14830	24444
c2	13.10	13.10	0.106	0.106	12.92	13.27	1	14129	24437
sigma	1.48	1.48	0.012	0.012	1.46	1.50	1	55592	29329
sigma_ID	3.38	3.38	0.078	0.078	3.26	3.52	1	66470	31122

Table 36: Median Coefficients

Model	f	a_1	b_1	c_1	b_2	c_2	σ	σ_{ID}
Longitudinal	67.98	59.49	0.27	-1.23	0.51	13.10	1.48	3.38
Cross Sectional	68.85	56.59	0.30	-1.41	0.33	12.64	3.67	NA

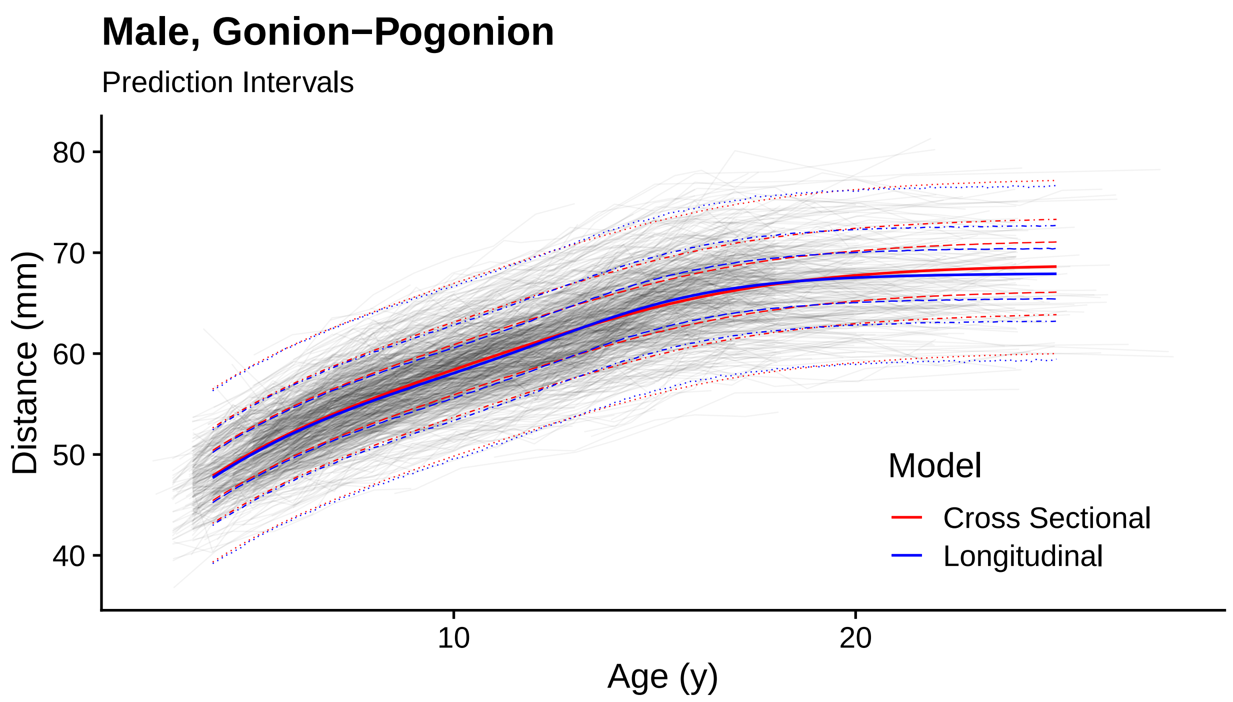

Male, Gonion−Pogonion
Prediction Intervals
80
70
60
50
40
Distance (mm)
10
20
Age (y)
Model
Cross Sectional
Longitudinal

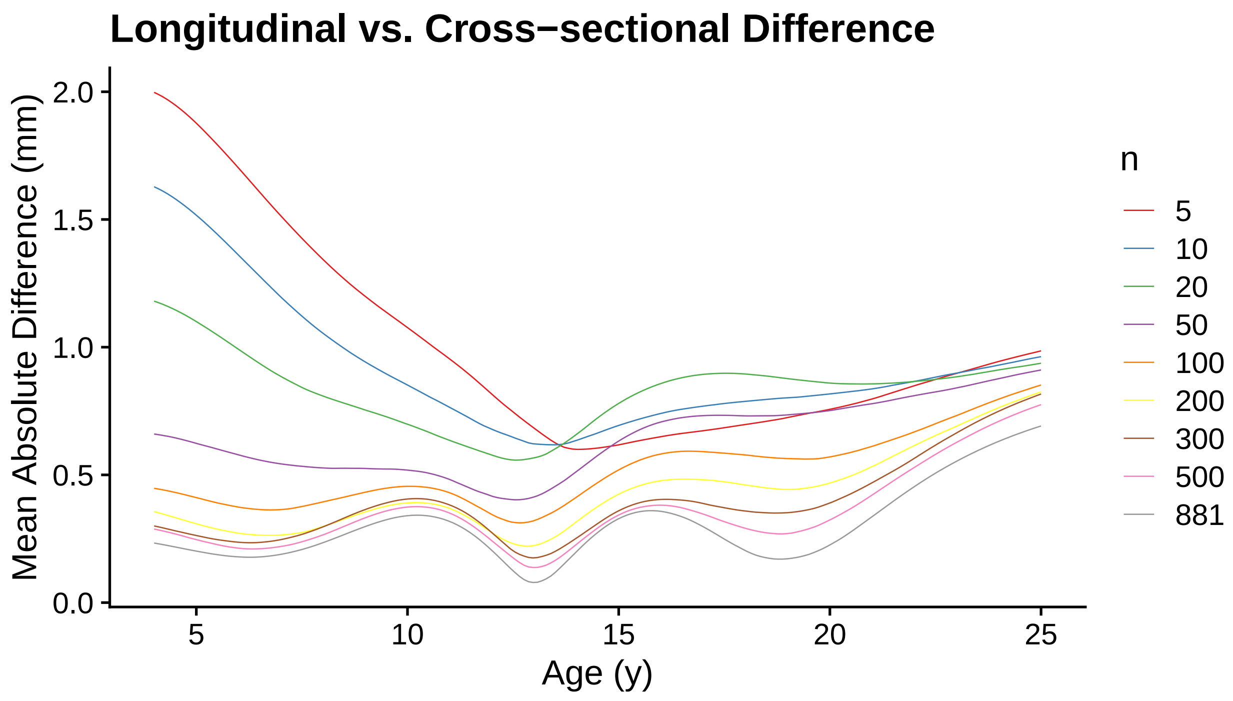

Longitudinal vs. Cross−sectional Difference
2.0
1.5
1.0
0.5
0.0
Mean Absolute Difference (mm)
5
10
15
20
25
Age (y)
n
5
10
20
50
100
200
300
500
881

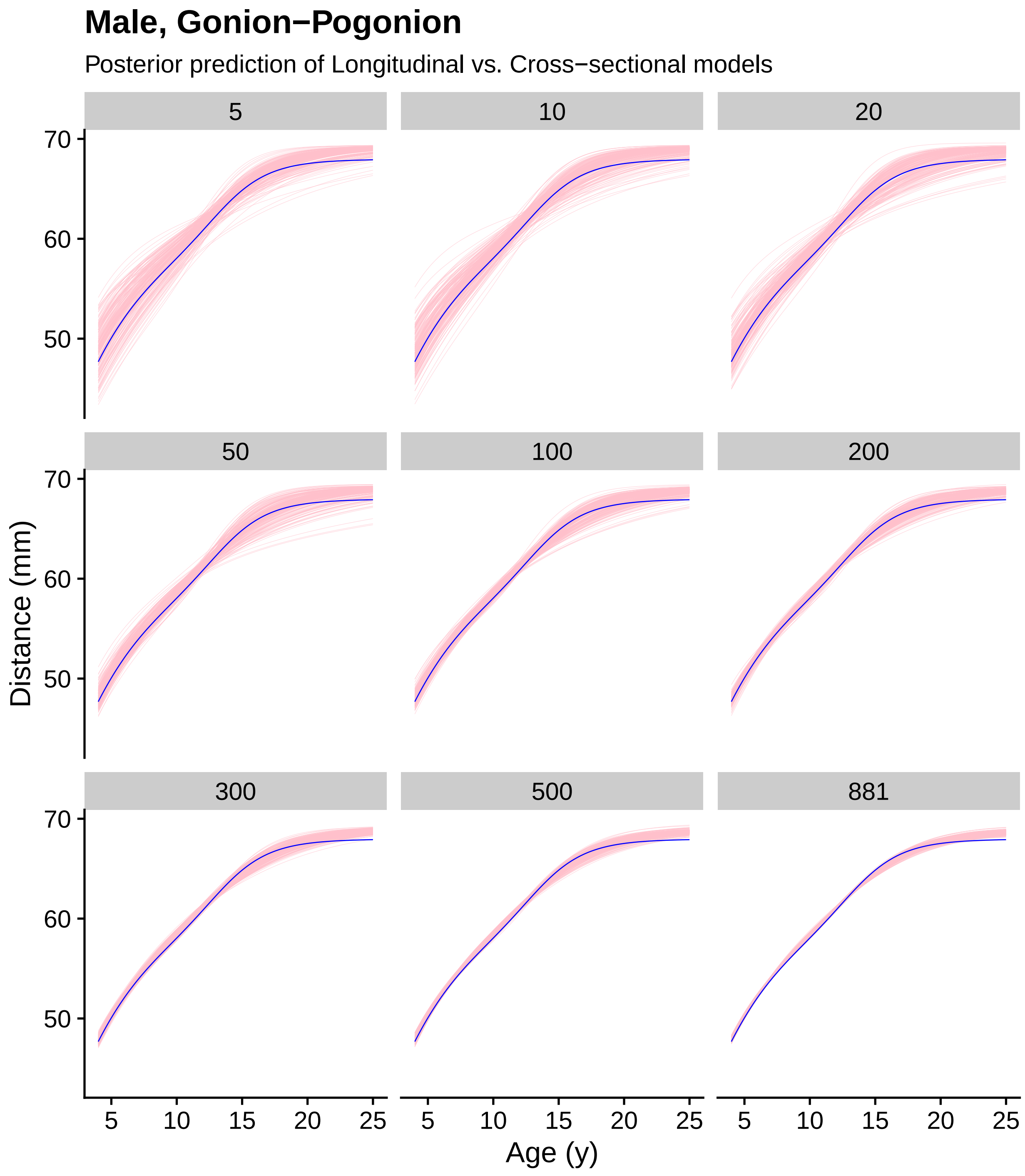

Male, Gonion−Pogonion
Posterior prediction of Longitudinal vs. Cross−sectional models
5
10
20
50
100
200
300
500
881
70
60
50
Distance (mm)
5
10
15
20
25
Age (y)

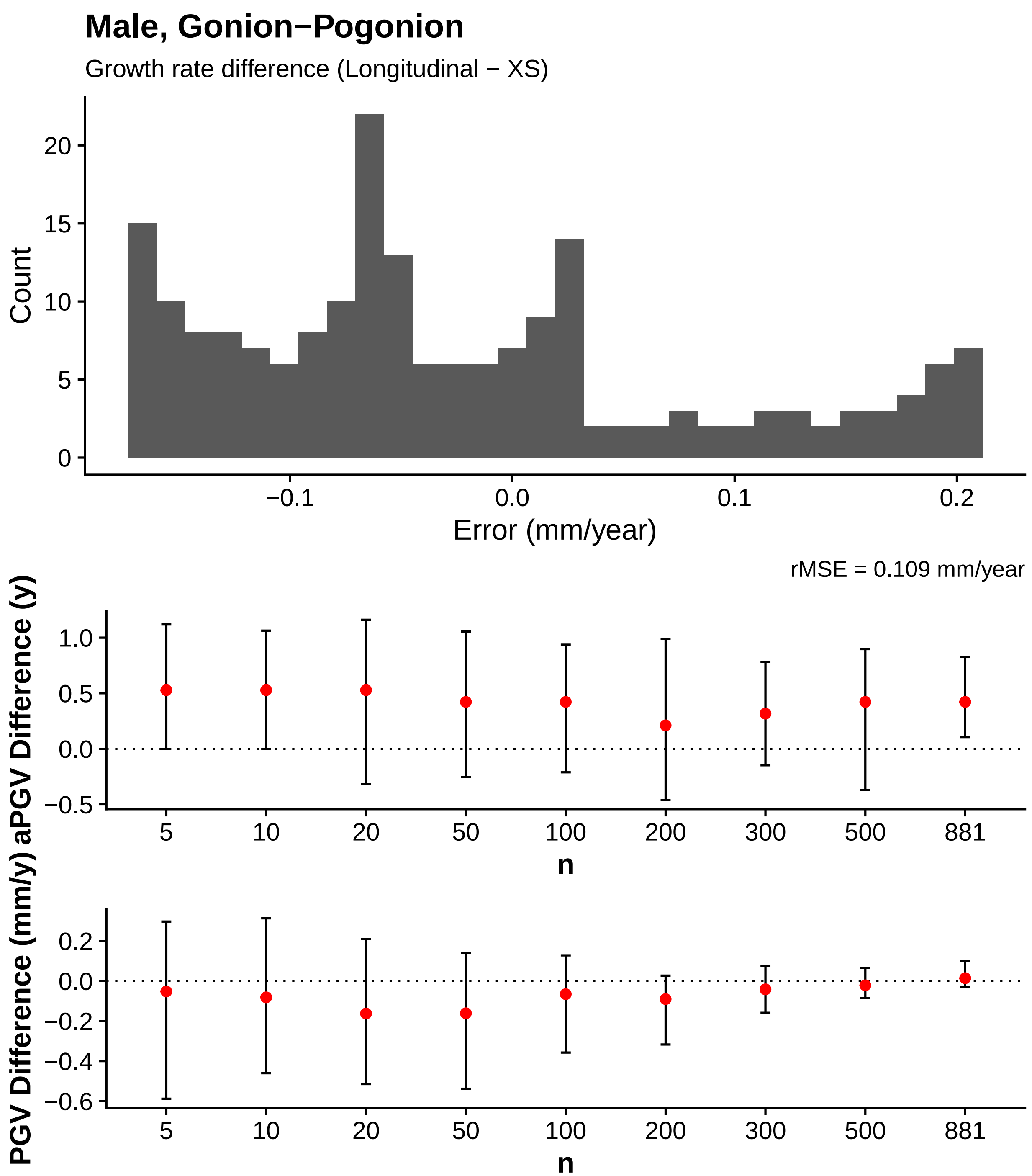

Male, Gonion−Pogonion
Growth rate difference (Longitudinal − XS)
Count
Error (mm/year)
rMSE = 0.109 mm/year
PGV Difference (mm/y) aPGV Difference (y)
n
n

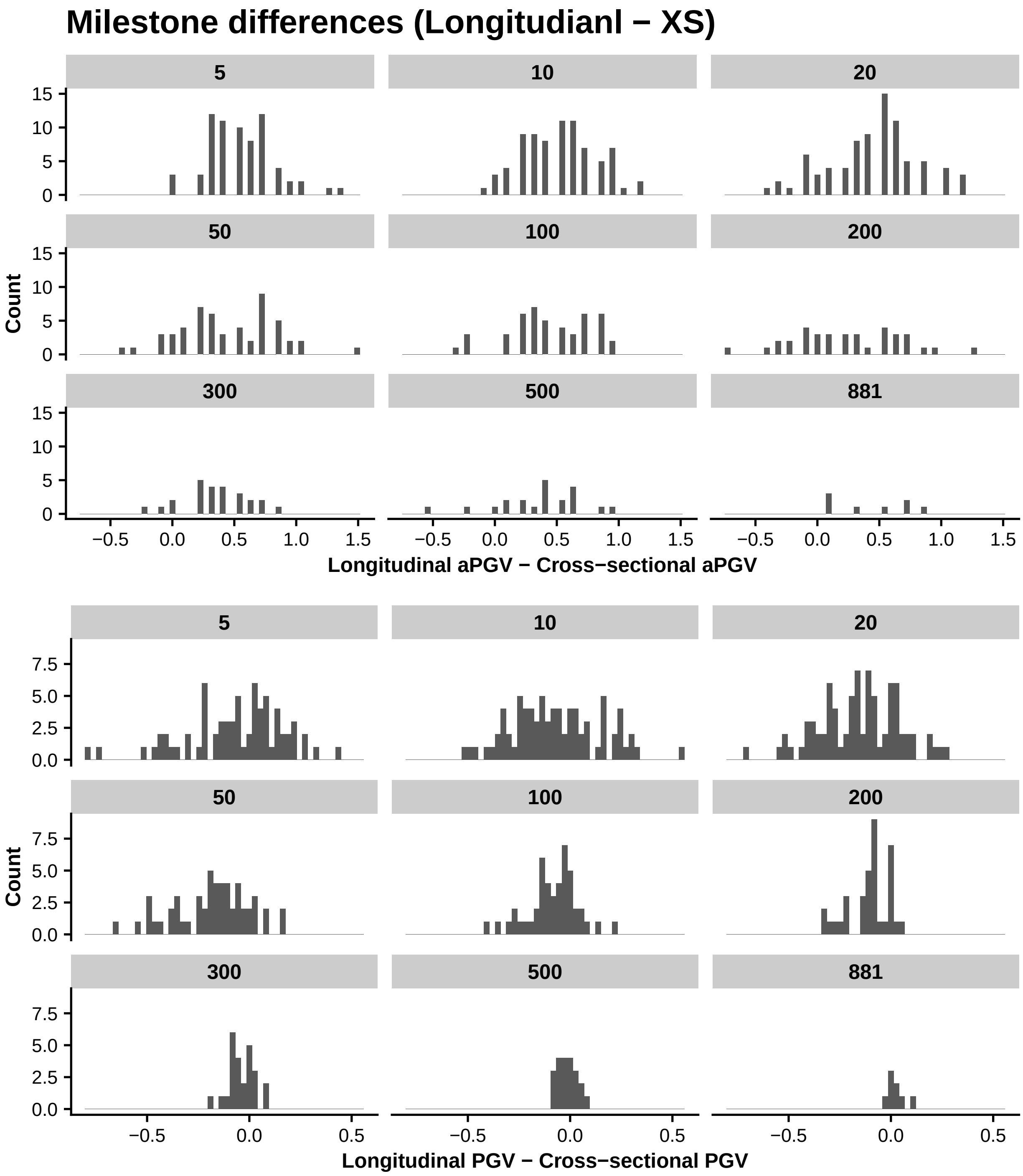
Milestone differences (Longitudianl − XS)
5
10
20
50
100
200
300
500
881
Count
15
10
5
0
−0.5
0.0
0.5
1.0
1.5
Longitudinal aPGV − Cross−sectional aPGV
7.5
5.0
2.5
0.0
Longitudinal PGV − Cross−sectional PGV

20 Male, Menton-ANS

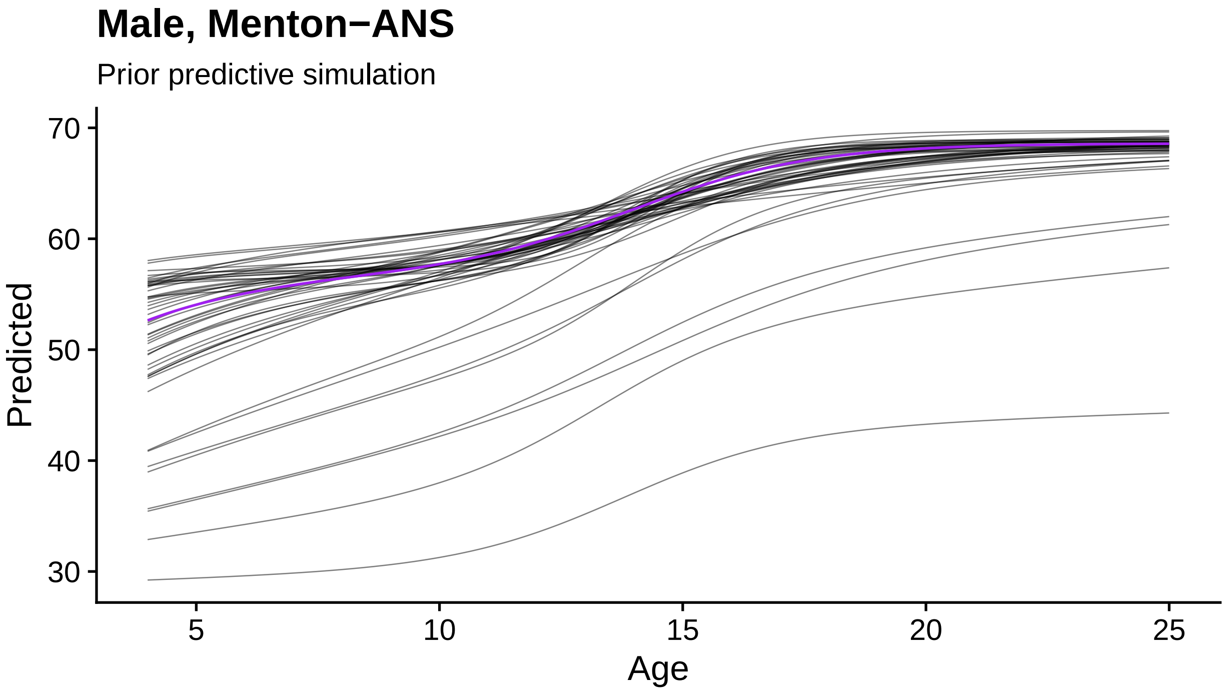

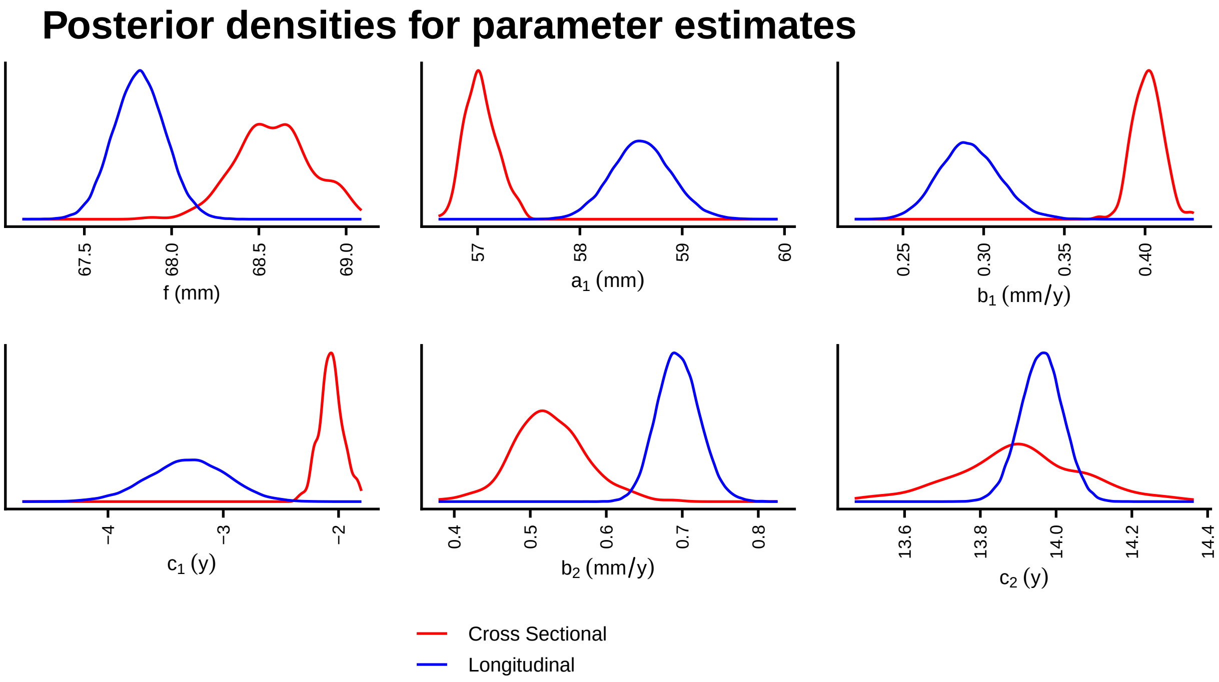

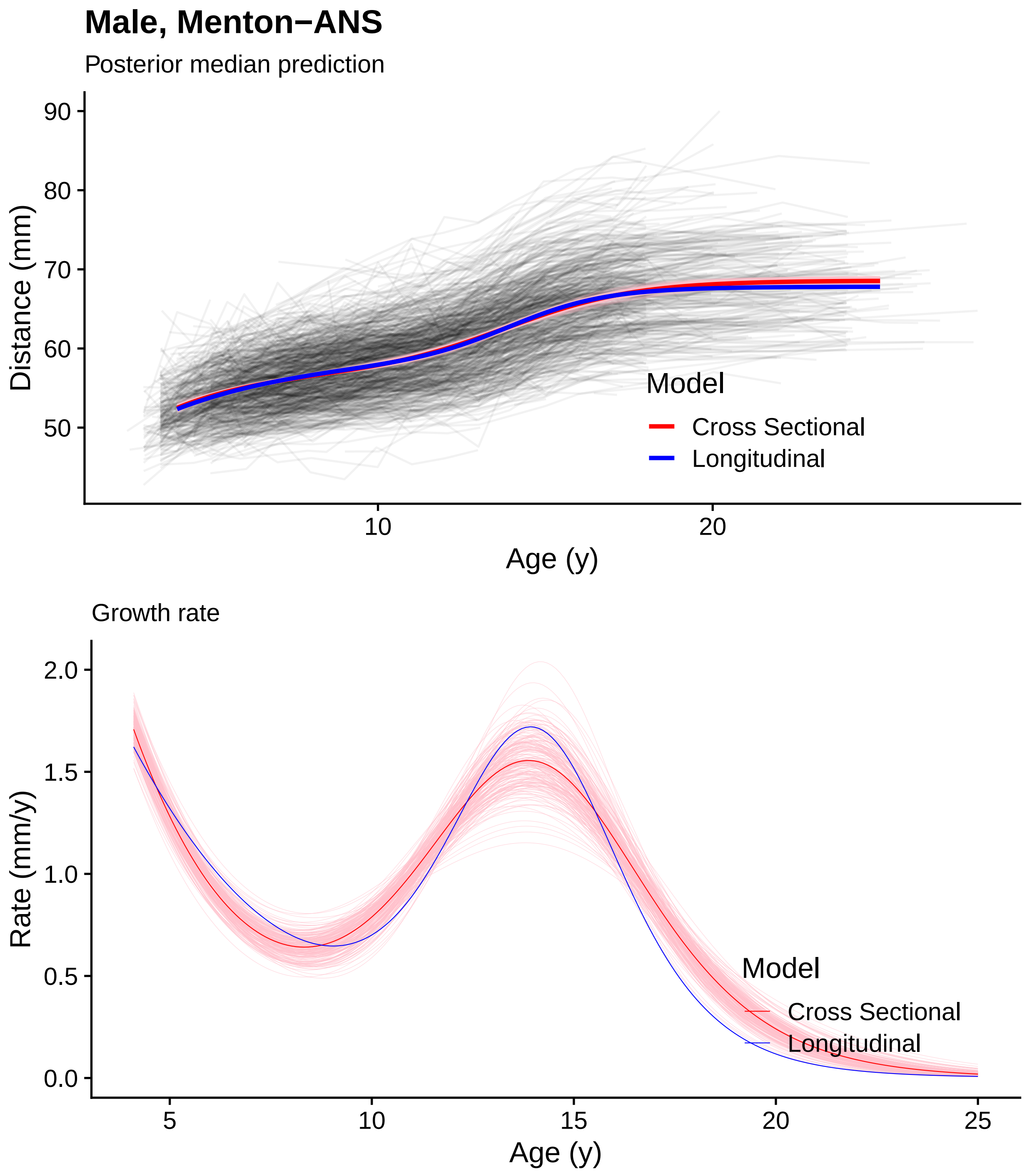
Male, Menton−ANS
Posterior median prediction
Distance (mm)
90
80
70
60
50
10
20
Age (y)
Model
Cross Sectional
Longitudinal
Growth rate
Rate (mm/y)
2.0
1.5
1.0
0.5
0.0
5
10
15
20
25
Age (y)
Model
Cross Sectional
Longitudinal

Table 37: Longitudinal Model Summary

Parameter	Mean	Median	Std. Dev.	MAD	5%	95%	$\hat{r}$	Bulk ESS	Tail ESS
f	67.82	67.82	0.148	0.148	67.57	68.06	1	1655	3633
a1	58.60	58.60	0.295	0.291	58.12	59.10	1	7759	16018
b1	0.29	0.29	0.019	0.019	0.26	0.32	1	13835	20711
c1	-3.31	-3.30	0.342	0.342	-3.88	-2.76	1	16128	23124
b2	0.70	0.69	0.028	0.028	0.65	0.74	1	18685	26727
c2	13.96	13.96	0.056	0.056	13.87	14.05	1	23273	29044
sigma	1.75	1.75	0.014	0.014	1.73	1.78	1	51121	31215
sigma_ID	4.01	4.01	0.093	0.093	3.86	4.16	1	59572	30264

Table 38: Median Coefficients

Model	f	a_1	b_1	c_1	b_2	c_2	σ	σ_{ID}
Longitudinal	67.82	58.60	0.29	-3.30	0.69	13.96	1.75	4.01
Cross Sectional	68.58	57.02	0.40	-2.07	0.52	13.90	4.33	NA

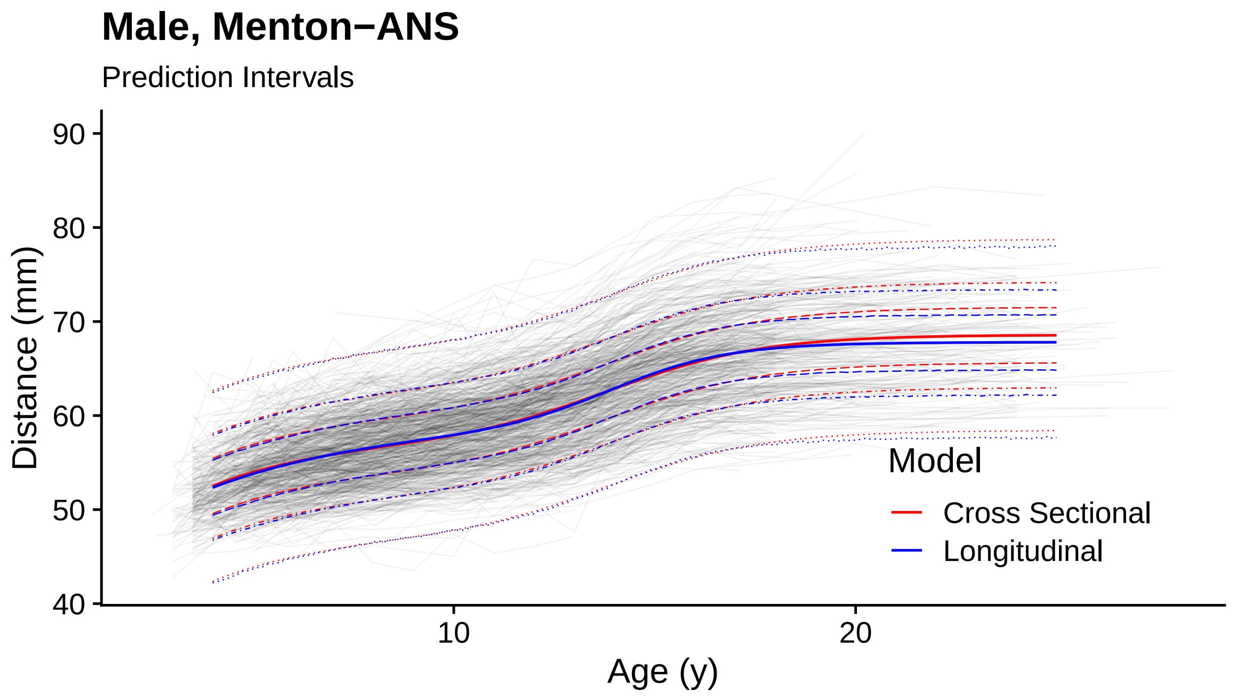
Male, Menton−ANS
Prediction Intervals
Distance (mm)
90
80
70
60
50
40
10
20
Age (y)
Model
Cross Sectional
Longitudinal

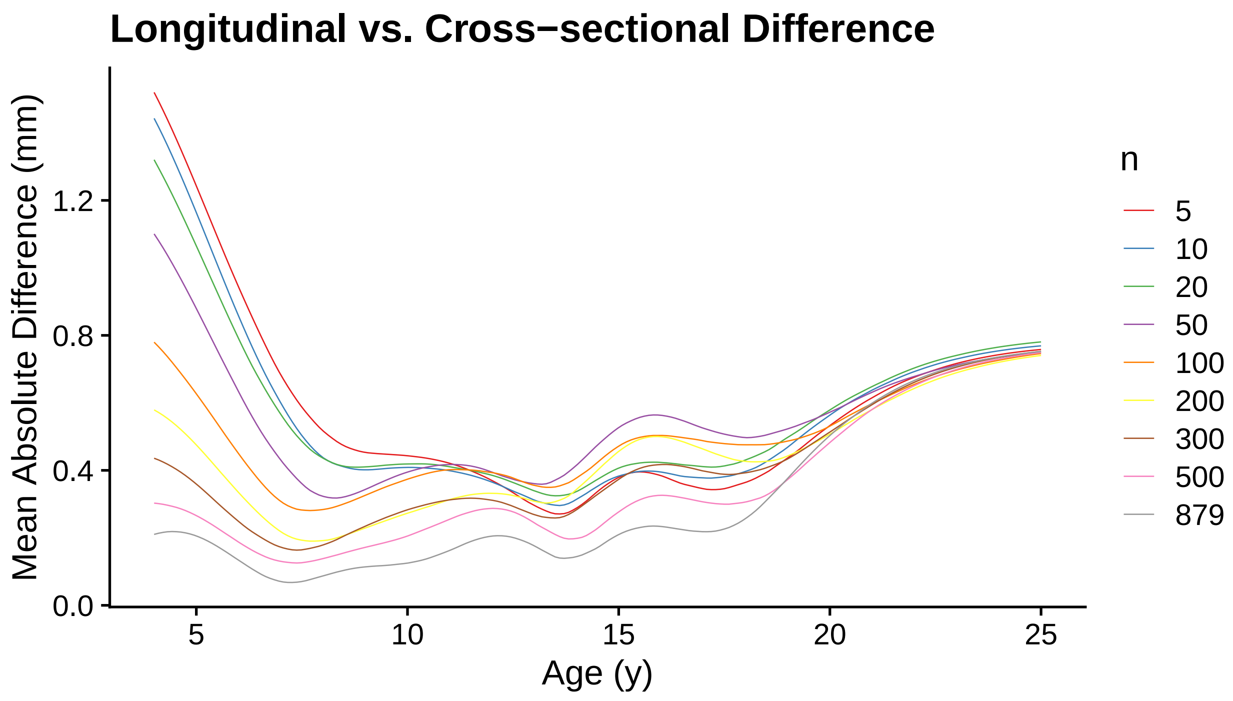
Longitudinal vs. Cross−sectional Difference
Mean Absolute Difference (mm)
1.2
0.8
0.4
0.0
5
10
15
20
25
Age (y)
n
5
10
20
50
100
200
300
500
879

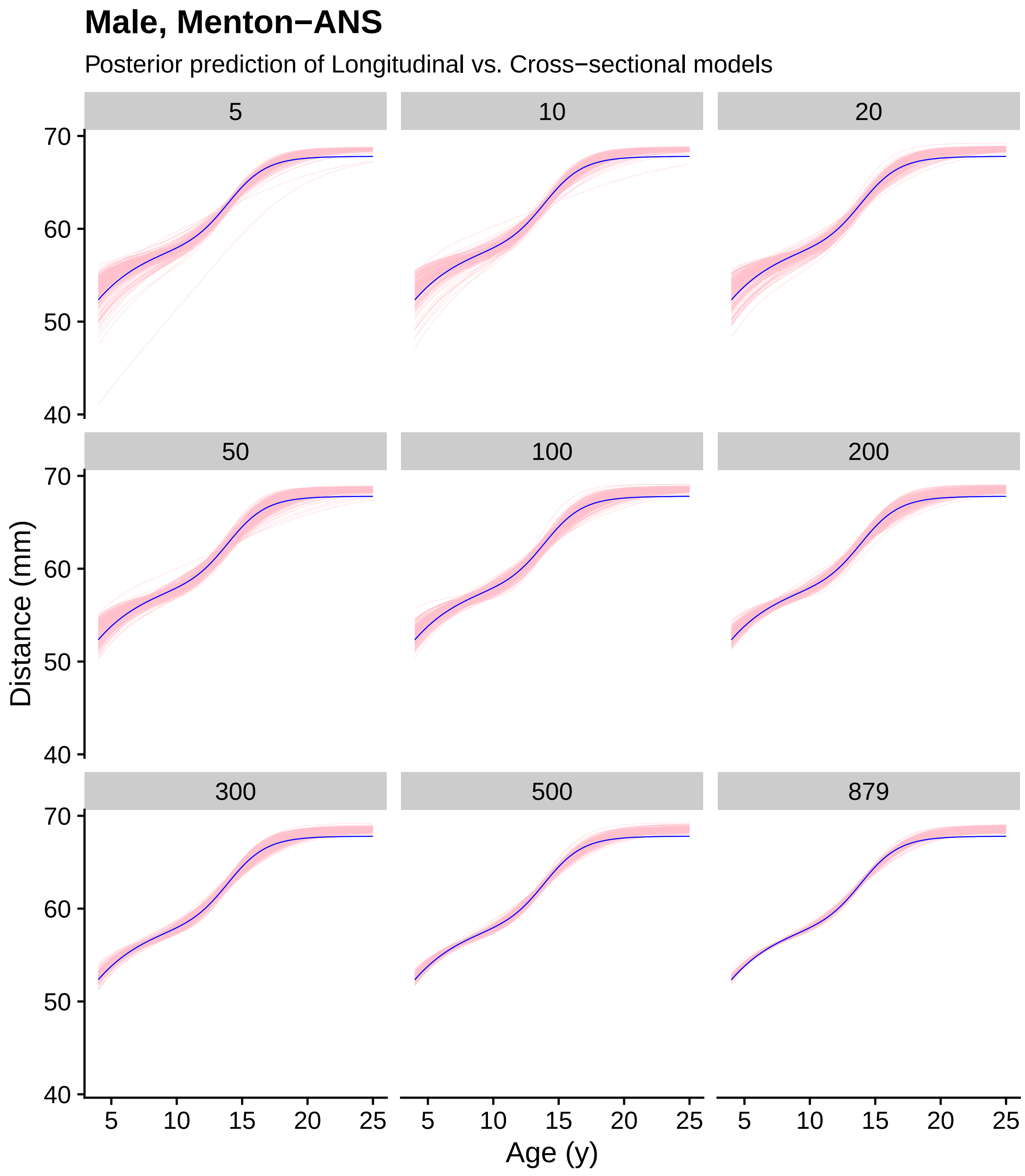
Male, Menton−ANS
Posterior prediction of Longitudinal vs. Cross−sectional models
5
10
20
50
100
200
300
500
879
Distance (mm)
Age (y)
70
60
50
40
5
10
15
20
25

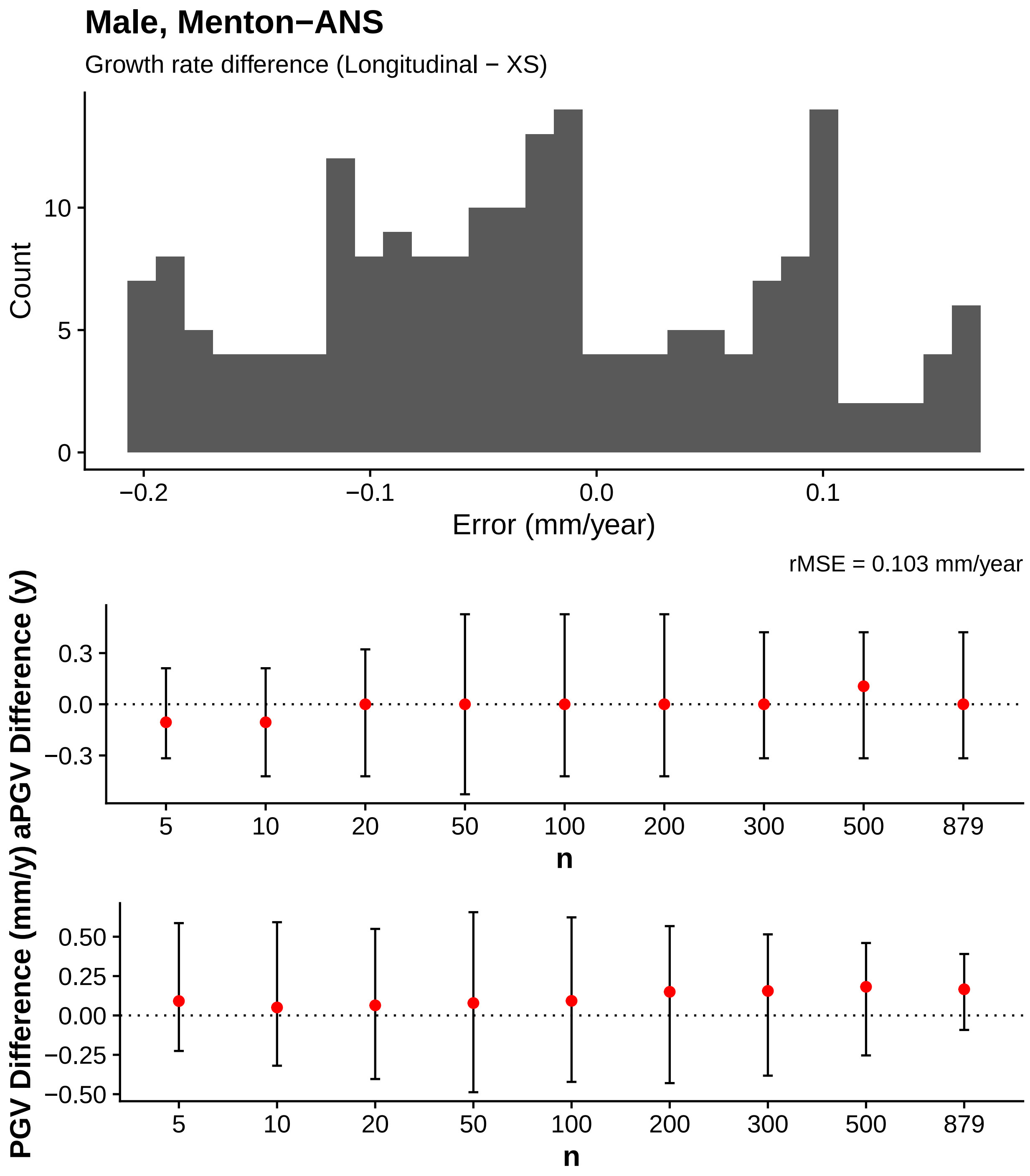
Male, Menton−ANS
Growth rate difference (Longitudinal − XS)
Count
Error (mm/year)
rMSE = 0.103 mm/year
PGV Difference (mm/y) aPGV Difference (y)
n
n

Milestone differences (Longitudianl − XS)

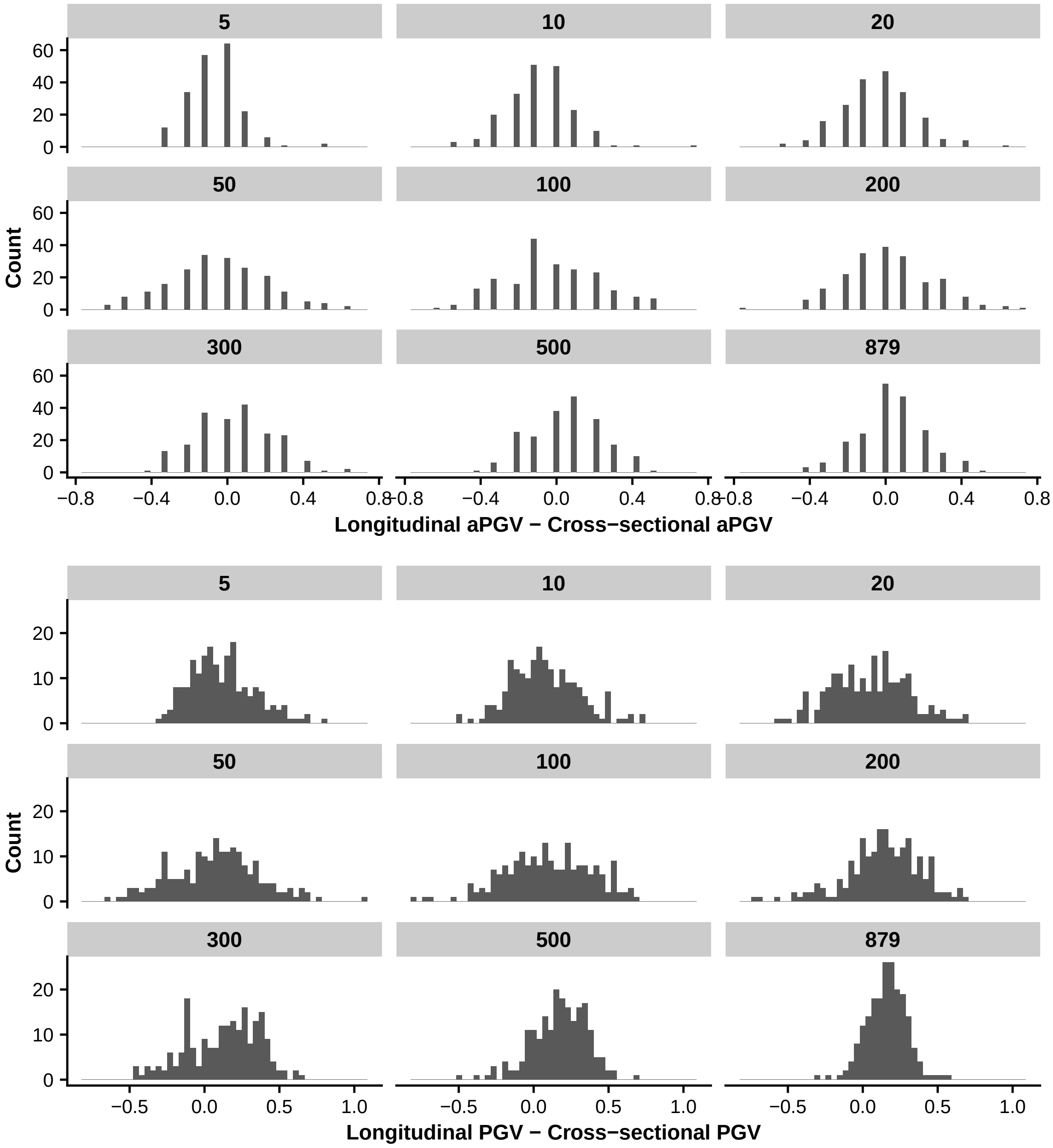

21 Male, Nasion-ANS

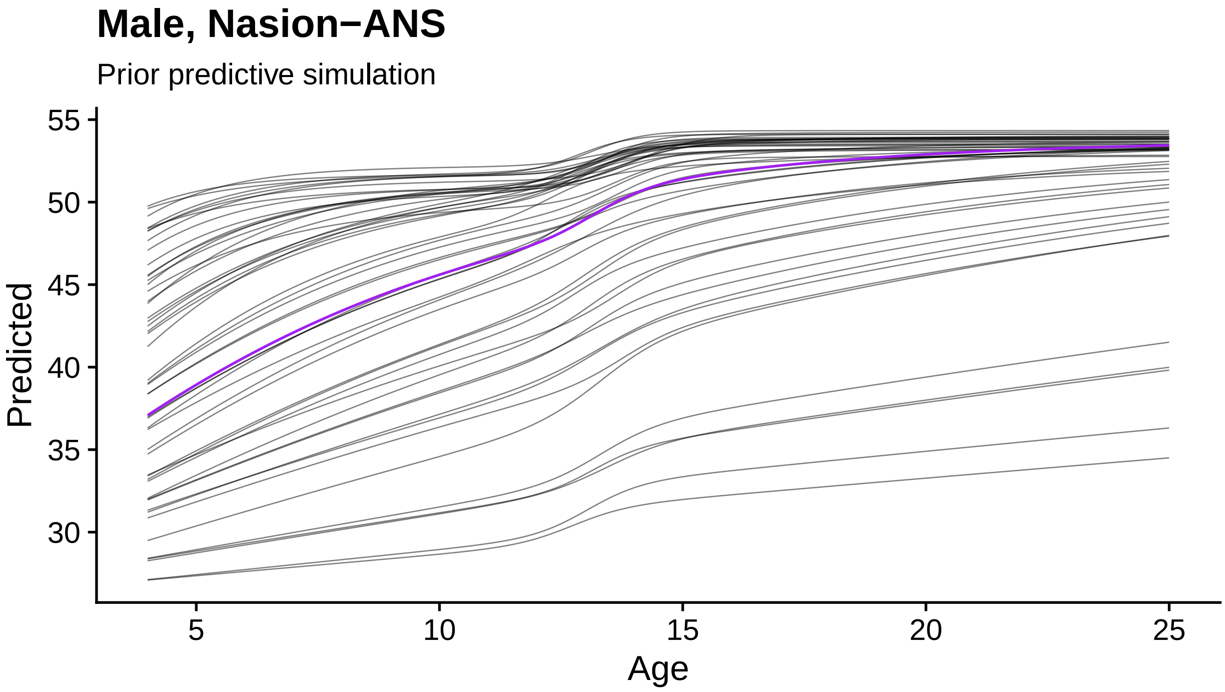

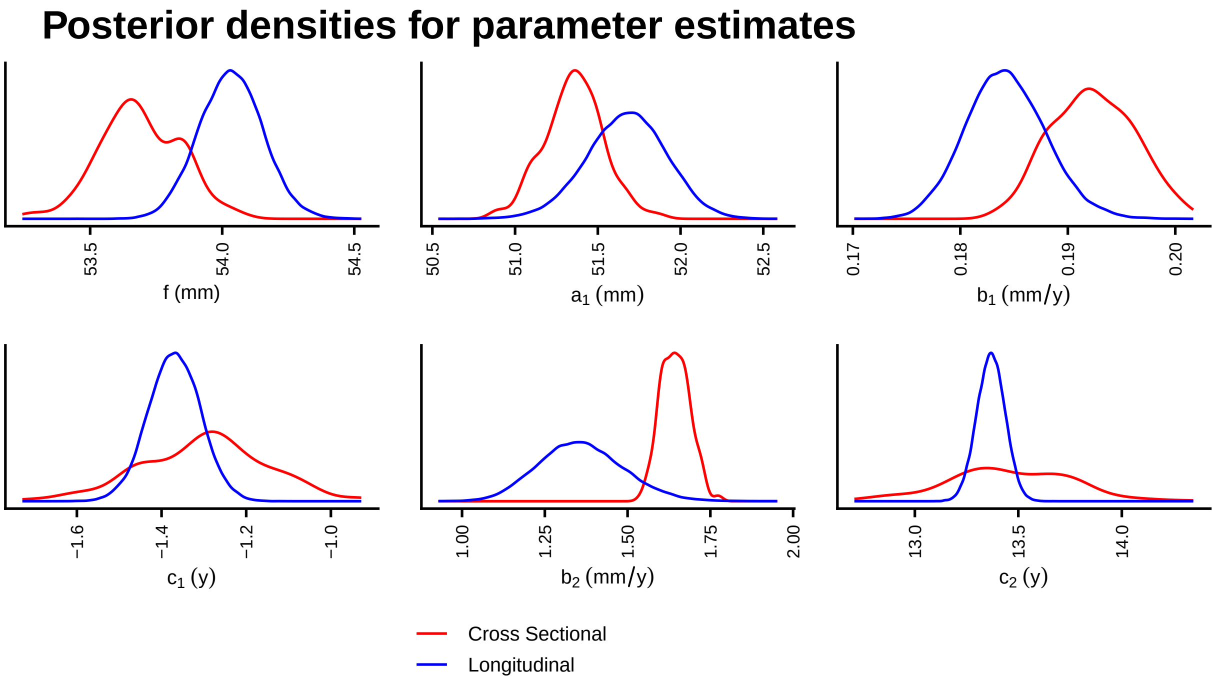

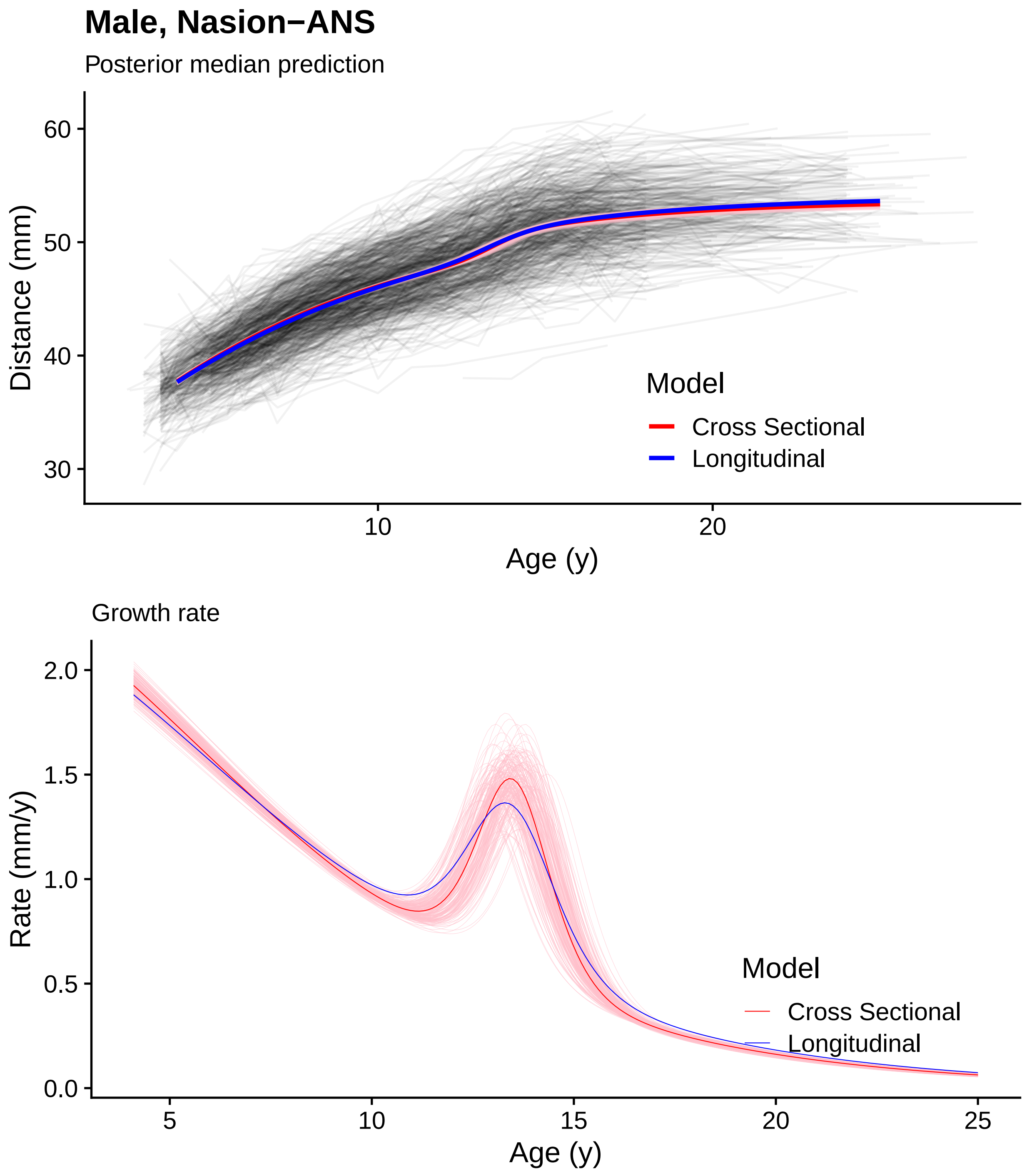
Male, Nasion−ANS
Posterior median prediction
60
50
40
30
Distance (mm)
10
20
Age (y)
Model
Cross Sectional
Longitudinal
Growth rate
2.0
1.5
1.0
0.5
0.0
Rate (mm/y)
5
10
15
20
25
Age (y)
Model
Cross Sectional
Longitudinal

Table 39: Longitudinal Model Summary

Parameter	Mean	Median	Std. Dev.	MAD	5%	95%	$\hat{r}$	Bulk ESS	Tail ESS
f	54.03	54.03	0.121	0.121	53.83	54.23	1	5108	12943
a1	51.67	51.68	0.242	0.240	51.27	52.06	1	15335	23055
b1	0.18	0.18	0.004	0.004	0.18	0.19	1	23689	25989
c1	-1.37	-1.37	0.062	0.062	-1.47	-1.27	1	26700	29268
b2	1.36	1.36	0.127	0.127	1.16	1.58	1	33504	31261
c2	13.37	13.37	0.067	0.068	13.26	13.48	1	59592	32145
sigma	1.28	1.28	0.010	0.010	1.27	1.30	1	54233	32023
sigma_ID	2.37	2.37	0.056	0.056	2.28	2.47	1	63527	30413

Table 40: Median Coefficients

Model	f	a_1	b_1	c_1	b_2	c_2	σ	σ_{ID}
Longitudinal	54.03	51.68	0.18	-1.37	1.36	13.37	1.28	2.37
Cross Sectional	53.68	51.36	0.19	-1.29	1.64	13.44	2.68	NA

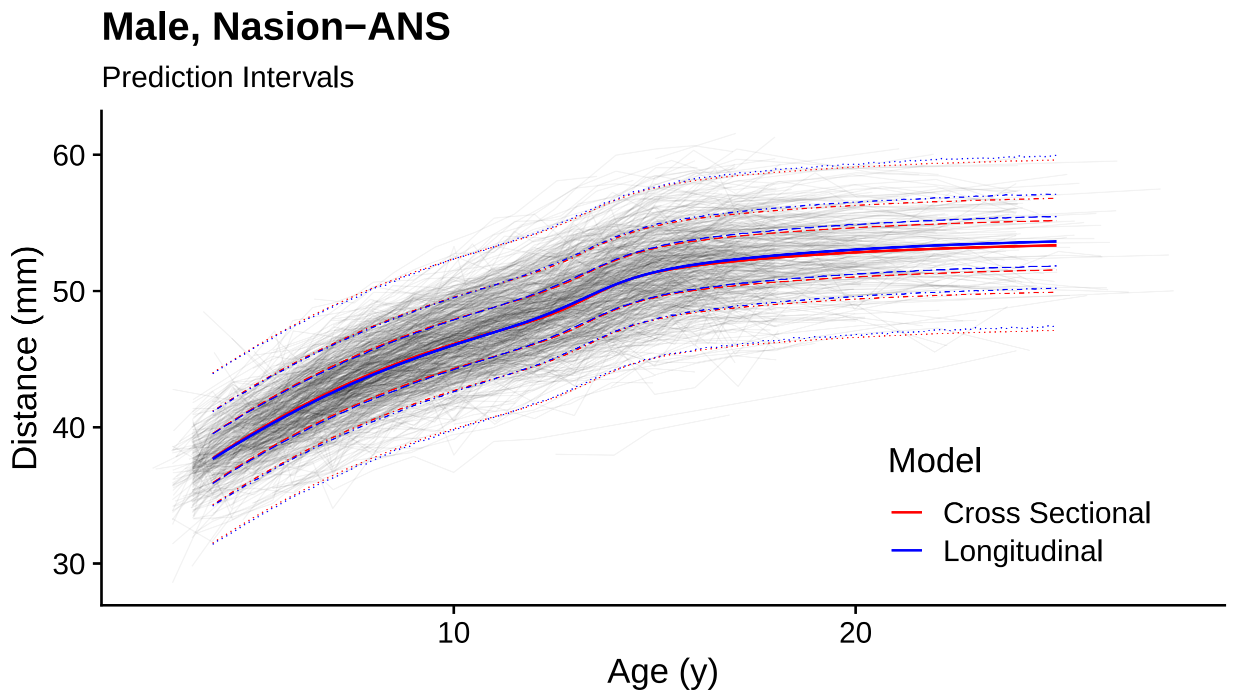

Male, Nasion−ANS
Prediction Intervals
Distance (mm)
60
50
40
30
10
20
Age (y)
Model
Cross Sectional
Longitudinal

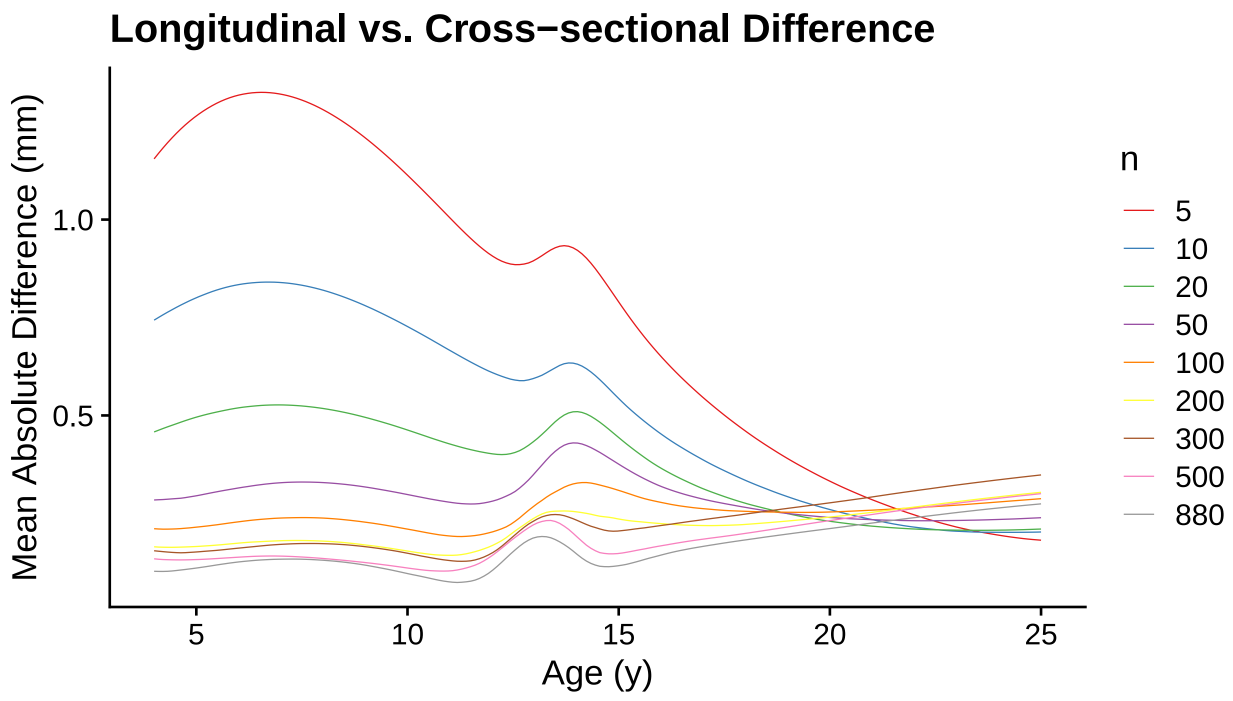

Longitudinal vs. Cross−sectional Difference
Mean Absolute Difference (mm)
1.0
0.5
5
10
15
20
25
Age (y)
n
5
10
20
50
100
200
300
500
880

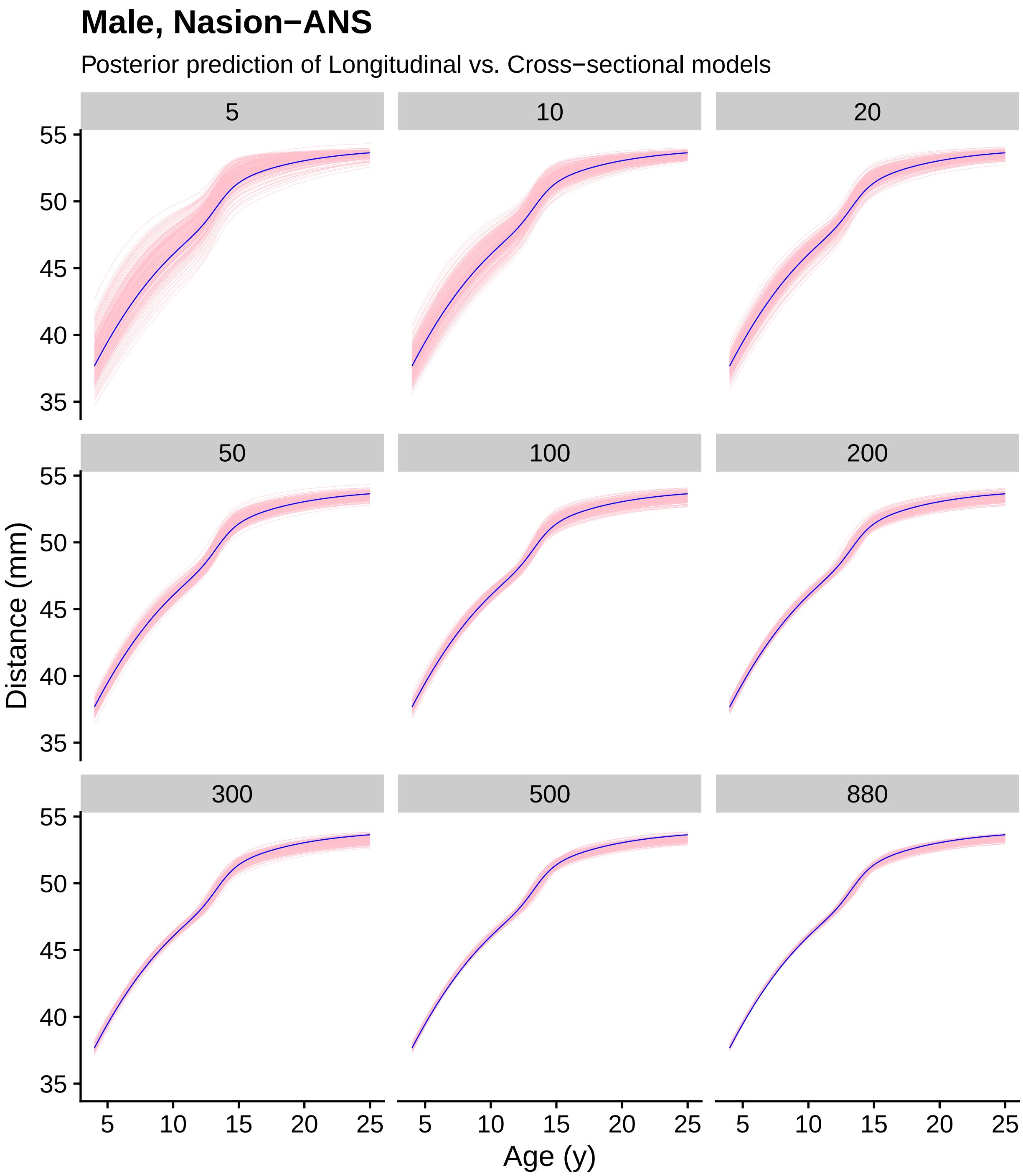
Male, Nasion−ANS
Posterior prediction of Longitudinal vs. Cross−sectional models
5
10
20
50
100
200
300
500
880
Distance (mm)
Age (y)
35
40
45
50
55
5
10
15
20
25

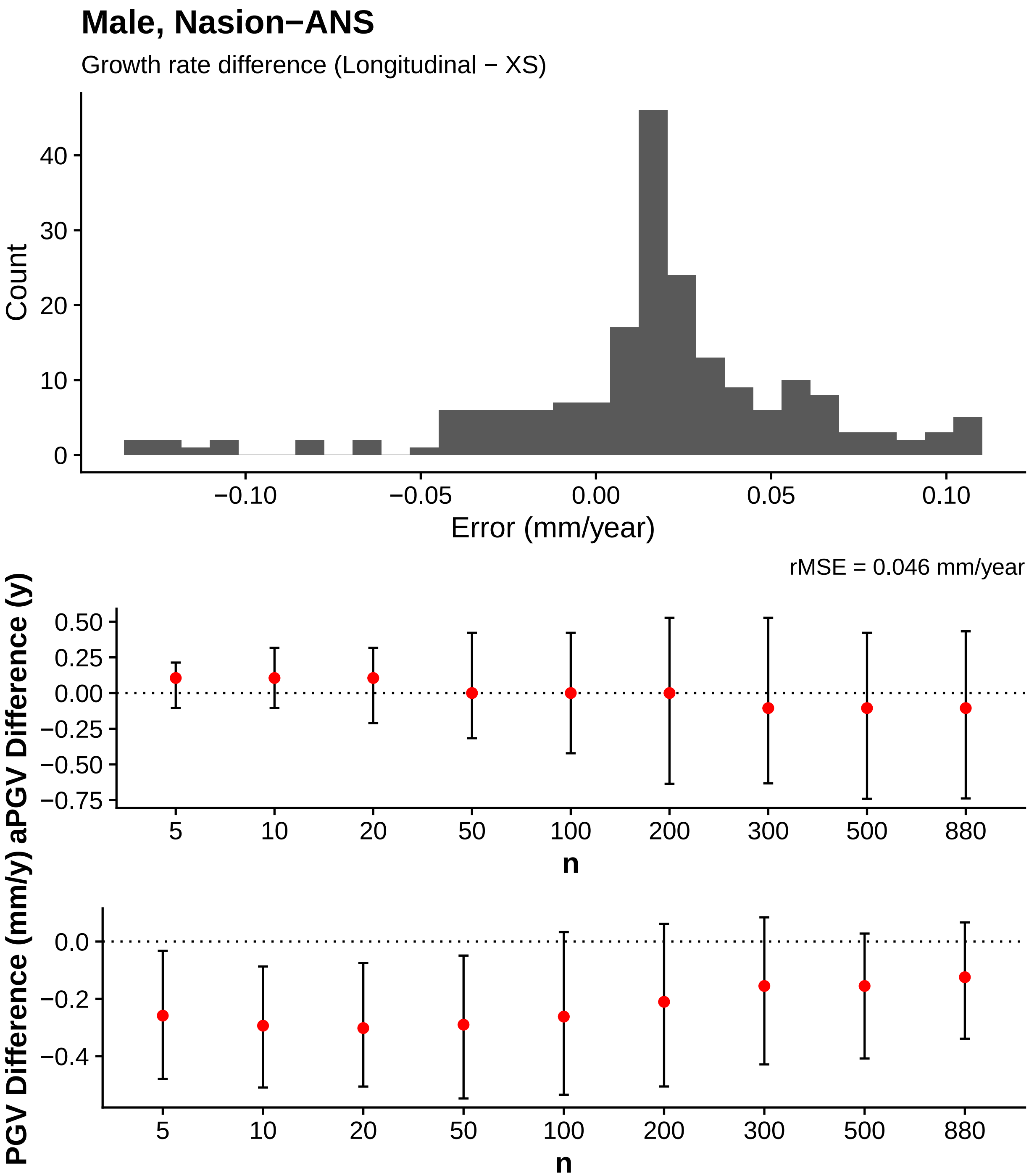
Male, Nasion−ANS
Growth rate difference (Longitudinal − XS)
Count
40
30
20
10
0
−0.10
−0.05
0.00
0.05
0.10
Error (mm/year)
rMSE = 0.046 mm/year
PGV Difference (mm/y) aPGV Difference (y)
0.50
0.25
0.00
−0.25
−0.50
−0.75
5
10
20
50
100
200
300
500
880
n
0.0
−0.2
−0.4
5
10
20
50
100
200
300
500
880
n

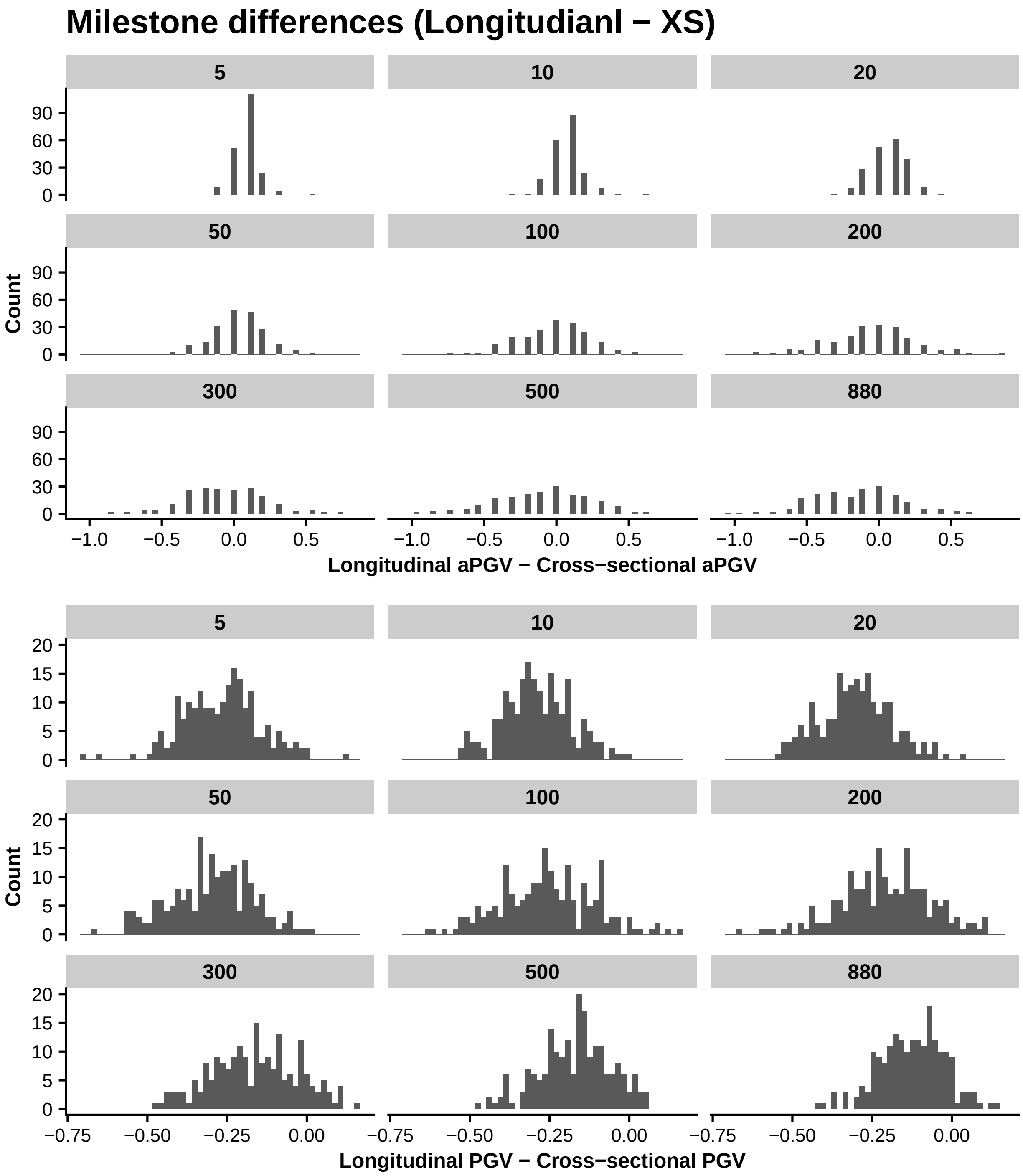
Milestone differences (Longitudianl − XS)
5
10
20
50
100
200
300
500
880
Count
Longitudinal aPGV − Cross−sectional aPGV
Longitudinal PGV − Cross−sectional PGV

22 Male, Nasion-Basion

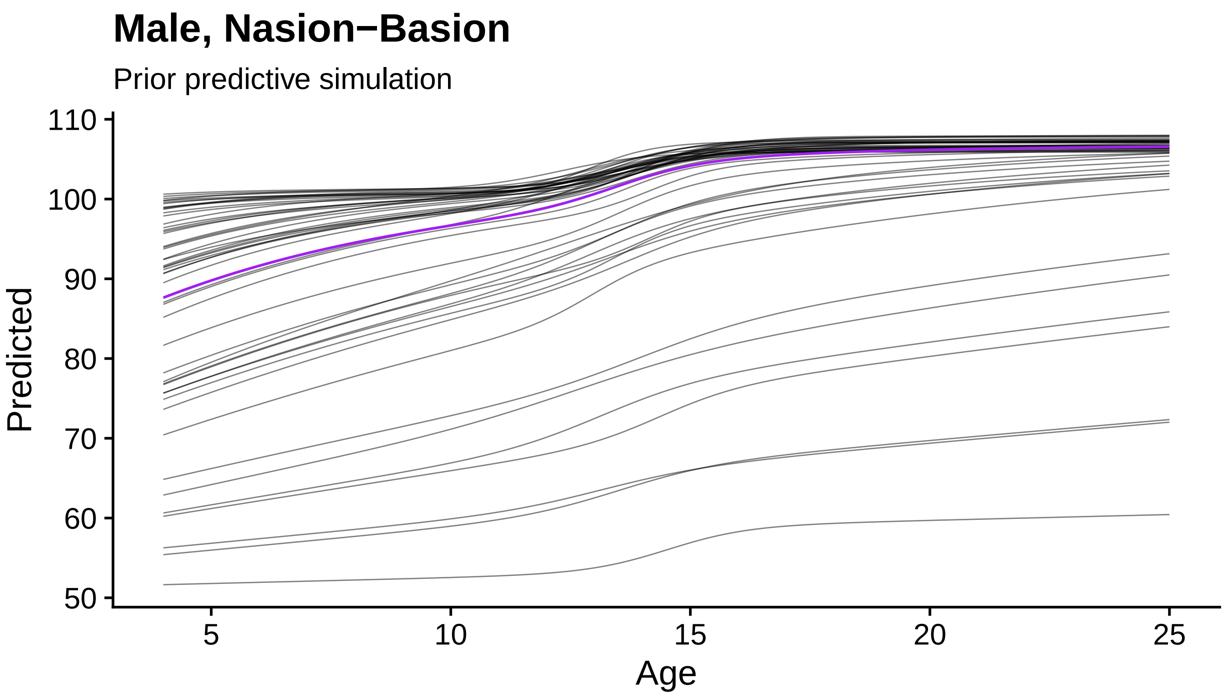

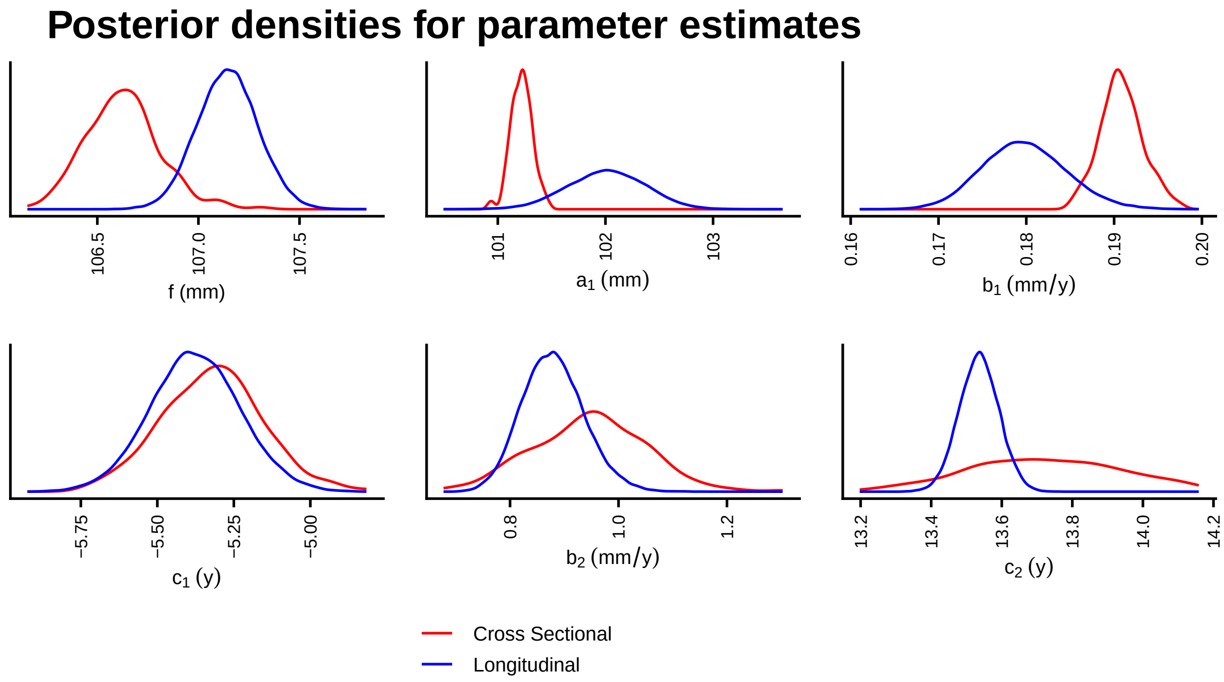

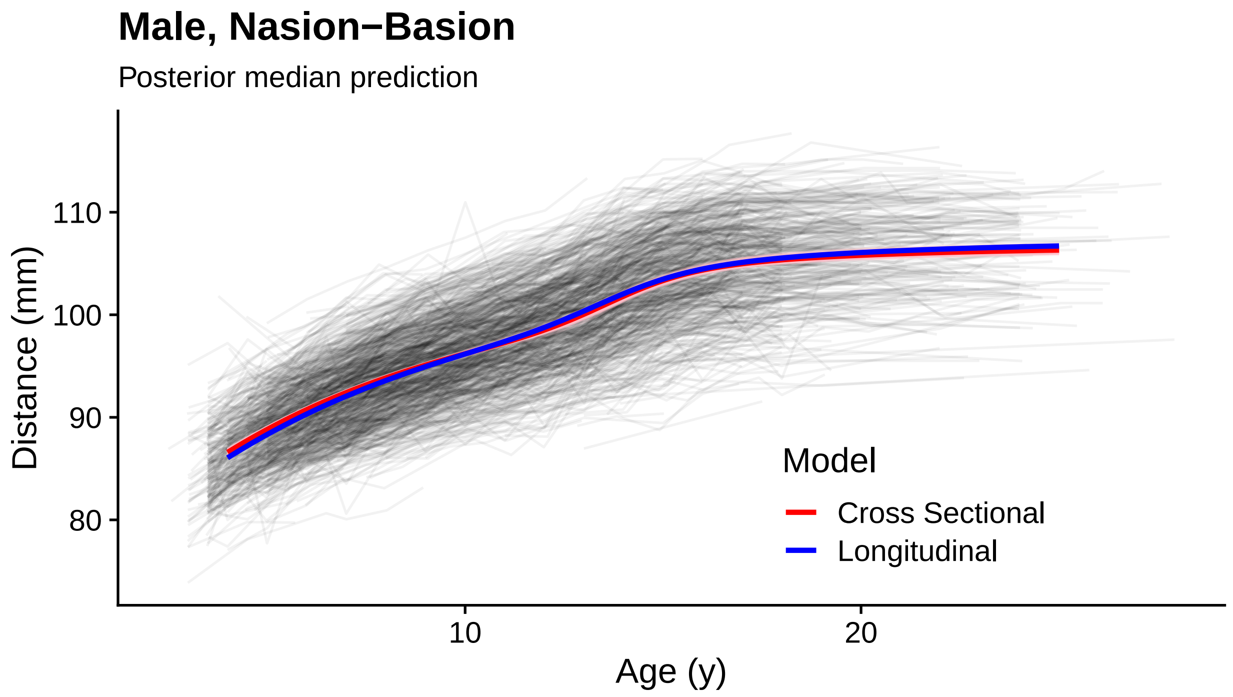
Male, Nasion−Basion
Posterior median prediction
Distance (mm)
110
100
90
80
10
20
Age (y)
Model
Cross Sectional
Longitudinal

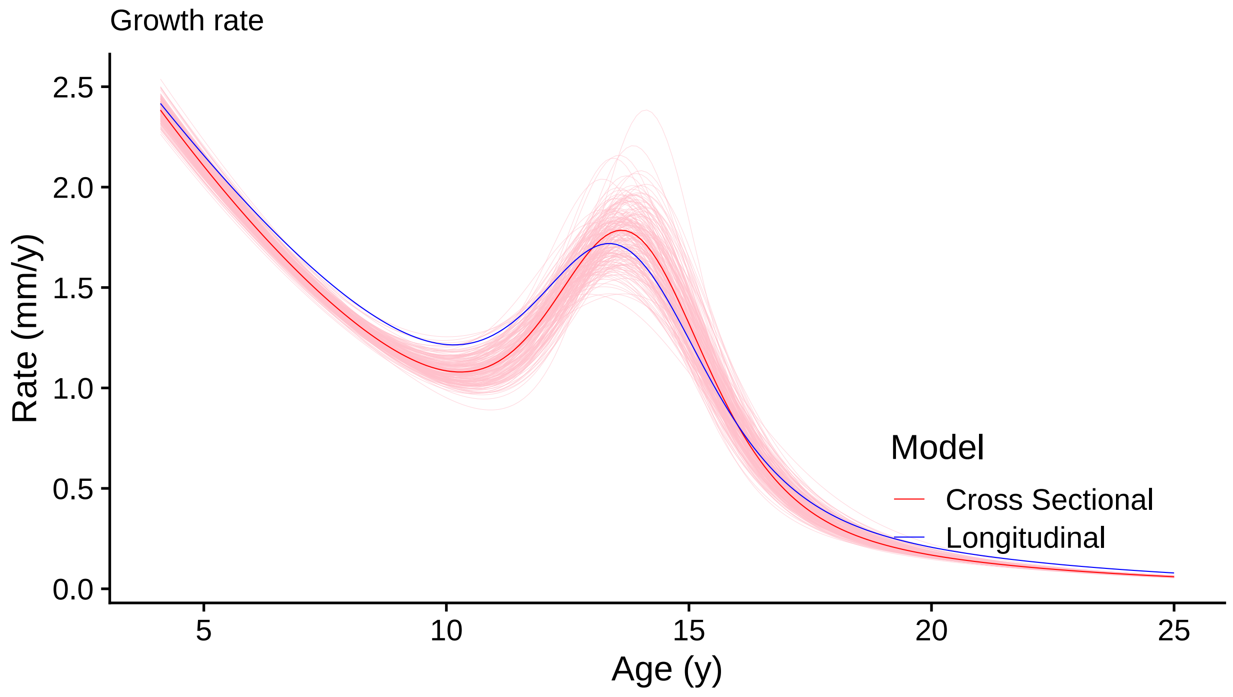
Growth rate
Rate (mm/y)
2.5
2.0
1.5
1.0
0.5
0.0
5
10
15
20
25
Age (y)
Model
Cross Sectional
Longitudinal

Table 41: Longitudinal Model Summary

Parameter	Mean	Median	Std. Dev.	MAD	5%	95%	$\hat{r}$	Bulk ESS	Tail ESS
f	107.14	107.14	0.152	0.151	106.90	107.39	1	1401	4232
a1	102.01	102.01	0.363	0.365	101.41	102.60	1	10764	19454
b1	0.18	0.18	0.005	0.005	0.17	0.19	1	7936	17141
c1	-5.38	-5.38	0.147	0.146	-5.62	-5.13	1	12719	21158
b2	0.88	0.88	0.057	0.057	0.79	0.98	1	13334	24125
c2	13.54	13.54	0.056	0.056	13.44	13.63	1	35905	27539
sigma	1.55	1.55	0.013	0.013	1.53	1.57	1	44605	31258
sigma_ID	3.82	3.82	0.089	0.089	3.68	3.97	1	46993	31316

Table 42: Median Coefficients

Model	f	a_1	b_1	c_1	b_2	c_2	σ	σ_{ID}
Longitudinal	107.14	102.01	0.18	-5.38	0.88	13.54	1.55	3.82
Cross Sectional	106.62	101.22	0.19	-5.32	0.95	13.71	4.09	NA

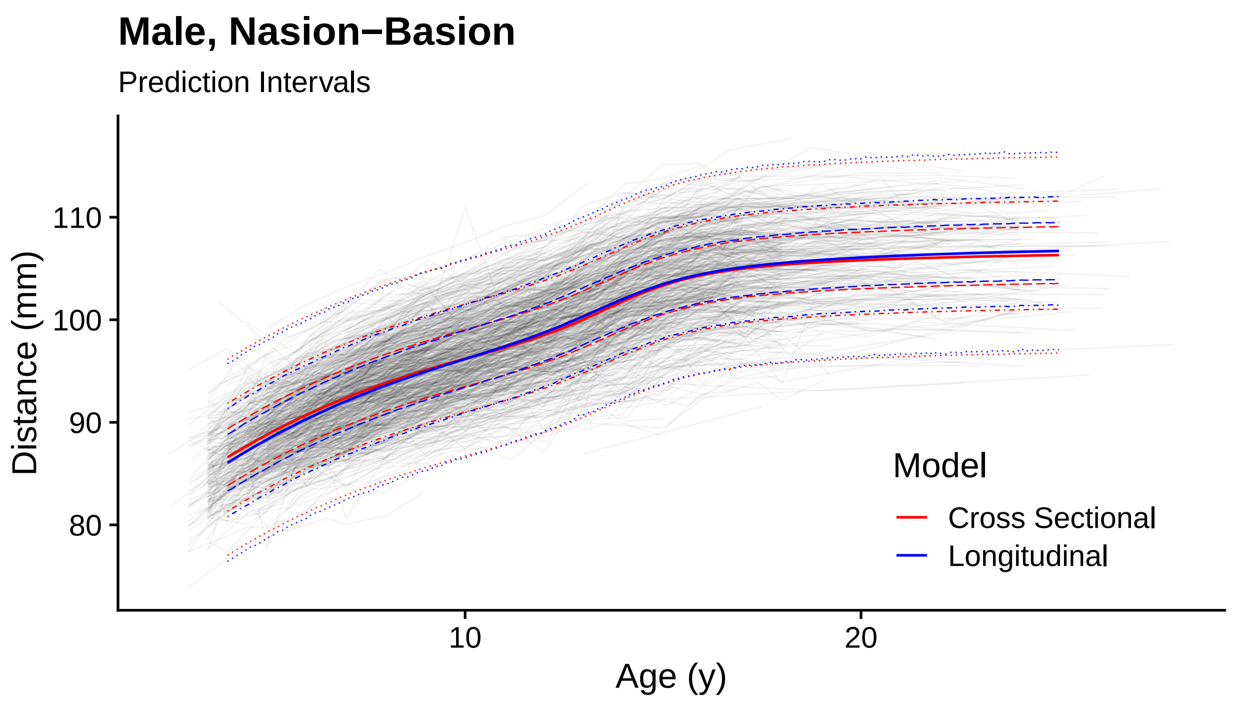
Male, Nasion−Basion
Prediction Intervals
Distance (mm)
110
100
90
80
10
20
Age (y)
Model
Cross Sectional
Longitudinal

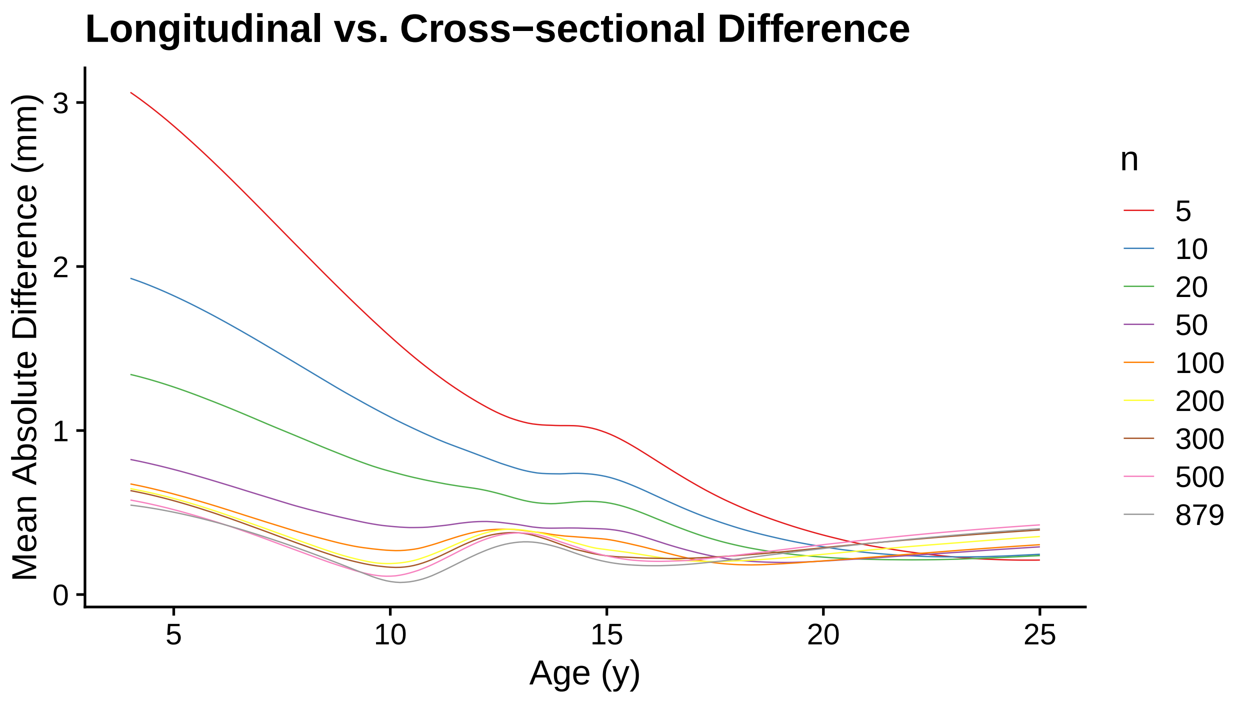
Longitudinal vs. Cross−sectional Difference
Mean Absolute Difference (mm)
3
2
1
0
5
10
15
20
25
Age (y)
n
5
10
20
50
100
200
300
500
879

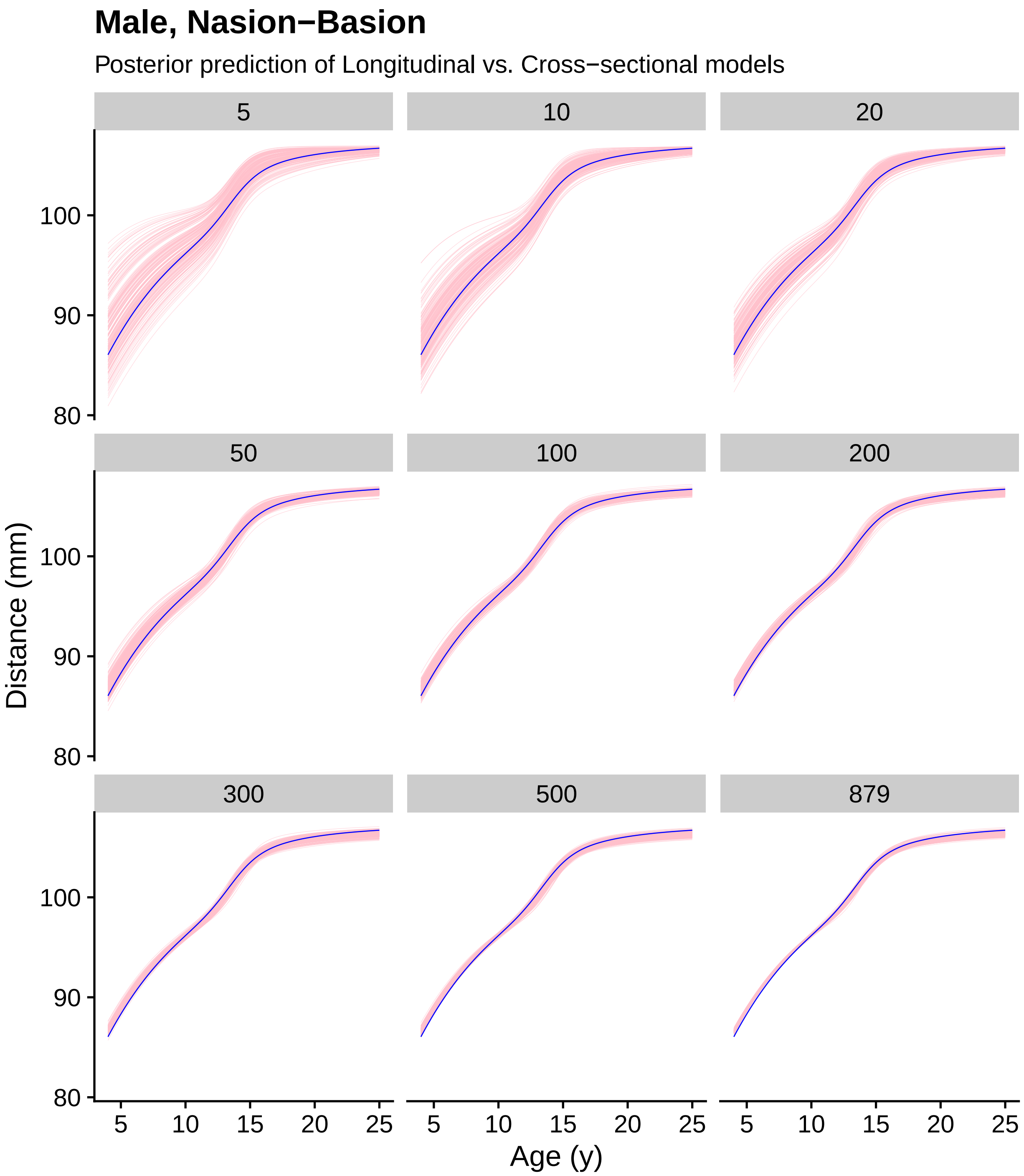

Male, Nasion−Basion
Posterior prediction of Longitudinal vs. Cross−sectional models
5
10
20
50
100
200
300
500
879
Distance (mm)
80
90
100
Age (y)
5
10
15
20
25

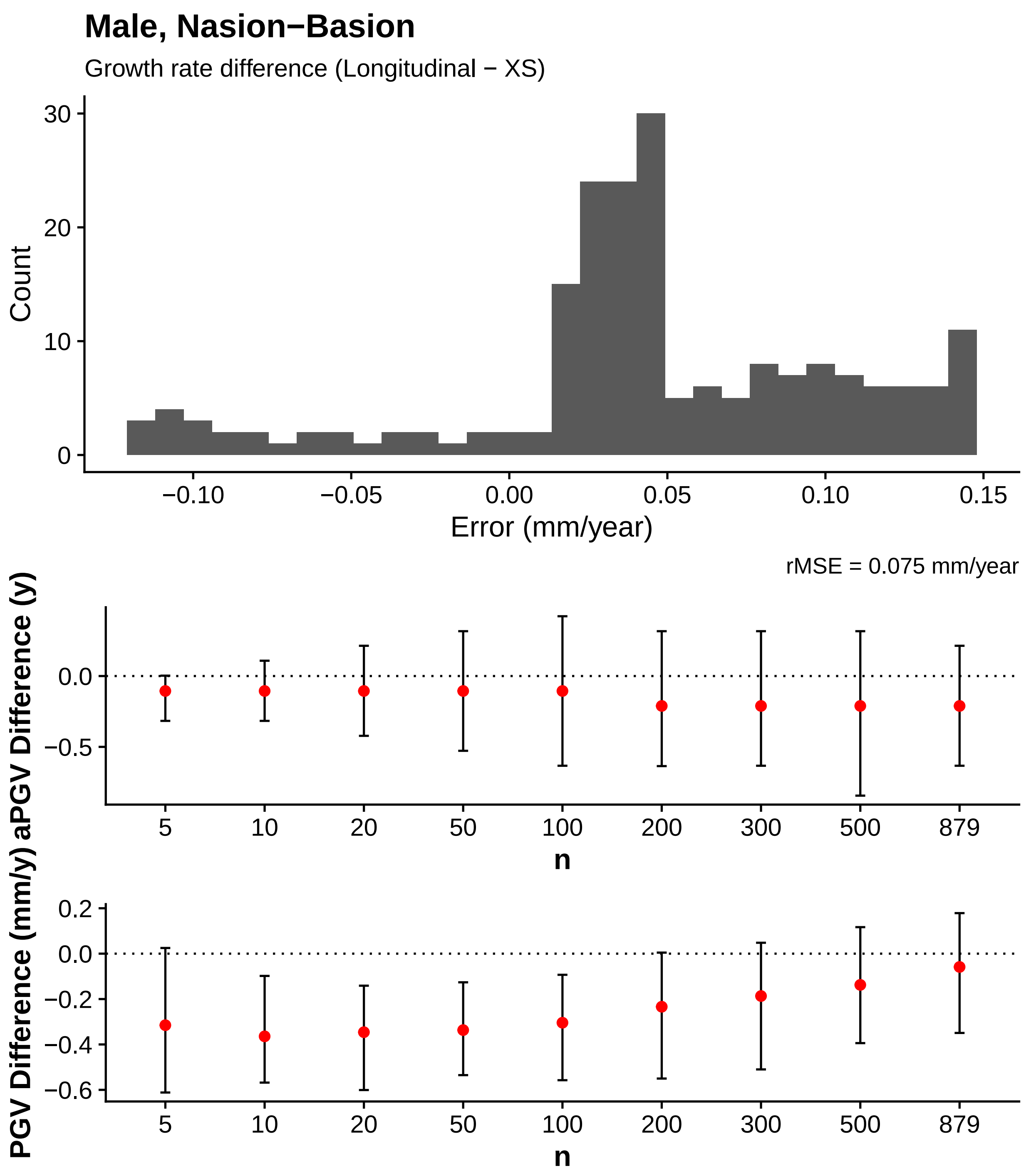
Male, Nasion−Basion
Growth rate difference (Longitudinal − XS)
Count
30
20
10
0
−0.10
−0.05
0.00
0.05
0.10
0.15
Error (mm/year)
rMSE = 0.075 mm/year
PGV Difference (mm/y) aPGV Difference (y)
0.0
−0.5
5
10
20
50
100
200
300
500
879
n
0.2
0.0
−0.2
−0.4
−0.6
n

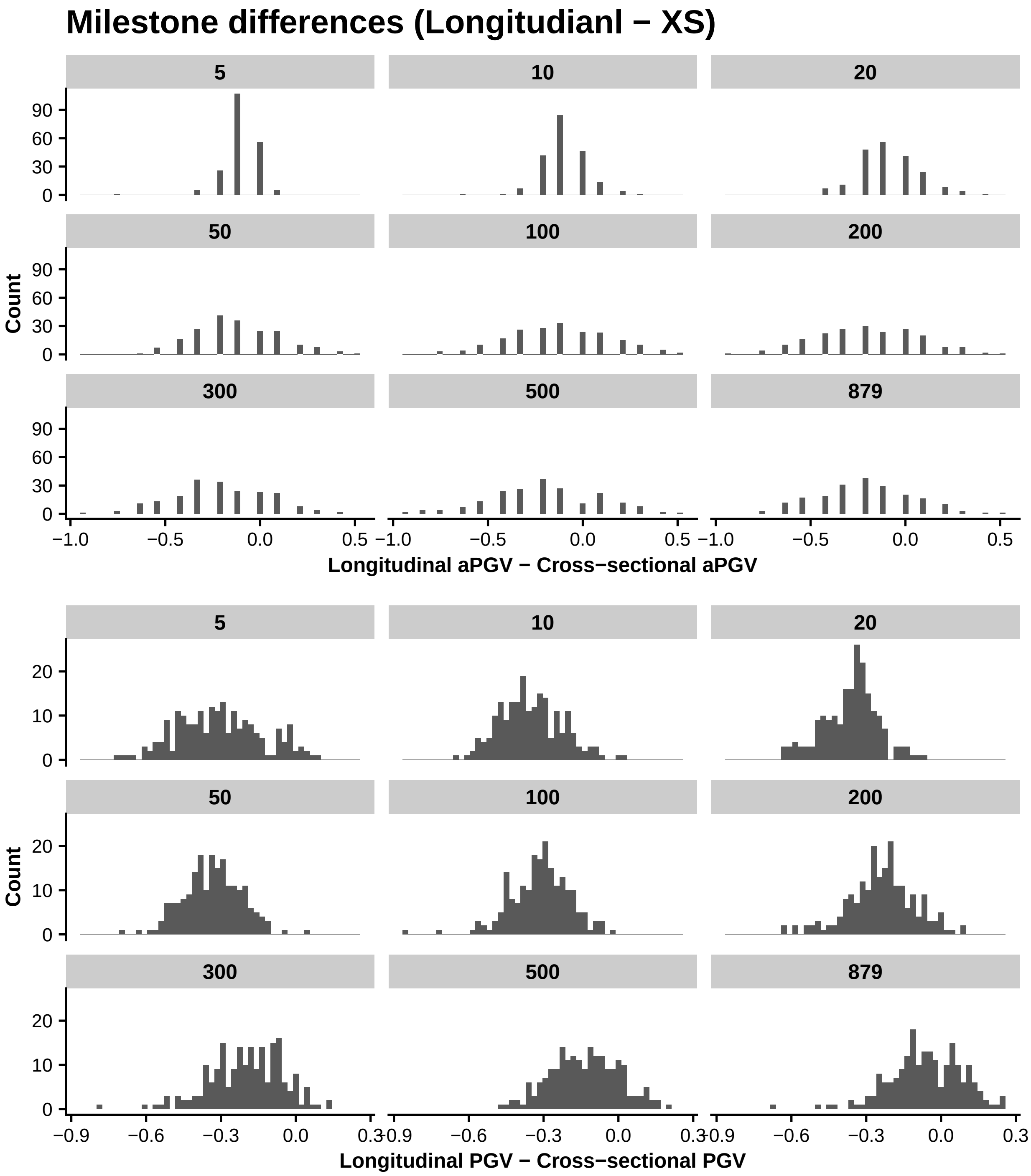
Milestone differences (Longitudianl − XS)
5
10
20
50
100
200
300
500
879
Count
90
60
30
0
−1.0
−0.5
0.0
0.5
Longitudinal aPGV − Cross−sectional aPGV
20
10
0
−0.9
−0.6
−0.3
0.0
0.3
Longitudinal PGV − Cross−sectional PGV

23 Male, Nasion-Menton

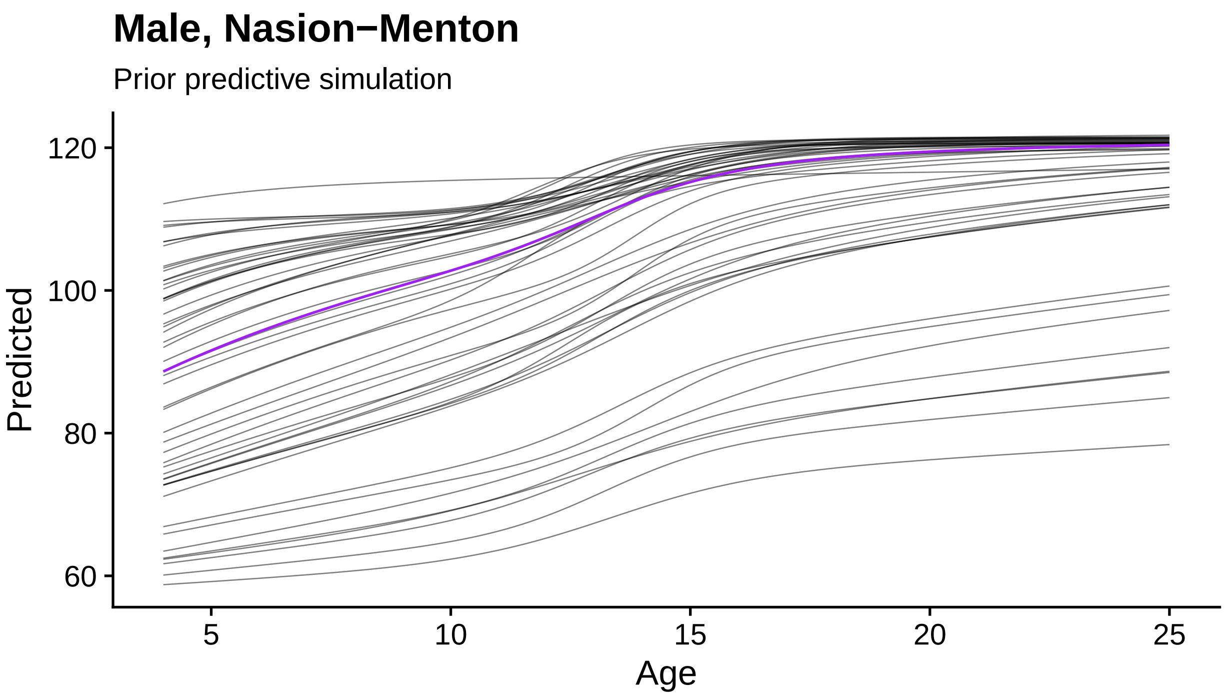

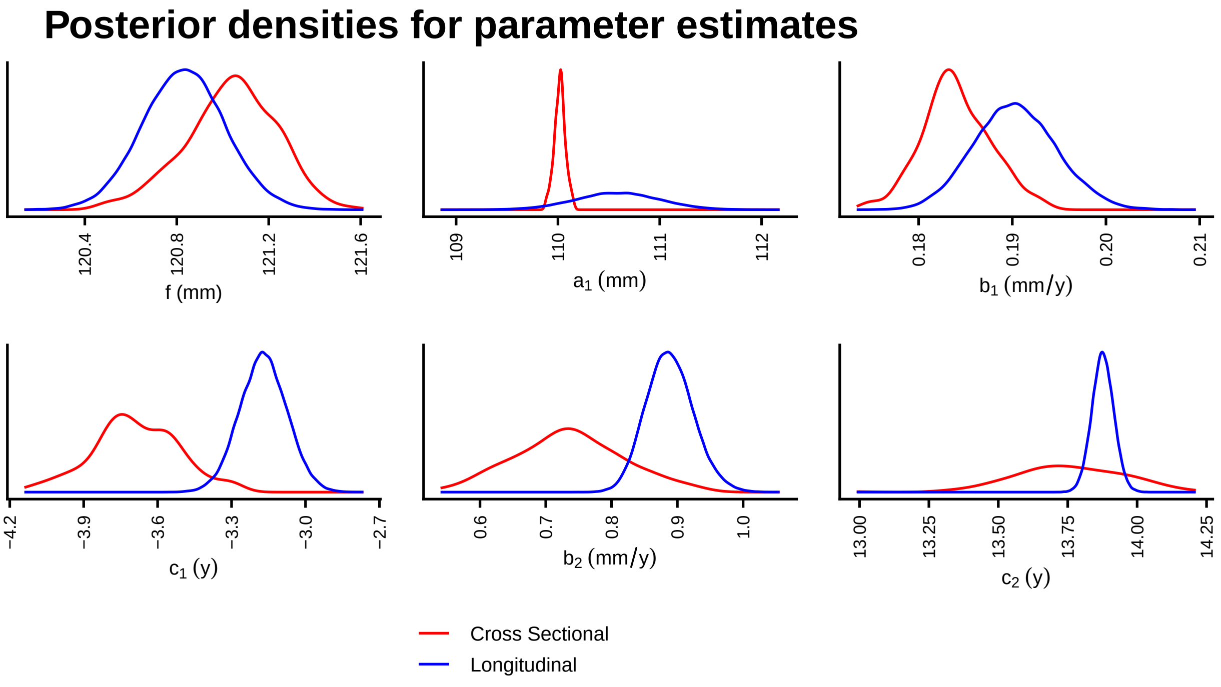

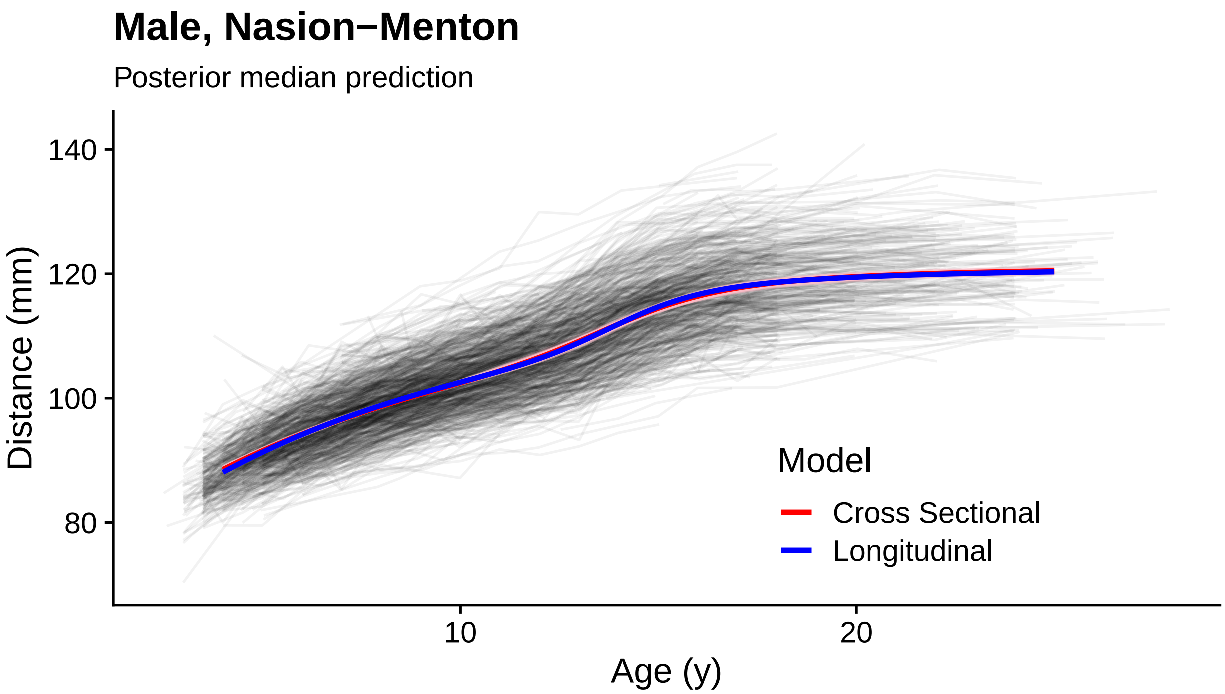
Male, Nasion−Menton
Posterior median prediction
Distance (mm)
140
120
100
80
10
20
Age (y)
Model
Cross Sectional
Longitudinal

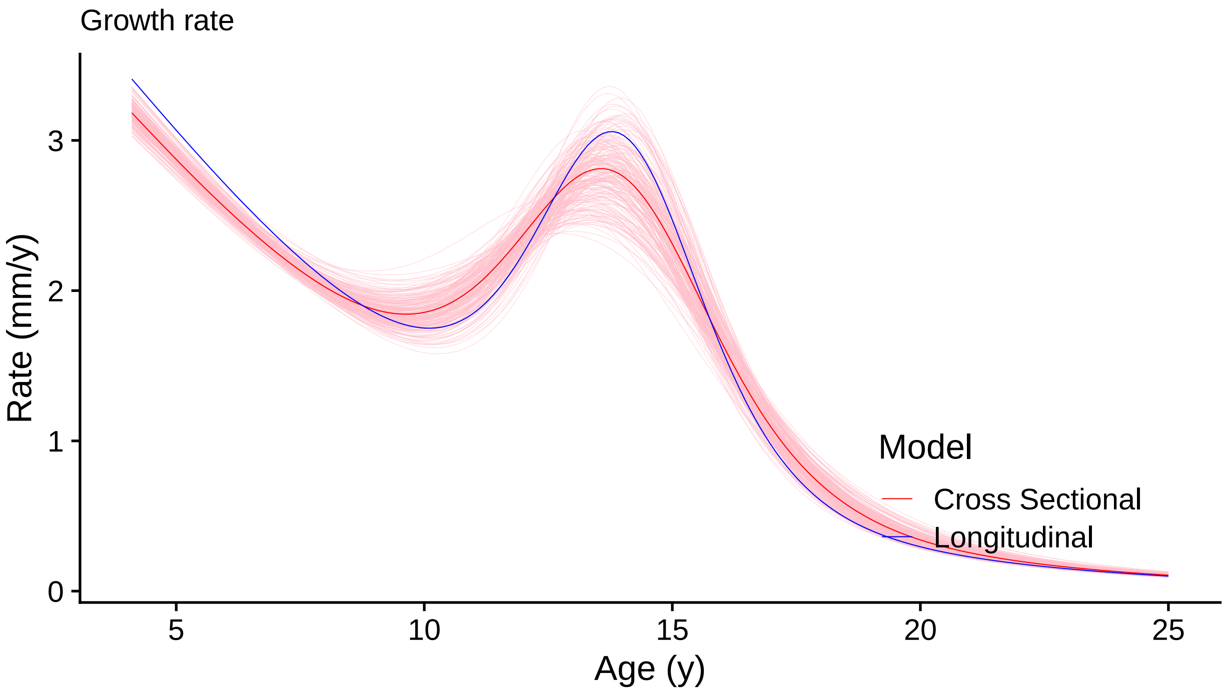
Growth rate
Rate (mm/y)
3
2
1
0
5
10
15
20
25
Age (y)
Model
Cross Sectional
Longitudinal

Table 43: Longitudinal Model Summary

Parameter	Mean	Median	Std. Dev.	MAD	5%	95%	$\hat{r}$	Bulk ESS	Tail ESS
f	120.83	120.83	0.181	0.179	120.53	121.13	1	3363	8886
a1	110.59	110.59	0.408	0.406	109.91	111.26	1	26639	29659
b1	0.19	0.19	0.004	0.004	0.18	0.20	1	12544	22848
c1	-3.17	-3.17	0.096	0.096	-3.33	-3.01	1	30703	29513
b2	0.89	0.89	0.036	0.036	0.83	0.95	1	24039	30690
c2	13.88	13.88	0.039	0.039	13.81	13.94	1	65401	29744
sigma	2.18	2.18	0.018	0.018	2.15	2.21	1	65172	31242
sigma_ID	4.90	4.90	0.114	0.115	4.72	5.09	1	84282	29347

Table 44: Median Coefficients

Model	f	a_1	b_1	c_1	b_2	c_2	σ	σ_{ID}
Longitudinal	120.83	110.59	0.19	-3.17	0.89	13.88	2.18	4.9
Cross Sectional	121.05	110.02	0.18	-3.70	0.74	13.76	5.35	NA

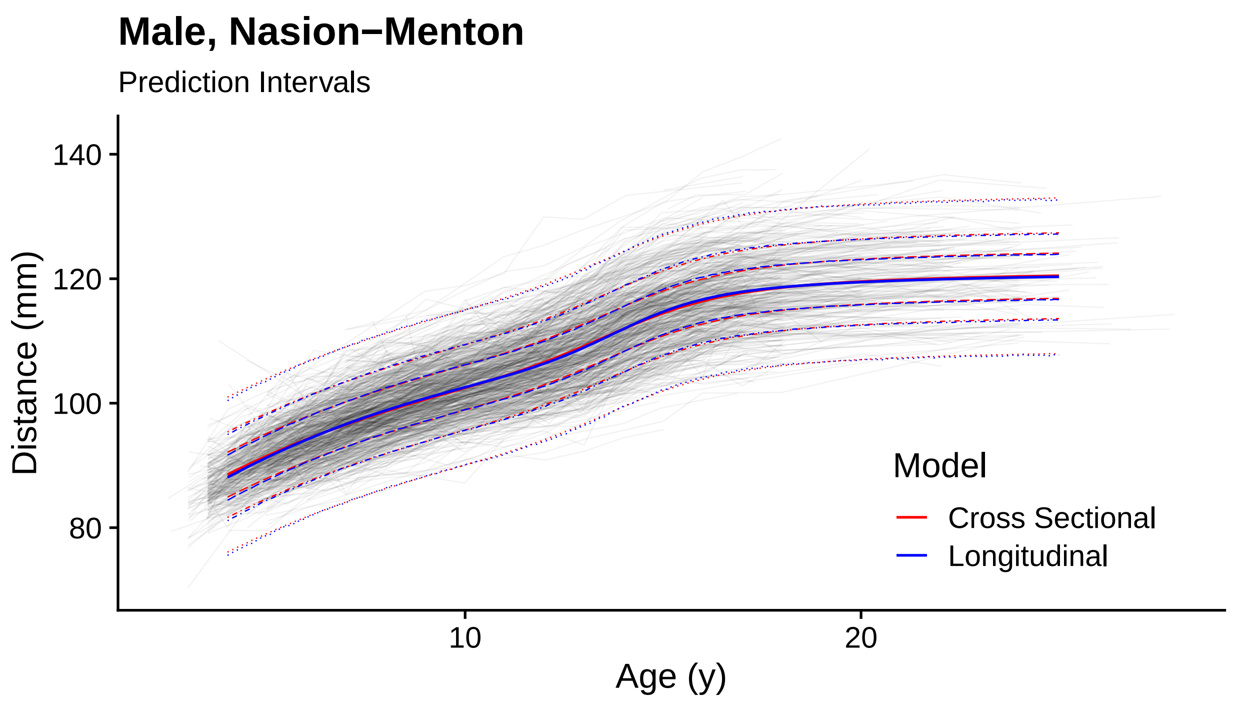
Male, Nasion−Menton
Prediction Intervals
Distance (mm)
140
120
100
80
10
20
Age (y)
Model
Cross Sectional
Longitudinal

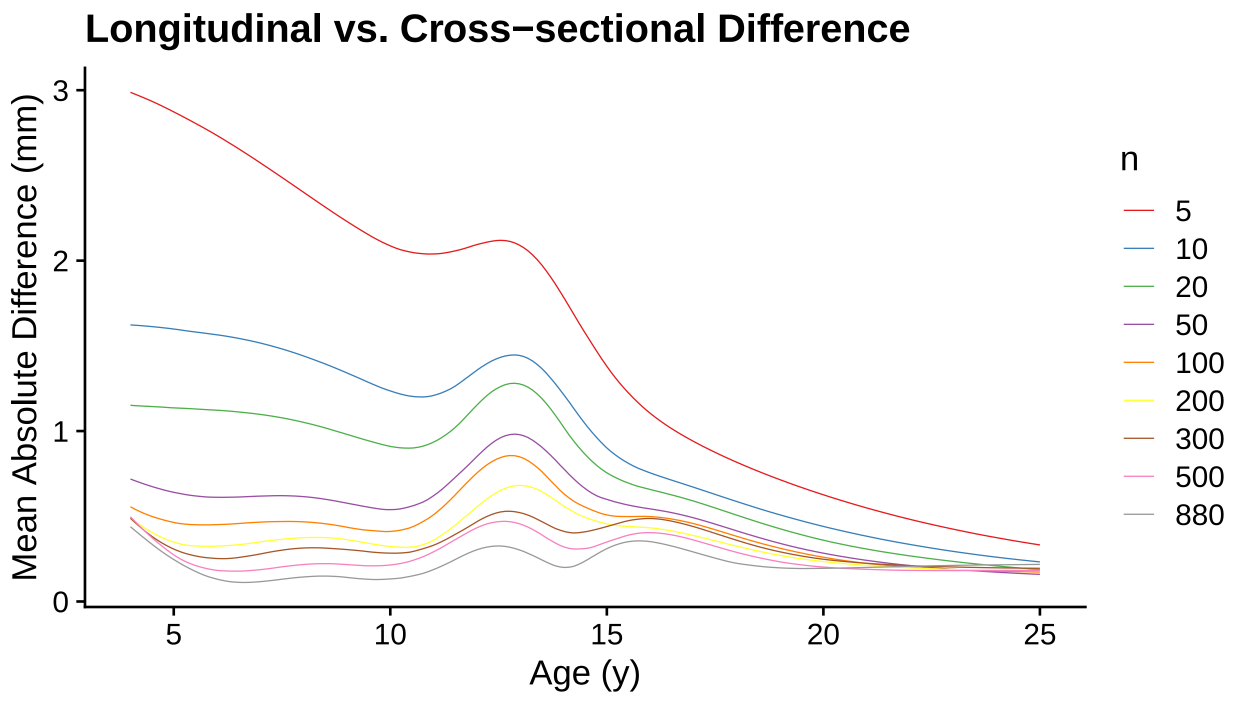
Longitudinal vs. Cross−sectional Difference
Mean Absolute Difference (mm)
3
2
1
0
5
10
15
20
25
Age (y)
n
5
10
20
50
100
200
300
500
880

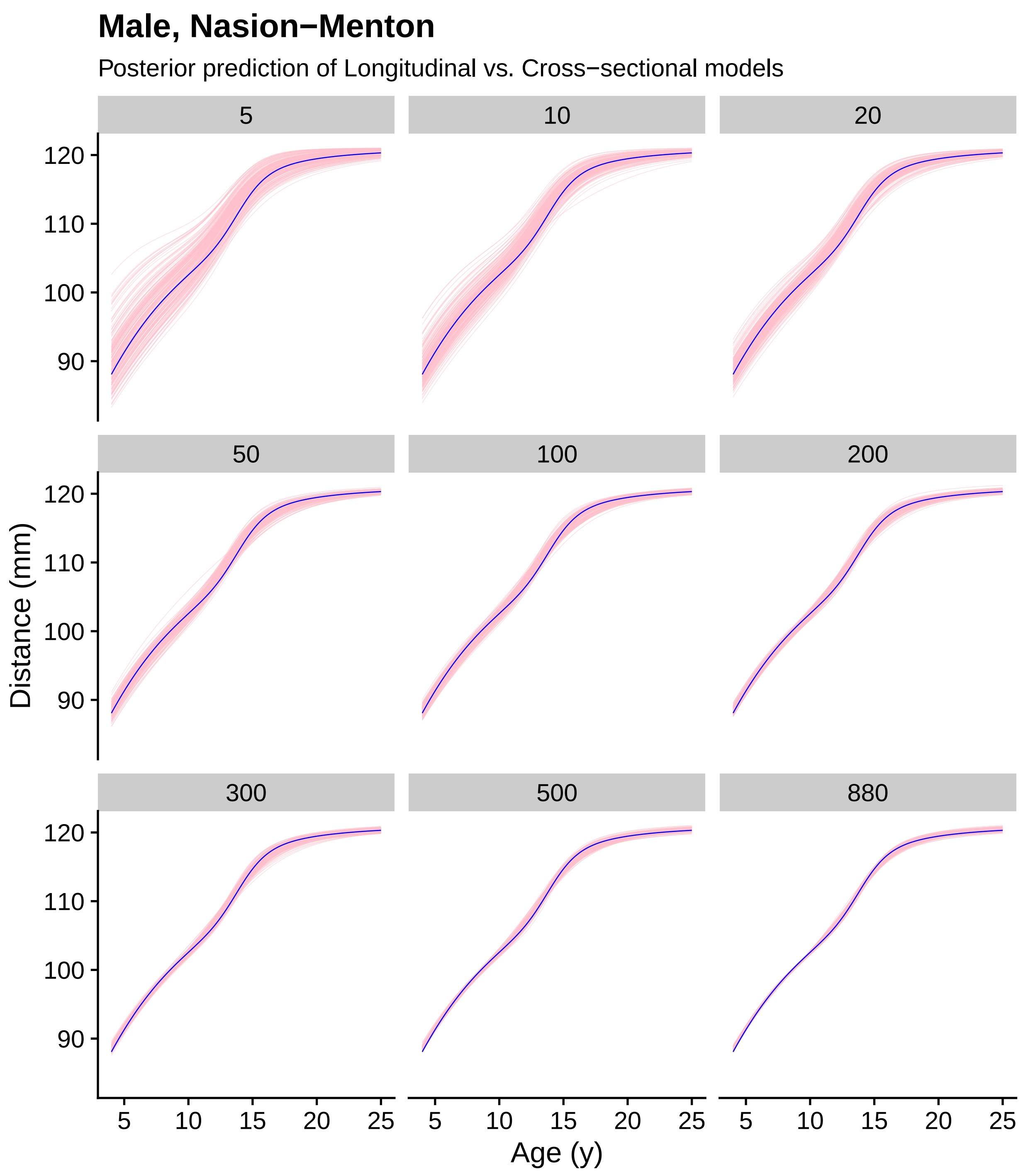
Male, Nasion−Menton
Posterior prediction of Longitudinal vs. Cross−sectional models
5
10
20
50
100
200
300
500
880
Distance (mm)
Age (y)

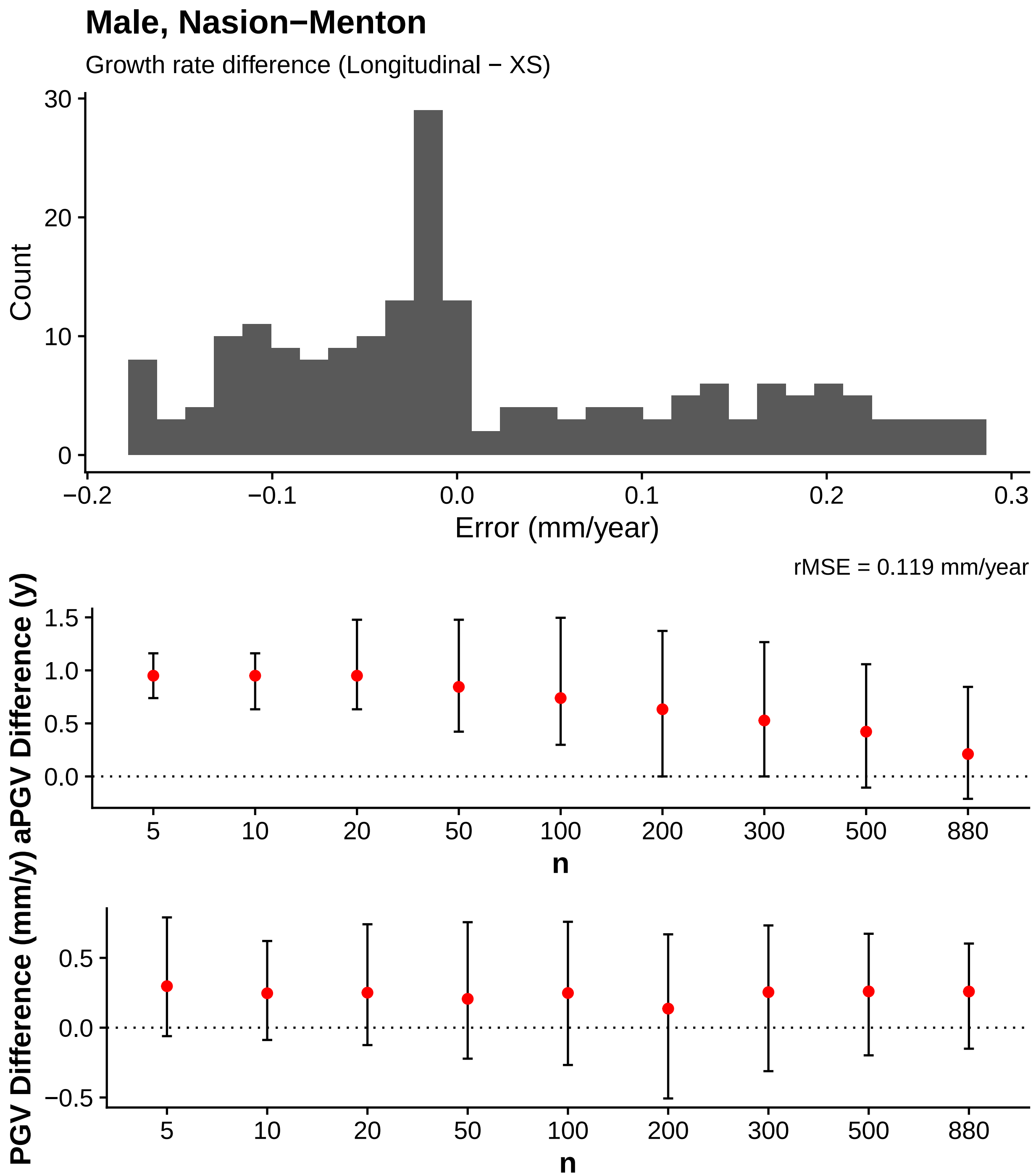
Male, Nasion−Menton
Growth rate difference (Longitudinal − XS)
Count
Error (mm/year)
rMSE = 0.119 mm/year
PGV Difference (mm/y) aPGV Difference (y)
n
n

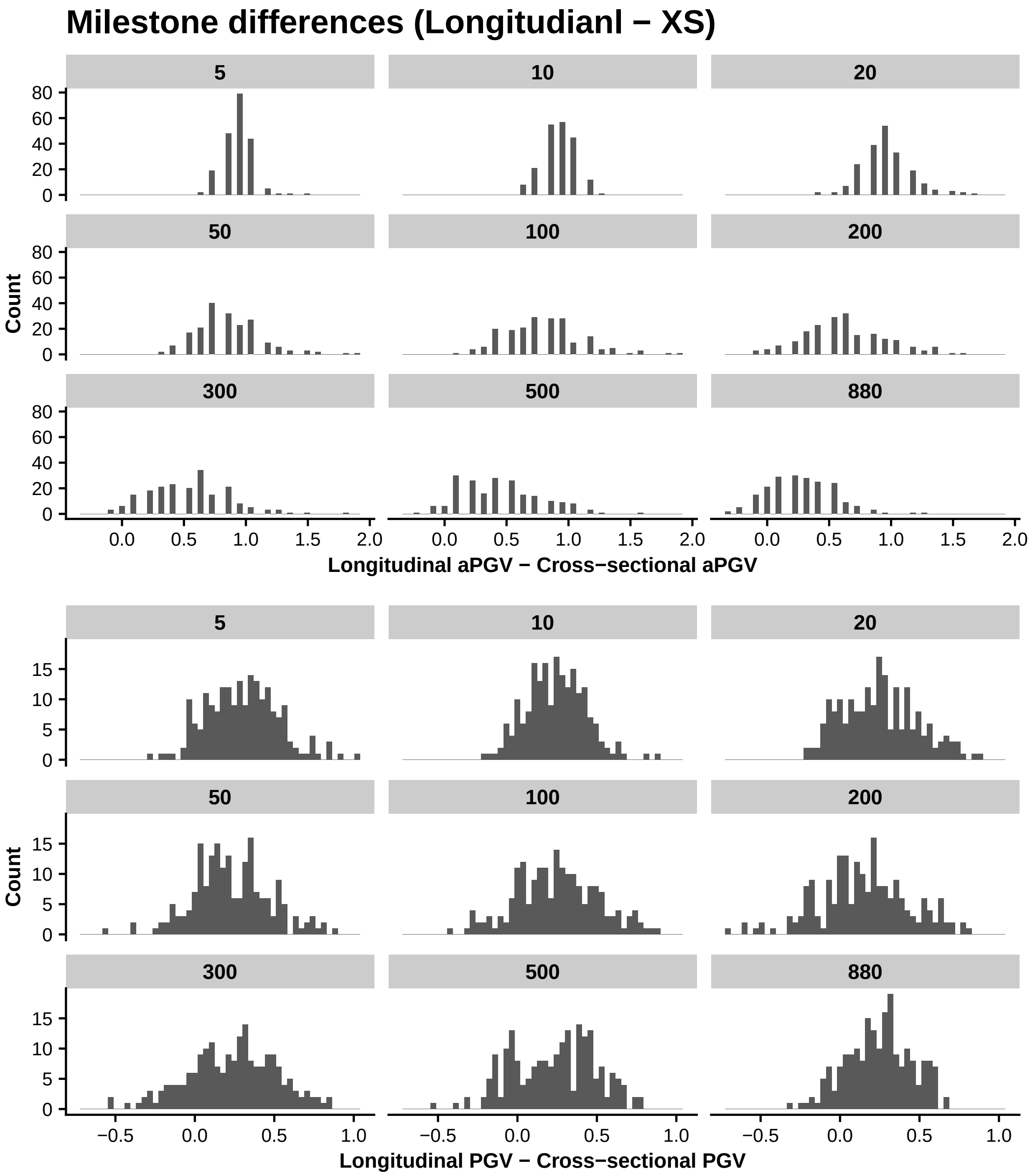
Milestone differences (Longitudianl − XS)
5
10
20
50
100
200
300
500
880
Count
0
20
40
60
80
0.0
0.5
1.0
1.5
2.0
Longitudinal aPGV − Cross−sectional aPGV
0
5
10
15
−0.5
0.0
0.5
1.0
Longitudinal PGV − Cross−sectional PGV

24 Male, Sella-Basion

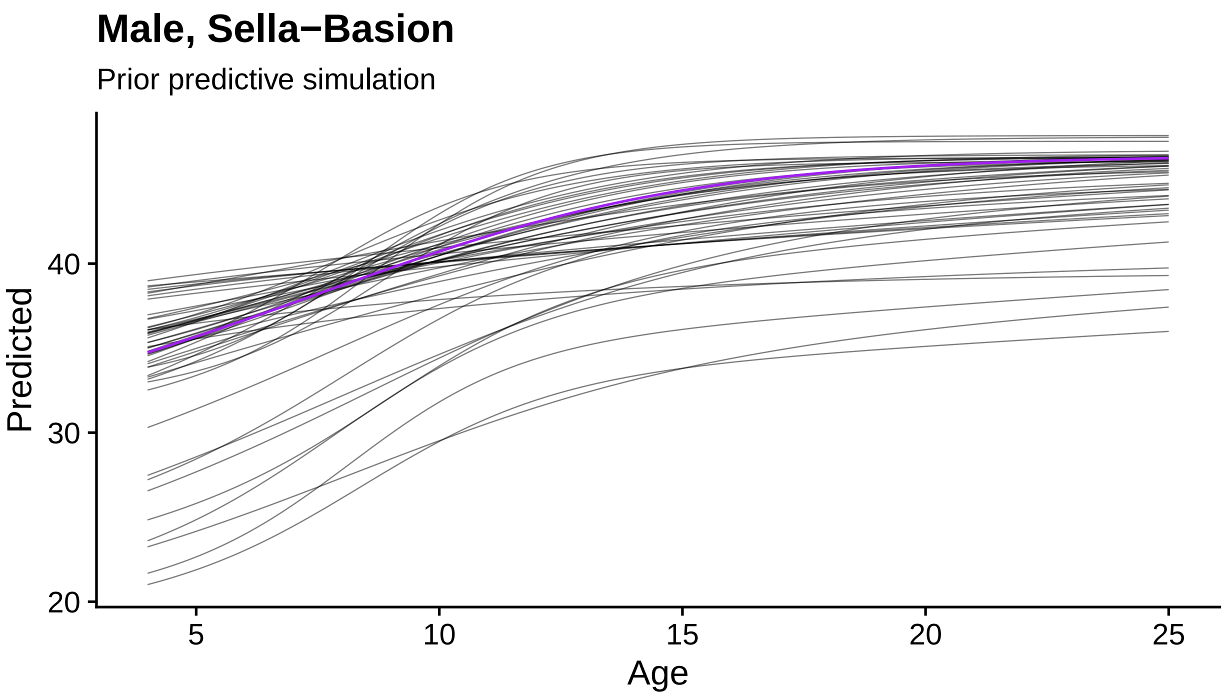

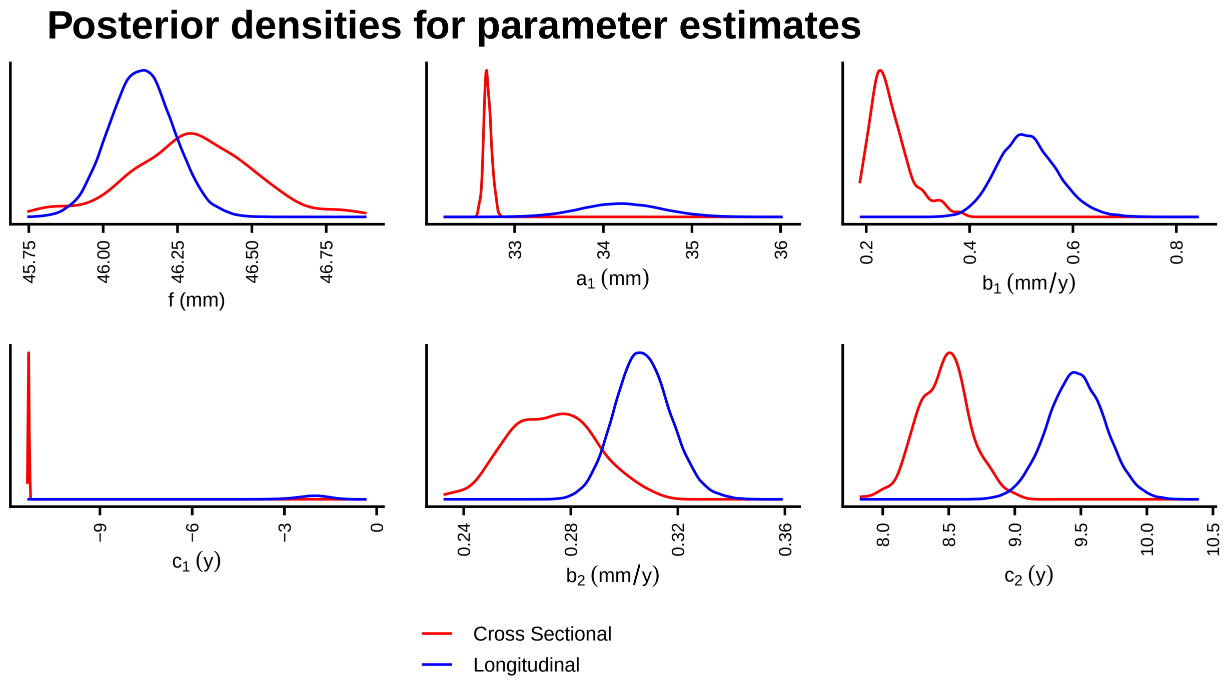

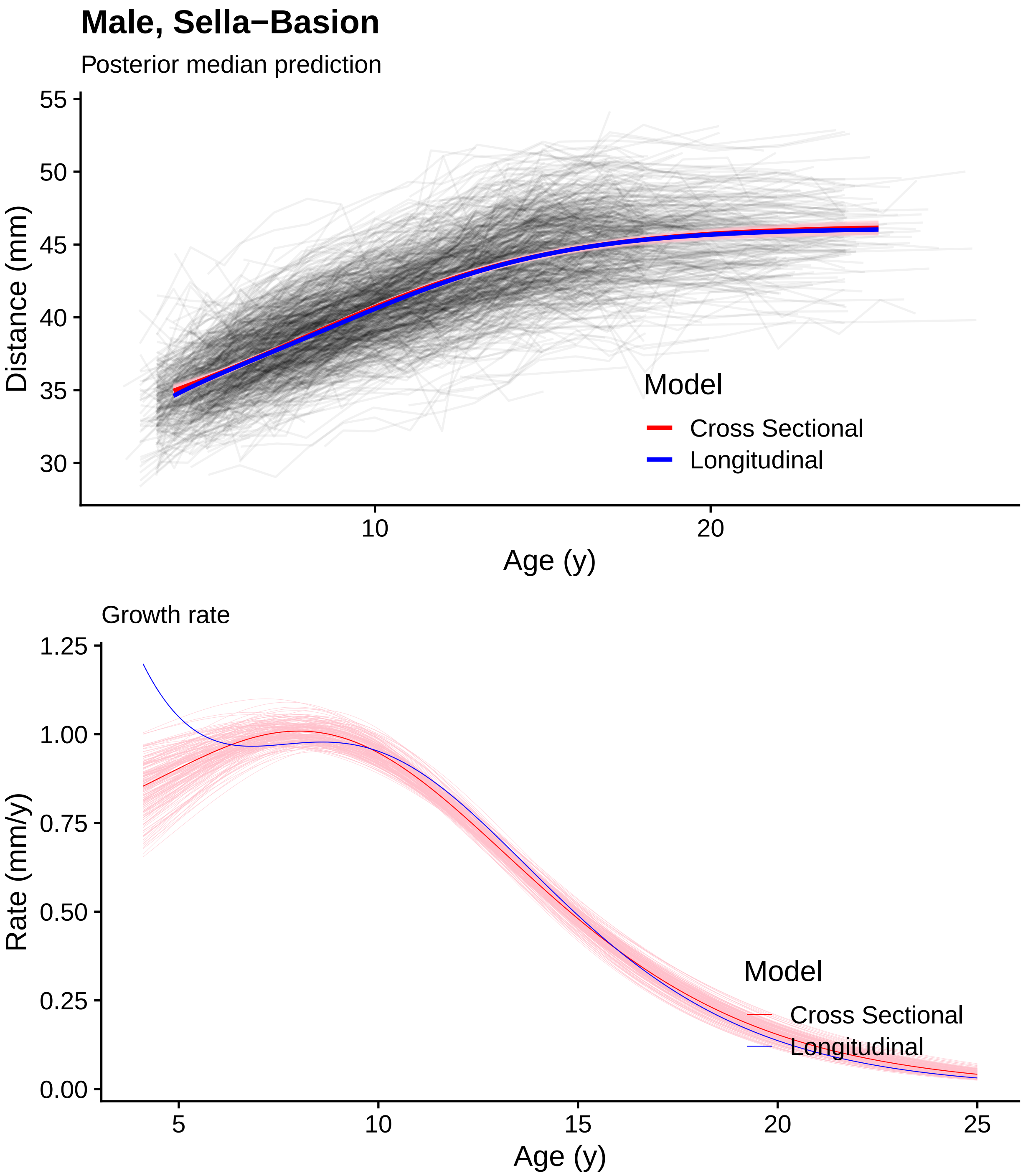

Male, Sella−Basion
Posterior median prediction
Distance (mm)
Age (y)
Model
Cross Sectional
Longitudinal
Growth rate
Rate (mm/y)
Age (y)
Model
Cross Sectional
Longitudinal

Table 45: Longitudinal Model Summary

Parameter	Mean	Median	Std. Dev.	MAD	5%	95%	$\hat{r}$	Bulk ESS	Tail ESS
f	46.13	46.13	0.107	0.107	45.96	46.31	1	2652	6944
a1	34.21	34.20	0.452	0.453	33.46	34.95	1	18487	24351
b1	0.51	0.51	0.058	0.057	0.42	0.61	1	24444	24102
c1	-2.12	-2.08	0.537	0.514	-3.07	-1.31	1	26952	21228
b2	0.31	0.31	0.010	0.010	0.29	0.32	1	17808	25158
c2	9.47	9.47	0.217	0.218	9.11	9.82	1	16431	24216
sigma	1.24	1.24	0.010	0.010	1.22	1.26	1	52309	29730
sigma_ID	2.33	2.33	0.055	0.055	2.25	2.43	1	60798	31374

Table 46: Median Coefficients

Model	f	a_1	b_1	c_1	b_2	c_2	σ	σ_{ID}
Longitudinal	46.13	34.20	0.51	-2.08	0.31	9.47	1.24	2.33
Cross Sectional	46.31	32.69	0.24	-11.32	0.27	8.47	2.63	NA

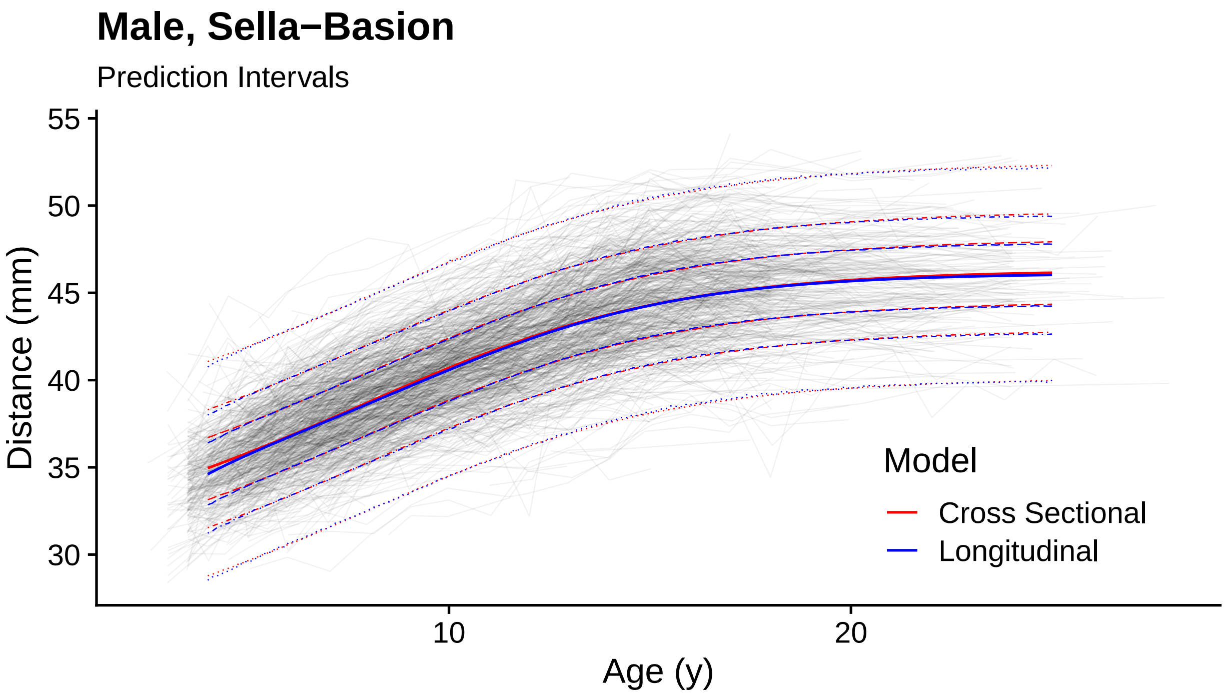
Male, Sella−Basion
Prediction Intervals
Distance (mm)
55
50
45
40
35
30
10
20
Age (y)
Model
Cross Sectional
Longitudinal

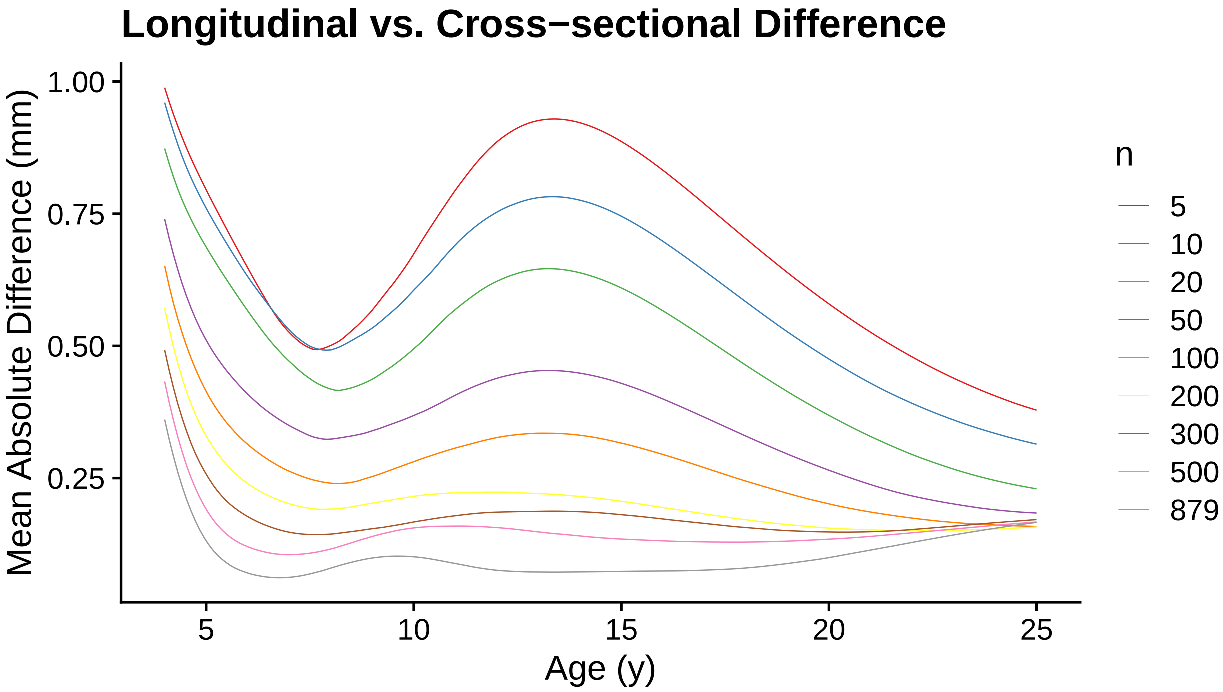
Longitudinal vs. Cross−sectional Difference
Mean Absolute Difference (mm)
1.00
0.75
0.50
0.25
5
10
15
20
25
Age (y)
n
5
10
20
50
100
200
300
500
879

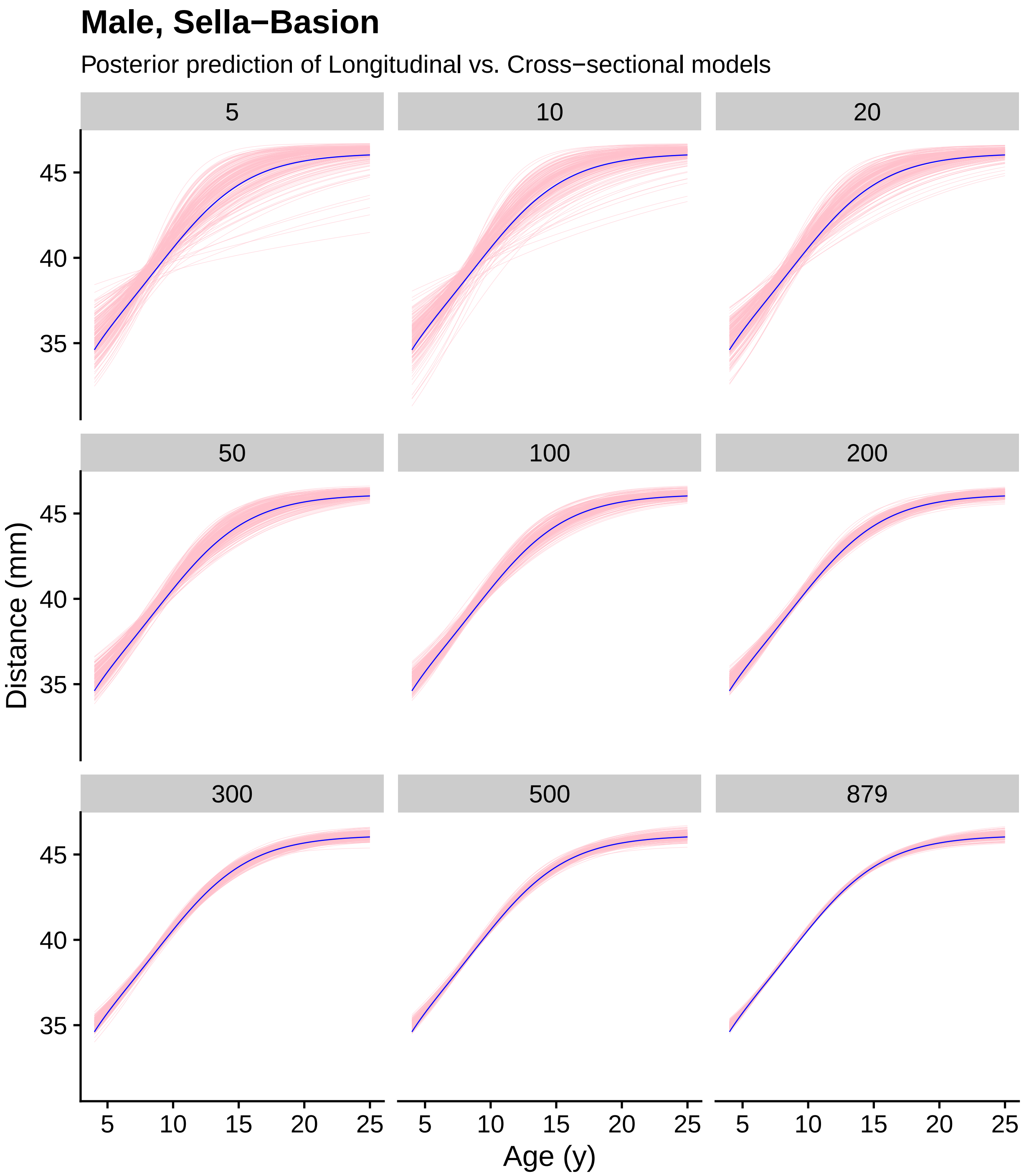

Male, Sella−Basion
Posterior prediction of Longitudinal vs. Cross−sectional models
5
10
20
50
100
200
300
500
879
45
40
35
Distance (mm)
5
10
15
20
25
Age (y)

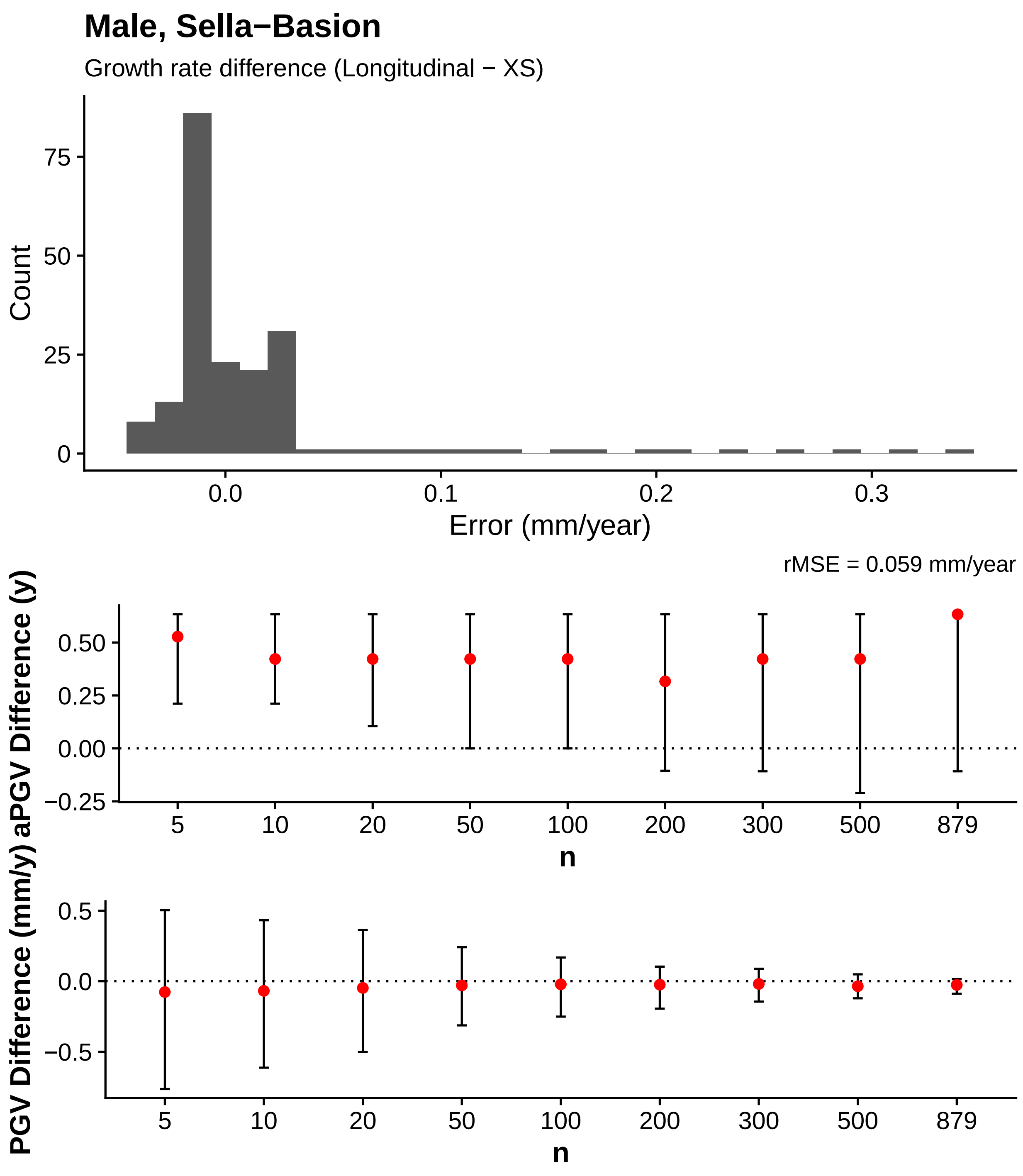

Male, Sella−Basion
Growth rate difference (Longitudinal − XS)
Count
75
50
25
0
0.0
0.1
0.2
0.3
Error (mm/year)
rMSE = 0.059 mm/year
PGV Difference (mm/y) aPGV Difference (y)
0.50
0.25
0.00
−0.25
5
10
20
50
100
200
300
500
879
n
0.5
0.0
−0.5
5
10
20
50
100
200
300
500
879
n

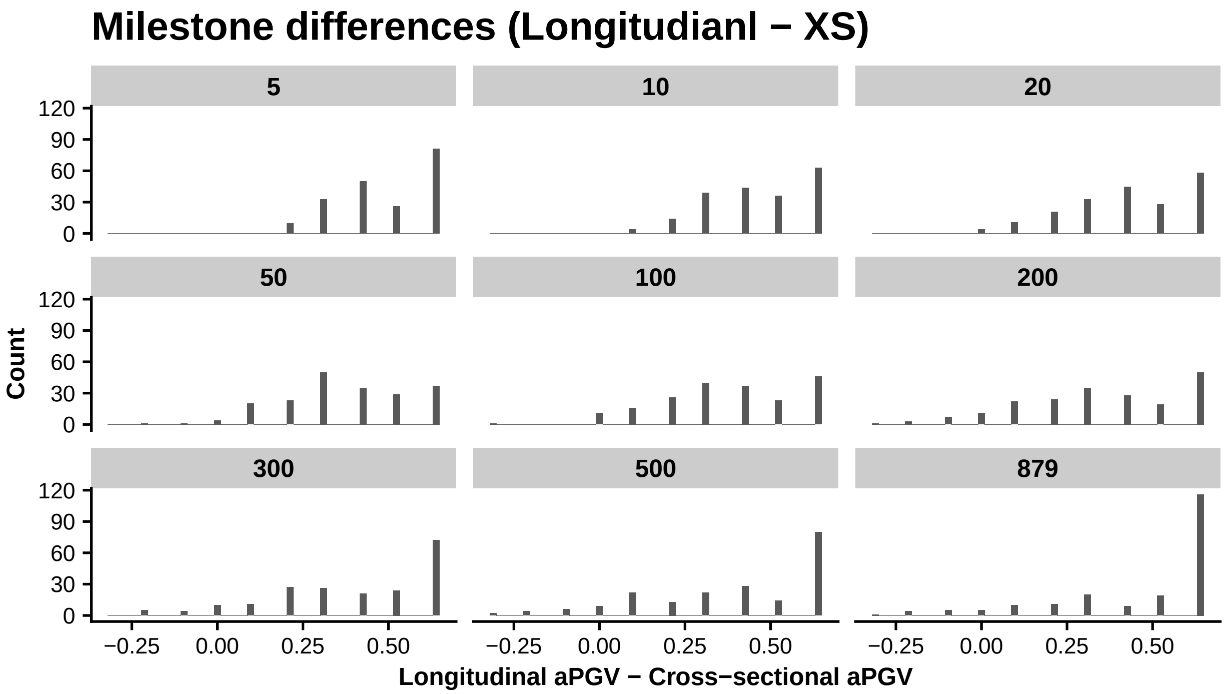
Milestone differences (Longitudianl − XS)
5
10
20
50
100
200
300
500
879
Count
0
30
60
90
120
−0.25
0.00
0.25
0.50
Longitudinal aPGV − Cross−sectional aPGV

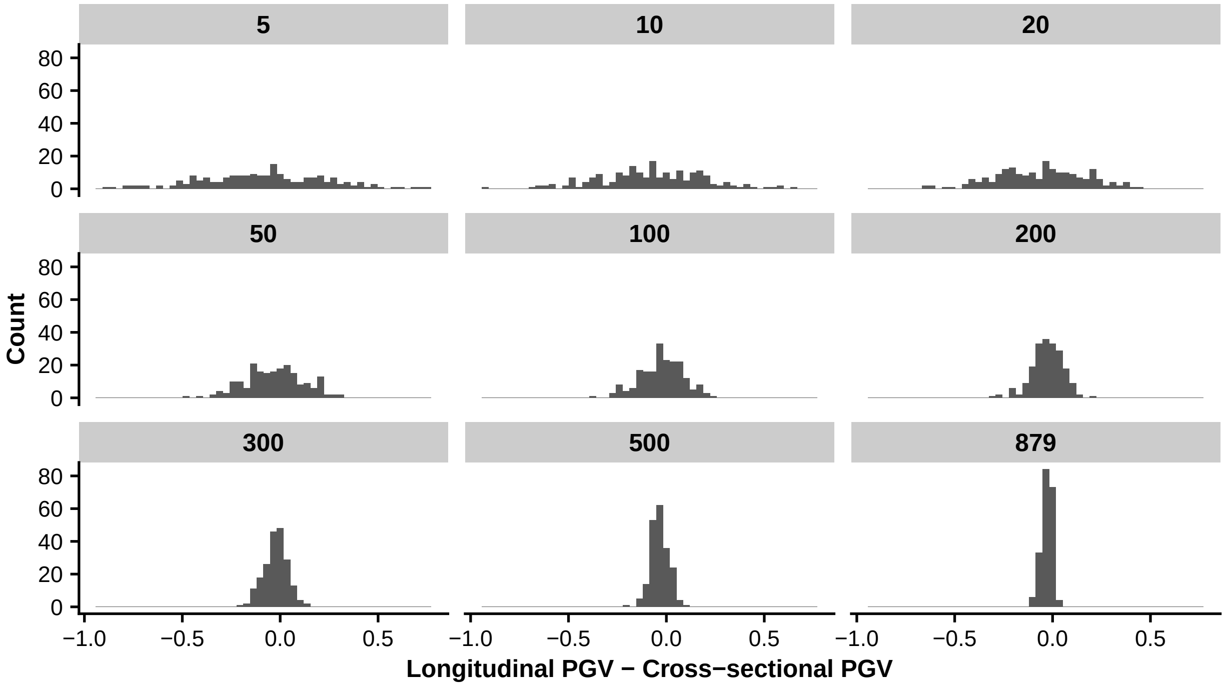
5
10
20
50
100
200
300
500
879
Count
0
20
40
60
80
−1.0
−0.5
0.0
0.5
Longitudinal PGV − Cross−sectional PGV

25 Male, Sella-Gonion

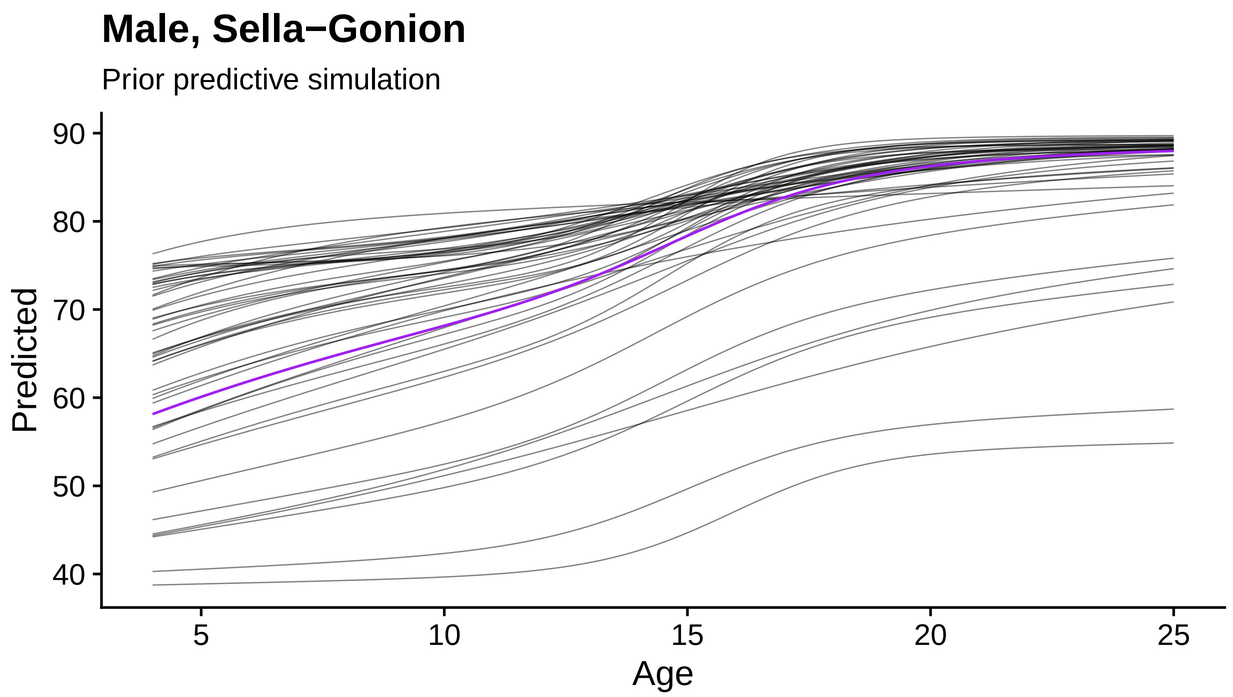

Posterior densities for parameter estimates

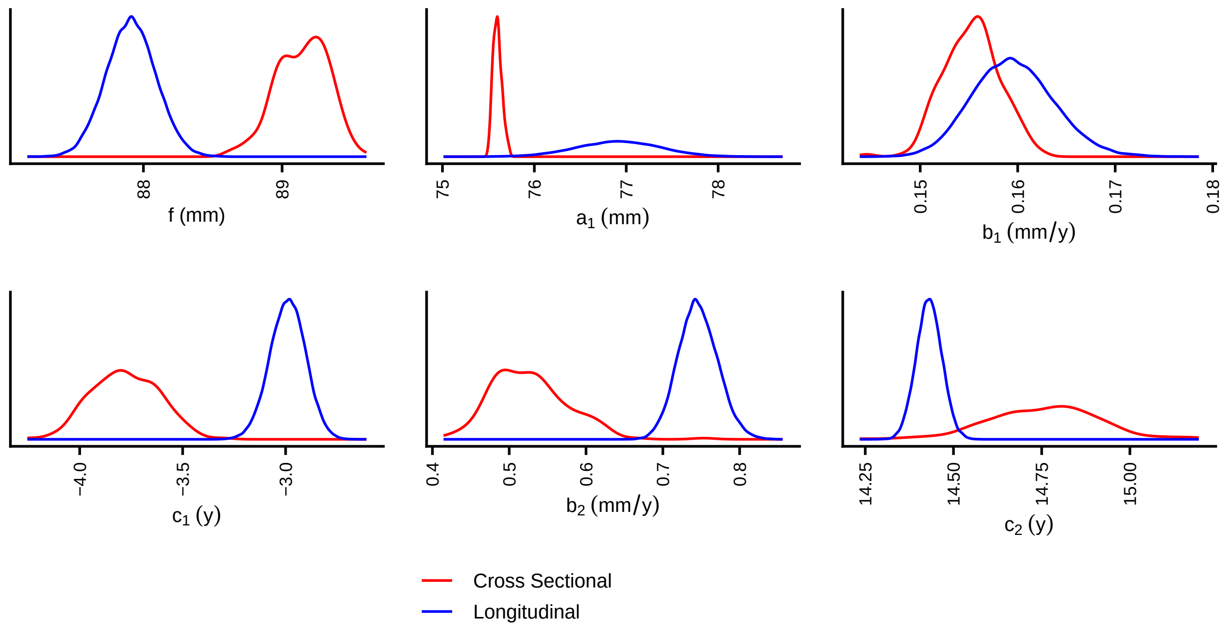

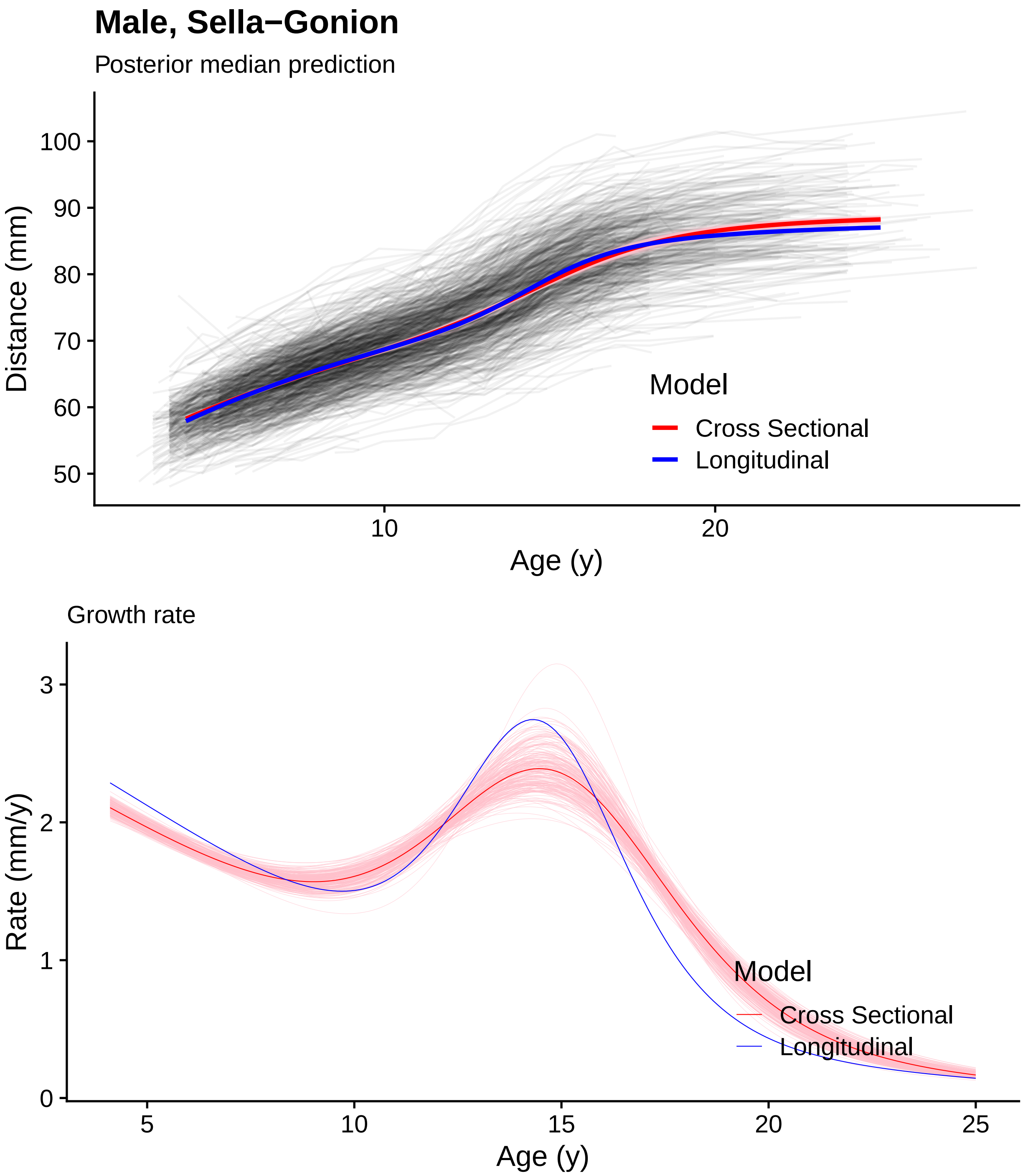
Male, Sella−Gonion
Posterior median prediction
Distance (mm)
100
90
80
70
60
50
10
20
Age (y)
Model
Cross Sectional
Longitudinal
Growth rate
Rate (mm/y)
3
2
1
0
5
10
15
20
25
Age (y)
Model
Cross Sectional
Longitudinal

Table 47: Longitudinal Model Summary

Parameter	Mean	Median	Std. Dev.	MAD	5%	95%	$\hat{r}$	Bulk ESS	Tail ESS
f	87.91	87.91	0.180	0.179	87.62	88.21	1	3701	9582
a1	76.91	76.92	0.451	0.456	76.17	77.65	1	21184	25234
b1	0.16	0.16	0.004	0.004	0.15	0.17	1	9713	18440
c1	-2.99	-2.99	0.087	0.087	-3.13	-2.85	1	32817	30432
b2	0.75	0.75	0.026	0.026	0.70	0.79	1	16693	27360
c2	14.43	14.43	0.036	0.036	14.37	14.49	1	52158	30894
sigma	1.70	1.70	0.014	0.014	1.67	1.72	1	58341	30722
sigma_ID	4.14	4.14	0.096	0.095	3.98	4.30	1	70970	31166

Table 48: Median Coefficients

Model	f	a_1	b_1	c_1	b_2	c_2	σ	σ_{ID}
Longitudinal	87.91	76.92	0.16	-2.99	0.75	14.43	1.70	4.14
Cross Sectional	89.16	75.59	0.16	-3.78	0.52	14.77	4.43	NA

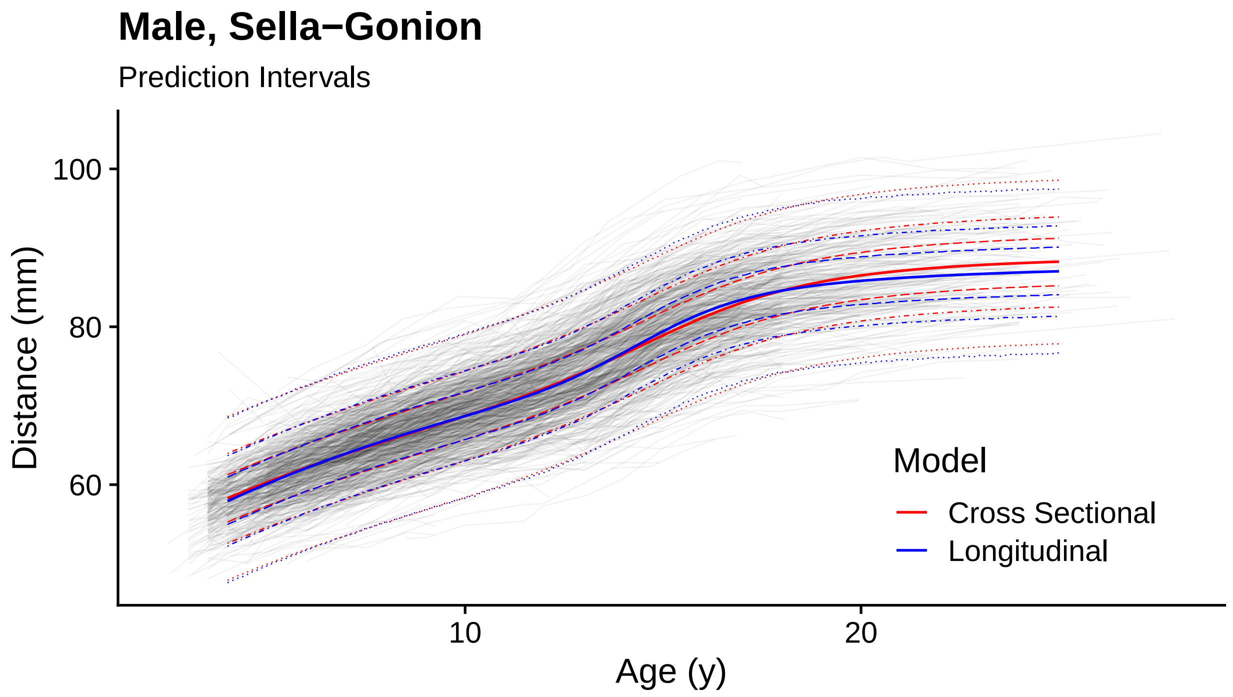
Male, Sella−Gonion
Prediction Intervals
Distance (mm)
100
80
60
10
20
Age (y)
Model
Cross Sectional
Longitudinal

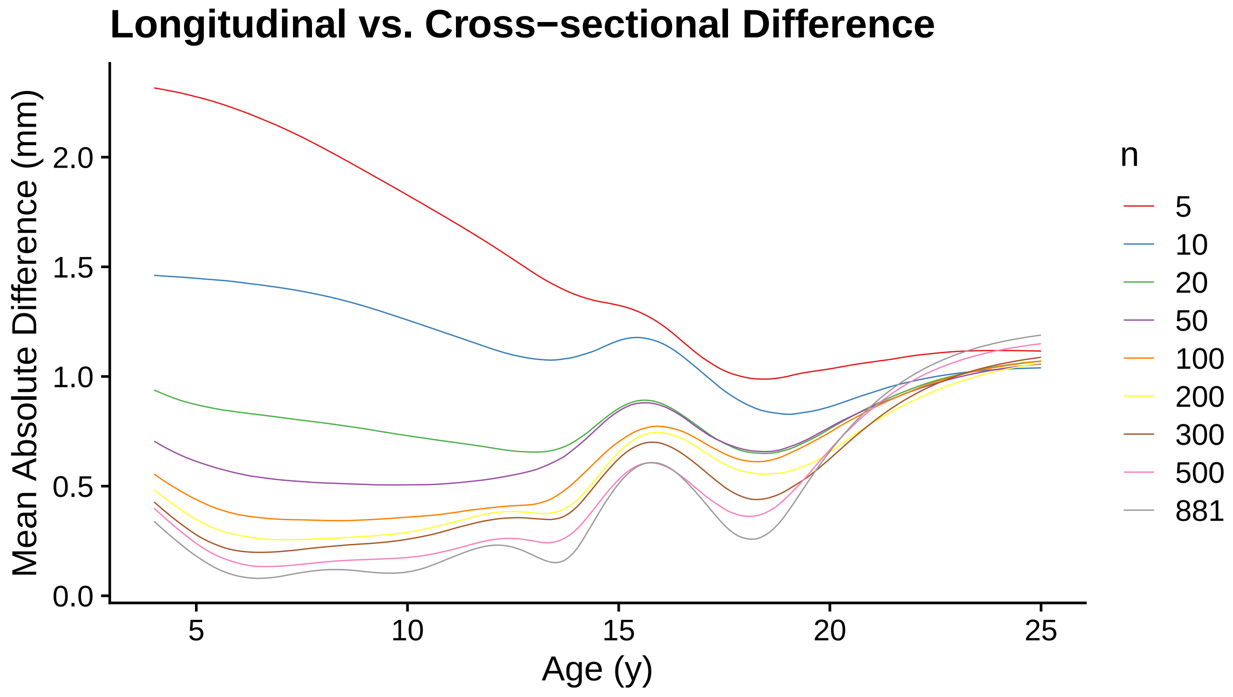
Longitudinal vs. Cross−sectional Difference
Mean Absolute Difference (mm)
2.0
1.5
1.0
0.5
0.0
5
10
15
20
25
Age (y)
n
5
10
20
50
100
200
300
500
881

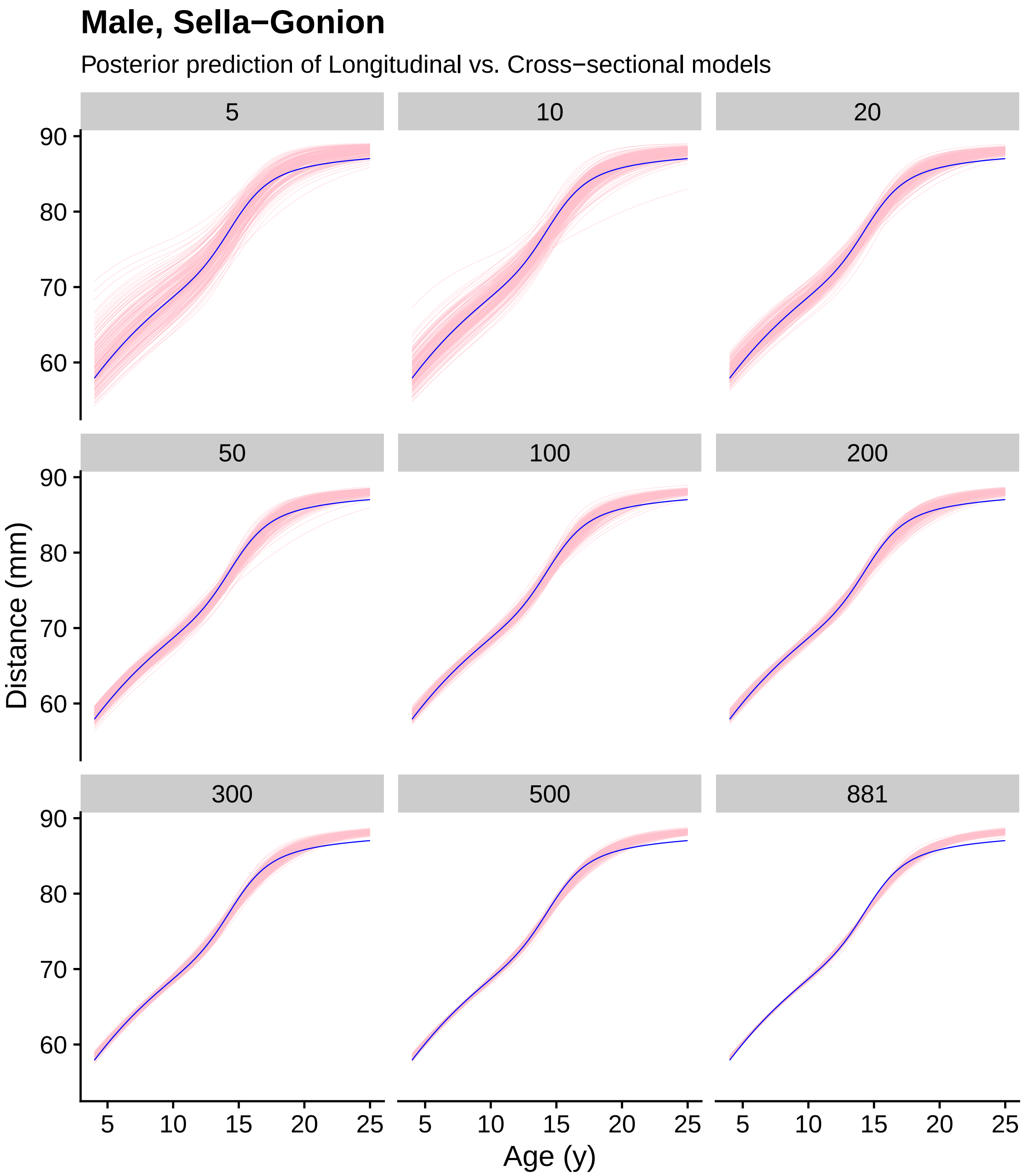
Male, Sella−Gonion
Posterior prediction of Longitudinal vs. Cross−sectional models
5
10
20
50
100
200
300
500
881
Distance (mm)
90
80
70
60
5
10
15
20
25
Age (y)

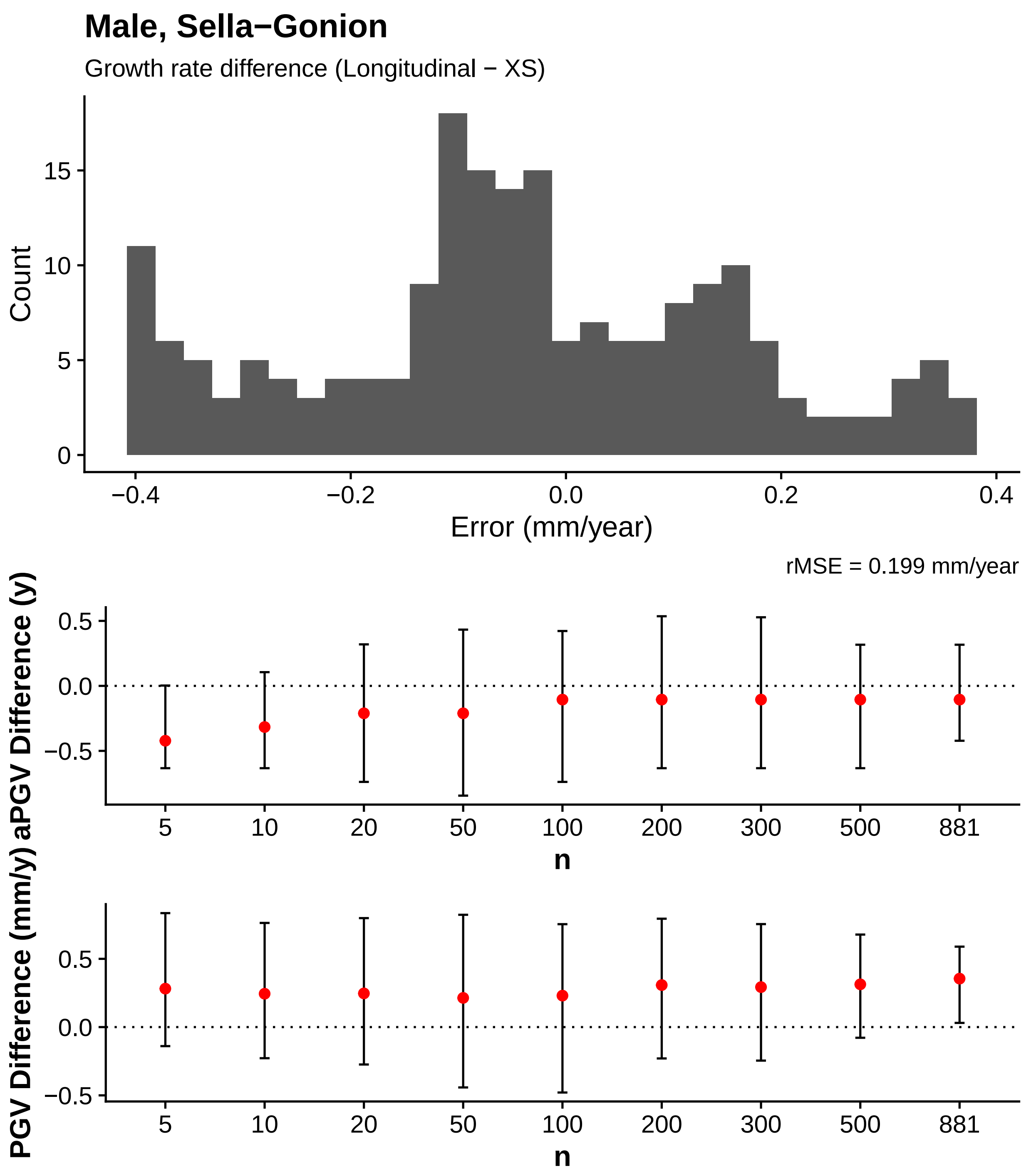
Male, Sella−Gonion
Growth rate difference (Longitudinal − XS)
Count
Error (mm/year)
rMSE = 0.199 mm/year
PGV Difference (mm/y) aPGV Difference (y)
n
5
10
20
50
100
200
300
500
881

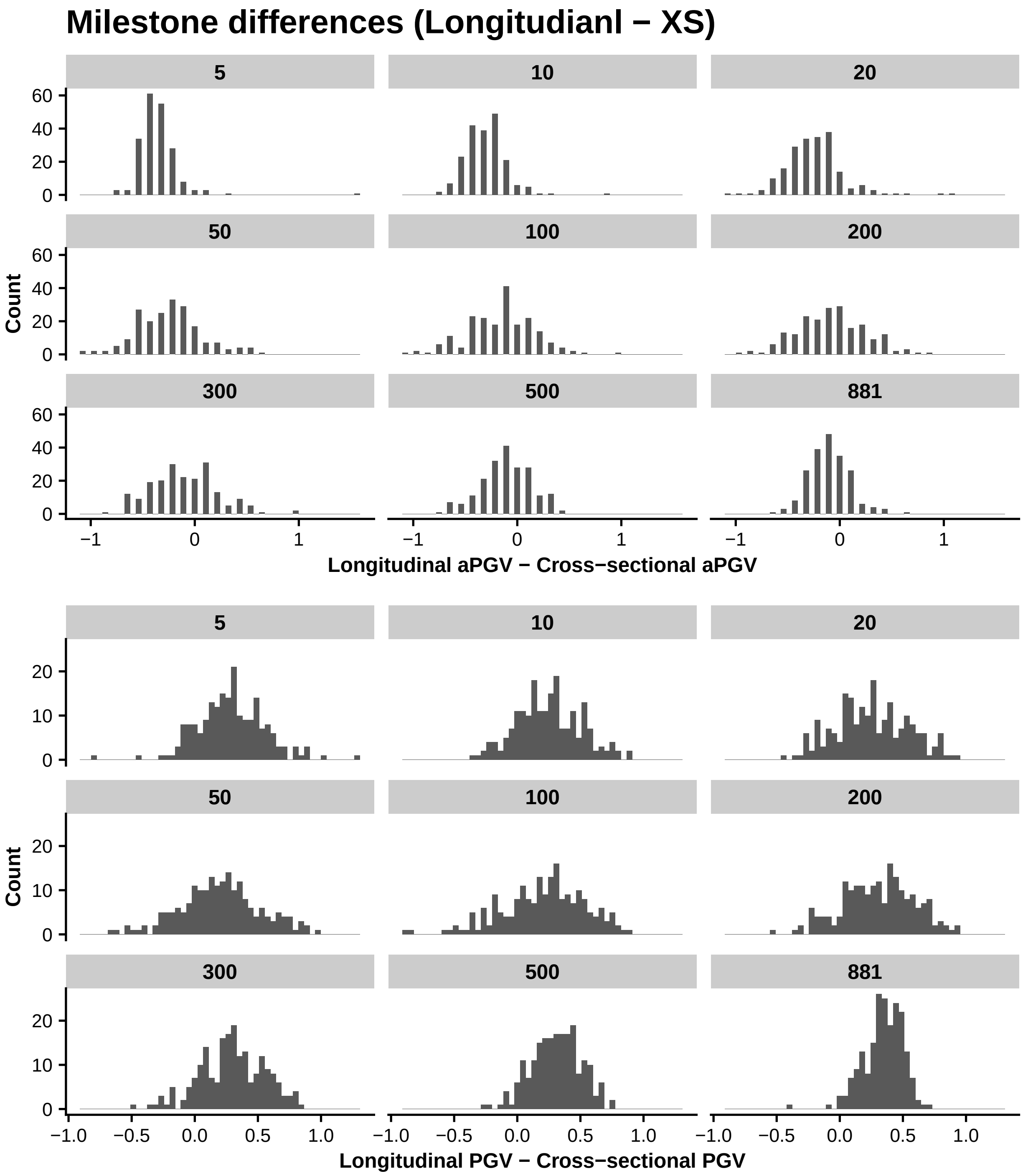

Milestone differences (Longitudianl − XS)
5
10
20
50
100
200
300
500
881
Count
Longitudinal aPGV − Cross−sectional aPGV
Longitudinal PGV − Cross−sectional PGV

26 Male, Sella-Nasion

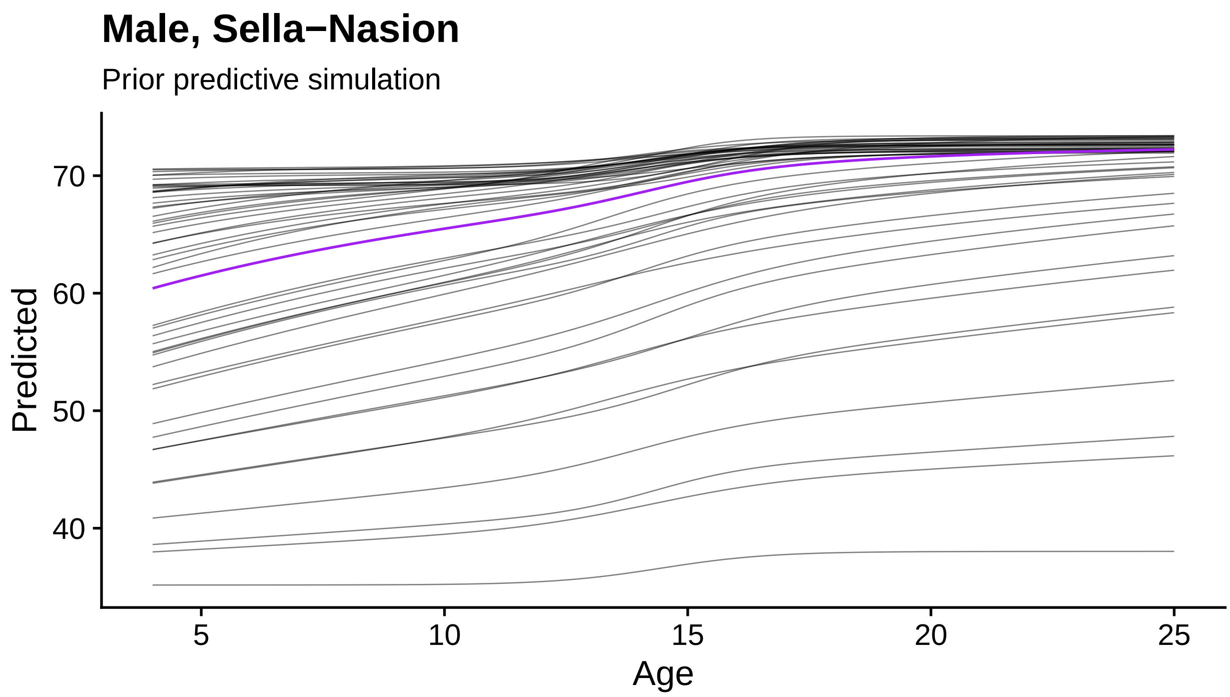

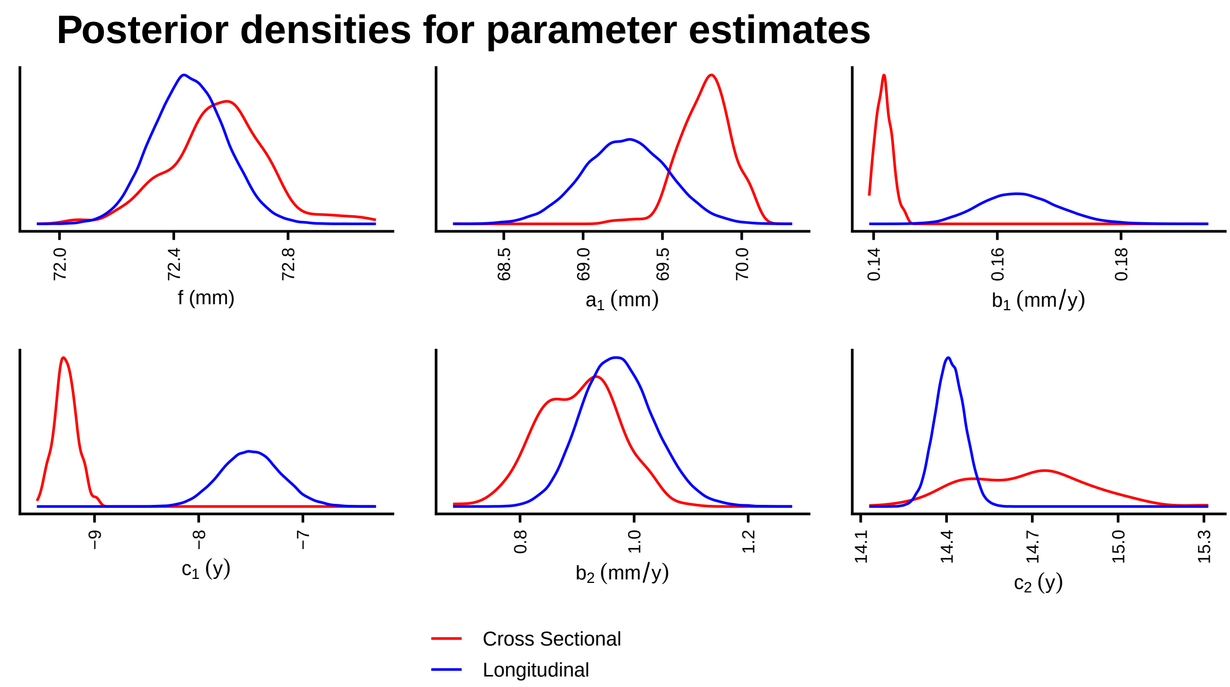

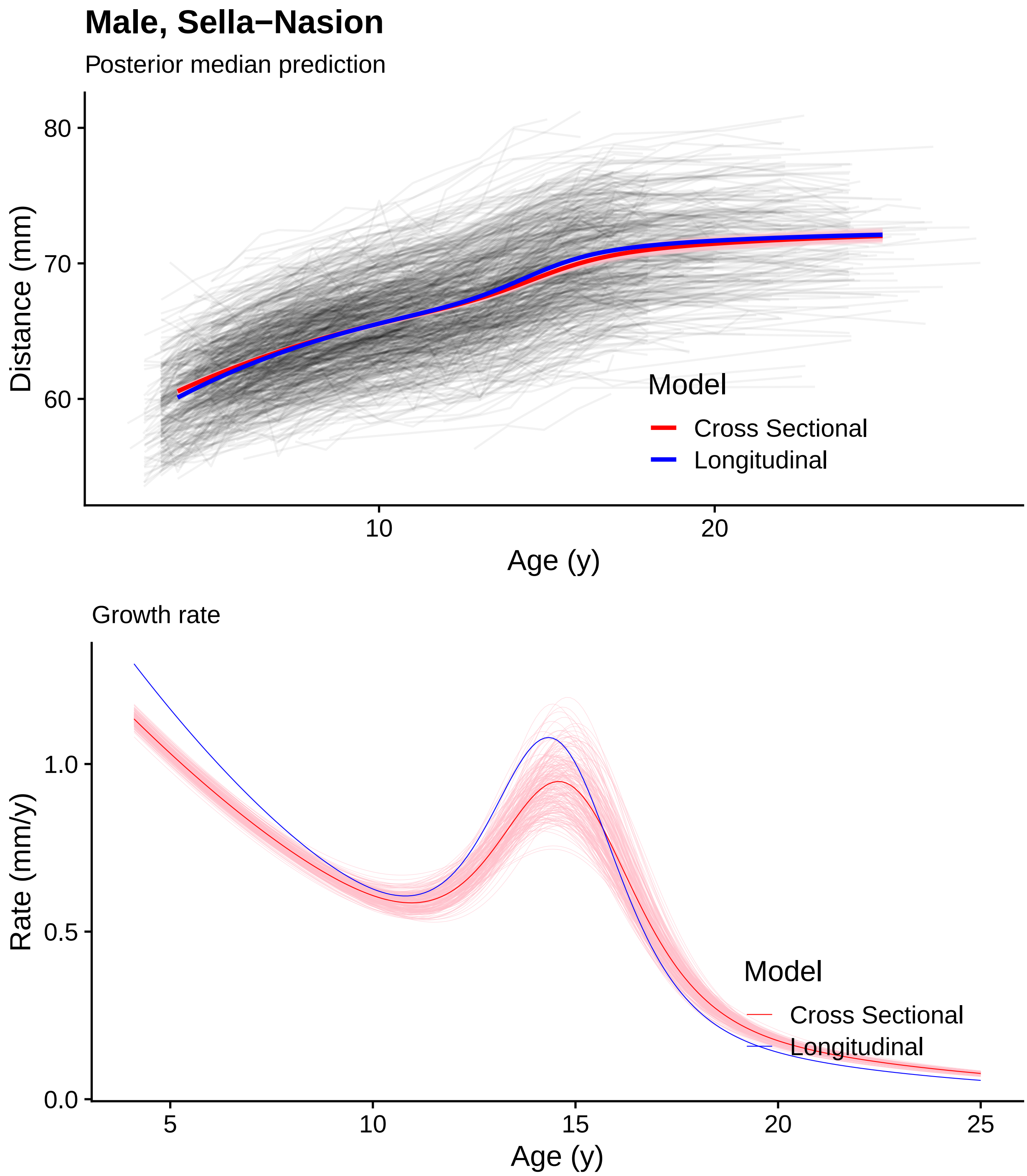
Male, Sella−Nasion
Posterior median prediction
Distance (mm)
80
70
60
10
20
Age (y)
Model
Cross Sectional
Longitudinal
Growth rate
Rate (mm/y)
1.0
0.5
0.0
5
10
15
20
25
Age (y)
Model
Cross Sectional
Longitudinal

Table 49: Longitudinal Model Summary

Parameter	Mean	Median	Std. Dev.	MAD	5%	95%	$\hat{r}$	Bulk ESS	Tail ESS
f	72.45	72.45	0.128	0.128	72.25	72.67	1	1908	5790
a1	69.26	69.27	0.269	0.271	68.82	69.70	1	9536	21523
b1	0.16	0.16	0.006	0.006	0.15	0.17	1	17215	24636
c1	-7.50	-7.50	0.286	0.288	-7.96	-7.02	1	18858	26280
b2	0.97	0.97	0.064	0.064	0.87	1.08	1	24694	30638
c2	14.41	14.41	0.052	0.052	14.33	14.50	1	75756	30417
sigma	0.96	0.96	0.008	0.008	0.95	0.97	1	69659	30908
sigma_ID	3.02	3.02	0.069	0.070	2.91	3.14	1	87904	30909

Table 50: Median Coefficients

Model	f	a_1	b_1	c_1	b_2	c_2	σ	σ_{ID}
Longitudinal	72.45	69.27	0.16	-7.50	0.97	14.41	0.96	3.02
Cross Sectional	72.56	69.78	0.14	-9.27	0.91	14.69	3.15	NA

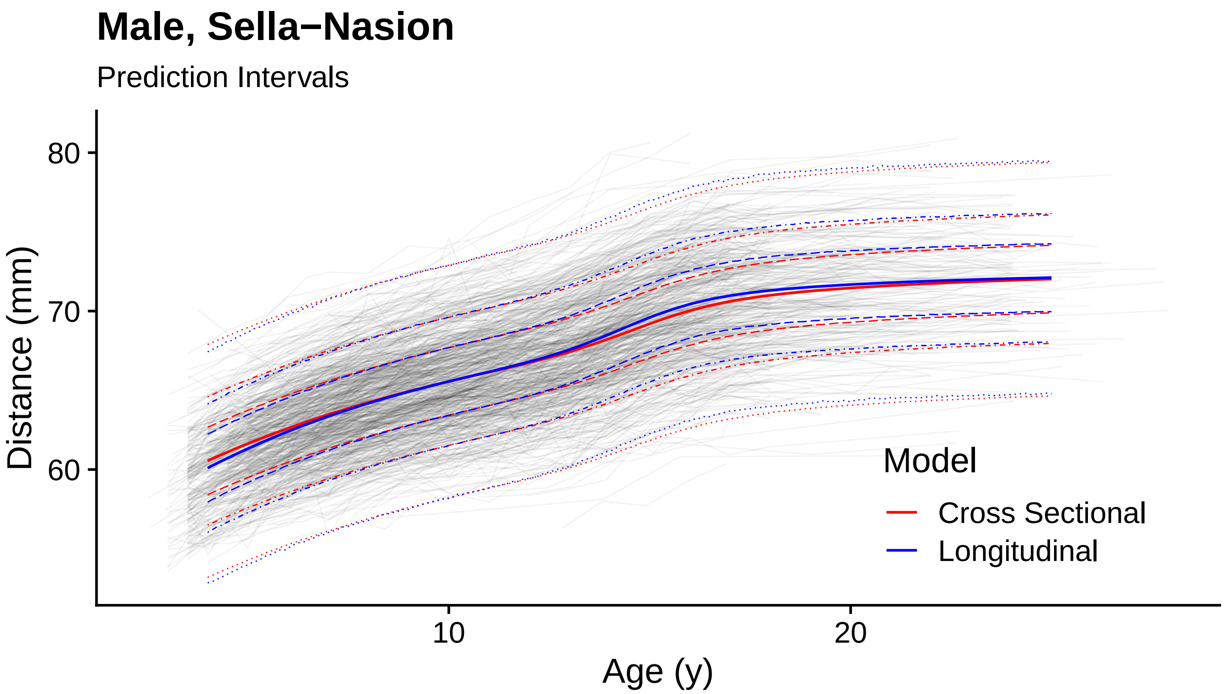
Male, Sella−Nasion
Prediction Intervals
80
70
60
Distance (mm)
10
20
Age (y)
Model
Cross Sectional
Longitudinal

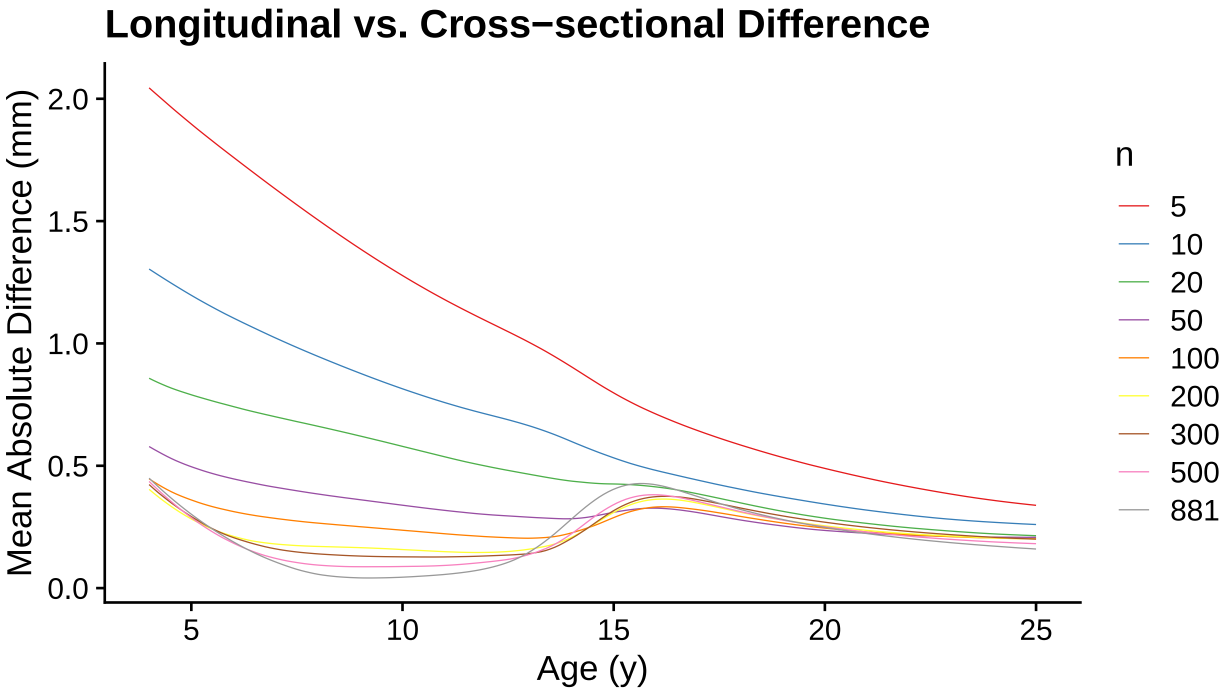
Longitudinal vs. Cross−sectional Difference
2.0
1.5
1.0
0.5
0.0
Mean Absolute Difference (mm)
5
10
15
20
25
Age (y)
n
5
10
20
50
100
200
300
500
881

Male, Sella−Nasion

Posterior prediction of Longitudinal vs. Cross−sectional models

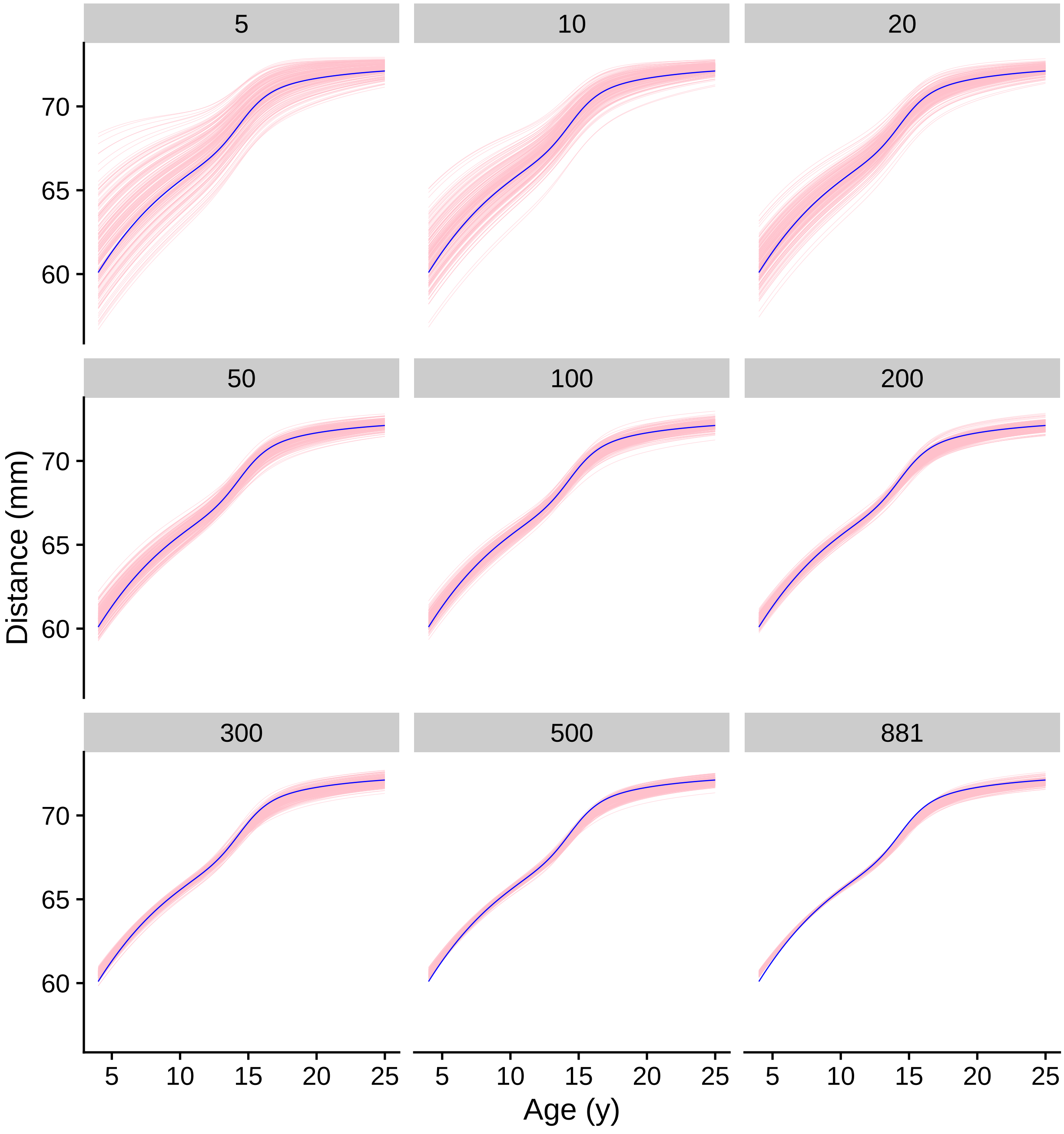

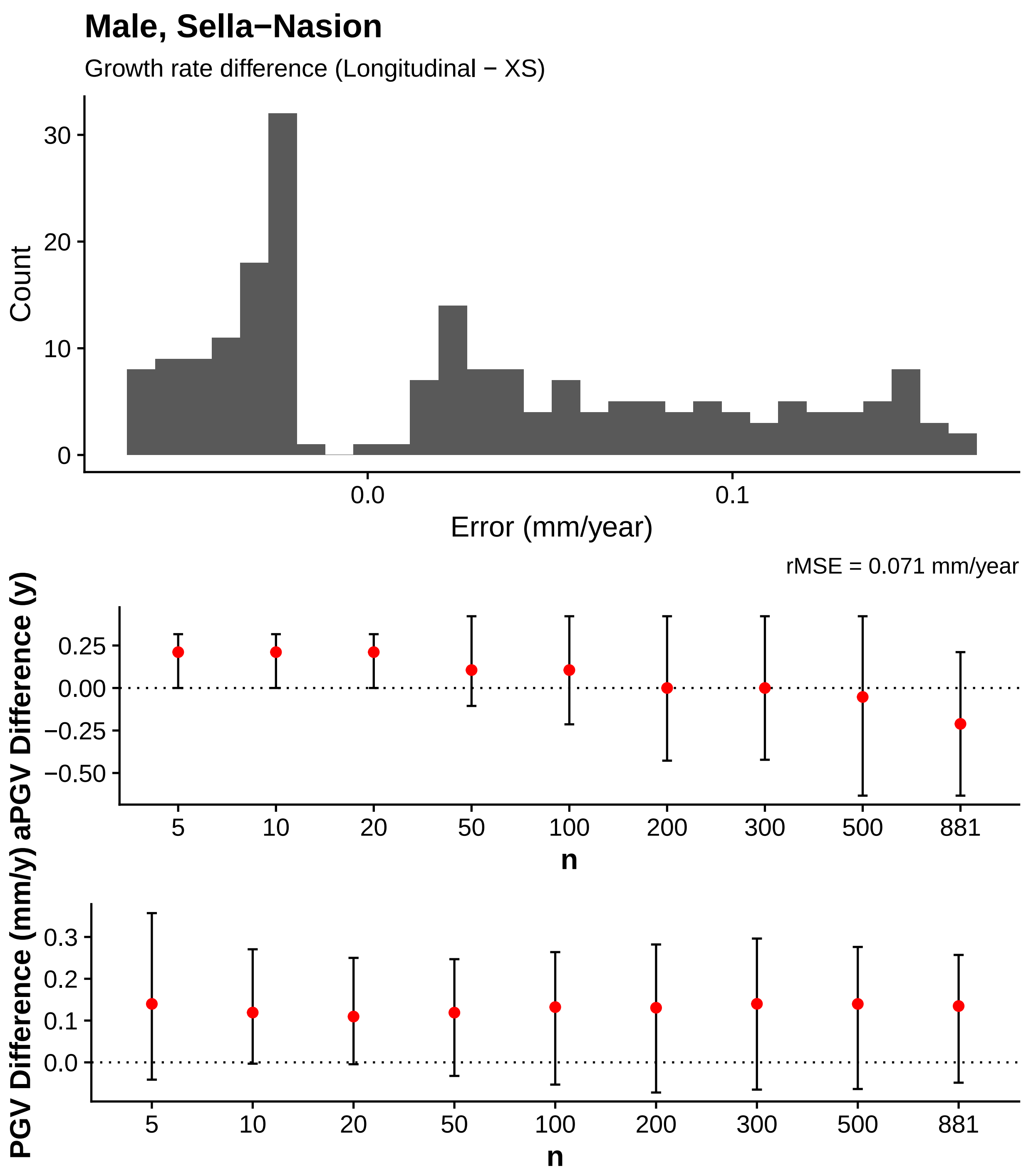

Male, Sella−Nasion
Growth rate difference (Longitudinal − XS)
Count
30
20
10
0
0.0
0.1
Error (mm/year)
rMSE = 0.071 mm/year
aPGV Difference (y)
0.25
0.00
−0.25
−0.50
5
10
20
50
100
200
300
500
881
n
PGV Difference (mm/y)
0.3
0.2
0.1
0.0
n

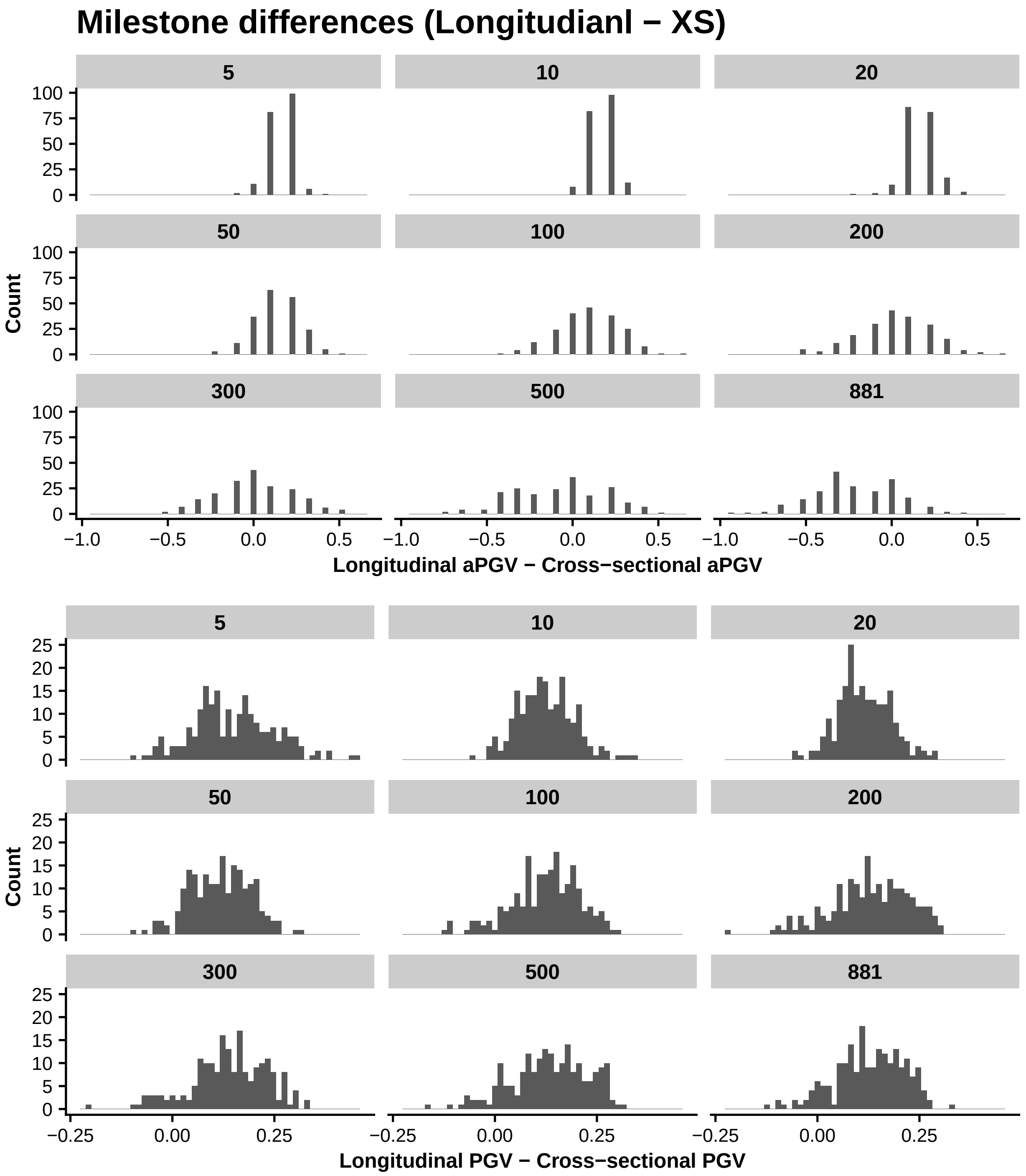

Milestone differences (Longitudianl − XS)
5
10
20
50
100
200
300
500
881
Count
Longitudinal aPGV − Cross−sectional aPGV
Count
Longitudinal PGV − Cross−sectional PGV
